# Identification of Differentially Expressed Transcripts and Pathways in Blood One Week and Six Months Following Implant of Left Ventricular Assist Devices

**DOI:** 10.1371/journal.pone.0077951

**Published:** 2013-10-21

**Authors:** Adam Mitchell, Weihua Guan, Rodney Staggs, Aimee Hamel, Sameh Hozayen, Neeta Adhikari, Suzanne Grindle, Snider Desir, Ranjit John, Jennifer L. Hall, Peter Eckman

**Affiliations:** 1 Lillehei Heart Institute, Department of Medicine, University of Minnesota, Minneapolis, Minnesota, United States of America; 2 Division of Biostatistics, University of Minnesota, Minneapolis, Minnesota, United States of America; 3 Division of Cardiology, Department of Medicine, University of Minnesota, Minneapolis, Minnesota, United States of America; 4 Lillehei Clinical Research Unit, University of Minnesota, Minneapolis, Minnesota, United States of America; 5 Department of Surgery, University of Minnesota, Minneapolis, Minnesota, United States of America; Tokai University, Japan

## Abstract

**Introduction:**

Continuous-flow left ventricular assist devices (LVADs) are an established therapy for patients with end-stage heart failure. The short- and long-term impact of these devices on peripheral blood gene expression has not been characterized, and may provide insight into the molecular pathways mediated in response to left ventricular remodeling and an improvement in overall systemic circulation. We performed RNA sequencing to identify genes and pathways influenced by these devices.

**Methods:**

RNA was extracted from blood of 9 heart failure patients (8 male) prior to LVAD implantation, and at 7 and 180 days postoperatively. Libraries were sequenced on an Illumina HiSeq2000 and sequences mapped to the human Ensembl GRCh37.67 genome assembly.

**Results:**

A specific set of genes involved in regulating cellular immune response, antigen presentation, and T cell activation and survival were down-regulated 7 days after LVAD placement. 6 months following LVAD placement, the expression levels of these genes were significantly increased; yet importantly, remained significantly lower than age and sex-matched samples from healthy controls.

**Conclusions:**

In summary, this genomic analysis identified a significant decrease in the expression of genes that promote a healthy immune response in patients with heart failure that was partially restored 6 months following LVAD implant.

## Introduction

The clinical care of patients with end stage heart failure has been transformed by the emergence of left ventricular assist devices (LVADs), which have been shown to improve patient survival and quality of life compared to medical therapy[1-3]. Contemporary continuous flow devices have been shown to have higher survival rates, improved quality of life, and fewer adverse effects as compared to the earlier pulsatile models[4-6].  In addition to the intended mechanical unloading of the left ventricle and improved systemic perfusion, there are several unintended effects of LVADs. These devices continually expose the patient’s blood to an intravascular foreign material, cause a high degree of shear stress on cells as they move through the device, and establish non-pulsatile blood flow with a nonphysiologic narrow pulse pressure. As a result, LVADs may have unrecognized immunologic or hematologic consequences. Among the most common complications associated with LVADs are infection, bleeding, and thrombosis[7].  

Several studies have examined mRNA expression profiles in myocardium after LVAD insertion using gene expression array technology[8-14] and one group studied expression changes in the peripheral blood mononuclear cell transcriptome using microarray technology 24 hours and 7 days following LVAD implant[15]. Nothing is known about how gene expression changes in the peripheral blood of patients after sustained LVAD therapy. Peripheral blood is an attractive biological “compartment” to study due to its noninvasive nature, and may be of particular interest in improving understanding of mechanisms operative in the delicate balance between thrombosis and hemostasis in this patient population. RNA-sequencing has emerged as a tool which uses high throughput sequencing to survey the whole genome expression profile - the transcriptome[16,17]. 

In this study we use RNA-sequencing to identify differentially expressed genes in the peripheral blood of stage D heart failure patients prior to LVAD implant, then 7 days and 6 months postoperatively. We hypothesized that this approach would reveal novel genes and pathways (both acute and chronic) associated with left ventricular remodeling and improved systemic perfusion.

## Methods

### Patient Selection

Ambulatory adults scheduled for clinically indicated LVAD implant for stage D heart failure were eligible to participate in this study. Written consent was obtained from participants and the University of Minnesota Institutional Review Board approved the study. Patient characteristics including INTERMACS profile, preoperative lab data, and medications are shown in Table 1. Seven participants received the HeartMate II (Thoratec Corporation, Pleasanton, CA), and 2 received the HVAD (HeartWare Inc., International, Framingham, MA). Blood was drawn into a 2.5mL PAXgene Blood RNA tube (Qiagen, Valencia, CA) prior to implant, 7 days after implant and 6 months after implant. For comparison, blood was drawn from four patients undergoing non-LVAD cardiac surgery prior to and 7 days postoperatively, as well as from six age-matched healthy volunteers without heart failure (Table S1 in File S1). Per the manufacturer's instructions, tubes were incubated at room temperature for a minimum of 2 hours, frozen at -20°C for a minimum of 24 hours, and then transferred to -80°C storage until analysis.

**Table 1:** Patient characteristics, INTERMACS level, preoperative lab data, and medications.

**Patient ID**	**1**	**2**	**3**	**4**	**5**	**6**	**7**	**8**	**9**
Gender	M	M	M	M	M	M	M	M	F
Age	62	66	55	46	64	76	54	71	51
Race	C	C	C	C	C	C	C	C	AA
HF Etiology	I	I	I	NI	I	I	NI	I	NI
BMI	34.2	25.7	30.1	28.1	27.1	23.9	31.3	36.5	22.0
INTERMACS	5	5	6	6	4	5	6	5	5
Diabetes	-	+	-	-	+	-	-	+	-
Device	HW	HW	HMII	HMII	HMII	HMII	HMII	HMII	HMII
BUN (mg/dL)	31	38	31	18	49	27	23	52	30
Creatinine (mg/dL)	1.41	1.56	2.07	1.29	1.81	1.51	0.93	1.58	1.09
NA (mmol/L)	134	139	139	141	126	139	133	143	143
K (mmol/L)	3.4	2.7	4.5	3.8	3.8	4.0	3.1	4.6	3.6
Albumin (g/dL)	3.9	3.5	4.2	3.6	3.9	3.9	4.6	4.6	4.3
Bilirubin (mg/dL)	0.4	1.8	0.6	0.6	0.9	1.5	1.6	0.7	1.2
WBC (10^9^/L)	5.8	7.7	6.2	5.6	4.6	4.3	8.7	11.1	5.8
Beta blocker	+	+	+	+	+	+	+	+	+
ACE/ARB	+		+	+		+	+	+	+
Aldosterone antagonist		+	+	+		+	+		+
Diuretic	+	+	+	+	+	+	+	+	+
Statin		+	+		+	+		+	
Aspirin	+	+	+	+	+	+	+	+	+
Amiodarone or Dronederone	+	+							
Warfarin		+			+	+	+		
LMW Heparin									+
Digoxin	+			+		+	+		+
Plavix		+							

M, male; F, female; C, caucasian; AA, African-American; I, ischemic cardiomyopathy; NI, non-ischemic cardiomyopathy; HW, Heartware; HMII, Heartmate II

### cDNA library preparation for RNA Sequencing

RNA extraction from whole blood was carried out per the manufacturer’s instructions using the PreAnalytiX PAXgene Blood RNA Kit, Version 2 from Qiagen. RNA was isolated in two 40 ul elution steps. Extraction yields were determined by RNA quantitation on the NanoDrop 8000 spectrophotometer.

Globin reduction of the total RNA was performed using the GLOBINclear Human Kit from Ambion per the manufacturer’s instructions with the exception of adjustments made in order to process samples in a BioRad96 well plate instead of individual tubes. Sample quality and quantity were assessed using both the Quanit-iT RiboGreen RNA Assay (Life Technologies) and the Agilent RNA 6000 Nano Kit on the Agilent 2100 Bioanalyzer (Agilent Technologies). 

Libraries were created using the Illumina TruSeq RNA Sample Preparation kit. The libraries were validated and quantified using an Agilent High Sensitivity chip (Agilent Technologies), Pico Green assay (Life Technologies) and KAPA qPCR (KAPA BioSystems). All clean-up steps performed, per the Illumina protocol, were SPRI bead based (Beckman Genomics).

The Illumina cBOT was used for cluster generation per the manufacture’s protocol. During cluster generation the template was immobilized to a random oligo lawn on the surface of the flow cell and then amplified, linearized, blocked and then the sequencing primer was hybridized on. After cluster generation the flow cell was loaded onto the Illumina HiSeq 2000 per the manufacture’s protocol. The run parameters were paired end, 100 cycles with at least 10 million reads achieved for each library. The Illumina SBS 200 Cycle Sequencing Kit was used for sequencing.

### RNA Sequencing

Libraries were sequenced to 100 nucleotide lengths on Illumina HiSeq2000 using RTA (real-time analysis) for image analysis, base calling, and data filtering. Casava 1.8.0 was used to demultiplex and generate fastq files for each patient index. Image analysis, base calling, and base call quality calibration were done using the Real Time Analysis software (Illumina). Fastq files were examined using fastqc. GC contents were similar to expected distributions, there were no significantly over-expressed pentamers, and the sequence quality across the majority of sequences was deemed acceptable for assembly.

### Data Analysis

Sequences were mapped to the human Ensembl GRCh37.66 genome assembly using TopHat[18] with an approach mapping concordant pairs to the transcriptome only. Annotations were assigned using the Ensembl gene transfer format (GTF) reference. Cufflinks[19,20] was used to generate preliminary FPKM (fragments per kilobase of exon model per million mapped reads) values for each patient sample at pre-LVAD, 7 days post LVAD, and 180 days post LVAD. All patient Cufflinks results were combined using Cuffmerge to create a single reference GTF file. Final FPKM values were generated with Cuffdiff for each patient comparing expression prior to and 7 days after LVAD implant. This was repeated to generate FPKM values comparing expression prior to and 180 days after LVAD implant. All called gene FPKM values, along with upper and lower confidence interval bounds for each patient sample, were collected for subsequent statistical analysis.

The number of sequence reads produced ranged from 25,363,852 to 71,209,508 across all samples. Between 43.58% and 77.46% of the total transcripts per library mapped to the genome. In the 7 day and 180 day comparisons respectively, accepting alignments to known transcripts resulted in a total of 10,671 and 10,690 unique locus alignments from the reference sequences corresponding to 12,845 and 12,794 unique gene symbols.

### Statistical Analysis

We used a paired t-test to compare expression levels (FKPM) prior to surgery and 7 days after, as well as prior to and 180 days after surgery. In order to identify genes with different change patterns between the two patient groups, we further used unpaired t-test to compare the change of gene expression levels from pre-LVAD to 7 days after surgery between the bleeders and non-bleeders. To control for multiple hypothesis tests, q-values were calculated using the false discovery rate (FDR) approach with 0.05 chosen as a cutoff for statistical significance [21].

### Confirmation of Gene Expression Analysis

**Figure 1:**
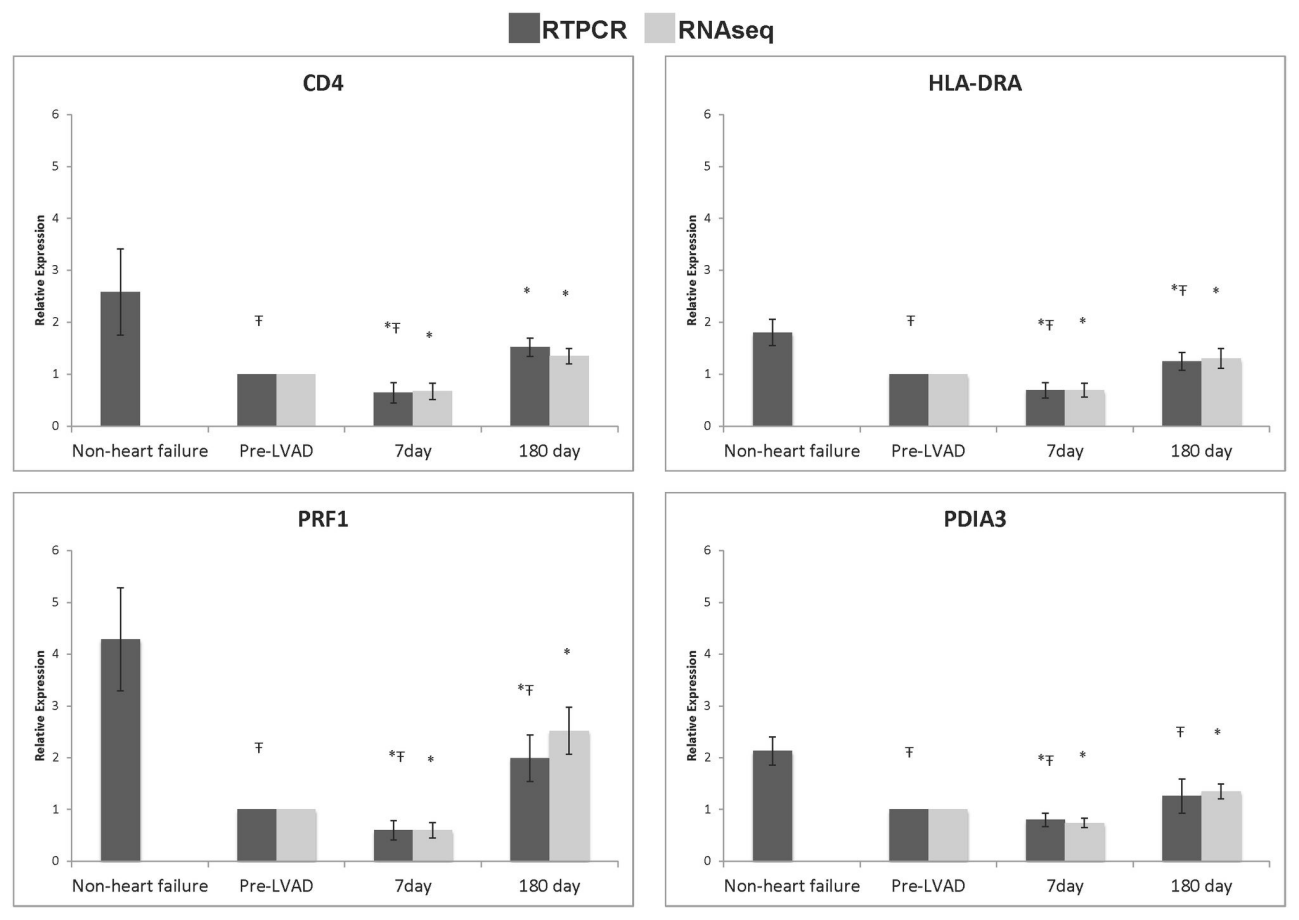
Relative expression of 4 genes related to cellular immune function in heart failure patients before and after LVAD implant and age matched control subjects with normal cardiac function. Expression of CD4, HLA-DRA, PRF1, and PDIA3 was evaluated with RT-qPCR and RNA-sequencing in patients with end-stage heart failure before LVAD, 7 days post LVAD, and 180 days post LVAD. Expression in age-matched subjects with normal cardiac function was measured with RT-qPCR alone. Ŧ p < 0.05 compared to non-heart failure patients, * p < 0.05 compared to pre-LVAD.

**Figure 2:**
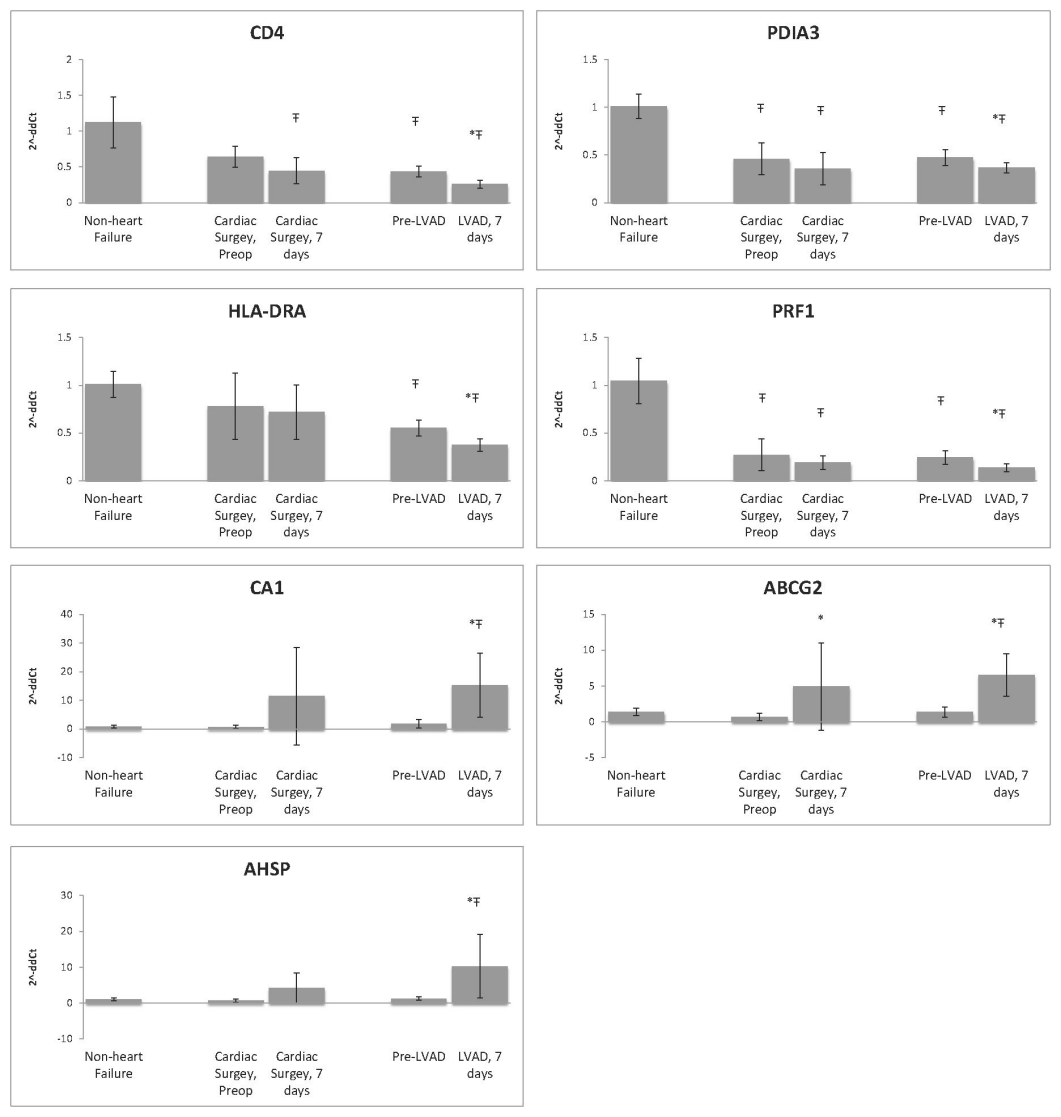
Relative expression (mean 2^- ΔΔCt^) of select genes among six individuals without heart failure, four patients that underwent non-LVAD cardiac surgery (prior to and 7 days after surgery), and nine end-stage heart failure patients (prior to and 7 days after LVAD implant) as measured by RT-qPCR. Ŧ p < 0.05 compared to non-heart failure patients, * p < 0.05 compared to pre surgery (non-LVAD cardiac surgery or LVAD implant).

RNA was extracted as outlined above and transcribed to cDNA (RT Kit, Clonetech). Taqman primer probe assays were used with the ABI7900HT Fast Real-Time PCR System as previously described (Table S8 in File S1)[22]. All nine subjects were selected from the initial RNAseq analysis, as well as four age-matched patients who underwent non-LVAD cardiac surgery, and six age-matched individuals without heart failure (Table S1 in File S1). Duplicates of each sample were run and normalized to hypoxanthine phosphoribosyltransferase 1 (HPRT1) expression. Target amplification and detection were performed on samples and controls in the same thermal cycling reaction in replicated fashion, allowing for minimization of experimental variability and calculation of ΔCt based on the corresponding reference control, hypoxanthine guanine phosphoribosyl transferase (ie, Target^ΔCtHPRT^). Relative expression at 7 and 180 days post LVAD were calculated and compared to that calculated from RNA-seq FPKM values (Figure 1 and Table S2 in File S1). Mean expression, calculated as mean 2^-ΔΔCt^, of several genes was compared between individuals without heart failure, patients that underwent non-LVAD cardiac surgery (prior to and 7 days after surgery), and end-stage heart failure (prior to and 7 days after LVAD implant (Figure 2).

### Pathways and upstream regulators analysis

Differentially expressed transcripts (FDR < 0.10) for each analysis were uploaded into Ingenuity Pathway Analysis software (Ingenuity® Systems, www.ingenuity.com). Ingenuity identifies canonical signaling pathways overrepresented in the dataset by calculating the fraction of transcripts differentially expressed in a known signaling pathway. Fisher’s exact test is used to calculate a p‐value that determines the probability the association between genes in the dataset and the canonical pathway is explained by chance alone. Ingenuity also identifies putative activation or inhibition of upstream regulators that explain observed gene expression changes in the dataset. A p-value is generated based on overlap between genes in the dataset and known targets affected by an upstream regulator, and a z-score is used to infer likely activation states of a regulator based on a model of known downstream effects.

### Supplemental Methods

Final tables of significant genes were refined by removing any cases of genes that had duplicate alignments in haplotype and chromosome sequences in the reference genome. In these cases the chromosomal location or statistically best haplotype was retained. Transcripts with a median FPKM of <1 at both time points were also removed from the final tables.

## Results

Blood samples were obtained from each of the 9 end-stage heart failure patients at the following time points: prior to LVAD, 7 days post implant, and 6 months post implant. RNA-sequencing was used to identify differentially expressed transcripts. Statistically significant (FDR < 0.05) transcripts with a fold change greater than 1.5 after LVAD placement at 7 days and 6 months are shown in Tables S3 and S4, respectively, in File S1. All sequence data is publicly available in the Gene Omnibus Expression (GEO) database under accession number GSE46665.

### 7 Days After LVAD Implantation

#### Canonical Signaling Pathways

Canonical signaling pathways that were altered 7 days after LVAD placement were identified using Ingenuity. Table S5 in File S1 outlines the significance of several of the canonical pathways affecting Cellular Immune Response, Cytokine Signaling, Apoptosis, and Cellular Growth, Proliferation, and Development. Transcripts in the interrelated canonical pathways of T cell biology were almost uniformly down-regulated 7 days post implant. Four transcripts related to T cell biology including CD4 (a marker of T helper cells), HLA-DRA (presents antigens to CD4 positive cells), PRF1 (perforin1)(crucial effector molecule in T cell mediated cytolysis) and PDIA3 (protein disulfide isomerase family A, member 3)(contributes to antigen presentation) were assessed by RT-qPCR (see Figure 1). All transcripts showed the same pattern: decreased transcript expression in the blood in patients with heart failure compared to non-failing controls (p < 0.05), a further reduction in expression 7 days post LVAD (p < 0.05) and an increase in expression at 6 months (p < 0.05). To determine if the change in gene expression at 7 days was due to the LVAD implant or due to the surgery, we measured several transcripts in a set of patients who underwent cardiac surgery but did not receive an LVAD. Transcripts related to cellular immune function (CD4, HLA-DRA, PAIA3, and PRF1) that were significantly decreased at 7 days in LVAD patients compared to pre-op expression, were not significantly different in the non-LVAD surgical controls. However, the trend for expression levels decreasing at 7 days was similar. Secondly, expression of ABCG2 increased in LVAD patients at 7 days and also increased significantly in non-LVAD cardiac surgery patients at 7 days compared to preoperative levels. 

Also noted at 7 days in the pathways analysis was an up-regulation of transcripts related to Glutathione Redox Reactions I (Table 2), suggesting the induction of a significant protective response to oxidative stress. Transcripts related to Heme Biosynthesis, including alpha hemoglobin stabilizing protein (AHSP) were also increased at 7 days. This was intriguing given that the majority of published work shows an association of anemia with implantation of a LVAD[23,24] (Table S2 in File S1, Table S5 in File S1). 

**Table 2:** Selected canonical signaling pathways affected at 7 days and 6 months after LVAD implant.

**Ingenuity Canonical Pathways**	**7 days post LVAD**		**6 months post LVAD**		**Comparison**
	**p**	**Genes**	**p**	**Genes**	**Genes that change direction**
Interferon Signaling	0.0010	**IFIT3, IFIT1, RELA, JAK1, IFI35, STAT1, IFNAR1, IRF1**	0.0500	*BAX*, **IFNAR1**, *PSMB8*, *TYK2*	
Activation of IRF by Cytosolic Pattern Recognition Receptors	0.0135	**IFIH1**, **RELA**, **RIPK1**, **LTA**, **DDX58**, *SIKE1*, **STAT1**, **IFIT2**, **IFNAR1**	0.0363	**MAP2K4**, **TRAF3**, *PPIB*, **MAPK9**, *IKBKE*, *IRF3*, **ATF2**, **TANK**, *IRF7*, *IKBKG*, *RIPK1*, *IKBKAP*, *PIN1*, *NFKBIB*, **TNF**, **IFNAR1**	RIPK1
CTLA4 Signaling in Cytotoxic T Lymphocytes	0.0001	**CD247**, *AP2B1*, **HLA**-**DOA**, **PPP2R5D**, **CD4**, **AP1S2**, *PPP2R5B*, **HLA**-**DQA1**, **HLA**-**DRB1**, *AP2S1*, **PPP2CB**, **PIK3C3**, **HLA**-**DRA**, *CLTCL1*, *PIK3R6*, **PPP2R5C**	0.0010	*FYN*, *CD3E*, **PIK3R1**, *CD4*, *CLTB*, *PIK3R5*, **TRAT1**, *HLA*-*DRB1*, *PPP2R3B*, *LCK*, **PIK3C3**, **PIK3CG**, HLA-DRA, **ATM**, **AKT2**, *PTPN6*, *AP1M1*, *CLTC*, *CD3D*, *PPP2R1A*, **PTPN11**, *CLTA*, *PPP2R2B*, *ZAP70*, *FCER1G*, **AP1G1**, **LCP2**	HLA-DRB1, HLA-DRA
NF-KB Activation by Viruses	0.0389	***RAF1***, **RELA**, **CCR5**, **RIPK1**, **CD4**, **PIK3C3**, ***PIK3R6***, ***ITGAV***, **ITGAL**	0.0015	*SIGIRR*, **AZI2**, **TRAF3**, **TGFBR1**, **PIK3R1**, **UBE2N**, **TLR8**, **PIK3R5**, **TNFAIP3**, *HRAS*, **KRAS**, **TANK**, **TGFBR2**, *IKBKG*, *LCK*, **TLR10**, **MAP3K7**, *PIK3C3*, **PIK3CG**, **CSNK2A1**, **TDP2**, *NFKBIB*, **CASP8**, **ATM**, *MAP3K14*, **AKT2**, *RRAS*, *RELB*, **MALT1**, TAB **3**, **TLR2**, **TLR4**, *RIPK1*, **BCL10**, *ZAP70*, *FCER1G*, **MAP3K8**, **TIRAP**, **TNF**, **MAP3K3**, *CARD11*, **IRAK4**, **PRKCB**	RIPK1, PIK3C3
Antigen Presentation Pathway	0.0003	**HLA-DOA, PDIA3, HLA-DRB3, HLA-DRA, HLA-DQA1, HLA-DRB1, CD74, HLA-DPB1, HLA-DPA1**	0.0102	*HLA-DRB4, HLA-A, PDIA3, HLA-DRB3, HLA-DRA, HLA-B, HLA-DRB1, PSMB8, CD74, HLA-F, HLA-DPB1, PSMB6*	HLA-DRB3, HLA-DRA, HLA-DRB1, CD74, HLA-DPB1
OX40 Signaling Pathway/T-Cell Survival	0.0009	**CD247**, *BCL2L1*, **RELA**, **HLA-DOA**, **CD4**, **HLA-DRB3**, **HLA-DRA**, **HLA-DQA1**, **HLA-DRB1**, **HLA-DPB1**, **HLA-DPA1**	0.0045	**MAP2K4**, **TNFSF4**, **TRAF3** *, TNFRSF4, CD3E, HLA-A, CD4, HLA-DRB1*, **MAPK9** *, CD3D, HLA-DRB4, HLA-DRB3, HLA-DRA, HLA-B, FCER1G, HLA-F, NFKBIB, HLA-DPB1*	CD4, HLA-DRB3, HLA- DRB1, HLA-DPB1
T Helper Cell Differentiation	0.0263	**STAT4, CD40LG, HLA-DOA, HLA-DRA, HLA-DQA1, HLA-DRB1, STAT1, TBX21, GATA3**	0.1432	**IL6ST**, *IL2RG*, **TGFBR1**, *IL12RB1*, **IL6R**, HLA-*DRB1*, **STAT3**, *TBX21*, **TGFBR2**, **ICOS**, *TGFB1*, HLA-DRA, *FCER1G*, **IL2RA**, **TNF**	TBX21 HLA-DRA
**Ingenuity Canonical Pathways**	**7 days post LVAD**		**6 months post LVAD**		**Comparison**
	**p**	**Genes**	**p**	**Genes**	**Genes that change direction**
Allograft Rejection Signaling	0.0062	**PRF1, CD40LG, HLA-DOA, HLA-DRB3, HLA-DRA, HLA-DQA1, HLA-DRB1, HLA-DPB1, HLA-DPA1**	0.0525	*HLA-A,HLA-DRB1, IGHG1, PRF1, IGHG3, HLA-DRB4, HLA-DRB3, HLA-DRA, FCER1G, HLA-B, IGHG4, HLA-F, HLA-DPB1*, **TNF** *, GZMB*	PRF1, HLA-DRB3, HLA-DRA, HLA-DRB1 HLA-DPB1
Glutathione Redox Reactions I or II	0.0093	*MGST1, GPX1, GPX4, PRDX6*	0.0275	**GSR, GLRX**	
IL-15 Signaling	0.0135	*BCL2L1*, *RAF1*, **RELA**, **STAT5A**, **JAK1**, *PIK3C3*, **PIK3R6**, *MAPK13*, **IL2RB**	0.0178	**RAF1**, *IL2RG*, **AKT2**, *RRAS*, **PIK3R1**, *TYK2*, **PIK3R5**, *HRAS*, *KRAS*, **STAT3**, *RAC3*, *LCK*, **MAPK14**, *PIK3C3*, **PIK3CG**, **STAT5B**, **ATM**	RAF1
14-3-3-mediated Signaling	0.0224	*RAF1* **, YWHAG, PDIA3, YWHAB**, *TUBB2A* **, MAP3K5, TUBB, TUBA1B, YWHAQ, PIK3C3**, *PIK3R6*, *SNCA*, *AKT1S1*	0.0008	**MAP2K4, TSC1, RAF1, BAD, PDIA3, PIK3R1, PIK3R5, HRAS, KRAS, MAP3K5, PLCH2**, *TUBB4A* **, PIK3CG, PIK3C3, PLCL1, PDCD6IP, PLCD4, ATM**, *TUBB1* **, AKT2, RRAS**, *TUBB4B*, *TUBG1* **, MAPK9, VIM, BAX, PLCL2, CBL, TUBA3C/TUBA3D, CDKN1B, TNF, PRKCB**	RAF1
Cytotoxic T Lymphocyte-mediated Apoptosis of Target Cells	0.0054	**CD247, PRF1, HLA-DOA, HLA-DRB3, HLA-DRA, HLA-DQA1, HLA-DRB1, HLA-DPB1, HLA-DPA1**	0.0039	**CASP3** *, CD3E, CYCS, HLA-A*, **APAF1** *, HLA-DRB1, CD3D, PRF1, HLA-DRB4, HLA-DRB3, HLA-DRA, FCER1G, HLA-B*, **CASP8** *, HLA-F, HLA-DPB1, GZMB*	HLA-DRB3, HLA-DRA, HLA-DRB1, HLA-DPB1
RAN Signaling	0.0003	**KPNB1, KPNA3, CSE1L, RANBP2, RAN, IPO5**	0.0363	**KPNA3**, **KPNA4**, *CSE1L*, *RANGAP1*, *RAN*, *RANBP1*	CSE1L
VEGF Signaling	0.0437	*EIF1AY*, *VEGFA*, *BCL2L1*, *RAF1*, **PIK3C3**, *PIK3R6*, **VEGFB**, **EIF2B3**, **ELAVL1**, *PGF*	0.0045	**MAP2K4**, **TNFSF4**, **TRAF3** *, TNFRSF4, CD3E, HLA-A, CD4, HLA-DRB1*, **MAPK9** *, CD3D, HLA-DRB4, HLA-DRB3, HLA-DRA, HLA-B, FCER1G, HLA-F, NFKBIB, HLA-DPB1*	
Cardiac Hypertrophy Signaling	-	-	0.0035	**MAP2K4, RAF1, TGFBR1**, *MAP3K11* **, PIK3R1**, *HRAS* **, KRAS**, *PLCH2* **, TGFBR2**, *MAP3K10* **, GNB4**, *RHOG*, *GNA15*, *TGFB1* **, PIK3CG, GNA13, PLCL1, ATM, MAP3K2**, *MAP3K14*, *RRAS* **, IL6R, PLCL2**, *CACNA1A* **, ATF2**, *MYL12A* **, RHOQ**, *EIF2B5*, *FNBP1*, *EIF2B4*, *PDIA3*, *RHOT2* **, PIK3R5**, *MAP3K5*, *EIF2B2* **, MTOR, MAP3K7**, *PIK3C3* **, SOS1**, *PLCD4*, *MAP3K6* **, MAPKAPK3, MEF2A, GNAQ, MAPK9, ROCK1, MAPK14, PRKAR2B, MAP3K8, MAP3K3, ADCY7, PRKAR1A**	

Differentially expressed transcripts with an FDR <0.10 at each time point were analyzed with Ingenuity Pathways Analysis (IPA). The fraction of genes in the dataset involved in known signaling pathways is determined and a p-value is calculated to estimate the likelihood the finding is due to chance alone. The transcripts within each pathway are shown with **Bold** = down, *italic* = up.

#### Upstream Regulators Analysis

Ingenuity’s Upstream Regulators Analysis was used to identify upstream factors that explain the changes in expression among transcripts differentially regulated 7 days after LVAD with a FDR < 0.1. Two major predicted and validated findings of the upstream regulators analysis included increased expression of SOCS1 (up-regulated 1.6 fold), a regulator of cytokine signaling through the JAK/STAT pathway, and decreased expression of STAT1 (down-regulated 1.7 fold), a transcription factor that mediates the expression of interferon stimulated genes. In all, 10 upstream regulators were predicted to be either activated or inhibited (p < 0.05, z-score >2), were confirmed by RNA sequencing, and are displayed in Table S7 in File S1. Upstream regulators predicted and validated to be activated include those involved in hematopoiesis (EPO, GATA1, RUNX1), terminal differentiation of neutrophils and eosinophils (CEBPE), and neutrophil differentiation (CSF3). Predicted and validated upstream factors that were inhibited include a specific marker of T cells and NK cells (CD2) and regulator of interferon related genes (STAT1).

#### RT-qPCR Confirmation

Relative expression of five circulating transcripts detected to be significantly differentially expressed at 7 days by RNA sequencing, alpha hemoglobin stabilizing protein (AHSP), ATP-binding cassette sub-family G member 2 (ABCG2), carbonic anhydrase 1 (CA1), BCL2/adenovirus E1B 19kDa interacting protein 3-like (BNIP3L), and thioredoxin interacting protein (TXNIP), was confirmed using RT-qPCR (Table S2 in File S1). AHSP is involved in hemoglobin synthesis and stabilization[25], ABCG2 has been identified as a marker of stem cell proliferation and may have cytoprotective effects[26,27], CA1 catalyzes the hydration of CO2 in erythrocytes for transport in the blood [28], BNIP3L is labeled as the cardiomyocyte suicide gene and is known to regulate apoptosis, necrosis and possibly autophagy [29-33], and TXNIP which is an important metabolic regulatory control protein[34].

RT-qPCR expression data correlated well with RNAseq data. Expression of AHSP, ABCG2, CA1, BNIP3L was significantly higher at 7 days compared to time of implant. TXNIP expression was highest in the non-heart failure patients, dropped in expression at 7 days post LVAD and partially recovered by 6 months. 

### 6 months After LVAD Implantation

#### Canonical Signaling Pathways

All statistically significant pathways identified at the 6-month time point following implant can be found in Table S6 in File S1. Many of the canonical signaling pathways differentially influenced at 7 days remained statistically significant at 6 months (Table 2). Transcripts related to Antigen Presentation were uniformly up-regulated by 6 months. Those involved in the T cell co-stimulatory receptor (OX40) canonical signaling were nearly all up-regulated, as were transcripts related to Allograft Rejection Signaling, and Cytotoxic T Lymphocyte-mediated Apoptosis of Target Cells (Table 2). Most transcripts related to NF-κB Signaling, including TNF, TNFAIP3, TLR4, TLR10, and BCL10, were down-regulated at 6 months. Two transcripts related glutathione redox reactions, glutathione reductase (GSR) and glutaredoxin (GLRX) were down-regulated at 6 months, differing from the 7 day time point when transcripts related to glutathione redox reactions were uniformly up-regulated. 

#### Upstream Regulators Analysis

Transcription factors predicted to be activated or inhibited by Ingenuity 6 months after LVAD implantation are presented in Table S7 in File S1. Factors predicted and validated to be activated were IL4, STAT4, PAX5, and CD38 (Table S7 in File S1).

#### RT-qPCR Confirmation

RT-qPCR confirmed the changes in expression of RAP1B, KLF2, NOSIP, and TLR4 from implant to 6 months post implant (Table S2 in File S1). RAP1B, a platelet GTPase, was significantly reduced 180 months days following LVAD placement, while expression pre-LVAD and 7 days post was not significantly different from non-heart failure controls. KLF2, a regulator of endothelial thrombotic function[35], increased at 6 months as detected by both methods but did not reach statistical significance with RT-qPCR. NOSIP, a negative regulator of NO synthesis[36], increased at 6 months increased as detected by RT-qPCR, though did not reach statistical significance. TLR4, involved in activation of the innate immune system, was down-regulated 1.64 fold at 6 months by RNAseq, although no change was detected by RT-qPCR. TLR4 expression in patients was about half of that detected in non-heart failure controls at all 3 time points.

## Discussion

We utilized high throughput RNA sequencing to identify changes in the peripheral blood transcriptome of heart failure patients 7 days and 6 months after implantation of a LVAD. A novel finding of this study was the improvement in canonical pathways regulating cellular immune response, antigen presentation, and T cell activation 6 months following placement of the LVAD. Compared to blood from non-failing control patients, a representative look at expression in 4 of these T cell related genes showed they were on average 2-4 fold lower for patients in heart failure compared to sex and age matched controls. This extends previous work from Liew and colleagues recently showing that T cell receptor gene expression in circulating blood predicts outcomes for patients with heart failure[37]. Work by Maisel and colleagues in the mid 1990’s showed that patients in heart failure exhibited altered immune cell function partly due to the consistent up-regulation of catecholamines[38]. In this study, we present novel data to show that 6 months after placement of a LVAD, T cell related genes increased from preoperative levels. A small study in 1994, patients with dilated cardiomyopathy treated with metoprolol had enhanced cell-mediated immunity and improvement in T-cell function, showed similar findings[39]. Ankersmit et al. published data showing that CD4+ T cells were decreased 1 month post LVAD and that this was accompanied by a significant increase in T cell apoptosis[40]. 

### Immunity

Multiple studies suggest there may be an impaired cellular immune response immediately following LVAD implantation, and that immune dysfunction may persist beyond the immediate postoperative period[41-43]. Kimball et al. found decreased cellular immune proliferative response and Th1/Th2 cytokine elaboration in LVAD patients 6 months after LVAD placement[42]. A microarray study of the peripheral blood mononuclear cell transcriptome by Sinha et al. also found down-regulation of genes related to T-cell mediated immunity at 1 and 7 days following LVAD placement, suggesting T cell suppression[15]. We observed decreased expression in a subset of genes related to cellular immune function 7 days after LVAD. These genes included STAT1, JAK1, RELA, STAT5A, STAT5B, and STAT4. At 6 months following LVAD implant, these genes were no longer down-regulated and a number of the circulating HLA genes that were down-regulated at 7 days were up-regulated; specifically, HLA-DPB1, HLA-DRA, HLA-DRB1, HLA-DRB3, as well as PDIA3, CD4, CD74, and PRF1. RT-qPCR studies demonstrated that expression of four genes related to T-cell function and survival were substantially lower in heart failure patients as compared to subjects with normal cardiac function (Figure 1). Notably, this trend also occurred in patients undergoing non-LVAD cardiac surgery. Expression of the same four genes related to cellular immunity was lower than in individuals without heart failure and trended down 7 days after non-LVAD cardiac surgery (Figure 2). These findings did not reach statistical significance, likely due to small sample size, but do suggest that heart disease and cardiac surgery themselves impact expression of these transcripts. A recent review of the literature suggests that surgical or traumatic injury suppresses cell-mediated immunity[44].

A recent paper by Tang et al. shows a decrease in CD4+ Treg cells in patients with chronic heart failure compared to controls[45]. Work by Vanburen et al. show that expression of 10 genes that regulate T cells is able to predict outcome of patients in heart failure[37]. We identified three of these genes that were significantly altered in our population, CD3E, CD3D, and TXK. Transcriptome-based evaluation of immune status could also be useful in predicting patient-specific susceptibility to infections.

### Blood vs. Heart

An important and unanswered question has been the identification of transcripts that are significantly altered in the heart as well as the blood following LVAD implant. ABCG2, identified as a marker of cardiac side population cells, was increased in the myocardium following mechanical unloading of the ventricle in a previous report from Wohlschlaeger and colleagues[26]. In this study, circulating ABCG2 increased ~ 8 fold at 7 days. Previous reports showed that TNF-alpha protein decreases in the heart following LVAD placement[46] and in plasma at one week following LVAD[47]. In this study we identified a decrease in TNF-alpha in plasma at 180 days. BCL2L1, an anti-apoptotic factor that increases in myocardium with LVAD support for 36 to 169 days[48], also increased 4-fold in blood at 7 days but returned to pre-LVAD levels by 180 days. 

### Genes related to Red Blood Cells/erythropoiesis

It is interesting to note that many of the top up-regulated genes 7 days after LVAD implant code for proteins found on or within red blood cells. The upstream regulators analysis also suggests that several genes involved in hematopoiesis are up-regulated and predicts activation of erythropoietin based on observed changes in the dataset (Table S7 in File S1). Carbonic anhydrase 1 (CA1) is one of the most abundant proteins in red blood cells and catalyzes the reversible hydration of carbon dioxide, regulating pH, ion transport, and water and electrolyte balance[28]. In intact human erythrocytes, CA1 contributes to approximately 50% of the total CA activity[28]. This gene was up-regulated 17 fold one-week post LVAD implant and was confirmed with RT-qPCR (Table S2 in File S1). CA1 was also up-regulated 14 fold after non-LVAD cardiac surgery (Figure 2), suggesting the underlying mechanism responsible is not specific to LVAD implant. 

Alpha-hemoglobin stabilizing protein (AHSP) expression increased 6-fold 7 days after implant of the LVAD, a finding that was confirmed with RT-qPCR (Table S2 in File S1). A similar increase in expression was observed in non-LVAD cardiac surgery (Figure 2). AHSP binds free alpha-hemoglobin, preventing harmful aggregation and precipitation of alpha-hemoglobin and potentially modulates states of elevated free hemoglobin[49]. AHSP has also been shown to be necessary for hemoglobin assembly during erythropoiesis[50]. At 180 days after surgery AHSP expression remained increased 1.8 fold, however this was not statistically significant.

Other genes related to red blood cell structure or function which were up-regulated between 6 and 23-fold include HBD (hemoglobin delta, a structural component of hemoglobin), TSPO2 (translocator protein 2, involved in cholesterol redistribution during erythropoiesis), ALAS2 (aminolevulinate, delta-, synthase 2, critical in heme biosynthesis), GYPA and GYPB (glycophorins A and B, major sialoglycoproteins of the human erythrocyte membranes), RHCE (Rh blood group, CcEe antigens, determinants of Rh blood group), RHAG (Rh-associated glycoprotein, an erythrocyte specific membrane channel) and SLC14A1 (solute carrier family 14, member 1, involved in urea transport) (Table S3 in File S1). It is suspected that increased expression in genes related to RBCs and erythropoiesis may be related to acute surgical blood loss and the subsequent response to replace RBCs. There was no evidence of clinical hemolysis in the patients studied as assessed by measuring plasma free hemoglobin, haptoglobin, and hemopexin (data not shown).

### Limitations

There are several limitations to this study. First, our sample size was limited to 9 and 7 patients at the 7-day and 6 month points, respectively, and the cohort is predominantly male. Second, heart failure patients are a medically complex, heterogeneous group. However, the study design of our experiment (a paired test which measures the same sample at multiple time points) minimizes the effects of confounding factors. Third, blood is a complex, variable system composed of multiple cell types. Expression profiles from blood are likely influenced by the relative amounts and degree of maturation of each cell type present. Fourth, while we are primarily interested in differential gene expression related to LVAD insertion, the proximity to surgery at the 1-week interval is a major confounding factor that impedes our ability to detect changes specific to the relatively non-pulsatile blood flow from contemporary LVADs. The similar trends in expression after non-LVAD cardiac surgery (Figure 2) imply that observations at the 7-day time point may be related to cardiac surgery in general rather than a process specific to the LVAD or continuous blood flow. Finally, the medication regimen between patients was not uniform and could have a significant impact on expression patterns. 

Heart failure is a major cause of morbidity and mortality in the United States, affecting 5.7 million Americans with an estimated cost of close to 40 billion dollars annually[51]. As the population ages the number of patients with end-stage heart failure is expected to increase. These patients benefit from continuous-flow left ventricular assist devices (LVADs), which have become an invaluable tool as a bridge to transplant or destination therapy. Although outcomes following CF-VAD have improved substantially, significant complications remain, particularly with regard to the delicate balance between thrombosis and hemorrhage. Substantial progress has been made in understanding molecular and genetic changes following VAD[17], but much remains unknown. The impact of altered blood flow from CF-VAD on endothelial function is unclear[52], and improved understanding is likely to accelerate efforts to mitigate hematologic complications. Finally, studying patients whose heart failure is briskly and substantially reversed with mechanical circulatory support may offer insights into the pathophysiology of heart failure beyond the findings in this specific population. Here we have identified several candidates for further investigation as we strive to improve our understanding of heart failure and treatments such as mechanical circulatory support.

## Supporting Information

File S1 **Tables S1-S8.**
(DOCX)
