# Supplementary material for: Identification of Differentially Expressed Transcripts and Pathways in Blood One Week and Six Months Following Implant of Left Ventricular Assist Devices
Source: PLoS One. 2013 Oct 21;8(10):e77951. doi: 10.1371/journal.pone.0077951 (PMC3804545; doi:10.1371/journal.pone.0077951)
Supplement: File S1 — Tables S1-S8. (DOCX) [file pone.0077951.s001.docx]

**Table S1**. Characteristics of four patients who underwent non-LVAD cardiac surgery (A) and six healthy individuals without heart failure (B). Blood samples were obtained from age-matched subjects without heart failure and patients undergoing non-LVAD cardiac surgery.

| **A. Individuals undergoing non-LVAD cardiac surgery** | | | | |
| --- | --- | --- | --- | --- |
| **Patient ID** | **Gender** | **Age** | **Ethnicity** | **Surgery** |
| A | Male | 56 | Caucasian | CABG |
| B | Male | 39 | African-American | CABG |
| C | Male | 75 | Caucasian | CABG + valve repair |
| D | Female | 59 | Hispanic | CABG |
| **B. Individuals without heart failure** | | | |  |
| 1 | Male | 51 | Caucasian | - |
| 2 | Male | 72 | Caucasian | - |
| 3 | Male | 44 | African-American | - |
| 7 | Male | 44 | Caucasian | - |
| 8 | Female | 54 | Caucasian | - |
| 9 | Female | 58 | Caucasian | - |

CABG, coronary artery bypass graft

**Table S2**. Relative expression detected by RT-qPCR and RNA sequencing normalized to pre-LVAD expression = 1. Mean 2^-ddCt or FPKM across all samples at each time point were divided by the mean 2^-ddCt or FPKM for pre-LVAD samples. A paired T-test on log2-transformed data was used to test for statistical significance.

* p < 0.05 vs. Pre-LVAD

^Ŧ^ p < 0.05 vs. non-heart failure

| Transcript | Non-heart failure | Pre-LVAD | 7 days post LVAD | | 180 days post LVAD | |
| --- | --- | --- | --- | --- | --- | --- |
|  |  |  | RT-qPCR | RNA-seq | RT-qPCR | RNA-seq |
| CA1 | 0.42 | 1.00 | 15.76*^Ŧ^ | 17.48* | 1.67 | 1.09 |
| ABCG2 | 1.02 | 1.00 | 6.55*^Ŧ^ | 8.53* | 1.03 | 1.20 |
| AHSP | 0.85 | 1.00 | 8.86*^Ŧ^ | 9.82* | 2.15 | 1.34 |
| BNIP3L | 0.65 | 1.00 | 3.49*^Ŧ^ | 4.29* | 1.18 | 1.02 |
| TXNIP | 2.24 | 1.00 ^Ŧ^ | 0.76^Ŧ^ | 0.67* | 1.42 | 1.11 |
| GAB1 | 2.33 | 1.00 ^Ŧ^ | 1.44^Ŧ^ | 1.10 | 1.33 ^Ŧ^ | 0.76* |
| RAP1B | 1.10 | 1.00 | 1.12 | 0.98 | 0.77* ^Ŧ^ | 0.60* |
| NOSIP | 2.90 | 1.00 ^Ŧ^ | 1.09 ^Ŧ^ | 0.94 | 1.64 | 1.58* |
| SCAMP2 | 3.93 | 1.00 ^Ŧ^ | 0.92 ^Ŧ^ | 0.86* | 1.79*^Ŧ^ | 1.08* |
| IL16 | 2.41 | 1.00 ^Ŧ^ | 0.97 ^Ŧ^ | 0.80* | 1.60*^Ŧ^ | 1.02 |
| MUTYH | 3.08 | 1.00 ^Ŧ^ | 0.79*^Ŧ^ | 0.90 | 1.73*^Ŧ^ | 1.34 |
| TLR4 | 1.94 | 1.00 ^Ŧ^ | 0.98 ^Ŧ^ | 0.91 | 1.07 ^Ŧ^ | 0.61* |
| BAG6 | 1.59 | 1.00 | 1.83* | 1.83* | 1.18 | 1.71* |
| KLF2 | 4.16 | 1.00 ^Ŧ^ | 0.87 ^Ŧ^ | 0.97 | 1.63 ^Ŧ^ | 1.35* |
| SP1 | 2.50 | 1.00 ^Ŧ^ | 1.14 ^Ŧ^ | 1.01 | 1.17 ^Ŧ^ | 0.99 |
| TMSB4X | 2.04 | 1.00 ^Ŧ^ | 1.35 ^Ŧ^ | 1.13 | 1.48 ^Ŧ^ | 0.80* |
| CD4 | 2.58 | 1.00^Ŧ^ | 0.64*^Ŧ^ | 0.67* | 1.52* | 1.35* |
| HLA-DRA | 1.83 | 1.00^Ŧ^ | 0.69*^Ŧ^ | 0.69* | 1.35*^Ŧ^ | 1.30* |
| PDIA3 | 2.13 | 1.00^Ŧ^ | 0.81* ^Ŧ^ | 0.74* | 1.19^Ŧ^ | 1.35* |
| PRF1 | 4.29 | 1.00^Ŧ^ | 0.60*^Ŧ^ | 0.60* | 2.44*^Ŧ^ | 2.52* |

Table S3. Differentially expressed transcripts (FDR < 0.05) before LVAD implant and 7 days after with fold change greater than 1.5. Transcripts with a median FPKM <1 at both time points are excluded.

| **Gene Symbol** | **Locus** | **p** | **q** | **mean FPKM pre-LVAD** | **mean FPKM 7 days post LVAD** | **Fold Change** |
| --- | --- | --- | --- | --- | --- | --- |
| AC016757.1,FAM132B | 2:239067622-239077541 | 0.0024281 | 0.035 | 1.31 | 18.27 | 52.77 |
| RAP1GAP | 1:21922707-21995856 | 0.0008745 | 0.022 | 8.32 | 86.52 | 39.33 |
| DEFA8P | 8:6808247-6809121 | 0.0006238 | 0.02 | 3.79 | 34.91 | 29.75 |
| IGLV7-46 | 22:22723981-22724454 | 0.0018053 | 0.03 | 8.34 | 71.78 | 25.75 |
| HBBP1 | 11:5263183-5264767 | 0.0024593 | 0.035 | 0.64 | 6.05 | 23.44 |
| RP11-63D14.1.1 | 11:38520544-38521736 | 0.004072 | 0.044 | 1.31 | 12.91 | 22.02 |
| RP11-382A20.4.1 | 15:83776158-84108197 | 0.0024443 | 0.035 | 0.44 | 3.62 | 18.97 |
| CA1 | 8:86239836-86393693 | 0.0000587 | 0.012 | 172.08 | 1395.62 | 17.44 |
| HBD | 11:5253907-5256600 | 0.0001832 | 0.014 | 62.31 | 588.99 | 17.29 |
| FAM83A | 8:124191199-124222314 | 0.0001826 | 0.014 | 0.26 | 6.54 | 16.87 |
| ISCA1P1 | 5:62072703-62073090 | 0.0003602 | 0.017 | 5.67 | 35.94 | 16.44 |
| MT1L | 16:56651387-56652730 | 0.0011327 | 0.024 | 4.87 | 49.78 | 13.77 |
| PRTN3 | 19:840984-848180 | 0.0030386 | 0.038 | 6.28 | 32.64 | 13.24 |
| OLFM4 | 13:53602829-53626196 | 0.0008962 | 0.022 | 5.72 | 32.33 | 12.10 |
| HES6 | 2:239140324-239198743 | 0.0007716 | 0.021 | 2.35 | 22.93 | 10.77 |
| DEFA4 | 8:6793343-6795860 | 0.0003553 | 0.017 | 70.59 | 295.18 | 10.70 |
| DEFA1B,DEFA3 | 8:6854287-6875823 | 0.0001678 | 0.014 | 2555.72 | 12985.10 | 10.30 |
| TSPO2 | 6:40994771-41067715 | 0.0004661 | 0.018 | 4.30 | 26.18 | 9.96 |
| ELANE | 19:852290-856242 | 0.0008615 | 0.022 | 17.39 | 82.43 | 9.85 |
| AHSP | 16:31539184-31540124 | 0.0000887 | 0.012 | 489.33 | 3432.33 | 9.81 |
| MMP8 | 11:102582525-102597781 | 0.000096 | 0.012 | 10.03 | 59.57 | 9.66 |
| DEFA1 | 8:6835170-6837602 | 0.0001962 | 0.014 | 1503.34 | 7694.22 | 9.54 |
| CTA-392E5.1.1 | 8:86851933-86963312 | 0.0024105 | 0.035 | 0.45 | 2.23 | 9.22 |
| ALAS2 | X:55026789-55057497 | 0.0005056 | 0.019 | 1578.53 | 7163.38 | 9.03 |
| AL359758.1.1 | 1:145139024-145139569 | 0.0009964 | 0.024 | 1.26 | 6.95 | 9.00 |
| BPGM | 7:134331559-134364565 | 0.0002019 | 0.014 | 78.07 | 418.77 | 8.82 |
| GYPA,GYPB | 4:144833483-145061904 | 0.0002674 | 0.016 | 67.29 | 332.03 | 8.75 |
| RNF182 | 6:13924676-13980533 | 0.0001189 | 0.013 | 1.60 | 6.44 | 8.45 |
| LTF | 3:46477135-46526724 | 0.0007113 | 0.02 | 44.64 | 182.04 | 8.33 |
| IGKV2-28 | 2:89521178-89521942 | 0.0030682 | 0.039 | 15.00 | 113.08 | 8.33 |
| ARG2 | 14:68086514-68162531 | 0.0025322 | 0.036 | 1.49 | 8.62 | 8.31 |
| AC233264.2,IGKV2D-28 | 2:89998788-89999564 | 0.0049174 | 0.048 | 14.29 | 104.11 | 8.30 |
| RP11-1D12.2.1 | 8:65713792-65730130 | 0.0014974 | 0.028 | 0.84 | 6.35 | 8.06 |
| RHCE | 1:25568727-25756683 | 0.000308 | 0.017 | 3.85 | 21.00 | 8.02 |
| LCN2 | 9:130911349-130915734 | 0.0002531 | 0.016 | 110.56 | 560.16 | 7.93 |
| CTSG | 14:25042727-25045466 | 0.0003511 | 0.017 | 9.70 | 34.91 | 7.87 |
| CEACAM8 | 19:43084392-43099082 | 0.0003209 | 0.017 | 8.49 | 37.60 | 7.82 |
| IGLC1,IGLJ1,IGLL5 | 22:23229959-23238014 | 0.000131 | 0.013 | 102.77 | 446.11 | 7.76 |
| AZU1 | 19:825096-832017 | 0.0001279 | 0.013 | 12.99 | 73.78 | 7.61 |
| SLC14A1 | 18:43304091-43332485 | 0.0001474 | 0.013 | 16.68 | 79.28 | 7.58 |
| CD177 | 19:43857824-43867480 | 0.0027431 | 0.037 | 17.20 | 33.55 | 7.40 |
| CAMP | 3:48264836-48266981 | 0.0000688 | 0.012 | 131.82 | 794.14 | 6.94 |
| TSTA3 | 8:144694787-144700218 | 0.00144 | 0.027 | 94.73 | 427.21 | 6.93 |
| TMEM56 | 1:95582893-95699538 | 0.0006986 | 0.02 | 2.14 | 10.04 | 6.76 |
| RP11-293A21.1.1 | 4:26859299-27027003 | 0.0002916 | 0.016 | 4.44 | 20.45 | 6.74 |
| RHAG | 6:49572870-49604552 | 0.0004239 | 0.018 | 1.18 | 5.47 | 6.73 |
| SMOX | 20:4101626-4168394 | 0.0015403 | 0.028 | 12.90 | 78.16 | 6.73 |
| HEPACAM2 | 7:92817898-92855837 | 0.000341 | 0.017 | 0.66 | 3.75 | 6.72 |
| RAB6B | 3:133502876-133614680 | 0.0050088 | 0.048 | 2.03 | 8.21 | 6.63 |
| CTNNAL1 | 9:111696460-111775809 | 0.0003805 | 0.018 | 6.05 | 27.61 | 6.53 |
| SELENBP1 | 1:151336777-151345209 | 0.0000726 | 0.012 | 161.52 | 785.79 | 6.50 |
| COL17A1,MIR936 | 10:105791043-105845760 | 0.0009095 | 0.023 | 0.86 | 4.64 | 6.44 |
| CEACAM6 | 19:42259328-42276113 | 0.0002684 | 0.016 | 3.37 | 13.34 | 6.38 |
| ABCC13 | 21:15608526-15735075 | 0.0000567 | 0.012 | 9.66 | 41.89 | 6.26 |
| PLEK2 | 14:67853699-67878917 | 0.0010212 | 0.024 | 10.99 | 52.55 | 6.23 |
| CTC-756D1.3.1 | 8:23350521-23351711 | 0.0005497 | 0.019 | 1.33 | 6.62 | 6.20 |
| ANKRD9 | 14:102973178-102976136 | 0.0006294 | 0.02 | 6.86 | 30.79 | 6.19 |
| SLC6A9 | 1:44457030-44497139 | 0.0002688 | 0.016 | 1.76 | 8.01 | 6.18 |
| ABCG2 | 4:89011415-89152474 | 0.0000259 | 0.012 | 0.57 | 3.27 | 6.16 |
| KRT1 | 12:53068519-53074191 | 0.0008948 | 0.022 | 22.03 | 116.09 | 6.03 |
| RP1-286D6.2.1 | 1:3689351-3692546 | 0.0005551 | 0.019 | 22.42 | 92.58 | 6.00 |
| FAM20A | 17:66531257-66597095 | 0.0001016 | 0.013 | 1.25 | 5.92 | 5.94 |
| HEMGN | 9:100689072-100707138 | 0.0001255 | 0.013 | 9.28 | 41.98 | 5.93 |
| NUDT4 | 12:93771658-93797024 | 0.0000766 | 0.012 | 36.03 | 163.55 | 5.88 |
| OSBP2 | 22:30279143-31530682 | 0.0006878 | 0.02 | 19.67 | 95.39 | 5.86 |
| FECH | 18:55215514-55254004 | 0.0001676 | 0.014 | 22.92 | 97.74 | 5.86 |
| XK | X:37208527-38548169 | 0.0004156 | 0.018 | 4.89 | 18.45 | 5.86 |
| SNCA | 4:90472506-90763129 | 0.0002065 | 0.014 | 296.97 | 1259.65 | 5.84 |
| FAM46C | 1:118148555-118170994 | 0.0005519 | 0.019 | 22.37 | 89.59 | 5.81 |
| CDC42EP1 | 22:37956453-37965412 | 0.0005146 | 0.019 | 1.71 | 9.31 | 5.81 |
| ACHE | 7:100487614-100494594 | 0.0001867 | 0.014 | 1.04 | 5.31 | 5.77 |
| EMID1 | 22:29601839-29655586 | 0.0013027 | 0.026 | 1.76 | 6.82 | 5.76 |
| CD24P4 | Y:21034386-21239302 | 0.0011028 | 0.024 | 37.09 | 217.20 | 5.74 |
| C17orf109 | 17:73622924-73663269 | 0.0000925 | 0.012 | 8.41 | 41.39 | 5.74 |
| CMBL | 5:10275986-10308138 | 0.0000707 | 0.012 | 1.56 | 8.57 | 5.74 |
| IGJ | 4:71494460-71552533 | 0.0014084 | 0.027 | 112.42 | 460.40 | 5.65 |
| OLR1 | 12:10310901-10344400 | 0.0000916 | 0.012 | 1.89 | 11.76 | 5.65 |
| HIST1H2AI | 6:27775898-27776429 | 0.0007283 | 0.02 | 0.71 | 2.96 | 5.64 |
| AC104389.16.1 | 11:5226270-5228538 | 0.0012075 | 0.025 | 0.09 | 0.98 | 5.59 |
| ISCA1 | 9:88879460-88897676 | 0.0000487 | 0.012 | 31.70 | 146.20 | 5.59 |
| IFIT1B | 10:90973325-91180758 | 0.000256 | 0.016 | 16.50 | 76.47 | 5.49 |
| CCL23 | 17:34340096-34345005 | 0.0025993 | 0.036 | 3.34 | 14.31 | 5.48 |
| GLRX5 | 14:95998633-96011061 | 0.0001076 | 0.013 | 130.94 | 558.10 | 5.48 |
| AC093484.6.1,UBB | 17:16284112-16287220 | 0.0000654 | 0.012 | 3217.79 | 14103.82 | 5.48 |
| SLC2A5 | 1:9095165-9148537 | 0.0000855 | 0.012 | 1.65 | 8.46 | 5.45 |
| PRRG1,RP11-357K9.2.1,RP5-972B16.2.1,TSPAN7 | X:37208527-38548169 | 0.0043296 | 0.045 | 1.10 | 4.77 | 5.43 |
| TMCC2 | 1:205197303-205242471 | 0.0005624 | 0.019 | 14.08 | 72.78 | 5.43 |
| RIOK3 | 18:21032786-21063104 | 0.0000529 | 0.012 | 37.07 | 169.70 | 5.38 |
| KLF1 | 19:12995235-12998017 | 0.0001369 | 0.013 | 21.69 | 106.24 | 5.38 |
| METTL7B | 12:56075329-56118489 | 0.0000938 | 0.012 | 0.59 | 3.02 | 5.35 |
| CTD-2154I11.1.1,RFESD | 5:94982457-95034415 | 0.0000366 | 0.012 | 1.71 | 8.74 | 5.28 |
| GMPR | 6:16238810-16295780 | 0.000137 | 0.013 | 84.55 | 368.88 | 5.28 |
| TMOD1 | 9:100263461-100436030 | 0.0003879 | 0.018 | 14.25 | 52.97 | 5.27 |
| CRISP3 | 6:49695096-49712150 | 0.0001969 | 0.014 | 8.66 | 42.13 | 5.27 |
| RETN | 19:7733971-7735340 | 0.0001843 | 0.014 | 47.34 | 226.18 | 5.25 |
| HBQ1 | 16:230451-231180 | 0.0001484 | 0.013 | 136.05 | 613.90 | 5.24 |
| CISD2 | 4:103790134-103940896 | 0.0000498 | 0.012 | 16.26 | 70.42 | 5.23 |
| RP11-266I14.3.1,TCTEX1D1 | 1:67218141-67244470 | 0.0015364 | 0.028 | 0.33 | 1.55 | 5.17 |
| MARCH3 | 5:126203405-126366500 | 0.0007519 | 0.021 | 3.16 | 13.08 | 5.10 |
| PRDX2 | 19:12907633-12912694 | 0.0001638 | 0.014 | 142.07 | 578.89 | 5.09 |
| FHL2 | 2:105974168-106054960 | 0.0002501 | 0.016 | 2.42 | 10.10 | 5.09 |
| HBM,HBZ,HBZP1 | 16:202685-216767 | 0.0009771 | 0.023 | 829.50 | 2889.88 | 5.05 |
| RP11-525A16.1.1 | 10:112188597-112255954 | 0.0047376 | 0.047 | 0.31 | 1.51 | 5.01 |
| IGKV2-24 | 2:89475811-89476644 | 0.0043832 | 0.045 | 6.65 | 18.98 | 4.99 |
| AC099344.4.1 | 2:11534106-11543203 | 0.000933 | 0.023 | 35.68 | 125.58 | 4.84 |
| MAOA | X:43515466-43606068 | 0.0007581 | 0.021 | 0.67 | 2.78 | 4.83 |
| MYL4 | 17:45286427-45301045 | 0.0024673 | 0.035 | 132.98 | 407.35 | 4.80 |
| RNASE3 | 14:21359557-21360507 | 0.0017612 | 0.03 | 25.22 | 109.17 | 4.78 |
| CCL27,RP11-195F19.30.1,RP11-195F19.5.1 | 9:34638129-34681295 | 0.0005208 | 0.019 | 0.35 | 1.86 | 4.76 |
| D87015.1,IGLV2-14 | 22:23101184-23101707 | 0.0029765 | 0.038 | 73.33 | 229.19 | 4.75 |
| PAGE2,PAGE2B | X:55101495-55119275 | 0.0006728 | 0.02 | 15.83 | 50.31 | 4.73 |
| DYRK3 | 1:206807993-206857764 | 0.0004385 | 0.018 | 0.80 | 3.55 | 4.73 |
| DPCD | 10:103330316-103369425 | 0.0005598 | 0.019 | 9.56 | 38.62 | 4.70 |
| RP11-122A3.2.1 | 8:91634222-91997485 | 0.0004513 | 0.018 | 1.03 | 4.20 | 4.65 |
| EPB42 | 15:43398422-43513481 | 0.0000697 | 0.012 | 57.32 | 223.66 | 4.65 |
| KANK2 | 19:11274943-11308243 | 0.0014242 | 0.027 | 0.99 | 3.39 | 4.59 |
| CRISP2 | 6:49660072-49681299 | 3.09E-06 | 0.004 | 0.54 | 3.30 | 4.59 |
| CA2 | 8:86239836-86393693 | 0.0000652 | 0.012 | 11.92 | 50.73 | 4.57 |
| AC003102.1,SLC4A1 | 17:42325752-42345509 | 0.0002901 | 0.016 | 152.83 | 492.53 | 4.57 |
| CADM3,DARC | 1:159141398-159176290 | 0.0016876 | 0.029 | 5.72 | 16.64 | 4.57 |
| RP11-317J10.2.1 | 8:86239836-86393693 | 0.0003777 | 0.018 | 6.64 | 22.81 | 4.54 |
| ABCA13 | 7:48211054-48687092 | 0.0000147 | 0.009 | 0.55 | 3.93 | 4.52 |
| BPI | 20:36838889-36965907 | 0.0003753 | 0.018 | 17.66 | 59.80 | 4.51 |
| BEX1 | X:102317578-102319168 | 0.0031062 | 0.039 | 1.25 | 5.65 | 4.51 |
| CHPT1 | 12:102090724-102137918 | 0.0002417 | 0.015 | 105.33 | 380.08 | 4.51 |
| PRRT4 | 7:127990378-128001739 | 0.0027193 | 0.037 | 0.63 | 2.64 | 4.49 |
| SIAH2 | 3:150458913-150481264 | 0.0001473 | 0.013 | 73.07 | 279.01 | 4.42 |
| THEM5 | 1:151814352-151826173 | 0.0005141 | 0.019 | 1.40 | 5.56 | 4.42 |
| MXI1 | 10:111967362-112047123 | 0.0005931 | 0.02 | 67.93 | 208.38 | 4.41 |
| BNIP3L | 8:26236774-26515694 | 0.0001274 | 0.013 | 222.95 | 803.42 | 4.29 |
| RP11-77H9.8.1 | 16:8985950-9060847 | 0.0017842 | 0.03 | 10.26 | 32.09 | 4.28 |
| C2orf88 | 2:190744334-191236391 | 0.0033541 | 0.04 | 11.73 | 40.23 | 4.27 |
| OR2W3,TRIM58 | 1:248020500-248060449 | 0.0005611 | 0.019 | 63.85 | 192.01 | 4.26 |
| BCL2L1 | 20:30252254-30311792 | 0.0003316 | 0.017 | 251.57 | 848.61 | 4.24 |
| KCNH2 | 7:150642048-150675403 | 0.0007056 | 0.02 | 2.75 | 8.17 | 4.24 |
| IGF2BP2 | 3:185361526-185542844 | 0.0012154 | 0.025 | 12.11 | 34.77 | 4.16 |
| MOSPD1 | X:134021655-134049297 | 0.0001057 | 0.013 | 4.60 | 17.89 | 4.15 |
| IGHA1 | 14:106173456-106175002 | 0.0011837 | 0.025 | 95.98 | 304.41 | 4.13 |
| SGIP1 | 1:66999065-67213982 | 0.0022094 | 0.033 | 3.08 | 7.61 | 4.13 |
| PCSK1N | X:48689503-48694035 | 0.0050754 | 0.049 | 5.73 | 18.65 | 4.12 |
| IGHA2 | 14:106053225-106054732 | 0.0021473 | 0.033 | 45.77 | 160.33 | 4.05 |
| CSDA | 12:10851682-10875911 | 0.0036224 | 0.041 | 755.00 | 1958.65 | 4.03 |
| CHIT1 | 1:203181954-203242769 | 0.0003015 | 0.017 | 5.59 | 28.77 | 4.01 |
| HTRA3 | 4:8271491-8308838 | 0.0017387 | 0.03 | 0.67 | 2.96 | 3.98 |
| SEPP1 | 5:42756902-42887494 | 0.0052982 | 0.05 | 0.95 | 3.27 | 3.98 |
| SLC2A1 | 1:43391518-43424539 | 0.0014379 | 0.027 | 20.91 | 79.96 | 3.97 |
| YOD1 | 1:207217193-207254369 | 0.0002247 | 0.015 | 6.40 | 22.74 | 3.97 |
| BSG | 19:571309-583492 | 0.0005064 | 0.019 | 468.34 | 1575.31 | 3.95 |
| RHD | 1:25568727-25756683 | 0.0040637 | 0.044 | 6.82 | 20.21 | 3.95 |
| C17orf99 | 17:76142433-76162258 | 0.0032785 | 0.039 | 0.67 | 2.06 | 3.94 |
| PNP | 14:20937112-20945253 | 0.0002204 | 0.015 | 53.25 | 204.61 | 3.94 |
| GPR146 | 7:1036622-1177896 | 0.0005112 | 0.019 | 12.09 | 37.46 | 3.93 |
| KLC3 | 19:45843997-45873876 | 0.0017511 | 0.03 | 5.05 | 16.77 | 3.93 |
| RBM38 | 20:55959215-55984389 | 0.0006701 | 0.02 | 220.44 | 711.26 | 3.91 |
| E2F1 | 20:32263488-32274210 | 0.0038801 | 0.043 | 2.25 | 6.79 | 3.90 |
| SLC1A5 | 19:47278139-47291851 | 0.0004396 | 0.018 | 31.11 | 105.32 | 3.89 |
| RP11-1280I22.1.1 | 5:138723168-138725770 | 0.0010704 | 0.024 | 13.30 | 34.56 | 3.88 |
| BLVRB | 19:40953692-40971725 | 0.0005862 | 0.02 | 316.73 | 1026.56 | 3.87 |
| EIF1AY | Y:22737610-22755040 | 0.001016 | 0.024 | 61.33 | 228.06 | 3.85 |
| DUSP13 | 10:76854189-76941881 | 0.0010705 | 0.024 | 1.12 | 3.89 | 3.84 |
| TBCEL | 11:120894780-120961484 | 0.0006491 | 0.02 | 2.73 | 10.04 | 3.84 |
| RNF208 | 9:140114706-140116033 | 0.0049413 | 0.048 | 1.14 | 3.59 | 3.82 |
| TMEM86B | 19:55738001-55740632 | 0.0010926 | 0.024 | 17.37 | 56.46 | 3.82 |
| RUNDC3A | 17:42384667-42396039 | 0.0013298 | 0.026 | 47.47 | 175.77 | 3.80 |
| HMBS | 11:118955575-118964259 | 0.0004128 | 0.018 | 28.26 | 95.25 | 3.80 |
| HIST1H4D | 6:26188937-26189304 | 0.0002385 | 0.015 | 1.63 | 5.30 | 3.71 |
| RP11-203M5.8.1 | 14:20937112-20945253 | 0.0005263 | 0.019 | 9.02 | 30.55 | 3.70 |
| SOX6 | 11:15987994-16778649 | 0.0009123 | 0.023 | 0.73 | 2.26 | 3.68 |
| C13orf15 | 13:42031694-42045018 | 0.0001922 | 0.014 | 33.45 | 113.38 | 3.67 |
| AC034228.7.1,ACSL6 | 5:131142682-131348892 | 0.0006374 | 0.02 | 4.86 | 15.63 | 3.67 |
| MPO | 17:56347216-56358296 | 0.0010363 | 0.024 | 4.73 | 13.97 | 3.65 |
| GUK1 | 1:228327662-228336685 | 0.0006375 | 0.02 | 1004.91 | 2930.93 | 3.64 |
| ARL4A | 7:12726480-12730559 | 0.0003336 | 0.017 | 6.98 | 25.15 | 3.58 |
| GSPT1 | 16:11961984-12009939 | 0.0003986 | 0.018 | 128.42 | 379.48 | 3.57 |
| HAGH | 16:1845620-1934295 | 0.0004018 | 0.018 | 179.51 | 527.87 | 3.53 |
| RAB3IL1 | 11:61664772-61687741 | 0.0021696 | 0.033 | 2.19 | 6.02 | 3.53 |
| RP11-368D24__A.1.1 | X:64808260-64820630 | 0.0053714 | 0.05 | 3.33 | 8.66 | 3.52 |
| SYCP3 | 12:102090724-102137918 | 0.000603 | 0.02 | 1.62 | 4.93 | 3.52 |
| DCAF12 | 9:34084329-34127397 | 0.0010282 | 0.024 | 147.03 | 423.52 | 3.49 |
| KLHDC8A | 1:205305219-205326166 | 0.0005049 | 0.019 | 1.59 | 5.01 | 3.46 |
| HIST3H2A | 1:228645064-228645560 | 0.000607 | 0.02 | 6.78 | 19.79 | 3.45 |
| PCDH1 | 5:141232937-141258811 | 0.003237 | 0.039 | 0.44 | 1.34 | 3.45 |
| FAM210B | 20:54933970-54943719 | 0.0011857 | 0.025 | 343.34 | 882.02 | 3.44 |
| TMPRSS9 | 19:2389768-2456994 | 0.0007759 | 0.021 | 0.76 | 2.81 | 3.44 |
| LGALS3 | 14:55590827-55612126 | 0.0006614 | 0.02 | 370.16 | 1057.96 | 3.39 |
| TRAPPC5 | 19:7745760-7747748 | 0.0008721 | 0.022 | 149.11 | 448.26 | 3.37 |
| MPP1 | X:154006958-154049282 | 0.0006126 | 0.02 | 183.14 | 535.84 | 3.37 |
| PDZK1IP1 | 1:47649264-47656716 | 0.0007289 | 0.02 | 100.99 | 276.34 | 3.36 |
| RP11-24F11.2.1 | 3:46406445-46448550 | 0.0037619 | 0.042 | 4.84 | 12.45 | 3.35 |
| FGFR1OP2 | 12:27091315-27119583 | 0.0001474 | 0.013 | 40.47 | 121.03 | 3.35 |
| SESN3 | 11:94883702-94967268 | 0.0009431 | 0.023 | 52.62 | 133.16 | 3.34 |
| YIPF6 | X:67718164-67757127 | 0.0000911 | 0.012 | 7.98 | 23.44 | 3.33 |
| CLIC2 | X:154505499-154563966 | 0.0017865 | 0.03 | 2.62 | 8.65 | 3.33 |
| RP11-89C21.2.1 | 10:11502508-11653753 | 0.0001126 | 0.013 | 0.94 | 3.01 | 3.31 |
| RP11-305E17.6.1 | 1:100729564-100731676 | 0.0032338 | 0.039 | 1.79 | 5.36 | 3.31 |
| YPEL4 | 11:57405496-57429340 | 0.0030027 | 0.038 | 1.82 | 5.91 | 3.31 |
| AC051642.1,SLC25A37 | 8:23386317-23432976 | 0.0033246 | 0.039 | 1951.77 | 4705.26 | 3.29 |
| MS4A3 | 11:59824059-59838601 | 0.0023888 | 0.035 | 22.53 | 84.64 | 3.28 |
| PKIG | 20:43160425-43280874 | 0.0016239 | 0.029 | 6.78 | 22.33 | 3.27 |
| FANK1 | 10:127585107-127698161 | 0.0039093 | 0.043 | 0.96 | 2.63 | 3.26 |
| NFIX | 19:13106583-13209610 | 0.0001131 | 0.013 | 5.40 | 16.31 | 3.25 |
| STRADB | 2:202241929-202345574 | 0.0016852 | 0.029 | 328.63 | 781.15 | 3.23 |
| CDKL1 | 14:50704280-50883179 | 0.0045177 | 0.046 | 9.45 | 21.40 | 3.22 |
| ALDH5A1 | 6:24403152-24537435 | 0.00015 | 0.013 | 2.68 | 7.39 | 3.22 |
| AC017076.5.1 | 2:6968644-6980595 | 0.0005638 | 0.019 | 2.59 | 6.90 | 3.19 |
| C7orf73 | 7:135347243-135433594 | 0.000158 | 0.013 | 143.99 | 394.50 | 3.19 |
| TREML1 | 6:41117000-41122075 | 0.0024934 | 0.035 | 36.97 | 107.45 | 3.17 |
| AC009469.1,TNS1 | 2:218664511-218867718 | 0.0005568 | 0.019 | 21.91 | 61.92 | 3.16 |
| RAD23A | 19:13056653-13064448 | 0.0015126 | 0.028 | 52.34 | 145.95 | 3.15 |
| ANK1 | 8:41510738-41754280 | 0.0004492 | 0.018 | 23.82 | 68.97 | 3.12 |
| TGM2 | 20:36756862-36794980 | 0.0008939 | 0.022 | 2.71 | 7.19 | 3.12 |
| AC003043.2.1 | 17:42396992-42402238 | 0.0022759 | 0.034 | 1877.62 | 4764.73 | 3.11 |
| SNX3 | 6:108532425-108582464 | 0.0004332 | 0.018 | 203.77 | 538.22 | 3.10 |
| EPB49 | 8:21906505-21940038 | 0.001128 | 0.024 | 273.61 | 688.95 | 3.10 |
| CREG1 | 1:167498913-167523004 | 0.0002461 | 0.016 | 87.44 | 252.72 | 3.10 |
| BX936347.1 | X:153656536-153656795 | 0.0003171 | 0.017 | 3.75 | 9.78 | 3.05 |
| TTC25 | HG185_PATCH:40086886-40121903 | 0.0021618 | 0.033 | 0.50 | 1.49 | 3.02 |
| AL953854.2.1 | 9:47295854-47314322 | 0.0043469 | 0.045 | 0.56 | 1.81 | 3.02 |
| ADIPOR1 | 1:202909950-202927700 | 0.0015736 | 0.028 | 903.58 | 2112.25 | 3.01 |
| TFDP1 | 13:114239012-114295504 | 0.0006284 | 0.02 | 49.35 | 128.37 | 3.00 |
| TSTD2 | 9:100263461-100436030 | 0.0016121 | 0.028 | 12.97 | 33.08 | 3.00 |
| TMEM92 | 17:48348766-48358844 | 0.0010239 | 0.024 | 0.55 | 1.90 | 2.99 |
| SLC6A8 | X:152935184-152962048 | 0.0040991 | 0.044 | 37.58 | 81.17 | 2.99 |
| ZFY-AS1 | Y:2803111-2870667 | 0.0015965 | 0.028 | 1.18 | 3.76 | 2.98 |
| ATP8B4 | 15:50150434-50528592 | 0.0025411 | 0.036 | 3.58 | 11.57 | 2.98 |
| SLC25A39 | 17:42396992-42402238 | 0.0018691 | 0.031 | 1392.87 | 3405.48 | 2.97 |
| CYBRD1 | 2:172378756-172414643 | 0.0003206 | 0.017 | 14.98 | 40.17 | 2.97 |
| DPM2 | 9:130697377-130700763 | 0.0006054 | 0.02 | 75.78 | 202.00 | 2.96 |
| HPS1 | 10:100175954-100206709 | 0.0002895 | 0.016 | 103.58 | 276.00 | 2.96 |
| DERL3 | 22:24129149-24181315 | 0.0026822 | 0.037 | 4.14 | 10.77 | 2.94 |
| UBE2O | 17:74385613-74449288 | 0.0025288 | 0.036 | 5.08 | 12.83 | 2.94 |
| EIF2AK1 | 7:6048875-6098861 | 0.0016048 | 0.028 | 91.02 | 223.32 | 2.93 |
| FIS1 | 7:100882738-100895597 | 0.0003418 | 0.017 | 156.39 | 430.77 | 2.93 |
| GPX1 | 3:49394608-49396033 | 0.0035886 | 0.041 | 1125.63 | 2878.69 | 2.92 |
| AC096579.7.1,IGKC,IGKJ1 | 2:89109983-89165653 | 0.0043653 | 0.045 | 658.10 | 1595.93 | 2.92 |
| TRAK2 | 2:202241929-202345574 | 0.0003697 | 0.018 | 8.91 | 23.55 | 2.92 |
| LYL1 | 19:13209847-13213681 | 0.0018405 | 0.03 | 111.77 | 303.91 | 2.91 |
| PFDN4 | 20:52824385-52844591 | 0.000498 | 0.019 | 5.64 | 15.31 | 2.89 |
| DCAF10 | 9:37510888-38069210 | 0.0008643 | 0.022 | 10.75 | 29.32 | 2.89 |
| DCUN1D1 | 3:182655861-182703741 | 0.0006985 | 0.02 | 17.79 | 42.03 | 2.89 |
| TRIM10 | HSCHR6_MHC_SSTO:30109309-30118301 | 0.0000415 | 0.012 | 0.51 | 1.56 | 2.88 |
| RNF11 | 1:51701942-51739127 | 0.0000619 | 0.012 | 50.51 | 135.64 | 2.88 |
| RNASE1 | 14:21269386-21271437 | 0.0000297 | 0.012 | 1.37 | 4.40 | 2.87 |
| TPM1 | 15:63334830-63364114 | 0.00029 | 0.016 | 15.30 | 43.13 | 2.86 |
| AC100793.2.1 | 17:41026686-41050751 | 0.0000575 | 0.012 | 2.85 | 8.27 | 2.85 |
| PINK1 | 1:20959947-20988000 | 0.0006623 | 0.02 | 36.99 | 92.28 | 2.85 |
| ST6GALNAC4 | 9:130670113-130679317 | 0.0034722 | 0.04 | 75.62 | 161.16 | 2.83 |
| USP12 | 13:27640292-27746033 | 0.0002152 | 0.015 | 7.41 | 18.71 | 2.82 |
| TFR2 | 7:100218038-100240402 | 0.0001973 | 0.014 | 2.24 | 6.02 | 2.81 |
| CDC34 | 19:531732-542084 | 0.0011249 | 0.024 | 164.41 | 407.24 | 2.81 |
| C14orf45 | 14:74318546-74551196 | 0.0003736 | 0.018 | 11.75 | 27.36 | 2.81 |
| UBXN6 | 19:4445005-4457791 | 0.0029898 | 0.038 | 395.20 | 909.59 | 2.81 |
| DNAJA4 | 15:78542843-78592136 | 0.0011821 | 0.025 | 7.48 | 18.28 | 2.81 |
| RP11-244F12.2.1 | 15:63334830-63364114 | 0.0001136 | 0.013 | 2.62 | 7.16 | 2.80 |
| NEDD4L | 18:55711618-56068772 | 0.0009293 | 0.023 | 0.92 | 2.59 | 2.80 |
| GYPC | 2:127413508-127454246 | 0.0044821 | 0.046 | 1461.47 | 3313.19 | 2.80 |
| CETP | 16:56995761-57017757 | 0.0023819 | 0.035 | 1.16 | 3.10 | 2.79 |
| TCF3 | 19:1609292-1652326 | 0.0012406 | 0.025 | 12.16 | 28.51 | 2.77 |
| ABCC4 | 13:95672082-95953687 | 0.0000947 | 0.012 | 1.51 | 4.21 | 2.76 |
| RP11-884K10.5.1 | 3:53918436-53926015 | 0.0002841 | 0.016 | 62.71 | 159.57 | 2.75 |
| PIGC | 1:171810620-172437971 | 0.0048746 | 0.048 | 37.92 | 118.47 | 2.74 |
| TUBB2A | 6:3153902-3157809 | 0.000772 | 0.021 | 39.44 | 69.66 | 2.74 |
| C5orf4 | 5:154198050-154256353 | 0.0028022 | 0.037 | 16.70 | 42.21 | 2.73 |
| CEBPE | 14:23586512-23588825 | 0.0004539 | 0.018 | 7.94 | 22.54 | 2.73 |
| CDKN2C | 1:51426416-51440305 | 0.0010996 | 0.024 | 4.28 | 10.89 | 2.72 |
| GABARAPL2 | 16:75562429-75657198 | 0.0005391 | 0.019 | 257.83 | 652.05 | 2.72 |
| H2AFJ | 12:14927269-14930936 | 0.0042814 | 0.045 | 27.29 | 66.79 | 2.72 |
| FKBP1B,MFSD2B | 2:24232950-24286551 | 0.0007282 | 0.02 | 17.73 | 42.41 | 2.68 |
| ARHGEF12 | 11:120207786-120360645 | 0.0014113 | 0.027 | 2.56 | 6.29 | 2.67 |
| RP11-603J24.17.1 | 12:56473640-56509935 | 0.0021172 | 0.033 | 11.61 | 28.07 | 2.67 |
| RP4-655J12.4.1 | 1:116966345-117113661 | 0.0035153 | 0.041 | 1.35 | 3.10 | 2.67 |
| PPM1A | 14:60706838-60765805 | 0.0003592 | 0.017 | 78.00 | 190.00 | 2.66 |
| STOM | 9:124101354-124132531 | 0.0023877 | 0.035 | 41.83 | 107.51 | 2.66 |
| FAM104A | 17:71188770-71258491 | 0.0011339 | 0.024 | 35.41 | 87.02 | 2.66 |
| RAB13 | 1:153954126-153958834 | 0.0003926 | 0.018 | 5.64 | 13.49 | 2.65 |
| TCP11L2 | 12:106631654-106740793 | 0.0000712 | 0.012 | 19.27 | 49.99 | 2.64 |
| SRRD | 22:26879842-26908471 | 0.0013596 | 0.026 | 20.41 | 49.37 | 2.63 |
| TFDP2 | 3:141663276-141868386 | 0.0001889 | 0.014 | 5.32 | 12.62 | 2.63 |
| ELL2 | 5:95220801-96143803 | 0.0000577 | 0.012 | 4.80 | 11.75 | 2.62 |
| RP11-526L8.1.1,SCLY,UBE2F | 2:238875468-239008054 | 0.0014307 | 0.027 | 72.18 | 164.72 | 2.61 |
| FBXO7 | 22:32870662-32894818 | 0.0020207 | 0.032 | 524.16 | 1154.25 | 2.61 |
| ACP1 | 2:217729-278283 | 0.0001049 | 0.013 | 43.08 | 106.36 | 2.60 |
| AC138035.1.1 | 5:180756241-180769214 | 0.0000522 | 0.012 | 1.06 | 2.74 | 2.60 |
| C9orf40 | 9:77561496-77567802 | 0.0000682 | 0.012 | 4.49 | 10.95 | 2.60 |
| RP11-322M19.1.1 | 10:88963609-89102369 | 0.000128 | 0.013 | 36.47 | 84.54 | 2.58 |
| TROAP | 12:49686413-49725514 | 0.0029099 | 0.038 | 0.96 | 2.40 | 2.56 |
| PIP5K1B | 9:71320574-71624092 | 0.0007389 | 0.02 | 3.66 | 8.73 | 2.56 |
| HJURP | 2:234684369-234763212 | 0.0016025 | 0.028 | 0.63 | 1.64 | 2.56 |
| AL031178.1,FBXO9 | 6:52866076-52965671 | 0.0022419 | 0.034 | 81.57 | 181.69 | 2.56 |
| PTMS | 12:6857169-6880118 | 0.0039428 | 0.043 | 49.87 | 105.86 | 2.56 |
| METTL22 | 16:8715539-8740081 | 0.0000388 | 0.012 | 13.06 | 33.59 | 2.56 |
| METTL9 | 16:21608538-21668794 | 0.0005313 | 0.019 | 154.87 | 365.71 | 2.56 |
| RP4-659J6.2.1 | 7:139877060-139879440 | 0.0017045 | 0.029 | 10.35 | 21.59 | 2.55 |
| PRUNE2 | 9:79226291-79521003 | 0.0028598 | 0.038 | 1.16 | 2.42 | 2.55 |
| CTA-363E6.6.1 | 16:19421817-19510435 | 0.0050253 | 0.048 | 108.73 | 220.08 | 2.53 |
| ERBB3,PA2G4,RP11-603J24.9.1 | 12:56473640-56509935 | 0.0004752 | 0.018 | 45.73 | 111.95 | 2.53 |
| OPTN | 10:12938626-13180291 | 0.0008243 | 0.021 | 63.11 | 148.94 | 2.52 |
| MAF1 | 8:145153535-145173218 | 0.0006562 | 0.02 | 105.62 | 248.87 | 2.52 |
| TSPAN5 | 4:99391517-99579780 | 0.0035616 | 0.041 | 33.24 | 71.95 | 2.52 |
| AC018642.1.1 | 7:130626518-131181395 | 0.0025012 | 0.035 | 1.21 | 2.74 | 2.52 |
| MARCH8,RP11-358L16.2.1 | 10:45950034-46090354 | 0.0028392 | 0.038 | 51.85 | 114.42 | 2.51 |
| CNPPD1 | 2:220036618-220050201 | 0.0006725 | 0.02 | 80.63 | 190.19 | 2.51 |
| HEBP1 | 12:13080659-13193613 | 0.0031566 | 0.039 | 33.59 | 81.82 | 2.51 |
| ANKRD34B | 5:79852573-79866307 | 0.00086 | 0.022 | 0.63 | 1.91 | 2.51 |
| SPTB | 14:65170819-65346601 | 0.0015603 | 0.028 | 7.29 | 16.08 | 2.51 |
| CTD-2007H13.3.1 | 5:98264874-98330717 | 0.0002622 | 0.016 | 0.77 | 2.26 | 2.51 |
| FSTL3 | 19:676388-683392 | 0.0000543 | 0.012 | 1.98 | 5.51 | 2.49 |
| GATA1 | X:48644961-48652718 | 0.0013658 | 0.026 | 31.49 | 70.59 | 2.49 |
| FHDC1 | 4:153857503-153900848 | 0.0039811 | 0.043 | 0.70 | 1.75 | 2.49 |
| FAM117A | 17:47778304-47906458 | 0.0005866 | 0.02 | 54.86 | 124.45 | 2.47 |
| PROS1 | 3:93591880-93692910 | 0.0012813 | 0.026 | 0.65 | 1.73 | 2.47 |
| PPP2R5B | 11:64685024-64703360 | 0.0002617 | 0.016 | 17.48 | 39.33 | 2.45 |
| MSI2 | 17:55333211-55757658 | 0.0004138 | 0.018 | 6.35 | 14.41 | 2.45 |
| MS4A4A | 11:60048013-60076445 | 0.0005764 | 0.02 | 9.20 | 21.14 | 2.44 |
| RANBP10 | 16:67757004-67840555 | 0.0008458 | 0.022 | 13.45 | 29.96 | 2.44 |
| ECSIT,ZNF653 | 19:11594244-11639987 | 0.0021478 | 0.033 | 27.83 | 60.76 | 2.44 |
| RP1-229K20.5.1 | 6:41237612-41237977 | 7.49E-06 | 0.005 | 10.12 | 23.74 | 2.44 |
| RNF10 | 12:120941076-121015397 | 0.0039817 | 0.043 | 301.82 | 638.75 | 2.43 |
| KCNH3 | 12:49932939-49961936 | 0.0001086 | 0.013 | 2.83 | 6.89 | 2.43 |
| POLR1D | 13:28194902-28241548 | 0.0003813 | 0.018 | 129.12 | 281.90 | 2.43 |
| PNPLA2 | 11:818901-842545 | 0.0038865 | 0.043 | 102.41 | 217.80 | 2.42 |
| SLC22A4 | 5:131520568-131731306 | 0.0020677 | 0.032 | 23.26 | 46.42 | 2.42 |
| FUNDC2 | X:154064062-154288578 | 0.0019256 | 0.031 | 109.84 | 239.47 | 2.42 |
| RPIA | 2:88991161-89050445 | 0.0002594 | 0.016 | 22.15 | 50.07 | 2.42 |
| IGKV1-5 | 2:89246818-89247475 | 0.0005898 | 0.02 | 30.67 | 82.95 | 2.42 |
| NSUN3,RP13-503K1.4.1 | 3:93698982-93847389 | 0.000043 | 0.012 | 6.13 | 14.61 | 2.41 |
| RNASE2 | 14:21423610-21424595 | 0.0008112 | 0.021 | 97.14 | 219.35 | 2.40 |
| ACSM3 | 16:20621564-20936328 | 0.0020553 | 0.032 | 2.88 | 6.68 | 2.40 |
| RNF123 | 3:49711434-49761384 | 0.0026225 | 0.036 | 13.68 | 28.81 | 2.40 |
| UROD | 1:45477818-45481247 | 0.0009953 | 0.024 | 34.62 | 79.88 | 2.40 |
| TRAPPC12-AS1 | 2:3383445-3488865 | 0.0033513 | 0.04 | 0.59 | 1.47 | 2.38 |
| VEGFA | 6:43737920-43754224 | 0.0005023 | 0.019 | 5.00 | 11.92 | 2.37 |
| ASCC2 | 22:30184596-30234271 | 0.0039552 | 0.043 | 100.01 | 218.30 | 2.36 |
| SCN1B | 19:35521591-35531352 | 0.0044485 | 0.045 | 1.88 | 4.13 | 2.36 |
| MARCH2 | 19:8478186-8503895 | 0.0047139 | 0.047 | 108.73 | 218.81 | 2.34 |
| FAM104B | X:55169534-55187743 | 0.0016741 | 0.029 | 7.40 | 16.22 | 2.31 |
| BABAM1 | 19:17378231-17390162 | 0.0005709 | 0.019 | 64.21 | 139.15 | 2.31 |
| B4GALT3 | 1:161136199-161147803 | 0.0004075 | 0.018 | 38.98 | 84.10 | 2.31 |
| CYB5A | 18:71920527-71959251 | 0.0000394 | 0.012 | 9.56 | 21.91 | 2.30 |
| PQLC1 | 18:77662419-77711664 | 0.0006753 | 0.02 | 34.90 | 74.46 | 2.29 |
| RP11-466C23.5.1 | 11:63602138-63602415 | 0.0000582 | 0.012 | 3.57 | 7.59 | 2.29 |
| PPARA | 22:46546423-46639653 | 0.0032929 | 0.039 | 1.41 | 2.95 | 2.29 |
| SMPD3 | 16:68344876-68482591 | 0.0034325 | 0.04 | 6.49 | 13.36 | 2.28 |
| HDGF | 1:156711898-156770607 | 0.0037555 | 0.042 | 142.09 | 291.08 | 2.26 |
| POP7 | 7:100303675-100305118 | 0.0019398 | 0.031 | 14.16 | 29.89 | 2.26 |
| MCOLN1 | 19:7587495-7598894 | 0.0017771 | 0.03 | 28.60 | 58.78 | 2.26 |
| RGS10 | 10:121259339-121302220 | 0.001373 | 0.026 | 184.99 | 354.85 | 2.25 |
| CCNDBP1,TMEM62 | 15:43398422-43513481 | 0.0005985 | 0.02 | 165.88 | 355.92 | 2.25 |
| FAM47E,RP11-67M24.5.1,STBD1 | 4:77135192-77232752 | 0.0030274 | 0.038 | 1.76 | 4.57 | 2.24 |
| E2F2 | 1:23832921-23857712 | 0.0031132 | 0.039 | 3.83 | 8.17 | 2.23 |
| C19orf59 | 19:7741942-7744719 | 0.004263 | 0.045 | 49.49 | 95.43 | 2.23 |
| ZNF451 | 6:56951641-57049735 | 0.0001044 | 0.013 | 6.02 | 13.02 | 2.23 |
| ACP5 | 19:11685476-11689801 | 0.0003006 | 0.017 | 20.92 | 45.76 | 2.23 |
| C17orf39 | 17:17942735-17971718 | 0.0006039 | 0.02 | 6.63 | 13.75 | 2.23 |
| EIF1B | 3:40351174-40353915 | 0.0006687 | 0.02 | 106.89 | 223.00 | 2.23 |
| RDH11,RP11-1012A1.4.1,VTI1B | 14:68086514-68162531 | 0.000474 | 0.018 | 66.88 | 144.95 | 2.22 |
| PRDX6 | 1:173446404-173457946 | 0.0031594 | 0.039 | 335.36 | 675.27 | 2.22 |
| RILP | 17:1549443-1553352 | 0.0031548 | 0.039 | 38.76 | 77.62 | 2.21 |
| MKRN1 | 7:140152839-140179369 | 0.0030734 | 0.039 | 225.85 | 453.83 | 2.21 |
| RAB2B | 14:21927178-21979517 | 0.0010587 | 0.024 | 8.50 | 18.00 | 2.20 |
| RNF187 | 1:228674761-228683467 | 0.0051217 | 0.049 | 124.84 | 258.00 | 2.20 |
| HIST2H2AA3 | 1:149813504-149814478 | 0.0038048 | 0.042 | 23.62 | 47.89 | 2.20 |
| PGLYRP1 | 19:46522414-46526556 | 0.0016891 | 0.029 | 110.30 | 238.24 | 2.19 |
| CARM1 | 19:10982252-11039357 | 0.0038464 | 0.043 | 20.45 | 40.27 | 2.19 |
| UQCR11 | 19:1597153-1605483 | 0.000027 | 0.012 | 43.87 | 93.40 | 2.17 |
| SUMO1P1 | 20:52491790-52492096 | 0.0013571 | 0.026 | 5.55 | 10.78 | 2.16 |
| FOLR3 | 11:71825914-71850936 | 0.000189 | 0.014 | 86.70 | 157.99 | 2.15 |
| DECR2,NME4 | 16:446724-462487 | 0.0002598 | 0.016 | 32.61 | 69.88 | 2.15 |
| RP11-597D13.7.1 | 4:159191529-159199828 | 0.00281 | 0.037 | 0.90 | 1.77 | 2.15 |
| RP11-67C2.2.1 | 10:45869660-45948569 | 0.0002294 | 0.015 | 13.42 | 27.58 | 2.14 |
| TFRC | 3:195754053-195809060 | 0.0028815 | 0.038 | 6.33 | 12.77 | 2.14 |
| AKT1S1 | 19:50372294-50392006 | 0.0033847 | 0.04 | 15.97 | 32.51 | 2.14 |
| CARHSP1 | 16:8768421-8962866 | 0.0030279 | 0.038 | 59.95 | 118.44 | 2.13 |
| BMP2K | 4:79697495-79860592 | 0.0017979 | 0.03 | 10.34 | 20.71 | 2.12 |
| BAG1 | 9:33218362-33264761 | 0.0032969 | 0.039 | 278.96 | 539.91 | 2.11 |
| RP11-84C10.2.1 | 14:21366143-21404414 | 0.0036628 | 0.042 | 11.36 | 22.19 | 2.10 |
| OAZ1 | 19:2269519-2273487 | 0.0027495 | 0.037 | 2523.45 | 4756.92 | 2.10 |
| RNF14 | 5:141323149-141369856 | 0.0001376 | 0.013 | 13.17 | 26.27 | 2.08 |
| TMEM63B | 6:44081193-44123256 | 0.0038845 | 0.043 | 10.67 | 20.89 | 2.08 |
| AC006547.13.1 | 22:20004536-20053600 | 0.0002226 | 0.015 | 9.75 | 19.80 | 2.08 |
| GCLC,RP1-27K12.2.1 | 6:53362138-53481969 | 0.0020689 | 0.032 | 4.98 | 9.86 | 2.07 |
| CLEC5A.1 | HG7_PATCH:141607612-141806547 | 0.0013222 | 0.026 | 1.93 | 4.23 | 2.07 |
| CLEC5A | 7:141607612-141806547 | 0.0013269 | 0.026 | 1.93 | 4.23 | 2.07 |
| AMFR | 16:56395363-56459450 | 0.0012849 | 0.026 | 28.80 | 55.08 | 2.06 |
| AC007036.5.1,C7orf41 | 7:30174425-30202378 | 0.0018004 | 0.03 | 20.85 | 40.76 | 2.06 |
| UBFD1 | 16:23474862-23585710 | 0.0000641 | 0.012 | 4.63 | 9.76 | 2.06 |
| C20orf196 | 20:5731038-5845020 | 0.0020851 | 0.032 | 9.72 | 19.67 | 2.05 |
| AOC3 | 17:41003200-41010138 | 0.0009715 | 0.023 | 2.93 | 5.66 | 2.05 |
| FAM19A2 | 12:62102039-62811211 | 7.20E-06 | 0.005 | 8.96 | 17.60 | 2.05 |
| CCDC23 | 1:43272722-43312093 | 0.0051828 | 0.049 | 42.88 | 81.57 | 2.04 |
| CTD-2007L18.5.1 | 11:68228185-68384179 | 0.0043305 | 0.045 | 1.03 | 2.03 | 2.04 |
| PCSK6 | 15:101835623-102065405 | 0.0044298 | 0.045 | 3.10 | 6.21 | 2.04 |
| AC004381.6.1 | 16:20621564-20936328 | 0.0006748 | 0.02 | 0.55 | 1.32 | 2.04 |
| ISCU | 12:108956357-108963160 | 0.0006673 | 0.02 | 125.81 | 251.23 | 2.04 |
| FAM134A | 2:220036618-220050201 | 0.0021302 | 0.033 | 97.20 | 181.58 | 2.03 |
| MICALL2 | 7:1468100-1499138 | 0.0004114 | 0.018 | 0.99 | 2.00 | 2.03 |
| RP11-506M13.3.1 | 10:81420644-81436855 | 0.0014749 | 0.027 | 1.45 | 3.05 | 2.03 |
| CTD-2015H6.3.1 | 5:79778111-79838382 | 0.0027749 | 0.037 | 0.86 | 1.88 | 2.02 |
| BIRC2 | 11:102217941-102249401 | 0.0001057 | 0.013 | 24.11 | 47.37 | 2.02 |
| TBC1D22B | 6:37179955-37300746 | 0.0007252 | 0.02 | 6.00 | 11.84 | 2.02 |
| TMEM183A | 1:202976513-202993976 | 0.000383 | 0.018 | 49.06 | 94.57 | 2.02 |
| AP2S1 | 19:47341422-47354203 | 0.0028143 | 0.037 | 105.48 | 198.39 | 2.01 |
| DYNLL1 | 12:120907652-120936296 | 0.0034872 | 0.04 | 177.34 | 347.91 | 2.01 |
| RP4-713B5.2.1 | 1:93850043-93850555 | 0.0053145 | 0.05 | 0.58 | 1.16 | 2.00 |
| PTPN4 | 2:120517206-120741394 | 0.004242 | 0.045 | 15.03 | 27.24 | 2.00 |
| RP11-265D17.2.1 | 11:12115542-12285334 | 0.0043047 | 0.045 | 44.83 | 80.81 | 2.00 |
| CTDSPL | 3:37903450-38025960 | 0.0048557 | 0.048 | 2.73 | 5.40 | 2.00 |
| C15orf61 | 15:67493370-67819628 | 0.0001756 | 0.014 | 2.97 | 6.00 | 2.00 |
| CNIH2 | 11:66036003-66056641 | 0.0042782 | 0.045 | 1.04 | 2.10 | 2.00 |
| MICAL2 | 11:12115542-12285334 | 0.0004076 | 0.018 | 26.97 | 50.90 | 2.00 |
| AF196779.12.1,PRAF2,WDR45 | X:48928819-48958108 | 0.0016294 | 0.029 | 122.48 | 231.42 | 1.99 |
| NTAN1 | 16:15068447-15248421 | 0.0032434 | 0.039 | 36.41 | 66.60 | 1.99 |
| SVIP | 11:22835344-22966939 | 0.0009559 | 0.023 | 7.85 | 14.78 | 1.98 |
| CMAS | 12:22199107-22589975 | 0.0019045 | 0.031 | 11.90 | 22.81 | 1.98 |
| GFOD2 | 16:67708435-67753324 | 0.0022656 | 0.034 | 7.80 | 14.92 | 1.98 |
| GALNT4,POC1B,POC1B-GALNT4 | 12:89813494-89920039 | 0.0001985 | 0.014 | 13.13 | 24.72 | 1.97 |
| SFRP2 | 4:154701743-154710272 | 0.0043944 | 0.045 | 1.65 | 3.30 | 1.96 |
| RGS6 | 14:72399155-73061833 | 0.0039415 | 0.043 | 3.01 | 5.21 | 1.96 |
| CENPV | 17:16229798-16256813 | 0.0029153 | 0.038 | 2.91 | 5.23 | 1.96 |
| OPA1 | 3:193310932-193415612 | 0.0004695 | 0.018 | 5.59 | 10.45 | 1.96 |
| ELOF1 | 19:11663857-11670051 | 0.0037397 | 0.042 | 77.73 | 137.47 | 1.95 |
| RP11-354E11.2.1 | 10:19492778-20079330 | 0.0037752 | 0.042 | 0.87 | 1.95 | 1.95 |
| RBM7,REXO2,RP11-212D19.4.1 | 11:114262164-114321001 | 0.0001845 | 0.014 | 32.93 | 63.73 | 1.95 |
| RP1-90J20.11.1 | 6:3025024-3027659 | 0.0011401 | 0.024 | 2.93 | 5.96 | 1.94 |
| DNAJB2 | 2:220143988-220151622 | 0.0029194 | 0.038 | 57.60 | 108.20 | 1.94 |
| VSTM1 | 19:54544078-54567207 | 0.0021186 | 0.033 | 28.90 | 56.21 | 1.94 |
| MBD5 | 2:148691731-149275805 | 0.0035704 | 0.041 | 18.66 | 33.59 | 1.93 |
| NMNAT3,RP11-553K23.2.1 | 3:139108656-139396859 | 0.0021457 | 0.033 | 1.53 | 2.91 | 1.93 |
| CLDN9 | 16:3062456-3064506 | 0.0001802 | 0.014 | 9.50 | 17.87 | 1.93 |
| ABCB10 | 1:229652328-229694442 | 0.0003205 | 0.017 | 5.42 | 9.88 | 1.92 |
| SERPINI1 | 3:167453030-167543356 | 0.0010653 | 0.024 | 1.37 | 2.67 | 1.92 |
| ASGR2 | 17:7004640-7018292 | 0.0031772 | 0.039 | 18.42 | 30.34 | 1.92 |
| PRKAR2B | 7:106685093-106802256 | 0.0024409 | 0.035 | 10.95 | 20.97 | 1.92 |
| SLC26A6 | 3:48663155-48672926 | 0.0042942 | 0.045 | 6.60 | 12.19 | 1.92 |
| C15orf63,SERF2 | 15:43803155-44095241 | 0.0040648 | 0.044 | 967.15 | 1701.44 | 1.91 |
| GPX4 | 19:1103935-1106786 | 0.0027544 | 0.037 | 164.19 | 305.85 | 1.91 |
| RP11-1070N10.3.1 | 14:95982472-95984248 | 0.0009069 | 0.023 | 0.56 | 1.15 | 1.90 |
| BTBD19 | 1:45274153-45281257 | 0.0032063 | 0.039 | 0.87 | 1.65 | 1.90 |
| FBXL4 | 6:99321333-99395849 | 0.0003568 | 0.017 | 3.74 | 6.98 | 1.89 |
| TBPL1 | 6:134273307-134308637 | 0.0000453 | 0.012 | 18.65 | 35.16 | 1.89 |
| MTHFD2L | 4:74979890-75168816 | 0.0010161 | 0.024 | 1.94 | 3.56 | 1.89 |
| RP5-848E13.3.1 | 1:43424719-43449029 | 0.0043727 | 0.045 | 0.78 | 1.70 | 1.89 |
| PDE4D | 5:58264864-59843484 | 0.0022714 | 0.034 | 1.67 | 3.27 | 1.88 |
| KAT2B | 3:20081514-20195896 | 0.0001823 | 0.014 | 19.28 | 34.68 | 1.88 |
| RP11-575L7.8.1 | 9:86582997-86595569 | 0.0046191 | 0.046 | 8.10 | 15.29 | 1.88 |
| SERPINB10,SERPINB2 | 18:61538925-61603345 | 0.0029509 | 0.038 | 4.93 | 9.61 | 1.87 |
| BRP44 | 1:167885966-168045081 | 0.0007774 | 0.021 | 33.31 | 60.40 | 1.87 |
| MIIP | 1:12079522-12092102 | 0.0012392 | 0.025 | 36.47 | 66.65 | 1.87 |
| CUL4A | 13:113831890-113919399 | 0.0019828 | 0.031 | 19.54 | 34.85 | 1.87 |
| AC114730.8.1 | 2:242673993-242708231 | 0.0002839 | 0.016 | 0.67 | 1.35 | 1.86 |
| PPOX | 1:161136199-161147803 | 0.0004241 | 0.018 | 11.25 | 20.52 | 1.86 |
| RBX1,XPNPEP3 | 22:41253080-41369313 | 0.003034 | 0.038 | 100.16 | 176.35 | 1.85 |
| SH3GLB2 | 9:131683173-131790582 | 0.0016432 | 0.029 | 40.06 | 70.50 | 1.85 |
| MAP4K5 | 14:50885218-51135056 | 0.0000329 | 0.012 | 3.57 | 6.61 | 1.85 |
| SRSF8 | 11:94800055-94804388 | 0.0014694 | 0.027 | 8.64 | 15.76 | 1.84 |
| BTF3 | 5:72794232-72801460 | 0.0053164 | 0.05 | 633.17 | 1093.99 | 1.84 |
| CHID1 | 11:867356-915058 | 0.0027435 | 0.037 | 17.71 | 30.88 | 1.84 |
| KIAA1586 | 6:56911346-56920023 | 0.0031587 | 0.039 | 1.95 | 3.57 | 1.83 |
| GGH | 8:63927637-63951730 | 0.0032815 | 0.039 | 2.88 | 5.17 | 1.83 |
| SLC25A38 | 3:39424838-39438679 | 0.0047874 | 0.047 | 19.96 | 33.80 | 1.83 |
| TTTY14 | Y:21034386-21239302 | 0.004674 | 0.047 | 1.31 | 2.53 | 1.82 |
| AC068491.1.1,AC108463.1 | 2:111965352-112268567 | 0.0006986 | 0.02 | 59.73 | 103.24 | 1.82 |
| IFT20 | 17:26646160-26662495 | 0.000068 | 0.012 | 23.51 | 42.24 | 1.82 |
| C21orf58 | 21:47720094-47743789 | 0.0007622 | 0.021 | 1.43 | 2.70 | 1.82 |
| C11orf91,CD59 | 11:33719806-33757991 | 0.001742 | 0.03 | 39.93 | 70.54 | 1.82 |
| IGFALS,RP11-31I10.4.1,SPSB3 | 16:1823207-1844972 | 0.0026645 | 0.037 | 56.07 | 97.21 | 1.81 |
| ZNF346 | 5:176449696-176508190 | 0.0005954 | 0.02 | 6.75 | 12.00 | 1.81 |
| PFDN5 | 12:53689074-53700961 | 0.0032329 | 0.039 | 598.59 | 1013.36 | 1.81 |
| RP5-1086K13.1.1 | 1:116966345-117113661 | 0.000679 | 0.02 | 2.53 | 4.49 | 1.81 |
| AIDA | 1:222791427-222908538 | 0.000524 | 0.019 | 25.00 | 43.77 | 1.81 |
| STOML2 | 9:35099887-35103154 | 0.0009322 | 0.023 | 32.10 | 55.80 | 1.80 |
| CTD-2574D22.3.1 | 16:29911695-29940254 | 0.0026763 | 0.037 | 1.06 | 2.02 | 1.80 |
| CLTCL1 | 22:19166985-19279239 | 0.0033557 | 0.04 | 3.54 | 6.77 | 1.79 |
| JAM3 | 11:133938819-134117686 | 0.0001905 | 0.014 | 1.25 | 2.34 | 1.79 |
| FAM211B | 22:24936405-25024972 | 0.0017353 | 0.03 | 0.66 | 1.21 | 1.79 |
| RP11-497H16.6.1 | 5:69803905-69808422 | 0.0005885 | 0.02 | 6.55 | 12.29 | 1.79 |
| C17orf57,ITGB3,RP11-290H9.2.1 | 17:45331207-45518678 | 0.0024338 | 0.035 | 5.05 | 9.65 | 1.78 |
| CTC-203F4.1.1,CTC-349C3.1.1 | 5:134362614-134735604 | 0.0002403 | 0.015 | 5.96 | 10.63 | 1.78 |
| NFE2 | 12:54624723-54745633 | 0.0052409 | 0.05 | 269.34 | 451.54 | 1.78 |
| SLC19A1 | 21:46825051-46964325 | 0.0010856 | 0.024 | 8.37 | 15.01 | 1.78 |
| FRMD4A | 10:13628926-14504141 | 0.0000758 | 0.012 | 5.65 | 9.77 | 1.77 |
| AOC2 | 17:40996608-41002722 | 0.0013659 | 0.026 | 5.47 | 9.37 | 1.77 |
| TSPAN17 | 5:176074387-176086058 | 0.0030302 | 0.038 | 15.69 | 25.65 | 1.76 |
| RP11-497H16.2.1 | 5:69783428-69786959 | 0.0004873 | 0.018 | 7.27 | 13.67 | 1.76 |
| ERMAP | 1:43272722-43312093 | 0.0018226 | 0.03 | 6.18 | 10.47 | 1.76 |
| MXD4 | 4:2249158-2264021 | 0.0043751 | 0.045 | 36.41 | 59.22 | 1.75 |
| TARS | 5:33440801-33469644 | 0.0003677 | 0.018 | 13.30 | 23.57 | 1.75 |
| SNX22 | 15:64443913-64455404 | 0.0004688 | 0.018 | 3.32 | 5.66 | 1.75 |
| LGALS12 | 11:63273555-63284246 | 0.0020588 | 0.032 | 8.76 | 15.59 | 1.75 |
| MBOAT2 | 2:8992819-9143942 | 0.0027699 | 0.037 | 27.74 | 45.55 | 1.75 |
| HK3 | 5:176237477-176326333 | 0.0002102 | 0.014 | 90.85 | 152.35 | 1.74 |
| PIP4K2A | 10:22823777-23003503 | 0.0049857 | 0.048 | 47.31 | 80.85 | 1.73 |
| CPEB4 | 5:173315282-173388979 | 0.0011717 | 0.025 | 6.06 | 10.55 | 1.73 |
| CTC-338M12.3.1,CTC-338M12.4.1 | 5:180663908-180699308 | 0.0005994 | 0.02 | 19.71 | 32.78 | 1.73 |
| MAN1A1 | 6:119498373-119670926 | 0.0006091 | 0.02 | 7.37 | 12.07 | 1.72 |
| RPL41 | 12:56510369-56511727 | 0.0019989 | 0.032 | 2587.64 | 4169.12 | 1.72 |
| ARL6IP1,RP11-1035H13.3.1,RPS15A | 16:18636821-18813000 | 0.001435 | 0.027 | 1243.27 | 2034.24 | 1.72 |
| GPR137 | 11:64037301-64056972 | 0.0018098 | 0.03 | 14.96 | 25.18 | 1.72 |
| ITGAM | 16:31271310-31344190 | 0.0000765 | 0.012 | 83.41 | 140.43 | 1.72 |
| RP11-115L11.1.1 | 4:15606161-15739936 | 0.0000441 | 0.012 | 6.31 | 10.91 | 1.71 |
| ODC1 | 2:10580093-10588630 | 0.0017436 | 0.03 | 48.00 | 80.68 | 1.71 |
| HRH4 | 18:22040592-22059921 | 0.0039577 | 0.043 | 1.45 | 2.54 | 1.71 |
| SPNS3 | 17:4337218-4391498 | 0.0010425 | 0.024 | 5.25 | 9.37 | 1.71 |
| GNMT | 6:42928495-42946958 | 0.0006182 | 0.02 | 1.63 | 2.77 | 1.70 |
| RP5-882C2.2.1 | 17:42299282-42301078 | 0.0033592 | 0.04 | 2.59 | 4.08 | 1.70 |
| MIR22,MIR22HG | 17:1614804-1619504 | 0.0004629 | 0.018 | 6.04 | 10.16 | 1.69 |
| UBAC1 | 9:138824814-138853226 | 0.0045709 | 0.046 | 23.10 | 37.67 | 1.69 |
| THBS3 | 1:155165378-155197214 | 0.000514 | 0.019 | 6.78 | 10.97 | 1.69 |
| ENTPD5 | 14:74318546-74551196 | 0.0001482 | 0.013 | 3.72 | 6.16 | 1.68 |
| RCOR3 | 1:211431718-211489727 | 0.000226 | 0.015 | 19.81 | 32.52 | 1.68 |
| AC025263.3.1,C12orf28,RAB3IP | 12:70132460-70748773 | 0.0037394 | 0.042 | 4.79 | 7.84 | 1.68 |
| MGST1 | 12:16500075-16763528 | 0.0020862 | 0.032 | 40.88 | 64.64 | 1.67 |
| HRSP12 | 8:99114571-99129469 | 0.0000441 | 0.012 | 4.50 | 7.51 | 1.67 |
| RP11-488L18.10.1 | 1:247350582-247351828 | 0.0024589 | 0.035 | 11.51 | 18.98 | 1.67 |
| THOC7 | 3:63727968-63849579 | 0.0011066 | 0.024 | 44.22 | 72.00 | 1.66 |
| UBE2C | 20:44441214-44445596 | 0.0048038 | 0.047 | 2.82 | 4.74 | 1.66 |
| PCMTD1 | 8:52730139-52811735 | 0.0011349 | 0.024 | 13.94 | 22.70 | 1.66 |
| CYP4F3 | 19:15751694-15773634 | 0.0017947 | 0.03 | 40.17 | 65.64 | 1.65 |
| PIK3R6 | 17:8706040-8770994 | 0.0022494 | 0.034 | 4.96 | 8.30 | 1.65 |
| JTB | 1:153931574-153950164 | 0.0036956 | 0.042 | 152.61 | 242.04 | 1.65 |
| RP11-245J9.2.1,RP11-245J9.4.1 | 3:63989697-64009658 | 0.0011302 | 0.024 | 1.41 | 2.50 | 1.65 |
| DCAF6 | 1:167885966-168045081 | 0.0015936 | 0.028 | 18.88 | 30.51 | 1.65 |
| RP11-989F5.3.1,STX2 | 12:131274144-131323811 | 0.001404 | 0.027 | 4.00 | 6.56 | 1.64 |
| PTS | 11:112097087-112140678 | 0.0011844 | 0.025 | 3.16 | 5.12 | 1.64 |
| NDUFA2 | 5:140018324-140042064 | 0.0012493 | 0.025 | 76.06 | 121.85 | 1.63 |
| RP11-809N8.4.1 | 11:73111531-73309234 | 0.0009075 | 0.023 | 9.54 | 14.83 | 1.63 |
| POMT2 | 14:77741298-77797940 | 0.0004914 | 0.018 | 1.84 | 3.03 | 1.62 |
| RP11-206L10.1.1 | 1:661610-663527 | 0.0029903 | 0.038 | 303.13 | 451.59 | 1.62 |
| MAFG | 17:79876148-79885588 | 0.0006314 | 0.02 | 7.04 | 11.04 | 1.62 |
| CCDC142,MRPL53 | 2:74699084-74722013 | 0.0007985 | 0.021 | 43.12 | 68.43 | 1.61 |
| RP11-356C4.5.1 | 16:90167797-90244752 | 0.0018479 | 0.03 | 4.38 | 6.89 | 1.61 |
| PSMD9,RP11-87C12.2.1,WDR66 | 12:122326636-122441833 | 0.0007104 | 0.02 | 37.75 | 59.55 | 1.60 |
| LINC00152 | 2:87754886-87906324 | 0.0002697 | 0.016 | 63.28 | 101.80 | 1.60 |
| RP4-811H24.6.1 | 1:32826870-32829913 | 0.0002526 | 0.016 | 5.18 | 8.18 | 1.60 |
| MGAT3 | 22:39795745-39914137 | 0.0012284 | 0.025 | 0.65 | 1.14 | 1.60 |
| PCK2 | 14:24549315-24602058 | 0.0026211 | 0.036 | 10.46 | 16.57 | 1.60 |
| UROS | 10:127477145-127511817 | 0.0014683 | 0.027 | 23.07 | 35.36 | 1.59 |
| LILRB5 | 19:54754262-54761167 | 0.0049171 | 0.048 | 1.24 | 2.01 | 1.59 |
| CCS | 11:66357639-66373490 | 0.0043924 | 0.045 | 26.02 | 41.46 | 1.59 |
| ASXL1 | 20:30946146-31027122 | 0.0029405 | 0.038 | 11.93 | 18.24 | 1.57 |
| CDK8 | 13:26828275-26979375 | 0.0003935 | 0.018 | 4.70 | 7.41 | 1.57 |
| RP11-298J20.4.1,ZRANB1 | 10:126605711-126849739 | 0.003336 | 0.04 | 11.03 | 16.83 | 1.57 |
| RPL9P9 | 15:82647359-82748784 | 0.0009494 | 0.023 | 88.22 | 133.15 | 1.56 |
| SIKE1 | 1:115312099-115323306 | 0.0045438 | 0.046 | 5.26 | 7.83 | 1.56 |
| RNASEH2C | 11:65479466-65488418 | 0.0023801 | 0.035 | 20.12 | 30.28 | 1.56 |
| RPS10 | 6:34385230-34393902 | 0.0009317 | 0.023 | 883.79 | 1335.33 | 1.56 |
| SLC22A15 | 1:116519118-116612675 | 0.0014286 | 0.027 | 8.77 | 14.02 | 1.56 |
| RP11-510J16.3.1 | 16:81993523-81996298 | 0.0039121 | 0.043 | 5.30 | 8.43 | 1.56 |
| LA16c-358B7.3.1 | 16:1355547-1377019 | 0.0042249 | 0.045 | 2.12 | 3.29 | 1.56 |
| AL391357.1,DDOST,PINK1-AS1 | 1:20959947-20988000 | 0.0030041 | 0.038 | 113.13 | 170.49 | 1.56 |
| RPL9P8 | 15:83023846-83108085 | 0.0009419 | 0.023 | 100.25 | 150.23 | 1.55 |
| RP11-304M2.2.1 | 11:127114-207428 | 0.0009845 | 0.023 | 109.88 | 160.77 | 1.55 |
| MARCH6 | 5:10353814-10435491 | 0.0001823 | 0.014 | 23.85 | 36.76 | 1.55 |
| MAPK13 | 6:36095585-36107842 | 0.0013656 | 0.026 | 19.18 | 28.59 | 1.55 |
| GCNT2 | 6:10492455-10629601 | 0.0008555 | 0.022 | 1.62 | 2.56 | 1.55 |
| LGALSL | 2:64681102-64688515 | 0.0024535 | 0.035 | 6.25 | 9.15 | 1.55 |
| RP3-475N16.1.1 | 6:42908101-42911708 | 0.0001452 | 0.013 | 8.82 | 13.37 | 1.55 |
| SWT1 | 1:185126211-185260897 | 0.0001717 | 0.014 | 2.13 | 3.39 | 1.54 |
| UFD1L | 22:19318220-19466738 | 0.0036382 | 0.041 | 54.01 | 80.55 | 1.54 |
| SLC36A1 | 5:150816606-150871942 | 0.0009186 | 0.023 | 10.08 | 15.00 | 1.54 |
| ABCC5 | 3:183637721-183735803 | 0.0017941 | 0.03 | 10.97 | 16.78 | 1.54 |
| SUGT1 | 13:53226843-53262433 | 0.0028955 | 0.038 | 20.41 | 30.47 | 1.54 |
| SYCP2L,TMEM14B | 6:10747972-10979553 | 0.0001797 | 0.014 | 51.61 | 77.79 | 1.54 |
| SUV39H2 | 10:14920818-14996431 | 0.0025107 | 0.036 | 0.87 | 1.44 | 1.54 |
| PLIN5 | 19:4522545-4535208 | 0.0033092 | 0.039 | 7.53 | 11.08 | 1.53 |
| LSM1 | 8:37962759-38070819 | 0.002255 | 0.034 | 23.01 | 34.81 | 1.53 |
| TSSK3 | 1:32826870-32829913 | 0.0006893 | 0.02 | 4.59 | 7.14 | 1.53 |
| DPY19L3 | 19:32896654-32976795 | 0.0031249 | 0.039 | 4.27 | 6.73 | 1.53 |
| ANP32B | 9:100745637-100778225 | 0.0036389 | 0.041 | 118.72 | 178.66 | 1.53 |
| UBL5 | 19:9938567-9940797 | 0.0021819 | 0.033 | 334.33 | 498.31 | 1.52 |
| NTNG2 | 9:135037333-135119921 | 0.0000197 | 0.01 | 14.26 | 21.43 | 1.52 |
| LTA4H | 12:96390298-96437298 | 0.0052836 | 0.05 | 160.88 | 236.09 | 1.52 |
| STK16 | 2:220094478-220142892 | 0.004999 | 0.048 | 33.85 | 49.66 | 1.52 |
| CLK3 | 15:74890840-74988633 | 0.0036264 | 0.041 | 68.20 | 99.61 | 1.52 |
| ATG14 | 14:55833109-55878576 | 0.0007149 | 0.02 | 3.89 | 5.89 | 1.51 |
| TBC1D25 | X:48397844-48420997 | 0.0016373 | 0.029 | 13.93 | 20.84 | 1.51 |
| HSPA7 | 1:161511548-161648444 | 0.0052973 | 0.05 | 25.01 | 38.24 | 1.51 |
| HBXIP | 1:110943870-110961033 | 0.0024664 | 0.035 | 90.35 | 133.95 | 1.51 |
| LRRFIP2 | 3:37034822-37225180 | 0.0001162 | 0.013 | 16.54 | 24.95 | 1.51 |
| AP2B1 | 17:33914281-34053434 | 0.0020112 | 0.032 | 24.43 | 36.20 | 1.51 |
| ATP8B3 | 19:1782073-1812275 | 0.0011514 | 0.025 | 1.26 | 2.00 | 1.51 |
| C7orf53,IFRD1 | 7:112063022-112130942 | 0.0038216 | 0.043 | 57.65 | 82.89 | 1.51 |
| TIGD1 | 2:233412778-233449247 | 0.0020366 | 0.032 | 0.75 | 1.27 | 1.51 |
| SLC37A3 | 7:139993492-140126050 | 0.0043958 | 0.045 | 13.31 | 19.36 | 1.51 |
| AC010507.1 | 19:200111-202173 | 0.0011123 | 0.024 | 76.94 | 111.61 | 1.50 |
| RP11-1407O15.2.1,TBC1D3 | 17:36337710-36421858 | 2.27E-06 | 0.004 | 12.91 | 19.58 | 1.50 |
| TBC1D17 | 19:50372294-50392006 | 0.003401 | 0.04 | 19.07 | 28.26 | 1.50 |
| HIVEP3 | 1:41972035-42501596 | 0.0016514 | 0.029 | 1.87 | 1.26 | -1.50 |
| NELF | 9:140342021-140353786 | 0.0000583 | 0.012 | 13.59 | 9.28 | -1.50 |
| TXNIP | 1:145438468-145442635 | 0.0032804 | 0.039 | 1070.62 | 692.55 | -1.50 |
| ATIC | 2:216176539-216214487 | 0.0013761 | 0.026 | 15.49 | 10.43 | -1.50 |
| IGFBP4 | 17:38599701-38613983 | 0.0040814 | 0.044 | 1.92 | 1.33 | -1.50 |
| DTX3 | 12:57998404-58003587 | 0.0026842 | 0.037 | 4.15 | 2.78 | -1.51 |
| MCM3 | 6:52128806-52149582 | 0.0048436 | 0.047 | 7.08 | 4.57 | -1.51 |
| PARP1 | 1:224102740-226595780 | 0.0030173 | 0.038 | 12.16 | 7.74 | -1.51 |
| LAP3 | 4:17578814-17616194 | 0.0011633 | 0.025 | 35.41 | 22.07 | -1.51 |
| ZNF766 | 19:52772823-52795977 | 0.0000845 | 0.012 | 5.59 | 3.73 | -1.51 |
| MFAP1 | 15:44096689-44117000 | 0.0005528 | 0.019 | 10.85 | 7.08 | -1.51 |
| IFIT5 | 10:90973325-91180758 | 0.0011074 | 0.024 | 9.97 | 6.68 | -1.51 |
| RPRD2 | 1:150335566-150449042 | 0.000209 | 0.014 | 3.49 | 2.34 | -1.51 |
| HNRNPR | 1:23630263-23670829 | 0.0003805 | 0.018 | 22.80 | 14.60 | -1.51 |
| ALDH18A1 | 10:97365695-97416463 | 0.0041876 | 0.045 | 3.42 | 2.26 | -1.51 |
| HLA-DRA | HSCHR6_MHC_DBB:32382334-32387545 | 0.0007053 | 0.02 | 109.44 | 69.19 | -1.52 |
| TSR1 | 17:2207247-2240678 | 0.0007655 | 0.021 | 3.00 | 1.99 | -1.52 |
| EIF2B3 | 1:45316449-45452282 | 6.36E-06 | 0.005 | 5.16 | 3.43 | -1.52 |
| PTPLAD1 | 15:65822755-65870687 | 0.0031775 | 0.039 | 3.85 | 2.55 | -1.53 |
| AKR1B1 | 7:134127126-134144036 | 0.0010903 | 0.024 | 36.18 | 23.11 | -1.53 |
| APBB1 | 11:6416353-6440644 | 0.0047259 | 0.047 | 9.62 | 6.12 | -1.54 |
| IFIH1 | 2:163123588-163175213 | 0.0013107 | 0.026 | 9.38 | 5.95 | -1.54 |
| C4orf42 | 4:1205235-1245224 | 0.0033882 | 0.04 | 2.91 | 1.90 | -1.54 |
| NSG1.1 | 4:4349866-4639631 | 0.0010266 | 0.024 | 5.43 | 3.43 | -1.54 |
| SIDT1 | 3:113251142-113348425 | 0.0028526 | 0.038 | 5.49 | 3.51 | -1.54 |
| ZNF669 | 1:247261405-247267674 | 0.0014003 | 0.027 | 3.05 | 2.04 | -1.55 |
| SERPINH1 | 11:75273100-75283832 | 0.0018906 | 0.031 | 2.09 | 1.42 | -1.55 |
| TRBV6-2 | HG7_PATCH:142049014-142049495 | 0.0052718 | 0.05 | 9.00 | 5.22 | -1.55 |
| TRBV6-3 | HG7_PATCH:142070667-142071148 | 0.0052718 | 0.05 | 9.00 | 5.22 | -1.55 |
| CTD-2135J3.4.1,PINX1,SOX7 | 8:10581277-10697394 | 0.0027733 | 0.037 | 5.14 | 3.39 | -1.55 |
| UMPS | 3:124449212-124464040 | 0.0043914 | 0.045 | 6.08 | 3.73 | -1.55 |
| AC113189.5.1 | 17:7482921-7518215 | 0.0006673 | 0.02 | 3.44 | 2.32 | -1.55 |
| WBP11 | 12:14939409-14956474 | 0.0000494 | 0.012 | 16.28 | 10.30 | -1.56 |
| WARS | 14:100800124-100996640 | 0.0010973 | 0.024 | 142.29 | 90.55 | -1.56 |
| CX3CR1 | 3:39304984-39323226 | 0.0000366 | 0.012 | 51.82 | 32.59 | -1.56 |
| IL2RB | 22:37515125-37595425 | 0.0032599 | 0.039 | 19.35 | 12.29 | -1.56 |
| PLD4 | 14:105391152-105399574 | 0.0035194 | 0.041 | 6.74 | 3.92 | -1.56 |
| LRBA | 4:151185593-151936879 | 0.004896 | 0.048 | 3.49 | 2.13 | -1.57 |
| CD8A | 2:87011728-87035519 | 0.0015138 | 0.028 | 53.37 | 33.56 | -1.57 |
| ZNF568 | 19:37407230-37488834 | 0.00345 | 0.04 | 1.28 | 0.87 | -1.57 |
| KHK | 2:27309614-27341995 | 0.0032423 | 0.039 | 1.63 | 1.04 | -1.58 |
| SH2D2A | 1:156776034-156851642 | 0.000845 | 0.022 | 10.16 | 6.34 | -1.58 |
| HSPA9 | 5:137890570-137911133 | 0.0001204 | 0.013 | 20.56 | 12.96 | -1.58 |
| OLFM2 | 19:9964394-10047070 | 0.0045592 | 0.046 | 3.19 | 2.01 | -1.58 |
| CD2 | 1:117297006-117311850 | 0.0047954 | 0.047 | 68.75 | 41.76 | -1.58 |
| LAS1L | X:64732461-64754655 | 0.0043484 | 0.045 | 6.02 | 3.73 | -1.58 |
| MAP4K1 | 19:39078280-39108643 | 0.0006621 | 0.02 | 16.61 | 10.43 | -1.59 |
| WDR89 | 14:64063756-64108579 | 0.0012307 | 0.025 | 3.10 | 1.98 | -1.59 |
| NOLC1 | 10:103911932-103923627 | 0.0000732 | 0.012 | 6.07 | 3.79 | -1.59 |
| TRIM32 | 9:119187503-120177348 | 0.0027486 | 0.037 | 1.49 | 0.95 | -1.59 |
| STAT4 | 2:191886251-192016322 | 0.0028762 | 0.038 | 16.11 | 9.48 | -1.60 |
| GBP2,GBP7 | 1:89571814-89641723 | 0.00005 | 0.012 | 172.66 | 107.46 | -1.60 |
| ALDOC | 17:26900132-26903952 | 0.0047924 | 0.047 | 12.43 | 7.01 | -1.60 |
| TRAC | 14:23016446-23021083 | 0.0042575 | 0.045 | 229.65 | 135.66 | -1.60 |
| IFIT2 | 10:90973325-91180758 | 0.0027817 | 0.037 | 70.48 | 40.54 | -1.61 |
| CD247 | 1:167399876-167487847 | 0.0014434 | 0.027 | 71.50 | 43.51 | -1.61 |
| NUDT7 | 16:77756410-77776897 | 0.0019523 | 0.031 | 1.54 | 1.02 | -1.62 |
| TTC9 | 14:71108503-71142077 | 0.0011206 | 0.024 | 6.31 | 3.61 | -1.62 |
| RP11-169D4.2.1 | 11:72287184-72385635 | 0.00441 | 0.045 | 2.13 | 1.32 | -1.62 |
| GPATCH4 | 1:156564278-156571288 | 0.0019064 | 0.031 | 4.54 | 2.80 | -1.62 |
| MAGED1 | X:51546102-51645453 | 0.0020165 | 0.032 | 3.98 | 2.40 | -1.62 |
| SBK1 | 16:28303839-28335170 | 0.001213 | 0.025 | 3.03 | 1.91 | -1.62 |
| AC005280.1 | 14:73945188-74025651 | 0.0006988 | 0.02 | 4.57 | 2.80 | -1.63 |
| C9orf91 | 9:117373485-117408702 | 0.002634 | 0.036 | 7.31 | 4.41 | -1.63 |
| TRBV9 | 7:142239536-142240058 | 0.0045521 | 0.046 | 8.40 | 5.12 | -1.63 |
| TEF | 22:41763336-41795330 | 0.000143 | 0.013 | 2.13 | 1.35 | -1.63 |
| RUNX3 | 1:25226001-25291612 | 0.0002048 | 0.014 | 28.16 | 17.10 | -1.63 |
| RP11-264M12.2.1 | 16:77756410-77776897 | 0.0024533 | 0.035 | 2.69 | 1.66 | -1.64 |
| DPP4 | 2:162848750-162931679 | 0.0052122 | 0.05 | 5.11 | 3.12 | -1.64 |
| COL6A2 | 21:47518010-47552763 | 0.0048132 | 0.047 | 4.08 | 2.46 | -1.65 |
| ZNF773 | 19:58011308-58024441 | 0.0000285 | 0.012 | 1.95 | 1.23 | -1.65 |
| DDX58 | 9:32455299-32526322 | 0.0001399 | 0.013 | 14.21 | 8.47 | -1.65 |
| BMS1 | 10:43278248-43326856 | 0.000866 | 0.022 | 3.06 | 1.85 | -1.65 |
| WDR3 | 1:118472342-118727846 | 0.000198 | 0.014 | 2.41 | 1.48 | -1.65 |
| TRDC | 14:22849082-22951948 | 0.0020374 | 0.032 | 37.25 | 21.15 | -1.66 |
| MSH2 | 2:47630107-47798078 | 0.0007411 | 0.02 | 2.60 | 1.61 | -1.66 |
| PRF1 | 10:72357103-72362531 | 0.0031996 | 0.039 | 53.74 | 31.98 | -1.67 |
| GIMAP4 | 7:150264364-150271041 | 0.000036 | 0.012 | 208.88 | 125.07 | -1.67 |
| APBA2 | 15:29129628-29410518 | 0.0031269 | 0.039 | 4.08 | 2.33 | -1.67 |
| DUSP5 | 10:112257595-112271302 | 0.0000466 | 0.012 | 6.55 | 4.02 | -1.68 |
| GNLY | 2:85912297-85925977 | 0.0008303 | 0.022 | 366.74 | 221.05 | -1.68 |
| STAT1 | 2:191745522-191885686 | 0.0001359 | 0.013 | 79.30 | 46.09 | -1.68 |
| PRKCH,RP11-47I22.3.1,RP11-47I22.4.1 | 14:61654276-62125414 | 0.000801 | 0.021 | 28.75 | 16.87 | -1.69 |
| ZNF416 | 19:58082934-58090243 | 0.0000722 | 0.012 | 1.62 | 1.02 | -1.69 |
| NCL | 2:232318241-232348352 | 0.0002589 | 0.016 | 45.77 | 26.15 | -1.69 |
| CD40LG | X:135730351-135742549 | 0.0025789 | 0.036 | 7.57 | 4.45 | -1.69 |
| AMIGO1 | 1:110046796-110052360 | 0.0027831 | 0.037 | 1.82 | 1.12 | -1.69 |
| HLA-DPB1 | HSCHR6_MHC_QBL:32961399-32984034 | 0.0001428 | 0.013 | 19.52 | 11.16 | -1.70 |
| OAS2 | 12:113344581-113455556 | 0.0009048 | 0.023 | 18.81 | 10.15 | -1.70 |
| BATF2 | 11:64755414-64764517 | 0.002978 | 0.038 | 8.98 | 4.86 | -1.71 |
| PPAP2A,RNF138P1 | 5:54603587-54830878 | 0.0027291 | 0.037 | 3.04 | 1.78 | -1.71 |
| TRBV20-1 | HG7_PATCH:142368230-142368981 | 0.0015404 | 0.028 | 27.39 | 15.53 | -1.73 |
| HLCS | 21:38071072-38362536 | 0.0020563 | 0.032 | 1.44 | 0.85 | -1.73 |
| HLA-DOA | 6:32971954-32977389 | 0.0013329 | 0.026 | 1.26 | 0.77 | -1.74 |
| DLG3 | X:69664710-69725337 | 0.0035083 | 0.041 | 1.22 | 0.73 | -1.74 |
| PYHIN1 | 1:158900585-158946844 | 0.0016021 | 0.028 | 11.15 | 6.02 | -1.75 |
| ROBO3 | 11:124735281-124751366 | 0.0033115 | 0.039 | 2.60 | 1.47 | -1.75 |
| REEP6 | 19:1491022-1497926 | 0.0053093 | 0.05 | 1.31 | 0.74 | -1.76 |
| PRAGMIN.1 | 8:8175257-8244008 | 0.0008209 | 0.021 | 1.74 | 1.00 | -1.76 |
| GBP1 | 1:89518001-89531043 | 0.0006317 | 0.02 | 47.05 | 25.61 | -1.76 |
| CTC-378H22.2.1 | 19:42592649-42700737 | 0.0034387 | 0.04 | 2.19 | 1.25 | -1.76 |
| EPSTI1 | 13:43460523-43566407 | 0.0007812 | 0.021 | 17.52 | 9.13 | -1.76 |
| TRBV19 | HG7_PATCH:142360431-142361115 | 0.0027328 | 0.037 | 15.67 | 8.33 | -1.78 |
| CASP5 | 11:104864961-104893895 | 0.0011646 | 0.025 | 7.68 | 4.57 | -1.79 |
| HLA-DPA1 | 6:33032345-33054978 | 2.95E-06 | 0.004 | 39.84 | 21.75 | -1.79 |
| GZMK | 5:54320080-54330398 | 0.0019722 | 0.031 | 24.64 | 11.76 | -1.80 |
| TRAV29DV5 | 14:22631133-22631766 | 0.0006222 | 0.02 | 10.18 | 5.62 | -1.81 |
| TRAV3 | 14:22191959-22192568 | 0.0044535 | 0.045 | 5.58 | 3.00 | -1.82 |
| DDX60 | 4:169137443-169239958 | 0.0051222 | 0.049 | 7.57 | 4.04 | -1.83 |
| IFT172 | 2:27665232-27712656 | 0.0031501 | 0.039 | 2.70 | 1.43 | -1.84 |
| RTN1 | 14:60062693-60337684 | 0.0022787 | 0.034 | 9.55 | 4.71 | -1.84 |
| TBX21 | 17:45810609-45823485 | 0.0013369 | 0.026 | 13.95 | 7.01 | -1.84 |
| PID1 | 2:229715241-230136001 | 0.0006516 | 0.02 | 7.88 | 4.05 | -1.85 |
| TRBV3-1 | 7:142008364-142008871 | 0.0010453 | 0.024 | 10.08 | 5.43 | -1.91 |
| GATA3 | 10:8095566-8117161 | 0.0008059 | 0.021 | 4.33 | 2.27 | -1.92 |
| IFIT3 | 10:90973325-91180758 | 0.0017522 | 0.03 | 86.57 | 41.47 | -1.92 |
| PLA2G7 | 6:46620677-46703430 | 0.0003059 | 0.017 | 6.27 | 2.95 | -1.96 |
| KIR2DS4 | 19:55235968-55378662 | 0.0006539 | 0.02 | 1.57 | 0.97 | -1.98 |
| TRAV23DV6 | 14:22554683-22555238 | 0.0011418 | 0.024 | 6.93 | 3.71 | -1.98 |
| SERPING1 | 11:57364859-57382326 | 0.0010386 | 0.024 | 22.00 | 9.94 | -1.98 |
| IFIT1 | 10:90973325-91180758 | 0.0032751 | 0.039 | 37.23 | 17.01 | -2.01 |
| GBP5 | 1:89724632-89738544 | 0.0004853 | 0.018 | 69.42 | 28.91 | -2.03 |
| HLA-DRB3 | HSCHR6_MHC_QBL:32417556-32430644 | 0.0008169 | 0.021 | 55.41 | 33.59 | -2.04 |
| HERC5 | 4:89378267-89427314 | 0.0017052 | 0.029 | 12.77 | 6.05 | -2.04 |
| EOMES | 3:27757439-27764206 | 0.0005432 | 0.019 | 5.85 | 2.69 | -2.04 |
| ALDH1B1 | 9:38392660-38398658 | 0.0001183 | 0.013 | 1.25 | 0.64 | -2.08 |
| ETV7 | 6:36322418-36458920 | 0.0042065 | 0.045 | 3.75 | 2.05 | -2.11 |
| ATP1A3 | 19:42470733-42498384 | 0.0014222 | 0.027 | 1.23 | 0.60 | -2.14 |
| NDRG2 | 14:21484921-21572881 | 0.0000378 | 0.012 | 5.33 | 2.42 | -2.20 |
| HLA-DQA1 | 6:32595955-32614839 | 0.0043823 | 0.045 | 56.74 | 25.58 | -2.26 |
| HLA-DRB1 | 6:32546545-32557625 | 0.001843 | 0.03 | 33.99 | 29.43 | -2.29 |
| KIR2DL4,KIR3DL1,KIR3DL2,KIR3DL3 | 19:55235968-55378662 | 0.00121 | 0.025 | 1.94 | 0.87 | -2.35 |
| CXCL10 | 4:76932336-77033955 | 0.0007667 | 0.021 | 3.50 | 1.51 | -2.45 |
| ENHO | 9:34521037-34524239 | 0.0042023 | 0.045 | 1.89 | 0.66 | -2.51 |
| GS1-251I9.3.1 | 8:92006023-92053292 | 0.005229 | 0.05 | 1.41 | 0.35 | -2.84 |

Table S4. Differentially expressed transcripts (FDR < 0.05) before LVAD and 6 months (180 days) later with fold change greater than 1.5. Transcripts with a median FPKM <1 at both time points were removed.

| **Gene Symbol** | **Locus** | **p** | **q** | **Mean FPKM pre-LVAD** | **Mean FPKM 180 days post LVAD** | **Fold Change** |
| --- | --- | --- | --- | --- | --- | --- |
| AL592188.2 | GL000220.1:107447-107561 | 0.0043772 | 0.022 | 159.04 | 3477.23 | 293706.10 |
| AL592188.1 | GL000220.1:151419-151533 | 0.0043772 | 0.022 | 159.04 | 3477.23 | 293706.10 |
| SNORA75 | 2:232318241-232348352 | 0.0153803 | 0.049 | 36.46 | 45.06 | 3845.59 |
| IGLC5 | 22:23257804-23258097 | 0.0000506 | 0.005 | 0.14 | 3.11 | 192.70 |
| RPS26P3 | 9:8314245-10612723 | 0.0052648 | 0.025 | 0.60 | 6.57 | 178.38 |
| AC084031.1.1 | 2:231577559-231685790 | 0.0041869 | 0.022 | 0.06 | 1.23 | 86.36 |
| UQCRFS1P1 | 22:40139048-40289868 | 0.0050658 | 0.024 | 0.16 | 1.25 | 58.40 |
| RPL12P4 | 20:53691154-53691685 | 0.0019477 | 0.015 | 0.19 | 1.45 | 56.34 |
| MFI2-AS1 | 3:196715491-196756687 | 0.0155067 | 0.049 | 0.49 | 1.57 | 45.75 |
| CTB-63M22.1.1 | 5:165809309-165809604 | 0.0042239 | 0.022 | 25.18 | 206.74 | 39.19 |
| AL161626.1 | 9:79186648-79186950 | 0.001928 | 0.015 | 58.26 | 401.04 | 14.01 |
| AP003035.1 | 11:83166054-85338966 | 0.0014789 | 0.013 | 1332.77 | 7032.07 | 7.95 |
| RPS26P6 | 8:101907973-101908387 | 0.0067706 | 0.029 | 14.46 | 39.25 | 7.44 |
| RN28S1 | X:108297360-108297792 | 0.0012264 | 0.012 | 2307.45 | 11583.09 | 7.08 |
| RP11-345J4.6.1 | 16:29262828-29606395 | 0.0061528 | 0.027 | 216.89 | 724.99 | 6.21 |
| LAMB2 | 3:49158546-49170551 | 0.0159949 | 0.05 | 1.08 | 4.37 | 6.12 |
| AC079949.1 | 12:127650452-127650987 | 0.0055768 | 0.026 | 125.56 | 462.24 | 5.66 |
| AC073621.1.1 | 17:17286690-17287326 | 0.011569 | 0.04 | 31.37 | 99.78 | 4.62 |
| C4orf48 | 4:2043688-2045697 | 0.0014277 | 0.013 | 19.26 | 68.91 | 4.59 |
| ZNF32-AS1 | 10:44124264-44170151 | 0.0119758 | 0.041 | 1.99 | 4.04 | 4.48 |
| RP11-569A11.1.1 | 1:202559723-202679545 | 0.0114918 | 0.04 | 1.84 | 7.65 | 4.41 |
| AL592188.10 | GL000220.1:115008-115124 | 0.0099368 | 0.037 | 687923.40 | 2127143.00 | 4.37 |
| AL592188.7 | GL000220.1:158980-159096 | 0.0099368 | 0.037 | 687923.40 | 2127143.00 | 4.37 |
| SCAND1 | 20:34541538-34547394 | 0.0052296 | 0.025 | 32.81 | 96.03 | 4.19 |
| RP11-20I23.7.1 | 16:2587964-2653189 | 0.0125212 | 0.043 | 3.64 | 11.14 | 4.17 |
| AC093503.1 | 19:47150868-47220384 | 0.0019484 | 0.015 | 1.30 | 3.77 | 4.14 |
| CTC-575D19.1.1 | 5:168043316-168044059 | 0.0112186 | 0.04 | 2.17 | 4.84 | 4.11 |
| TMSL4 | 9:131102924-131123502 | 6.30E-06 | 0.003 | 2.64 | 10.32 | 4.10 |
| TPGS1 | 19:507496-519653 | 0.0112423 | 0.04 | 10.02 | 25.67 | 3.89 |
| AC023490.1.1,AC023490.2.1 | 22:20377668-20380440 | 0.0144505 | 0.047 | 2.26 | 5.35 | 3.65 |
| TRBV25-1 | 7:142378571-142379076 | 0.0115396 | 0.04 | 0.76 | 1.71 | 3.64 |
| COMTD1 | 10:76993413-76995788 | 0.0018925 | 0.014 | 5.17 | 15.16 | 3.56 |
| XX-2136C48.7.1 | 10:135505634-135505917 | 0.000672 | 0.009 | 2.21 | 6.18 | 3.54 |
| PRKCDBP | 11:6340175-6341877 | 0.003773 | 0.021 | 0.71 | 2.35 | 3.51 |
| PCSK1N | X:48689503-48694035 | 0.0084417 | 0.033 | 6.51 | 15.36 | 3.48 |
| IGHG4 | 14:106090686-106092403 | 0.0112589 | 0.04 | 2.97 | 7.14 | 3.47 |
| LCNL1,PTGDS | 9:139871955-139880862 | 0.0002118 | 0.006 | 13.30 | 42.18 | 3.46 |
| TMEM238 | 19:55890612-55895627 | 0.0001233 | 0.006 | 1.87 | 5.78 | 3.39 |
| JOSD2 | 19:51009254-51014435 | 0.0035728 | 0.02 | 15.79 | 40.81 | 3.31 |
| RP11-31I10.2.1 | 16:1823207-1844972 | 0.0000189 | 0.004 | 3.82 | 12.14 | 3.30 |
| AL162151.3.1 | 14:99439650-99439808 | 0.0081855 | 0.032 | 247.57 | 575.79 | 3.23 |
| IGHV4-31 | 14:106805208-106805716 | 0.0033747 | 0.019 | 2.42 | 7.30 | 3.22 |
| MMP23A | 1:1631368-1633249 | 0.0001247 | 0.006 | 0.98 | 2.77 | 3.20 |
| FAM176B | 1:36771987-36794818 | 0.0032168 | 0.019 | 18.33 | 48.40 | 3.19 |
| FAM173A | 16:691812-818865 | 0.0040569 | 0.021 | 8.93 | 22.82 | 3.18 |
| RPL24P2 | 20:21095363-21095838 | 0.0039491 | 0.021 | 8.08 | 21.47 | 3.15 |
| CASC1 | 12:25261353-25348096 | 0.011002 | 0.039 | 0.47 | 1.22 | 3.14 |
| PTGFRN | 1:117452678-117532980 | 0.0054237 | 0.025 | 0.28 | 0.89 | 3.14 |
| RP11-998D10.7.1 | 14:21484921-21572881 | 0.0033177 | 0.019 | 1.47 | 3.86 | 3.12 |
| RP5-902P8.10.1,SCNN1D | 1:1210602-1227409 | 0.0130026 | 0.044 | 0.91 | 2.27 | 3.05 |
| RP11-247A12.2.1 | 9:131937834-131972827 | 0.0055739 | 0.026 | 0.81 | 2.09 | 3.05 |
| AL928768.3.1 | 14:106170300-106170939 | 0.0010298 | 0.011 | 8.50 | 20.81 | 3.03 |
| CLIC3 | 9:139889086-139891255 | 0.0001228 | 0.006 | 12.33 | 36.18 | 3.00 |
| C1orf138 | 1:150533479-150533969 | 0.002312 | 0.016 | 3.50 | 8.80 | 3.00 |
| IGHA1 | 14:106173456-106175002 | 0.0092409 | 0.035 | 85.53 | 307.63 | 2.99 |
| IGLC1,IGLJ1,IGLL5 | 22:23229959-23238014 | 0.0050102 | 0.024 | 66.24 | 197.81 | 2.98 |
| LA16c-390E6.5.1 | 16:1494934-1525581 | 0.0005675 | 0.009 | 10.15 | 27.65 | 2.98 |
| CPSF1.1,MIR939.1 | HG243_PATCH:145428003-145464191 | 8.75E-06 | 0.003 | 6.83 | 19.53 | 2.97 |
| GPR25 | 1:200842082-200843306 | 0.0024342 | 0.016 | 1.00 | 2.72 | 2.93 |
| C19orf60 | 19:18699494-18703146 | 0.0015124 | 0.013 | 26.50 | 67.34 | 2.90 |
| ZDHHC19 | 3:195924319-195938308 | 0.0122618 | 0.042 | 1.34 | 4.35 | 2.88 |
| CCR10 | 17:40830906-40833861 | 0.0035621 | 0.02 | 0.57 | 1.42 | 2.86 |
| GADD45GIP1 | 19:13064971-13068050 | 0.0012018 | 0.012 | 24.68 | 62.51 | 2.84 |
| IGLC2 | 22:23243155-23243617 | 0.0044156 | 0.023 | 168.66 | 542.91 | 2.83 |
| ZNF579 | 19:56088892-56092211 | 0.0003809 | 0.007 | 1.39 | 3.56 | 2.83 |
| UCN | 2:27505259-27603657 | 0.0087973 | 0.034 | 1.11 | 2.61 | 2.82 |
| METRN | 16:691812-818865 | 0.0049931 | 0.024 | 2.75 | 7.06 | 2.82 |
| IGLC3 | 22:23248511-23248973 | 0.0068169 | 0.029 | 194.61 | 560.55 | 2.82 |
| RPSAP9 | X:86958276-86959307 | 0.0036317 | 0.02 | 1.36 | 2.86 | 2.81 |
| AC135050.1 | 16:31072163-31085641 | 0.0025382 | 0.017 | 1.90 | 4.61 | 2.80 |
| HES4 | 1:934341-935552 | 0.0025836 | 0.017 | 2.91 | 7.70 | 2.77 |
| RP11-395B7.4.1 | 7:100612903-100662230 | 0.0119833 | 0.041 | 1.35 | 2.92 | 2.75 |
| SYCP3 | 12:102090724-102137918 | 0.009094 | 0.035 | 1.63 | 3.91 | 2.73 |
| TMEM160 | 19:47549167-47551882 | 0.0011489 | 0.012 | 24.39 | 59.37 | 2.72 |
| RP11-274B21.3.1 | 7:128294022-128294776 | 0.0025539 | 0.017 | 30.71 | 73.36 | 2.72 |
| MIB2 | 1:1550794-1565990 | 0.0004662 | 0.008 | 16.19 | 42.00 | 2.72 |
| PTRH1,RP11-56D16.2.1,RP11-56D16.8.1 | 9:130374539-130497604 | 0.0006094 | 0.009 | 4.38 | 10.64 | 2.71 |
| GZMM | 19:544026-549919 | 0.0006361 | 0.009 | 40.36 | 106.44 | 2.70 |
| FAM212A | 3:49840686-49842463 | 0.0002683 | 0.007 | 1.30 | 3.25 | 2.70 |
| AC079742.4.1 | 7:6617064-6629005 | 0.0053912 | 0.025 | 5.57 | 11.93 | 2.70 |
| DOC2GP | 11:67380123-67383728 | 0.0020253 | 0.015 | 0.82 | 1.91 | 2.69 |
| FBXL15 | 10:104162375-104182893 | 0.0040081 | 0.021 | 9.05 | 20.71 | 2.69 |
| RAMP1 | 2:238767535-238820756 | 0.0065773 | 0.028 | 0.62 | 1.53 | 2.69 |
| KCNMB3,LRRFIP1P1 | 3:178865901-178984790 | 0.0008519 | 0.01 | 1.23 | 2.85 | 2.67 |
| ZNF296 | 19:45574758-45579845 | 0.0004268 | 0.008 | 2.60 | 6.18 | 2.67 |
| RP11-322M19.2.1 | 10:89102680-89115187 | 0.0090718 | 0.035 | 0.80 | 1.80 | 2.66 |
| RP4-811H24.6.1 | 1:32826870-32829913 | 0.0008997 | 0.01 | 5.49 | 13.15 | 2.66 |
| PEX11G | 19:7541760-7553905 | 0.0015027 | 0.013 | 0.79 | 1.66 | 2.66 |
| PRSS57 | 19:685547-695461 | 0.0079985 | 0.032 | 1.12 | 2.38 | 2.66 |
| CPSF1,MIR1234,MIR939 | 8:145596789-145634753 | 0.0000302 | 0.004 | 7.80 | 19.67 | 2.63 |
| TMEM121 | 14:105992939-105996539 | 0.0033516 | 0.019 | 0.75 | 1.63 | 2.59 |
| CES1P1 | 16:55794459-55828070 | 0.0098063 | 0.036 | 2.78 | 5.70 | 2.58 |
| IGHA2 | 14:106053225-106054732 | 0.0149344 | 0.048 | 36.00 | 99.93 | 2.57 |
| CD7 | 17:80272745-80275480 | 0.000149 | 0.006 | 39.17 | 96.89 | 2.55 |
| GZMB | 14:25064928-25126980 | 0.0008992 | 0.01 | 67.45 | 159.49 | 2.55 |
| CCDC85B | 11:65657874-65659105 | 0.000123 | 0.006 | 26.46 | 64.94 | 2.53 |
| C2orf62 | 2:219135114-219232822 | 0.0036034 | 0.02 | 0.80 | 1.73 | 2.53 |
| PRF1 | 10:72357103-72362531 | 0.0000433 | 0.005 | 63.08 | 159.83 | 2.52 |
| NTHL1 | 16:2089815-2185899 | 0.0000118 | 0.003 | 3.54 | 8.65 | 2.50 |
| AC009065.1 | 16:2303116-2323419 | 0.0000303 | 0.004 | 22.02 | 52.63 | 2.50 |
| ACTL10 | 20:32244892-32262269 | 0.0026333 | 0.017 | 0.58 | 1.25 | 2.50 |
| AC104655.2.1 | 2:105552868-105719402 | 0.0033543 | 0.019 | 0.78 | 1.59 | 2.47 |
| IGHV5-51 | 14:107034728-107035221 | 0.0052357 | 0.025 | 4.60 | 11.92 | 2.47 |
| CTD-3232M19.2.1 | 8:145462708-145485885 | 0.0003314 | 0.007 | 1.74 | 4.14 | 2.45 |
| MTX1P1 | 1:155200765-155204116 | 0.0005517 | 0.009 | 9.34 | 21.18 | 2.45 |
| ODF3B,SCO2,TYMP | 22:50946644-50971009 | 0.0018305 | 0.014 | 324.56 | 749.84 | 2.45 |
| MIR937,SCRIB | 8:144873089-144897549 | 0.0002934 | 0.007 | 2.36 | 5.51 | 2.45 |
| RPS23P8 | X:70182813-70183140 | 0.000016 | 0.004 | 30.39 | 73.05 | 2.44 |
| AP003068.9.1 | 11:64851694-64883856 | 0.0011318 | 0.012 | 22.97 | 51.69 | 2.44 |
| SLC9A3R2 | 16:2075356-2089027 | 0.0017649 | 0.014 | 1.48 | 3.07 | 2.43 |
| SCRIB.1 | HG104_HG975_PATCH:144873089-144897549 | 0.0002267 | 0.007 | 2.27 | 5.25 | 2.43 |
| CD163,CD163L1 | 12:7499280-7656489 | 0.0030648 | 0.018 | 8.06 | 18.68 | 2.43 |
| POMC | 2:25383721-25391772 | 0.0091158 | 0.035 | 1.47 | 2.71 | 2.43 |
| ALKBH7 | 19:6372443-6377338 | 0.0119962 | 0.041 | 18.52 | 36.61 | 2.43 |
| AC145291.1 | 8:145317002-145331153 | 0.000468 | 0.008 | 1.71 | 3.92 | 2.42 |
| IER5L | 9:131937834-131972827 | 0.0012349 | 0.012 | 2.45 | 5.09 | 2.41 |
| ZNHIT2 | 11:64883874-64885170 | 0.0069853 | 0.029 | 4.44 | 9.00 | 2.41 |
| EGFL7 | 9:139543061-139567130 | 0.0009336 | 0.011 | 1.75 | 4.14 | 2.41 |
| FAM27A | 9:45727106-45728274 | 0.0129527 | 0.043 | 0.61 | 1.64 | 2.41 |
| LIME1,SLC2A4RG,ZGPAT | 20:62289162-62374858 | 0.0002935 | 0.007 | 102.84 | 238.66 | 2.40 |
| F5 | 1:169483403-169555826 | 0.0004438 | 0.008 | 13.28 | 30.78 | 2.37 |
| CLEC11A | 19:51226604-51228979 | 0.0136296 | 0.045 | 2.93 | 6.20 | 2.36 |
| PHF14 | 7:11013498-11209250 | 0.0000117 | 0.003 | 6.61 | 15.20 | 2.35 |
| A1BG | 19:58858171-58864865 | 0.0066508 | 0.029 | 1.74 | 3.56 | 2.35 |
| C9orf172 | 9:139738866-139741797 | 0.0032721 | 0.019 | 0.61 | 1.45 | 2.34 |
| DPM3 | 1:155112366-155113071 | 0.004111 | 0.022 | 28.31 | 59.82 | 2.33 |
| CHPF | 2:220238267-220586393 | 0.0013029 | 0.012 | 1.05 | 2.49 | 2.33 |
| C19orf70 | 19:5678432-5680911 | 0.0014266 | 0.013 | 17.12 | 36.65 | 2.33 |
| FLNA | X:153576891-153603006 | 0.0001371 | 0.006 | 213.51 | 472.09 | 2.32 |
| C9orf142 | 9:139886869-139888436 | 0.0005359 | 0.009 | 44.01 | 98.50 | 2.31 |
| RP11-23P13.4.1 | 15:42066631-42120134 | 0.012475 | 0.042 | 0.84 | 1.61 | 2.31 |
| MMP23B | 1:1567473-1570639 | 0.0035775 | 0.02 | 1.94 | 4.26 | 2.31 |
| RP11-661A12.7.1 | 8:144661805-144682485 | 0.0005056 | 0.008 | 20.46 | 43.40 | 2.31 |
| RP11-110I1.6.1 | 11:118914898-118927940 | 0.0045472 | 0.023 | 4.08 | 8.09 | 2.30 |
| HOXB7,HOXB8,RP11-357H14.19.1,RP11-357H14.20.1 | 17:46684589-46724385 | 0.0047891 | 0.024 | 0.51 | 1.19 | 2.30 |
| NAPRT1 | 8:144655659-144660819 | 0.0025152 | 0.017 | 38.11 | 83.58 | 2.30 |
| TNFRSF4 | 1:1146705-1149518 | 0.0019217 | 0.015 | 3.05 | 6.46 | 2.29 |
| COL6A2 | 21:47518010-47552763 | 0.0096685 | 0.036 | 5.15 | 11.01 | 2.28 |
| AVPI1 | 10:99437180-99447080 | 0.0085208 | 0.033 | 0.75 | 1.55 | 2.27 |
| DFNB31 | 9:117164359-117267730 | 0.0143068 | 0.046 | 0.63 | 1.07 | 2.26 |
| PTCRA | 6:42883726-42893898 | 0.0010578 | 0.011 | 9.05 | 19.83 | 2.26 |
| AC233264.4,IGKV1D-39,IGKV2D-38 | 2:89901291-89911512 | 0.0042716 | 0.022 | 20.35 | 51.53 | 2.26 |
| IGHG1 | 14:106202679-106209408 | 0.0010097 | 0.011 | 45.14 | 98.80 | 2.26 |
| IGHG3 | 14:106235438-106237742 | 0.0138151 | 0.045 | 6.56 | 12.34 | 2.26 |
| AC079767.4.1 | 2:208527098-208531748 | 0.0092989 | 0.035 | 2.12 | 4.18 | 2.24 |
| NDUFB7 | 19:14676891-14682886 | 0.0048248 | 0.024 | 63.40 | 127.36 | 2.24 |
| NPDC1 | 9:139933921-139940655 | 0.0089438 | 0.034 | 11.45 | 22.53 | 2.24 |
| C1orf122 | 1:38268615-38275126 | 0.0018987 | 0.014 | 20.80 | 43.88 | 2.23 |
| IRF7 | 11:612552-615999 | 0.0117427 | 0.041 | 54.58 | 115.19 | 2.23 |
| MZT2B | 2:130908980-130948302 | 0.0038635 | 0.021 | 71.17 | 142.31 | 2.22 |
| RP11-148K1.12.1 | 7:150750898-150773617 | 0.0002251 | 0.007 | 1.66 | 3.52 | 2.22 |
| AC096579.7.1,IGKC,IGKJ1 | 2:89109983-89165653 | 0.0067464 | 0.029 | 550.61 | 1306.57 | 2.22 |
| ZNF287 | 17:16454700-16472520 | 0.0002181 | 0.006 | 0.95 | 1.98 | 2.22 |
| IGKV1-39 | 2:89619382-89619904 | 0.0074498 | 0.031 | 19.44 | 47.39 | 2.21 |
| HIST2H2AA4 | 1:149822642-149823191 | 0.0003244 | 0.007 | 72.10 | 158.19 | 2.21 |
| RECQL4 | 8:145736666-145743229 | 0.0052623 | 0.025 | 1.12 | 2.12 | 2.21 |
| HSPBP1 | 19:55773591-55791751 | 0.0000133 | 0.003 | 5.59 | 12.24 | 2.21 |
| FCER2 | 19:7753643-7767032 | 0.0073208 | 0.03 | 8.02 | 16.18 | 2.19 |
| PAM16 | 16:4390251-4401373 | 0.0029803 | 0.018 | 12.93 | 27.34 | 2.19 |
| GALK1 | 17:73754017-73761307 | 0.0001675 | 0.006 | 17.26 | 36.66 | 2.18 |
| PGLS | 19:17622431-17632095 | 0.0005115 | 0.008 | 94.40 | 197.95 | 2.18 |
| TBL3 | 16:2022037-2034193 | 0.0011354 | 0.012 | 7.57 | 15.31 | 2.18 |
| DUSP2 | 2:96808904-96811179 | 0.0112273 | 0.04 | 5.40 | 11.67 | 2.16 |
| TMUB1 | 7:150778166-150780633 | 0.0013323 | 0.012 | 25.89 | 52.48 | 2.16 |
| TFPT | 19:54606035-54635140 | 0.0000636 | 0.005 | 9.64 | 20.44 | 2.16 |
| NRN1L | 16:67918780-67920275 | 0.0112807 | 0.04 | 0.76 | 1.31 | 2.16 |
| IGLV1-44 | 22:22735134-22735715 | 0.0075451 | 0.031 | 16.45 | 39.70 | 2.15 |
| RP1-20B21.4.1 | 1:17733255-17766220 | 0.0048804 | 0.024 | 14.17 | 25.33 | 2.15 |
| GZMH | 14:25064928-25126980 | 0.0000921 | 0.005 | 143.11 | 305.14 | 2.14 |
| DOC2A | 16:30003644-30034591 | 0.0000782 | 0.005 | 1.38 | 2.93 | 2.14 |
| IGKV3-15 | 2:89384672-89385283 | 0.002522 | 0.017 | 18.90 | 41.37 | 2.14 |
| RCN3 | 19:50030874-50046889 | 0.0046382 | 0.023 | 12.24 | 25.72 | 2.13 |
| SEMA6C | 1:151104160-151119104 | 0.0113764 | 0.04 | 0.65 | 1.30 | 2.13 |
| TNFRSF18 | 1:1138887-1142071 | 0.0001105 | 0.006 | 3.08 | 6.68 | 2.13 |
| NDUFS7 | 19:1383525-1396466 | 0.0042079 | 0.022 | 52.66 | 101.25 | 2.12 |
| SH2B2 | 7:101928404-101962178 | 0.0008166 | 0.01 | 9.49 | 19.71 | 2.12 |
| JUND | 19:18390562-18392432 | 0.0002726 | 0.007 | 82.43 | 169.93 | 2.12 |
| CCDC107 | 9:35658300-35681156 | 0.0003009 | 0.007 | 9.01 | 18.58 | 2.12 |
| SCN1B | 19:35521591-35531352 | 0.0028116 | 0.018 | 2.14 | 4.37 | 2.12 |
| CORO1B,PTPRCAP | 11:67202980-67211292 | 0.0007276 | 0.01 | 166.52 | 337.39 | 2.11 |
| HLA-DRB1 | 6:32546545-32557625 | 0.0002128 | 0.006 | 49.40 | 101.33 | 2.11 |
| AC004258.1,C19orf24 | 19:1275437-1279227 | 0.001605 | 0.013 | 31.00 | 61.17 | 2.11 |
| C17orf90 | 17:79632066-79633618 | 0.007264 | 0.03 | 16.34 | 31.47 | 2.11 |
| PDZD4 | X:153067620-153096020 | 0.0005118 | 0.008 | 5.29 | 11.57 | 2.11 |
| MBD3 | 19:1576677-1592710 | 0.0006785 | 0.009 | 15.15 | 30.20 | 2.11 |
| SART1 | 11:65729159-65747299 | 0.0012655 | 0.012 | 30.86 | 60.79 | 2.10 |
| PCP2 | 19:7694670-7698570 | 0.0032049 | 0.019 | 1.27 | 2.63 | 2.10 |
| VCAN-AS1 | 5:82767283-82878122 | 0.0024992 | 0.017 | 35.44 | 71.02 | 2.10 |
| NBAS | 2:15307031-15701454 | 0.0001536 | 0.006 | 4.47 | 9.19 | 2.10 |
| MIPEP | 13:24304327-24476794 | 0.0002973 | 0.007 | 1.32 | 2.74 | 2.10 |
| AC008073.6.1 | 2:24299395-24397874 | 0.0037247 | 0.021 | 5.40 | 10.73 | 2.10 |
| RNASEH2C | 11:65479466-65488418 | 0.0018507 | 0.014 | 20.03 | 40.39 | 2.10 |
| AURKAIP1 | 1:1309109-1310875 | 0.0002368 | 0.007 | 73.27 | 149.90 | 2.10 |
| RTN4R | 22:20228937-20270769 | 0.0051317 | 0.024 | 0.56 | 1.17 | 2.08 |
| IGLV1-40 | 22:22764097-22764614 | 0.0006 | 0.009 | 23.37 | 52.78 | 2.08 |
| CTSW | 11:65647279-65656010 | 0.0000426 | 0.005 | 60.22 | 127.64 | 2.08 |
| MRPS34 | 16:1821890-1823156 | 0.000027 | 0.004 | 34.21 | 70.04 | 2.08 |
| RP11-6I2.3.1 | 8:74964574-75012088 | 0.0028373 | 0.018 | 1.66 | 3.17 | 2.08 |
| C6orf226 | 6:42858004-42858554 | 0.0148973 | 0.048 | 8.27 | 14.98 | 2.07 |
| AP001062.8.1,C21orf2 | 21:45748826-45760353 | 0.000456 | 0.008 | 5.67 | 11.71 | 2.07 |
| HLA-DPB1 | 6:33032345-33054978 | 0.0033226 | 0.019 | 30.34 | 57.41 | 2.07 |
| LA16c-366D1.3.1 | 16:577716-604636 | 0.0007715 | 0.01 | 2.81 | 5.45 | 2.06 |
| CD79A | 19:42381189-42385439 | 0.0019395 | 0.015 | 35.73 | 69.79 | 2.06 |
| IGHM | 14:106320348-106322323 | 0.0003539 | 0.007 | 110.29 | 208.23 | 2.06 |
| MMP17 | 12:132312937-132336328 | 0.0002278 | 0.007 | 5.53 | 10.53 | 2.06 |
| XCL2 | 1:168510002-168513235 | 0.0005315 | 0.009 | 9.59 | 19.32 | 2.06 |
| CST3 | 20:23608533-23619110 | 0.0023887 | 0.016 | 634.61 | 1221.19 | 2.05 |
| ZNF524 | 19:56111729-56114504 | 0.0001256 | 0.006 | 23.23 | 46.64 | 2.05 |
| SSBP4 | 19:18530220-18549111 | 0.0000112 | 0.003 | 26.36 | 53.54 | 2.05 |
| AC079250.1.1 | 2:47917854-47918385 | 0.0007041 | 0.009 | 1.26 | 2.40 | 2.04 |
| JUP.1 | HG185_PATCH:39910855-39943183 | 0.0092846 | 0.035 | 5.02 | 8.52 | 2.04 |
| NME3 | 16:1756183-1821731 | 0.0045832 | 0.023 | 24.21 | 45.21 | 2.04 |
| C6orf108 | 6:43193366-43197222 | 0.0006039 | 0.009 | 11.30 | 21.58 | 2.04 |
| PRR24 | 19:47777709-47778964 | 0.0019303 | 0.015 | 9.94 | 19.63 | 2.04 |
| PMFBP1 | 16:72078187-72210777 | 0.0107594 | 0.039 | 4.20 | 7.29 | 2.04 |
| LENG9 | 19:54955990-54974894 | 0.0027249 | 0.017 | 3.77 | 6.83 | 2.03 |
| DDX10 | 11:108535751-108811657 | 0.000898 | 0.01 | 2.91 | 5.64 | 2.03 |
| FANCG | 9:35073831-35080013 | 0.000686 | 0.009 | 4.25 | 8.07 | 2.03 |
| PLEKHF1 | 19:30156326-30166376 | 0.0002406 | 0.007 | 8.97 | 18.52 | 2.03 |
| INTS1 | 7:1509912-1545489 | 0.0006499 | 0.009 | 14.89 | 29.11 | 2.03 |
| COL18A1 | 21:46825051-46964325 | 0.0000312 | 0.004 | 24.06 | 51.35 | 2.03 |
| TMEM161A | 19:19229977-19249275 | 0.0005531 | 0.009 | 3.77 | 7.24 | 2.02 |
| PLCH2 | 1:2398897-2436969 | 0.0001107 | 0.006 | 1.55 | 3.01 | 2.02 |
| BOP1.1 | HG243_PATCH:145317004-145346273 | 0.0017235 | 0.014 | 2.09 | 3.85 | 2.02 |
| TP53I13 | 17:27895738-27900172 | 0.0009957 | 0.011 | 10.90 | 21.45 | 2.02 |
| PUSL1 | 1:1227755-1260071 | 0.0004826 | 0.008 | 4.98 | 9.75 | 2.02 |
| RP3-412A9.11.1 | 22:30279143-31530682 | 0.003354 | 0.019 | 9.14 | 15.96 | 2.02 |
| ATRIP,TREX1 | 3:48488113-48509044 | 0.0016135 | 0.013 | 39.66 | 77.64 | 2.01 |
| MATK | 19:3777967-3801810 | 0.000299 | 0.007 | 18.48 | 39.24 | 2.01 |
| C11orf83 | 11:62414319-62441159 | 0.0012692 | 0.012 | 3.02 | 5.75 | 2.01 |
| EIF4EBP1 | 8:37887858-37917883 | 0.0020796 | 0.015 | 21.83 | 41.55 | 2.01 |
| PRSS30P | 16:2889568-2892745 | 0.0058287 | 0.026 | 2.00 | 4.09 | 2.00 |
| ZMYND10 | 3:50378536-50384283 | 0.003203 | 0.019 | 1.07 | 2.01 | 2.00 |
| ABCA7 | 19:1040101-1065571 | 0.0003613 | 0.007 | 68.16 | 139.84 | 2.00 |
| IGHV3-21 | 14:106691672-106692203 | 0.0067303 | 0.029 | 5.85 | 11.87 | 2.00 |
| ABHD16B | 20:62492565-62494341 | 0.0025871 | 0.017 | 3.67 | 6.78 | 2.00 |
| AL592284.1 | 1:144275917-144521970 | 0.0005697 | 0.009 | 10.42 | 19.97 | 2.00 |
| HLA-DQB1,XXbac-BPG254F23.6.2 | HSCHR6_MHC_DBB:32602483-32612425 | 0.0087046 | 0.034 | 12.98 | 20.02 | 1.99 |
| CFD | 19:859664-863606 | 0.0023068 | 0.016 | 150.08 | 261.83 | 1.99 |
| RP3-508I15.18.1,RP3-508I15.19.1 | 22:39101727-39190203 | 0.0000177 | 0.004 | 48.81 | 96.98 | 1.99 |
| MZF1 | 19:59073284-59111168 | 0.0041351 | 0.022 | 3.81 | 7.18 | 1.99 |
| FAM207A | 21:46359924-46396904 | 0.0004359 | 0.008 | 6.73 | 12.97 | 1.99 |
| TSPO | 22:43547519-43559248 | 0.0024376 | 0.016 | 369.52 | 687.74 | 1.98 |
| PCCA | 13:100741268-101182686 | 0.001617 | 0.013 | 3.07 | 5.83 | 1.98 |
| ITGA2B | 17:42449549-42466873 | 0.0035213 | 0.02 | 10.26 | 19.64 | 1.98 |
| CHCHD6 | 3:126423062-126679249 | 0.0027473 | 0.017 | 2.34 | 4.09 | 1.98 |
| SELP | 1:169558036-169599380 | 0.0026492 | 0.017 | 3.28 | 6.17 | 1.98 |
| RASSF7 | 11:537526-564021 | 0.0010663 | 0.011 | 10.23 | 19.23 | 1.97 |
| GP1BB,SEPT5 | 22:19696896-19712295 | 0.0049839 | 0.024 | 71.88 | 131.53 | 1.97 |
| COPZ2 | 17:46103534-46115139 | 0.0066819 | 0.029 | 1.72 | 3.17 | 1.97 |
| ZNF683 | 1:26688124-26701013 | 0.0024107 | 0.016 | 9.53 | 16.95 | 1.97 |
| MSC | 8:72740401-73030628 | 0.0134822 | 0.045 | 3.00 | 4.78 | 1.97 |
| H2AFX | 11:118964563-118966177 | 0.0000769 | 0.005 | 11.13 | 21.55 | 1.96 |
| DGCR6 | 22:18893540-18924066 | 0.0041688 | 0.022 | 2.03 | 3.52 | 1.96 |
| CLDND2 | 19:51870351-51872257 | 0.0009163 | 0.011 | 3.11 | 6.70 | 1.96 |
| RP11-802E16.3.1,TCIRG1 | 11:67806482-67888736 | 0.0006582 | 0.009 | 236.91 | 457.83 | 1.96 |
| EPRS | 1:220141942-220220000 | 0.0009193 | 0.011 | 11.54 | 21.52 | 1.96 |
| MED25 | 19:50321535-50342036 | 0.0018979 | 0.014 | 48.33 | 93.13 | 1.96 |
| SLC1A7 | 1:53552854-53608289 | 0.0114161 | 0.04 | 0.97 | 1.51 | 1.96 |
| RP11-229P13.20.1,RP11-229P13.21.1 | 9:139931491-139933342 | 0.0127257 | 0.043 | 8.48 | 15.89 | 1.96 |
| MFSD10 | 4:2932287-2936586 | 0.0014733 | 0.013 | 37.05 | 68.73 | 1.96 |
| AZI1 | 17:79163392-79196799 | 0.0004447 | 0.008 | 1.04 | 2.01 | 1.95 |
| ALDH16A1 | 19:49956472-49974304 | 0.0004879 | 0.008 | 9.02 | 16.53 | 1.95 |
| RNASEH2A | 19:12917427-12924462 | 0.0015022 | 0.013 | 6.07 | 10.74 | 1.94 |
| C9orf16 | 9:130922538-130926207 | 0.0014632 | 0.013 | 76.29 | 142.41 | 1.94 |
| LAG3 | 12:6881677-6887621 | 0.0000109 | 0.003 | 5.62 | 11.32 | 1.94 |
| CD320 | 19:8367010-8373240 | 0.0005814 | 0.009 | 11.30 | 21.71 | 1.94 |
| HCG25 | 6:33217310-33244287 | 0.0003465 | 0.007 | 3.33 | 6.51 | 1.94 |
| NDUFS8 | 11:67798083-67804111 | 0.0041883 | 0.022 | 41.56 | 74.40 | 1.94 |
| GIPC1 | 19:14588571-14606944 | 9.53E-06 | 0.003 | 6.54 | 12.65 | 1.94 |
| TRBV13 | HG7_PATCH:142277447-142277943 | 0.0038185 | 0.021 | 4.01 | 7.91 | 1.94 |
| BCRP2 | 22:21457304-21482352 | 0.0137359 | 0.045 | 0.62 | 1.15 | 1.94 |
| JAG2,NUDT14,RP11-44N21.4.1 | 14:105607317-105647660 | 0.0117995 | 0.041 | 7.61 | 12.48 | 1.94 |
| VAC14 | 16:70721341-70835064 | 0.0011332 | 0.012 | 9.69 | 18.21 | 1.94 |
| RP11-244F12.2.1 | 15:63334830-63364114 | 0.0035915 | 0.02 | 2.72 | 5.48 | 1.93 |
| MACROD1 | 11:63742078-63933578 | 0.0122422 | 0.042 | 1.64 | 3.04 | 1.93 |
| SURF2 | 9:136218609-136228045 | 0.0001334 | 0.006 | 4.08 | 7.82 | 1.93 |
| MAMDC4,PHPT1 | 9:139743175-139755249 | 0.0055938 | 0.026 | 21.16 | 37.35 | 1.93 |
| IFI27L2 | 14:94594115-94596590 | 0.0069049 | 0.029 | 44.15 | 80.16 | 1.93 |
| NFKBIB | 19:39390603-39399533 | 0.0008911 | 0.01 | 14.03 | 25.61 | 1.93 |
| RGS9 | 17:63133455-63223819 | 0.0002843 | 0.007 | 1.27 | 2.41 | 1.93 |
| IGHD | 14:106303098-106312010 | 0.0063006 | 0.028 | 13.32 | 23.27 | 1.93 |
| HEXDC | 17:80347085-80400515 | 0.0013614 | 0.013 | 11.70 | 21.87 | 1.93 |
| UBE2S | 19:55896712-55919325 | 0.0008712 | 0.01 | 15.82 | 29.44 | 1.92 |
| POLR2L | 11:818901-842545 | 0.0081299 | 0.032 | 55.05 | 96.90 | 1.92 |
| SPTAN1 | 9:131314865-131419066 | 0.0004138 | 0.008 | 12.24 | 23.09 | 1.92 |
| MRPL41 | 9:140445650-140447007 | 0.0005321 | 0.009 | 22.56 | 42.68 | 1.92 |
| AL590822.1,C1orf86,RP11-181G12.2.1,RP11-181G12.4.1,RP11-181G12.5.1 | 1:1981908-2152175 | 0.0011933 | 0.012 | 36.76 | 66.61 | 1.92 |
| CTU1 | 19:51600862-51611647 | 0.0017316 | 0.014 | 3.22 | 5.99 | 1.92 |
| GLTPD1 | 1:1260135-1264277 | 0.0006372 | 0.009 | 10.28 | 18.94 | 1.91 |
| CD8B | 2:87042461-87092827 | 0.0033681 | 0.019 | 50.03 | 99.73 | 1.91 |
| AKR1C3 | 10:5077548-5149878 | 0.0013795 | 0.013 | 4.13 | 7.78 | 1.91 |
| ATP5D | 19:1241748-1244823 | 0.0031286 | 0.019 | 68.92 | 122.53 | 1.91 |
| ASPSCR1 | 17:79935425-79975280 | 0.0039542 | 0.021 | 7.43 | 13.20 | 1.91 |
| ENDOG | 9:131580752-131592100 | 0.0000702 | 0.005 | 2.71 | 5.10 | 1.91 |
| ARMC5 | 16:31468031-31478487 | 0.0088083 | 0.034 | 2.41 | 4.28 | 1.91 |
| PODXL2 | 3:127348023-127391652 | 0.0009925 | 0.011 | 0.56 | 1.07 | 1.91 |
| IGHV5-78 | 14:107259337-107259632 | 0.0086714 | 0.034 | 4.12 | 7.03 | 1.91 |
| HSPA4 | 5:132387653-132442141 | 0.0000132 | 0.003 | 13.92 | 26.33 | 1.91 |
| POLD1 | 19:50887592-50921270 | 0.0000543 | 0.005 | 2.62 | 4.93 | 1.91 |
| CLDN5 | 22:19510546-19515068 | 0.0003453 | 0.007 | 2.82 | 5.62 | 1.91 |
| MFSD3 | 8:145734456-145736569 | 0.0008957 | 0.01 | 3.08 | 5.68 | 1.90 |
| IGFALS,RP11-31I10.4.1,SPSB3 | 16:1823207-1844972 | 0.0068768 | 0.029 | 61.58 | 111.49 | 1.90 |
| CFP | X:47483611-47489704 | 0.0002322 | 0.007 | 265.44 | 500.03 | 1.90 |
| RPP21,TRIM39 | HSCHR6_MHC_APD:30266816-30322899 | 0.004716 | 0.023 | 2.98 | 5.70 | 1.90 |
| PNOC | 8:28107579-28200872 | 0.0032368 | 0.019 | 3.42 | 5.74 | 1.90 |
| RPL13 | 16:89627064-89630950 | 0.0010687 | 0.011 | 2310.99 | 4252.77 | 1.90 |
| CARD9,DNLZ | 9:139221931-139268133 | 0.0004715 | 0.008 | 14.08 | 25.70 | 1.90 |
| PLA2G4C | 19:48551099-48614109 | 0.014411 | 0.047 | 0.88 | 1.29 | 1.89 |
| CCDC17 | 1:46085715-46089729 | 0.0023159 | 0.016 | 1.66 | 3.06 | 1.89 |
| RBM42 | 19:36119979-36128586 | 0.0010927 | 0.011 | 30.31 | 54.85 | 1.89 |
| EIF3C | 16:28671016-28752102 | 0.0000485 | 0.005 | 37.08 | 69.23 | 1.89 |
| WDR34 | 9:131314865-131419066 | 0.0011613 | 0.012 | 2.20 | 4.03 | 1.88 |
| RP4-728D4.2.1 | 1:36035413-36060929 | 0.0095662 | 0.036 | 2.99 | 5.06 | 1.88 |
| ZNF444 | 19:56652555-56672261 | 9.48E-06 | 0.003 | 7.09 | 13.26 | 1.88 |
| ADAM15,DCST1 | 1:155006299-155035252 | 0.0077551 | 0.031 | 25.95 | 46.20 | 1.88 |
| SIGIRR | 11:405715-417455 | 0.0002862 | 0.007 | 65.64 | 121.51 | 1.88 |
| ZNF787 | 19:56598731-56632649 | 0.0001872 | 0.006 | 24.37 | 45.29 | 1.88 |
| PPP1R14A | 19:38741876-38747231 | 0.0002127 | 0.006 | 1.94 | 3.78 | 1.88 |
| PIN1 | 19:9945998-9960365 | 0.0046035 | 0.023 | 32.65 | 57.14 | 1.88 |
| TPPP3,U1 | 16:67423711-67427438 | 0.0014033 | 0.013 | 7.08 | 12.93 | 1.88 |
| RP11-108M9.5.1 | 1:17215032-17216144 | 0.0015677 | 0.013 | 1.50 | 2.69 | 1.88 |
| CTNNBL1 | 20:36322407-36500531 | 0.0002741 | 0.007 | 19.08 | 34.76 | 1.87 |
| FAM41C | 1:803450-812283 | 0.012417 | 0.042 | 3.59 | 4.93 | 1.87 |
| LRCH4,SAP25 | 7:100169854-100205798 | 0.0011069 | 0.011 | 147.43 | 269.45 | 1.87 |
| LINC00173 | 12:116971226-116974323 | 0.0159076 | 0.05 | 16.43 | 29.58 | 1.87 |
| NKG7 | 19:51874873-51875960 | 0.0000657 | 0.005 | 507.11 | 956.82 | 1.87 |
| RHPN1 | 8:144451056-144466390 | 0.0004378 | 0.008 | 1.18 | 2.22 | 1.87 |
| WDR18 | 19:984327-994569 | 0.002615 | 0.017 | 8.17 | 14.19 | 1.87 |
| ZC3H3 | 8:144519824-144623623 | 0.0013885 | 0.013 | 9.08 | 16.41 | 1.87 |
| HK1 | 10:71029739-71161638 | 0.0147383 | 0.047 | 40.34 | 67.89 | 1.87 |
| BFSP1 | 20:17474549-17549865 | 0.0080003 | 0.032 | 0.78 | 1.36 | 1.86 |
| LRFN1 | 19:39797207-39805976 | 0.0003749 | 0.007 | 10.54 | 19.38 | 1.86 |
| AC092535.1,SPON2 | 4:1160719-1202750 | 0.001823 | 0.014 | 33.54 | 64.23 | 1.86 |
| SPHK1 | 17:74307013-74383941 | 0.0151101 | 0.048 | 3.85 | 6.63 | 1.86 |
| DOHH | 19:3490819-3500938 | 0.0003121 | 0.007 | 3.36 | 6.14 | 1.86 |
| AC008984.5.1,LILRA3 | 19:54799853-54809952 | 0.0048131 | 0.024 | 37.14 | 59.89 | 1.86 |
| AC107081.5.1 | 2:62095223-62374382 | 0.0000991 | 0.005 | 3.84 | 6.85 | 1.86 |
| KIAA1429 | 8:95499920-95565757 | 0.0006236 | 0.009 | 11.60 | 20.97 | 1.85 |
| SLC22A18 | 11:2909009-2946476 | 0.0160835 | 0.05 | 22.86 | 38.49 | 1.85 |
| NOC4L | 12:132628992-132637013 | 0.0005308 | 0.009 | 7.60 | 13.43 | 1.85 |
| SOLH | 16:577716-604636 | 0.000126 | 0.006 | 9.01 | 16.69 | 1.85 |
| C16orf42 | 16:1383601-1401912 | 0.0007558 | 0.01 | 30.66 | 54.81 | 1.85 |
| ANO9 | 11:417932-442011 | 0.0098916 | 0.037 | 7.34 | 12.23 | 1.85 |
| MLC1 | 22:50497819-50524331 | 0.002106 | 0.015 | 2.83 | 5.02 | 1.85 |
| FLNB | 3:57994126-58157982 | 0.0029825 | 0.018 | 2.29 | 4.16 | 1.84 |
| CP | 3:148847370-148939842 | 0.0006851 | 0.009 | 2.07 | 3.82 | 1.84 |
| TTF1 | 9:135251007-135282213 | 0.0020677 | 0.015 | 5.86 | 10.09 | 1.84 |
| CISD3 | 17:36886509-36904558 | 0.0016798 | 0.014 | 4.82 | 8.60 | 1.84 |
| RP11-304M2.2.1 | 11:127114-207428 | 0.001995 | 0.015 | 107.25 | 196.75 | 1.84 |
| PARP10.1 | HG104_HG975_PATCH:145059322-145129202 | 0.001417 | 0.013 | 14.87 | 26.69 | 1.84 |
| EXOSC4 | 8:145132904-145135550 | 0.0081737 | 0.032 | 4.54 | 7.62 | 1.84 |
| EXOSC4.1 | HG104_HG975_PATCH:145160173-145162819 | 0.0081737 | 0.032 | 4.54 | 7.62 | 1.84 |
| MAP1S | 19:17830290-17845322 | 0.0019163 | 0.015 | 11.51 | 20.07 | 1.84 |
| C1orf35 | 1:228288426-228293112 | 0.0069744 | 0.029 | 6.69 | 11.37 | 1.84 |
| KLRF1 | 12:9980076-9997606 | 0.0003994 | 0.008 | 19.41 | 35.44 | 1.84 |
| DDRGK1 | 20:3170995-3185331 | 0.0000611 | 0.005 | 17.53 | 31.97 | 1.84 |
| KPTN | 19:47978399-47987521 | 0.0027421 | 0.017 | 3.01 | 5.37 | 1.84 |
| CCDC61 | 19:46498338-46521874 | 0.0115297 | 0.04 | 4.82 | 8.13 | 1.84 |
| RP11-206L10.1.1 | 1:661610-663527 | 0.0036349 | 0.02 | 305.78 | 551.56 | 1.83 |
| PIEZO1 | 16:88772870-88851619 | 0.0078331 | 0.032 | 22.59 | 38.86 | 1.83 |
| GAS2L1 | 22:29702571-29708774 | 0.0034416 | 0.02 | 6.41 | 11.63 | 1.83 |
| S1PR4 | 19:3178735-3180329 | 0.0020305 | 0.015 | 227.68 | 410.26 | 1.83 |
| GAK | 4:843063-926161 | 0.0003344 | 0.007 | 26.97 | 48.51 | 1.83 |
| DGCR6L | 22:20301798-20307603 | 0.0011275 | 0.012 | 19.63 | 34.56 | 1.83 |
| ASCL2 | 11:2289724-2292182 | 0.0000346 | 0.005 | 8.40 | 14.99 | 1.83 |
| ECI1 | 16:2289395-2302301 | 0.0027299 | 0.017 | 17.31 | 30.24 | 1.83 |
| FDXR | 17:72858619-72869156 | 0.0001732 | 0.006 | 1.92 | 3.48 | 1.82 |
| CST7 | 20:24929865-24940564 | 0.004921 | 0.024 | 226.06 | 375.82 | 1.82 |
| CSF1R | 5:149432853-149492935 | 0.0059089 | 0.027 | 30.72 | 54.02 | 1.82 |
| FAM108A1 | 19:1876808-1885500 | 0.000637 | 0.009 | 77.78 | 138.98 | 1.82 |
| NMB | 15:85198359-85201794 | 0.0008815 | 0.01 | 1.33 | 2.21 | 1.82 |
| HEXIM2 | 17:43238270-43247406 | 0.0013008 | 0.012 | 3.73 | 6.50 | 1.82 |
| BBC3 | 19:47724078-47736023 | 0.0017229 | 0.014 | 14.69 | 26.44 | 1.82 |
| MED16 | 19:867963-893218 | 0.0005378 | 0.009 | 15.38 | 26.92 | 1.82 |
| SDF2L1 | 22:21996549-21998587 | 0.0008101 | 0.01 | 19.63 | 34.92 | 1.82 |
| PARP10 | 8:145051320-145101933 | 0.0011939 | 0.012 | 15.12 | 26.85 | 1.82 |
| YIF1A | 11:66036003-66056641 | 0.0081991 | 0.032 | 18.94 | 32.15 | 1.82 |
| DPP7 | 9:140004993-140009629 | 0.0008417 | 0.01 | 65.28 | 114.23 | 1.82 |
| BLK | 8:11351509-11422113 | 0.0006022 | 0.009 | 6.61 | 11.82 | 1.81 |
| FASTK | 7:150773678-150777953 | 0.0005615 | 0.009 | 23.93 | 42.34 | 1.81 |
| STRA13 | 17:79976578-79980794 | 0.0137711 | 0.045 | 10.92 | 18.29 | 1.81 |
| FAM100A | 16:4656110-4665028 | 0.0035191 | 0.02 | 19.30 | 33.18 | 1.81 |
| RRAS | 19:50138551-50143400 | 0.0007563 | 0.01 | 21.65 | 38.18 | 1.81 |
| C10orf125 | 10:135168657-135171529 | 0.0136386 | 0.045 | 11.88 | 19.65 | 1.81 |
| LTBP4 | 19:41099071-41135725 | 0.0005112 | 0.008 | 7.74 | 14.07 | 1.81 |
| FBXO2 | 1:11708423-11723384 | 0.0018699 | 0.014 | 1.71 | 2.94 | 1.81 |
| IGHV1-8 | 14:106539078-106539577 | 0.0129802 | 0.044 | 4.59 | 8.32 | 1.81 |
| AL353791.1.1 | 9:40028619-40032417 | 0.003984 | 0.021 | 2.69 | 4.90 | 1.81 |
| DUS3L | 19:5785154-5791249 | 0.0061514 | 0.027 | 5.50 | 9.35 | 1.81 |
| GPBAR1 | 2:219124218-219128582 | 0.0116931 | 0.041 | 24.06 | 39.13 | 1.80 |
| ANAPC11 | 17:79849598-79858363 | 0.0136521 | 0.045 | 29.88 | 48.76 | 1.80 |
| TBXA2R | 19:3594503-3606831 | 0.0089562 | 0.034 | 0.85 | 1.40 | 1.80 |
| TRAPPC6A | 19:45666186-45681485 | 0.0038225 | 0.021 | 22.49 | 38.38 | 1.80 |
| DDX24 | 14:94517265-94547591 | 2.19E-06 | 0.003 | 23.45 | 41.99 | 1.80 |
| C16orf86 | 16:67700718-67702660 | 0.0006782 | 0.009 | 1.98 | 3.45 | 1.80 |
| RP11-1100L3.7.1 | 12:52463029-52502043 | 0.0008699 | 0.01 | 11.72 | 20.46 | 1.80 |
| RRBP1 | 20:17594322-17662940 | 0.0004466 | 0.008 | 5.11 | 8.90 | 1.80 |
| FBXW5 | 9:139834886-139839148 | 0.0001622 | 0.006 | 49.49 | 87.90 | 1.80 |
| MAP3K6 | 1:27681669-27693383 | 0.0063022 | 0.028 | 3.53 | 6.11 | 1.80 |
| NT5C | 17:73106081-73127877 | 0.0018949 | 0.014 | 17.02 | 29.87 | 1.80 |
| MDM2,RP11-611O2.3.1 | 12:69201955-69365350 | 0.0013089 | 0.012 | 33.25 | 59.00 | 1.80 |
| CHST12 | 7:2443222-2474242 | 0.0002225 | 0.007 | 9.03 | 15.88 | 1.79 |
| TLN1 | 9:35696944-35758572 | 0.0003274 | 0.007 | 98.20 | 175.35 | 1.79 |
| KIAA0415 | 7:4815252-4833943 | 0.001369 | 0.013 | 20.03 | 35.13 | 1.79 |
| RP11-277L2.3.1 | 1:149575481-149651107 | 0.0123184 | 0.042 | 10.93 | 17.64 | 1.79 |
| H1FX | 3:129033614-129045068 | 0.0065249 | 0.028 | 61.59 | 100.13 | 1.79 |
| YIF1B | 19:38794199-38806606 | 0.0006436 | 0.009 | 12.99 | 22.67 | 1.79 |
| CRIP2 | 14:105939298-105946499 | 0.0030432 | 0.018 | 4.93 | 8.07 | 1.79 |
| MRPL12 | 17:79670403-79688041 | 0.0007386 | 0.01 | 17.12 | 29.92 | 1.78 |
| HOMER3 | 19:19040009-19052041 | 0.0083539 | 0.033 | 13.93 | 23.43 | 1.78 |
| CHD4 | 12:6679248-6716642 | 0.0000511 | 0.005 | 28.41 | 49.62 | 1.78 |
| LRRC45 | 17:79981279-79989024 | 0.0006189 | 0.009 | 2.66 | 4.69 | 1.78 |
| POLRMT | 19:617222-633568 | 0.002175 | 0.015 | 5.06 | 8.78 | 1.78 |
| TMEM200B | 1:29213602-29450447 | 0.0023351 | 0.016 | 8.44 | 14.71 | 1.78 |
| ISYNA1 | 19:18530220-18549111 | 0.0000241 | 0.004 | 3.41 | 6.11 | 1.78 |
| SLC15A3 | 11:60680637-60720002 | 0.0002941 | 0.007 | 71.60 | 125.81 | 1.78 |
| SH2D2A | 1:156776034-156851642 | 0.0003181 | 0.007 | 11.74 | 21.01 | 1.78 |
| HCFC1R1 | 16:3072625-3077756 | 0.0055056 | 0.026 | 21.70 | 36.64 | 1.78 |
| HSD17B4 | 5:118788137-118878028 | 0.0034206 | 0.019 | 34.03 | 58.01 | 1.78 |
| HSD17B1 | 17:40703983-40707857 | 0.0014008 | 0.013 | 3.03 | 5.12 | 1.78 |
| GSDMD | 8:144635376-144645232 | 0.0001821 | 0.006 | 59.86 | 103.97 | 1.78 |
| HIST2H2AA3 | 1:149813504-149814478 | 0.0028204 | 0.018 | 23.92 | 41.78 | 1.78 |
| UBXN11 | 1:26605666-26647014 | 0.0062533 | 0.028 | 49.56 | 85.86 | 1.77 |
| INF2 | 14:105155942-105185942 | 0.0034099 | 0.019 | 6.96 | 11.56 | 1.77 |
| CC2D1A | 19:14016955-14041693 | 0.0000545 | 0.005 | 4.13 | 7.20 | 1.77 |
| POR | 7:75528517-75623977 | 0.0000394 | 0.005 | 22.84 | 40.55 | 1.77 |
| TSSC4 | 11:2421717-2425106 | 0.0023325 | 0.016 | 22.91 | 39.37 | 1.77 |
| PSMD1 | 2:231921577-232037541 | 0.0001259 | 0.006 | 17.36 | 30.26 | 1.77 |
| RP4-734P14.4.1,SNRPB,ZNF343 | 20:2442279-2505348 | 7.18E-06 | 0.003 | 87.28 | 153.24 | 1.77 |
| RP11-114H24.6.1 | 15:78268383-78370066 | 0.0017479 | 0.014 | 5.57 | 9.72 | 1.77 |
| ANXA6 | 5:150480272-150537443 | 2.47E-06 | 0.003 | 79.10 | 140.05 | 1.77 |
| IGHV1-2 | 14:106452670-106453170 | 0.0153609 | 0.048 | 4.47 | 9.25 | 1.77 |
| BCL7C | 16:30844946-30967832 | 0.0003268 | 0.007 | 20.83 | 36.32 | 1.77 |
| DMPK | 19:46272977-46285815 | 0.0026816 | 0.017 | 3.59 | 6.12 | 1.77 |
| RABEP2 | 16:28889725-28950667 | 0.000019 | 0.004 | 6.52 | 11.54 | 1.77 |
| DCXR | 17:79993756-79995573 | 0.0007325 | 0.01 | 32.65 | 55.77 | 1.76 |
| TRIM28 | 19:59055835-59062082 | 0.000043 | 0.005 | 33.49 | 58.63 | 1.76 |
| C16orf13 | 16:684428-686358 | 0.0028214 | 0.018 | 31.58 | 53.06 | 1.76 |
| ATAD3A | 1:1447530-1470067 | 0.0018105 | 0.014 | 3.90 | 6.43 | 1.76 |
| THAP7 | 22:21353392-21368581 | 0.0001219 | 0.006 | 11.16 | 19.45 | 1.76 |
| U2AF1L4 | 19:36233429-36236336 | 0.0135044 | 0.045 | 14.72 | 23.80 | 1.76 |
| HIC1 | 17:1958392-1962980 | 0.0135742 | 0.045 | 0.90 | 1.49 | 1.76 |
| NAT14 | 19:55996593-55998935 | 0.0031308 | 0.019 | 1.94 | 3.16 | 1.76 |
| OCEL1 | 19:17337054-17340027 | 2.04E-06 | 0.003 | 11.93 | 20.94 | 1.76 |
| MAD1L1 | 7:1855382-2272878 | 0.0000607 | 0.005 | 22.29 | 38.70 | 1.76 |
| RP11-536K7.3.1 | 10:5903688-5987863 | 0.000679 | 0.009 | 10.27 | 17.46 | 1.76 |
| CCDC88B | 11:64107689-64125006 | 0.0002864 | 0.007 | 83.43 | 146.84 | 1.76 |
| CTD-2515O10.1.1,HIRIP3 | 16:30003644-30034591 | 0.0144068 | 0.047 | 3.97 | 6.57 | 1.76 |
| CAPN12 | 19:39138309-39235114 | 0.0004481 | 0.008 | 3.90 | 6.67 | 1.76 |
| GNLY | 2:85912297-85925977 | 0.000431 | 0.008 | 457.26 | 800.41 | 1.76 |
| TUBB4A | 19:6494330-6502330 | 0.0017124 | 0.014 | 0.57 | 1.04 | 1.75 |
| ZNF446 | 19:58987794-59000899 | 0.0038153 | 0.021 | 2.73 | 4.45 | 1.75 |
| TMIGD2 | 19:4292236-4302428 | 0.0022011 | 0.016 | 4.79 | 8.13 | 1.75 |
| TNNT3 | 11:1940791-1959936 | 0.0002376 | 0.007 | 2.21 | 3.86 | 1.75 |
| G6PC3 | 17:42148097-42153711 | 0.0036398 | 0.02 | 10.29 | 16.94 | 1.75 |
| MRPL28 | 16:416926-420568 | 0.001488 | 0.013 | 35.90 | 60.78 | 1.75 |
| HIST2H2AC | 1:149858485-149872351 | 0.0143196 | 0.046 | 2.76 | 4.37 | 1.75 |
| KIAA0020 | 9:2717238-2844241 | 0.0002358 | 0.007 | 5.52 | 9.60 | 1.75 |
| MZT2A | 2:132222472-132250316 | 0.0079097 | 0.032 | 31.23 | 50.22 | 1.75 |
| PSMB10 | 16:67968404-67970990 | 0.0043067 | 0.022 | 179.62 | 297.33 | 1.75 |
| CD79B | 17:62006099-62009714 | 0.000963 | 0.011 | 35.97 | 60.20 | 1.74 |
| FAM125B | 9:129089127-129269320 | 0.0005855 | 0.009 | 1.94 | 3.32 | 1.74 |
| ARHGAP39,C8orf82,LRRC24 | 8:145743375-145911194 | 0.00109 | 0.011 | 7.76 | 12.94 | 1.74 |
| B3GAT3 | 11:62382767-62389647 | 0.0012 | 0.012 | 19.78 | 33.62 | 1.74 |
| CISD1 | 10:60028817-60049346 | 0.0037015 | 0.021 | 6.85 | 11.06 | 1.74 |
| HLA-DRB3 | HSCHR6_MHC_COX:32411910-32425028 | 3.65E-06 | 0.003 | 19.31 | 34.34 | 1.74 |
| SLC39A3 | 19:2732201-2740074 | 0.0001702 | 0.006 | 12.06 | 20.81 | 1.74 |
| LTB | 6:31548301-31550299 | 0.0037331 | 0.021 | 64.64 | 107.92 | 1.74 |
| CHI3L2 | 1:111729795-111786062 | 0.0024716 | 0.017 | 6.12 | 10.20 | 1.74 |
| GPSM1 | 9:139221931-139268133 | 0.0115219 | 0.04 | 0.87 | 1.34 | 1.74 |
| TBC1D3B | 17:34493060-34503984 | 0.0011302 | 0.012 | 0.54 | 0.97 | 1.74 |
| PSENEN | 19:36236493-36237903 | 0.0003786 | 0.007 | 153.58 | 266.41 | 1.74 |
| TMEM102 | 17:7338761-7340998 | 0.0006105 | 0.009 | 5.96 | 10.01 | 1.74 |
| HAUS7,TREX2 | X:152710177-152775012 | 0.0014232 | 0.013 | 11.02 | 18.12 | 1.73 |
| ZNF580 | 19:56152391-56154835 | 0.0028194 | 0.018 | 19.86 | 33.86 | 1.73 |
| NMRAL1 | 16:4511694-4560348 | 0.0001038 | 0.005 | 17.78 | 30.04 | 1.73 |
| MAP3K10 | 19:40697650-40721481 | 0.0004179 | 0.008 | 2.52 | 4.33 | 1.73 |
| DGAT1 | 8:145539953-145550573 | 0.0003798 | 0.007 | 23.67 | 41.45 | 1.73 |
| ZNFX1 | 20:47826981-47905797 | 0.0009864 | 0.011 | 36.51 | 62.53 | 1.73 |
| ZNF414 | 19:8575462-8579048 | 0.0010526 | 0.011 | 6.23 | 10.49 | 1.73 |
| RP4-647C14.3.1 | 14:73704204-73741348 | 0.0010443 | 0.011 | 1.86 | 3.08 | 1.73 |
| CAPG | 2:85621345-85645555 | 0.0002568 | 0.007 | 102.37 | 173.11 | 1.73 |
| EEFSEC | 3:127770483-128127485 | 2.40E-07 | 0.002 | 5.69 | 9.86 | 1.73 |
| AGPAT2 | 9:139567594-139581875 | 0.0002451 | 0.007 | 36.34 | 62.73 | 1.73 |
| GTPBP6 | X:192988-230886 | 0.0063372 | 0.028 | 17.66 | 28.44 | 1.73 |
| LSMD1 | 17:7760004-7780399 | 0.0013439 | 0.013 | 50.22 | 86.10 | 1.73 |
| GZMK | 5:54320080-54330398 | 0.0013942 | 0.013 | 30.72 | 51.20 | 1.73 |
| AL928654.7.1,C14orf80,CRIP1 | 14:105952653-105965912 | 0.000254 | 0.007 | 286.79 | 483.53 | 1.73 |
| BAG6 | 6:31606804-31625987 | 0.0081486 | 0.032 | 18.57 | 30.87 | 1.72 |
| LRPPRC | 2:44113362-44223144 | 0.0006504 | 0.009 | 7.57 | 12.99 | 1.72 |
| CD19 | 16:28889725-28950667 | 0.0042646 | 0.022 | 3.53 | 5.88 | 1.72 |
| WDR46 | 6:33246884-33267176 | 0.0003369 | 0.007 | 3.05 | 5.17 | 1.72 |
| LYPLA2 | 1:24117459-24122029 | 0.0000355 | 0.005 | 45.10 | 77.36 | 1.72 |
| FLYWCH2 | 16:2933186-2949383 | 0.0084246 | 0.033 | 8.25 | 13.27 | 1.72 |
| CHST13 | 3:126243125-126277808 | 0.0011388 | 0.012 | 6.01 | 9.69 | 1.72 |
| PRSS36 | 16:31150245-31161415 | 0.0049963 | 0.024 | 1.24 | 2.01 | 1.72 |
| PEX16 | 11:45931219-45940363 | 0.0050175 | 0.024 | 26.23 | 43.26 | 1.72 |
| GPR108 | 19:6677845-6737614 | 0.0001518 | 0.006 | 55.86 | 95.38 | 1.72 |
| CLTB | 5:175792470-175843570 | 0.0005445 | 0.009 | 52.54 | 88.71 | 1.72 |
| TBCB | 19:36602104-36616847 | 0.0002659 | 0.007 | 49.90 | 84.79 | 1.72 |
| SNAPC2 | 19:7985193-8008708 | 0.0002479 | 0.007 | 9.15 | 15.25 | 1.72 |
| IRF2BP1 | 19:46386865-46389376 | 0.0000371 | 0.005 | 5.84 | 10.00 | 1.71 |
| TIMP1 | X:47420515-47479252 | 0.001121 | 0.012 | 179.47 | 300.51 | 1.71 |
| C19orf43 | 19:12841454-12845529 | 0.0004031 | 0.008 | 177.21 | 298.98 | 1.71 |
| C19orf6 | 19:1000417-1021141 | 0.0046196 | 0.023 | 51.73 | 83.94 | 1.71 |
| MOV10 | 1:113215762-113243368 | 0.0044235 | 0.023 | 15.93 | 26.46 | 1.71 |
| TMC6 | 17:76108998-76139049 | 0.0002191 | 0.006 | 53.89 | 91.93 | 1.71 |
| CLSTN1 | 1:9711789-9884584 | 0.0000751 | 0.005 | 9.94 | 16.79 | 1.71 |
| GCHFR | 15:41056217-41059906 | 0.002296 | 0.016 | 12.18 | 20.54 | 1.71 |
| ZNF358 | 19:7581003-7585912 | 0.0015339 | 0.013 | 5.30 | 8.67 | 1.71 |
| FDX1L,ZGLP1 | 19:10415478-10426691 | 0.00975 | 0.036 | 8.12 | 13.01 | 1.71 |
| CTC-260F20.3.1,NDUFA13,YJEFN3 | 19:19623229-19648393 | 0.000143 | 0.006 | 125.81 | 213.81 | 1.71 |
| PTGIR | 19:47123725-47128354 | 0.0086104 | 0.033 | 4.34 | 6.99 | 1.70 |
| IFI27L1 | 14:94547627-94570192 | 0.0127307 | 0.043 | 2.89 | 4.47 | 1.70 |
| LRSAM1 | 9:130213764-130265780 | 0.0023232 | 0.016 | 9.15 | 15.53 | 1.70 |
| MGMT | 10:131265447-131566271 | 0.0071416 | 0.03 | 9.19 | 14.77 | 1.70 |
| NCL | 2:232318241-232348352 | 0.0001317 | 0.006 | 49.31 | 83.91 | 1.70 |
| AC012615.1,ADAT3,SCAMP4 | 19:1905370-1926012 | 0.0009166 | 0.011 | 17.28 | 28.94 | 1.70 |
| PPP2R3B | X:294697-347690 | 0.0116346 | 0.041 | 4.58 | 7.41 | 1.70 |
| MRPL55 | 1:228294379-228297013 | 0.0124345 | 0.042 | 21.55 | 34.10 | 1.70 |
| ZBTB48 | 1:6640060-6649340 | 0.0061173 | 0.027 | 26.08 | 43.17 | 1.70 |
| IGKV3-20 | 2:89442056-89442643 | 0.0009999 | 0.011 | 67.76 | 118.93 | 1.70 |
| PWWP2B | 10:134210671-134231367 | 0.0031787 | 0.019 | 6.12 | 9.95 | 1.70 |
| RP11-977G19.5.1 | 12:56544579-56584068 | 0.0097026 | 0.036 | 10.39 | 16.57 | 1.70 |
| DNTTIP2 | 1:94333372-94345474 | 0.0002936 | 0.007 | 17.69 | 29.32 | 1.70 |
| ATIC | 2:216176539-216214487 | 0.0001527 | 0.006 | 16.67 | 28.05 | 1.70 |
| RP11-274B21.4.1 | 7:128292894-128293719 | 0.002138 | 0.015 | 41.30 | 66.99 | 1.69 |
| EXOSC5 | 19:41836812-42006550 | 0.0006234 | 0.009 | 10.06 | 16.60 | 1.69 |
| SF3A2 | 19:2236815-2248677 | 0.0003136 | 0.007 | 28.16 | 46.87 | 1.69 |
| C12orf52 | 12:113623330-113630173 | 0.0001993 | 0.006 | 5.30 | 8.82 | 1.69 |
| CCL5 | 17:34195970-34212867 | 0.0000149 | 0.004 | 399.23 | 681.96 | 1.69 |
| CCDC102A | 16:57546089-57570511 | 0.0144673 | 0.047 | 1.44 | 2.50 | 1.69 |
| METRNL | 17:81037566-81052585 | 0.0101939 | 0.037 | 32.72 | 53.30 | 1.69 |
| COPE | 19:19010322-19030199 | 0.0033413 | 0.019 | 161.71 | 263.52 | 1.69 |
| GLRX2 | 1:193065597-193075244 | 0.0002839 | 0.007 | 6.80 | 11.28 | 1.69 |
| RPUSD1 | 16:834973-850737 | 0.0030811 | 0.019 | 10.13 | 16.35 | 1.69 |
| NDUFB11 | X:47001614-47046212 | 0.0030956 | 0.019 | 111.54 | 180.69 | 1.69 |
| ECH1 | 19:39306067-39322497 | 0.0030162 | 0.018 | 69.70 | 111.93 | 1.69 |
| SUPV3L1 | 10:70939987-70968855 | 0.0022644 | 0.016 | 9.13 | 14.68 | 1.69 |
| ZNF628 | 19:55987698-55995854 | 0.0096968 | 0.036 | 1.16 | 1.85 | 1.69 |
| WDR19 | 4:39184023-39287430 | 0.0109552 | 0.039 | 2.91 | 4.38 | 1.69 |
| SCAF1 | 19:50145381-50161905 | 0.0008159 | 0.01 | 8.93 | 14.83 | 1.69 |
| ARHGAP4,L1CAM,NAA10,RENBP | X:153126968-153210232 | 0.002187 | 0.016 | 205.35 | 339.76 | 1.68 |
| CLTC | 17:57697218-57773671 | 0.0009342 | 0.011 | 32.76 | 53.51 | 1.68 |
| GRN | 17:42422490-42430470 | 0.0131493 | 0.044 | 261.93 | 407.80 | 1.68 |
| THOC6 | 16:3072625-3077756 | 0.0047354 | 0.024 | 13.94 | 21.91 | 1.68 |
| BOP1 | 8:145486054-145515082 | 0.0138821 | 0.046 | 3.34 | 5.28 | 1.68 |
| GLYCTK-AS1 | 3:52288436-52333083 | 0.0004459 | 0.008 | 5.26 | 8.89 | 1.68 |
| MCM4 | 8:48872744-48890720 | 0.0086142 | 0.033 | 2.22 | 3.40 | 1.68 |
| BST2 | 19:17502081-17516457 | 0.0119503 | 0.041 | 128.79 | 213.56 | 1.68 |
| GLG1 | 16:74485855-74641012 | 0.0010106 | 0.011 | 17.78 | 28.54 | 1.68 |
| FARS2 | 6:5261583-5771816 | 0.0002284 | 0.007 | 5.00 | 8.16 | 1.67 |
| TELO2 | 16:1543344-1662111 | 0.0003625 | 0.007 | 8.28 | 13.45 | 1.67 |
| MRPL23 | 11:1968507-2011150 | 0.0146877 | 0.047 | 49.10 | 75.82 | 1.67 |
| RP11-467M13.1.1 | 16:16472911-16487811 | 0.0049282 | 0.024 | 8.94 | 14.39 | 1.67 |
| FAM110A,RP11-276A18.2.1 | 20:814357-837992 | 0.0006396 | 0.009 | 30.91 | 50.54 | 1.67 |
| CDK5 | 7:150750898-150773617 | 0.000448 | 0.008 | 7.28 | 11.90 | 1.67 |
| THOP1 | 19:2785505-2813588 | 0.0002789 | 0.007 | 3.06 | 5.00 | 1.67 |
| RPS28 | 19:8386383-8408146 | 0.0016196 | 0.013 | 1376.78 | 2245.82 | 1.67 |
| MAP1LC3A | 20:33134657-33148149 | 0.0041662 | 0.022 | 19.52 | 32.99 | 1.67 |
| IFITM1,IFITM2 | 11:307630-315272 | 0.0124517 | 0.042 | 7197.27 | 11631.96 | 1.67 |
| KRT10 | 17:38974368-38992522 | 0.0001431 | 0.006 | 4.67 | 7.88 | 1.67 |
| TRBC2.1 | HG7_PATCH:142561933-142563641 | 0.0001884 | 0.006 | 277.40 | 466.00 | 1.67 |
| TMEM134 | 11:67231823-67236743 | 0.0036671 | 0.02 | 24.99 | 40.43 | 1.67 |
| ZNF777 | 7:149128453-149158214 | 0.0005238 | 0.009 | 2.71 | 4.33 | 1.67 |
| GFER | 16:2034207-2044276 | 0.0072386 | 0.03 | 12.51 | 19.14 | 1.67 |
| HLA-A | 6:29909036-29913661 | 0.0047181 | 0.023 | 887.26 | 1368.01 | 1.67 |
| GTF3C1 | 16:27470875-27561251 | 0.0016 | 0.013 | 12.31 | 19.60 | 1.66 |
| BCAT2 | 19:49298321-49314320 | 0.0038633 | 0.021 | 5.77 | 9.25 | 1.66 |
| RABAC1 | 19:42460837-42463528 | 0.0004585 | 0.008 | 114.60 | 189.50 | 1.66 |
| FCGRT | 19:50015535-50029685 | 0.0010017 | 0.011 | 239.67 | 397.07 | 1.66 |
| TRBC2 | 7:142498724-142500432 | 0.0001959 | 0.006 | 277.22 | 464.09 | 1.66 |
| CARD11 | 7:2945774-3083579 | 0.0013974 | 0.013 | 12.34 | 21.05 | 1.66 |
| RP1-59M18.2.1,SERGEF,TPH1 | 11:17809594-18063973 | 0.0007089 | 0.009 | 14.23 | 22.98 | 1.66 |
| KIR2DL4,KIR3DL1,KIR3DL2,KIR3DL3 | 19:55235968-55378662 | 0.0059932 | 0.027 | 1.93 | 3.36 | 1.66 |
| CXXC5 | 5:139026883-139063467 | 0.0000931 | 0.005 | 6.00 | 9.85 | 1.66 |
| PSMG4 | 6:3224516-3457256 | 0.0000828 | 0.005 | 7.23 | 11.85 | 1.66 |
| TIGD5 | 8:144661805-144682485 | 0.0026157 | 0.017 | 1.31 | 2.09 | 1.66 |
| ACADVL | 17:7093208-7128587 | 0.004348 | 0.022 | 48.33 | 77.01 | 1.66 |
| NIPBL | 5:36876860-37066515 | 0.0004336 | 0.008 | 13.74 | 22.54 | 1.66 |
| WASH2P | 2:114341663-114359144 | 0.0004939 | 0.008 | 14.49 | 24.59 | 1.66 |
| RP5-1109J22.1.1 | 1:46897800-46911374 | 0.0151015 | 0.048 | 1.38 | 2.13 | 1.65 |
| C12orf57,RNU7-1 | 12:7052140-7055166 | 0.0013575 | 0.013 | 82.14 | 134.05 | 1.65 |
| PLXNB2 | 22:50713407-50746056 | 0.0065817 | 0.028 | 20.99 | 33.26 | 1.65 |
| AP000350.4.1 | 22:24198889-24241117 | 0.0068002 | 0.029 | 9.11 | 14.57 | 1.65 |
| LENG8 | 19:54955990-54974894 | 0.0031153 | 0.019 | 81.95 | 133.95 | 1.65 |
| DYRK1B | 19:40315992-40324841 | 0.0003839 | 0.007 | 8.01 | 13.01 | 1.65 |
| ILVBL | 19:15225794-15236596 | 0.0003054 | 0.007 | 5.20 | 8.56 | 1.65 |
| SDHAF1 | 19:36486089-36487220 | 0.0007151 | 0.01 | 10.18 | 16.67 | 1.65 |
| RBBP6 | 16:24549013-24584184 | 0.0110202 | 0.039 | 10.76 | 16.94 | 1.65 |
| PAFAH1B3 | 19:42801184-42814973 | 0.0012806 | 0.012 | 7.18 | 11.57 | 1.65 |
| IGKV4-1 | 2:89184912-89185669 | 0.0080921 | 0.032 | 20.64 | 34.28 | 1.65 |
| MAGED1 | X:51546102-51645453 | 0.0008024 | 0.01 | 4.38 | 7.20 | 1.65 |
| RP11-571M6.7.1 | 12:58087737-58115340 | 0.0009529 | 0.011 | 91.35 | 147.20 | 1.65 |
| RUVBL2 | 19:49497155-49519182 | 0.0003745 | 0.007 | 14.82 | 23.94 | 1.65 |
| BCL11A | 2:60678301-60780702 | 0.0131106 | 0.044 | 6.09 | 9.65 | 1.65 |
| TNK2 | 3:195590234-195638816 | 0.0003196 | 0.007 | 31.66 | 51.72 | 1.65 |
| TRMT61A | 14:103995520-104003410 | 0.0025116 | 0.017 | 2.92 | 4.68 | 1.65 |
| SURF2.1 | HG79_PATCH:136227289-136236704 | 0.0041759 | 0.022 | 7.88 | 12.32 | 1.65 |
| ARFRP1 | 20:62289162-62374858 | 0.0010851 | 0.011 | 19.86 | 31.74 | 1.65 |
| MYO7B | 2:128293377-128395304 | 0.0061622 | 0.027 | 8.07 | 13.92 | 1.65 |
| KCNK7 | 11:65360325-65363467 | 0.0123351 | 0.042 | 2.56 | 4.17 | 1.64 |
| GSTP1 | 11:67351065-67354131 | 0.0012179 | 0.012 | 202.81 | 322.70 | 1.64 |
| ZNF865 | 19:56116770-56128635 | 0.0004726 | 0.008 | 9.06 | 14.80 | 1.64 |
| RP5-1085F17.3.1 | 20:31444847-31446557 | 0.001261 | 0.012 | 7.34 | 11.85 | 1.64 |
| TRGC2 | 7:38279180-38289173 | 0.0023192 | 0.016 | 45.88 | 80.33 | 1.64 |
| AP000721.4.1,COX8A,OTUB1 | 11:63742078-63933578 | 0.0032213 | 0.019 | 307.44 | 483.20 | 1.64 |
| IL32,U1 | 16:3115297-3131908 | 0.0001264 | 0.006 | 374.42 | 619.10 | 1.64 |
| DUSP23 | 1:159750721-159752333 | 0.0010685 | 0.011 | 33.17 | 53.29 | 1.64 |
| FAM50A | X:153672472-153679002 | 0.000758 | 0.01 | 30.82 | 49.57 | 1.64 |
| CYBA | 16:88709690-88717560 | 0.0053726 | 0.025 | 1217.23 | 1919.25 | 1.64 |
| GLTSCR2 | 19:48248792-48260323 | 0.0011856 | 0.012 | 293.34 | 470.66 | 1.64 |
| SNTA1 | 20:31995760-32031698 | 0.0031068 | 0.019 | 1.53 | 2.46 | 1.64 |
| CCDC142,MRPL53 | 2:74699084-74722013 | 0.003216 | 0.019 | 43.35 | 70.09 | 1.64 |
| LGR6 | 1:202162937-202288909 | 0.0015631 | 0.013 | 1.74 | 2.95 | 1.64 |
| GP9 | 3:128779609-128781249 | 0.0002854 | 0.007 | 20.05 | 32.64 | 1.64 |
| BGLAP,PMF1,RP11-54H19.8.1 | 1:156182772-156213123 | 0.0001879 | 0.006 | 38.91 | 63.57 | 1.64 |
| SAC3D1 | 11:64808372-64826021 | 0.0109613 | 0.039 | 4.68 | 7.30 | 1.64 |
| RNASET2,RP11-514O12.4.1,RPS6KA2 | 6:166822851-167370679 | 0.0011518 | 0.012 | 482.46 | 780.62 | 1.64 |
| CYP2D6,CYP2D7P1 | 22:42475694-42540576 | 0.0117102 | 0.041 | 2.90 | 4.92 | 1.64 |
| ABHD8 | 19:17402940-17414282 | 0.0002285 | 0.007 | 7.02 | 11.14 | 1.64 |
| RASAL3 | 19:15562437-15575382 | 0.0000883 | 0.005 | 27.58 | 45.19 | 1.64 |
| ABI3 | 17:47280152-47300587 | 0.0013666 | 0.013 | 25.16 | 41.57 | 1.64 |
| ERI3 | 1:44686741-44820932 | 0.0001942 | 0.006 | 12.53 | 20.30 | 1.64 |
| SUV420H2 | 19:55851220-55859488 | 0.002734 | 0.017 | 5.17 | 8.26 | 1.64 |
| PRR14 | 16:30662037-30667761 | 0.000161 | 0.006 | 34.39 | 56.21 | 1.64 |
| XXyac-YRM2039.2.1 | 9:11986-30891 | 0.0055617 | 0.026 | 15.16 | 25.06 | 1.64 |
| CHMP2A | 19:59062933-59066486 | 0.0005594 | 0.009 | 187.21 | 307.45 | 1.64 |
| EPHB6 | 7:142552791-142568847 | 0.0011011 | 0.011 | 3.61 | 5.75 | 1.64 |
| YDJC | 22:21982377-21984353 | 0.0000379 | 0.005 | 13.77 | 22.54 | 1.64 |
| AC006276.7.1 | 19:44084695-44088114 | 0.0139331 | 0.046 | 1.61 | 2.30 | 1.64 |
| GMPPA | 2:220238267-220586393 | 0.0000873 | 0.005 | 14.14 | 22.81 | 1.63 |
| ACAP3 | 1:1227755-1260071 | 0.0045547 | 0.023 | 12.31 | 18.92 | 1.63 |
| NOC2L | 1:860259-894689 | 0.0030703 | 0.018 | 11.04 | 17.82 | 1.63 |
| SF3B4 | 1:149895208-149900236 | 0.0013881 | 0.013 | 42.68 | 69.49 | 1.63 |
| RPS15 | 19:1438362-1440492 | 0.0108272 | 0.039 | 1052.80 | 1632.42 | 1.63 |
| NUDT18 | 8:21964382-21966932 | 0.0009542 | 0.011 | 7.01 | 11.16 | 1.63 |
| FLOT1 | HSCHR6_MHC_MANN:30740182-30743574 | 0.0003469 | 0.007 | 19.68 | 32.45 | 1.63 |
| ARAP1-AS1 | 11:72396113-72505213 | 0.0027417 | 0.017 | 128.12 | 205.93 | 1.63 |
| HEXB | 5:73923233-74072737 | 0.0013524 | 0.013 | 71.75 | 115.52 | 1.63 |
| ITPA | 20:3189513-3204516 | 0.0053686 | 0.025 | 23.42 | 37.67 | 1.63 |
| MRVI1-AS1 | 11:10562818-10715535 | 0.0147389 | 0.047 | 2.63 | 3.83 | 1.63 |
| HLA-DQB1,HLA-DRB1,HLA-DRB4,XXbac-BPG254F23.6.6 | HSCHR6_MHC_SSTO:32509572-32771022 | 0.0068856 | 0.029 | 195.46 | 313.37 | 1.63 |
| FUZ | 19:50270179-50320633 | 0.000454 | 0.008 | 16.48 | 27.09 | 1.63 |
| AARS | 16:70286292-70407286 | 0.0011755 | 0.012 | 5.54 | 9.10 | 1.63 |
| SYMPK | 19:46318692-46366548 | 0.000569 | 0.009 | 10.64 | 17.04 | 1.63 |
| RP11-265M18.2.1 | 15:63796792-63894627 | 0.0048756 | 0.024 | 14.37 | 22.43 | 1.63 |
| OBSCN | 1:228391206-228566575 | 0.0017487 | 0.014 | 1.49 | 2.36 | 1.63 |
| AQR | 15:35147731-35262040 | 0.0053262 | 0.025 | 6.42 | 9.94 | 1.63 |
| SYTL1 | 1:27668512-27680421 | 0.0012289 | 0.012 | 57.12 | 93.45 | 1.63 |
| TBC1D10C | 11:67159175-67193078 | 0.0017281 | 0.014 | 138.99 | 224.23 | 1.63 |
| NAGPA | 16:5074844-5147809 | 0.0038014 | 0.021 | 8.52 | 12.99 | 1.63 |
| ANXA11 | 10:81910644-81965433 | 0.0018867 | 0.014 | 206.50 | 336.33 | 1.63 |
| APOBR | 16:28467692-28510291 | 0.0002041 | 0.006 | 45.82 | 73.95 | 1.63 |
| CD1C | 1:158259562-158264564 | 0.0097215 | 0.036 | 8.21 | 11.85 | 1.62 |
| LSR | 19:35739558-35758865 | 0.0015137 | 0.013 | 3.36 | 5.07 | 1.62 |
| EVI5L | 19:7895160-7929861 | 0.0128538 | 0.043 | 2.32 | 3.51 | 1.62 |
| PUF60 | 8:144898513-144912029 | 0.0000279 | 0.004 | 20.02 | 32.27 | 1.62 |
| PUF60.1 | HG104_HG975_PATCH:144898513-144912029 | 0.0000279 | 0.004 | 20.02 | 32.27 | 1.62 |
| SLC39A4.1 | HG243_PATCH:145464644-145471798 | 0.0032387 | 0.019 | 3.83 | 6.02 | 1.62 |
| KCTD14,NDUFC2,NDUFC2-KCTD14 | 11:77726760-77791265 | 0.0002984 | 0.007 | 99.32 | 159.43 | 1.62 |
| HMOX2 | 16:4511694-4560348 | 0.0001184 | 0.006 | 31.32 | 50.95 | 1.62 |
| IL2RG | X:70327253-70331958 | 0.0002916 | 0.007 | 234.00 | 374.72 | 1.62 |
| MVD | 16:88718342-88729569 | 0.0074785 | 0.031 | 14.23 | 21.70 | 1.62 |
| HSD17B10 | X:53458205-53461320 | 0.0001507 | 0.006 | 38.58 | 61.70 | 1.62 |
| GPAT2 | 2:96687693-96705199 | 0.0072616 | 0.03 | 0.81 | 1.27 | 1.62 |
| MIA | 19:41277552-41302847 | 0.0043691 | 0.022 | 67.00 | 104.97 | 1.62 |
| GHDC | 17:40341105-40346550 | 0.0010458 | 0.011 | 8.30 | 13.14 | 1.62 |
| MRPL4 | 19:10362639-10370736 | 0.005312 | 0.025 | 14.49 | 22.42 | 1.62 |
| NCR3 | 6:31553900-31560762 | 0.0000524 | 0.005 | 1.95 | 3.23 | 1.62 |
| TUBGCP2 | 10:135093134-135166187 | 0.0014078 | 0.013 | 23.72 | 37.56 | 1.62 |
| FOLR2 | 11:71927644-71932994 | 0.0001203 | 0.006 | 4.13 | 6.61 | 1.62 |
| SLC39A4 | 8:145635125-145642279 | 0.0033748 | 0.019 | 3.83 | 6.01 | 1.62 |
| OGFR | 20:61431978-61445352 | 0.000087 | 0.005 | 57.12 | 91.92 | 1.62 |
| PSTPIP1 | 15:77285699-77329673 | 0.000992 | 0.011 | 112.20 | 182.07 | 1.61 |
| TMEM175 | 4:926174-952444 | 0.0036031 | 0.02 | 26.02 | 40.79 | 1.61 |
| COG4 | 16:70514470-70557468 | 0.0055329 | 0.026 | 18.33 | 28.78 | 1.61 |
| CDK2AP2 | 11:67273967-67276120 | 0.0000116 | 0.003 | 51.07 | 82.62 | 1.61 |
| RP11-196G11.3.1 | 16:31085742-31107301 | 0.0016891 | 0.014 | 3.17 | 5.02 | 1.61 |
| MYO1G | 7:45002260-45018697 | 0.000203 | 0.006 | 60.56 | 96.59 | 1.61 |
| MED24 | 17:38175349-38210661 | 0.0010061 | 0.011 | 9.33 | 14.45 | 1.61 |
| TMEM40 | 3:12775023-12810956 | 0.0081104 | 0.032 | 9.50 | 14.44 | 1.61 |
| AL358781.1 | 9:134269479-134375584 | 0.0000542 | 0.005 | 7.81 | 12.53 | 1.61 |
| TYK2 | 19:10461208-10491352 | 0.0008036 | 0.01 | 149.59 | 241.97 | 1.61 |
| TRIP6 | 7:100464759-100471076 | 0.0033674 | 0.019 | 4.15 | 6.38 | 1.61 |
| TMEM88 | 17:7758383-7759415 | 0.00427 | 0.022 | 6.82 | 10.53 | 1.61 |
| AXIN1 | 16:337439-402673 | 0.0001862 | 0.006 | 14.17 | 22.55 | 1.61 |
| BAIAP3 | 16:1383601-1401912 | 0.0051563 | 0.025 | 4.15 | 6.95 | 1.61 |
| LLGL2 | 17:73521782-73571289 | 0.0018503 | 0.014 | 5.60 | 9.30 | 1.61 |
| RRP9 | 3:51967445-51975957 | 0.0009662 | 0.011 | 4.34 | 6.83 | 1.61 |
| C1orf21 | 1:184356191-184598154 | 0.0005674 | 0.009 | 8.01 | 12.75 | 1.61 |
| RNASE6 | 14:21249209-21250626 | 0.0058552 | 0.027 | 92.00 | 143.34 | 1.61 |
| SLC6A12 | 12:299242-323736 | 0.014447 | 0.047 | 1.97 | 3.11 | 1.61 |
| MIR761,NRD1 | 1:52027537-52344477 | 0.0017294 | 0.014 | 85.46 | 135.92 | 1.61 |
| DGCR14 | 22:19117791-19132197 | 0.0002808 | 0.007 | 8.04 | 12.77 | 1.61 |
| PPP1R35 | 7:100032879-100036127 | 0.0017796 | 0.014 | 17.36 | 27.28 | 1.60 |
| CXCR3 | X:70835765-70838367 | 0.0080792 | 0.032 | 8.70 | 13.57 | 1.60 |
| AP1G2,JPH4 | 14:23980968-24048009 | 0.0000405 | 0.005 | 47.99 | 77.01 | 1.60 |
| SEPT1 | 16:30389426-30411429 | 0.000807 | 0.01 | 44.21 | 70.59 | 1.60 |
| SAFB2 | 19:5587010-5622938 | 0.0001282 | 0.006 | 16.01 | 25.40 | 1.60 |
| B3GNT1 | 11:66112842-66115163 | 0.0007965 | 0.01 | 1.10 | 1.74 | 1.60 |
| PEX10 | 1:2336235-2345236 | 0.0005231 | 0.009 | 10.65 | 16.94 | 1.60 |
| ZNF574 | 19:42572863-42585717 | 0.0083862 | 0.033 | 3.85 | 5.95 | 1.60 |
| RP11-1079K10.4.1 | 17:47481413-47492246 | 0.000539 | 0.009 | 11.08 | 17.50 | 1.60 |
| C20orf27 | 20:3734154-3749034 | 0.0082965 | 0.033 | 34.93 | 53.24 | 1.60 |
| CHMP6 | 17:78965640-78973932 | 0.0050815 | 0.024 | 14.81 | 22.75 | 1.60 |
| NDUFB8,RP11-411B6.6.1,SEC31B | 10:102246398-102309763 | 0.0001459 | 0.006 | 105.27 | 165.85 | 1.60 |
| SRM | 1:11114640-11120081 | 0.00011 | 0.006 | 20.25 | 32.05 | 1.60 |
| BX248519.1,NCR3 | HSCHR6_MHC_SSTO:31543844-31550705 | 0.0000574 | 0.005 | 1.93 | 3.14 | 1.60 |
| RAD54L2,TEX264 | 3:51575595-51738339 | 0.0001485 | 0.006 | 32.57 | 52.09 | 1.60 |
| BX927320.1,NCR3 | HSCHR6_MHC_MANN:31593387-31600249 | 0.0000581 | 0.005 | 1.93 | 3.14 | 1.60 |
| CD97 | 19:14492212-14519531 | 0.0002022 | 0.006 | 181.79 | 288.71 | 1.60 |
| DCTN1 | 2:74588280-74621009 | 0.0153142 | 0.048 | 26.97 | 40.31 | 1.59 |
| MMP19 | 12:56137063-56236750 | 0.0019745 | 0.015 | 2.28 | 3.56 | 1.59 |
| RANGAP1 | 22:41641614-41756151 | 0.0018428 | 0.014 | 14.76 | 22.73 | 1.59 |
| AC004410.1 | 19:2328628-2355099 | 0.0013717 | 0.013 | 5.05 | 7.95 | 1.59 |
| GNL1 | HSCHR6_MHC_APD:30517399-30529581 | 0.0000465 | 0.005 | 1.79 | 2.84 | 1.59 |
| NDUFA10 | 2:240831866-240964819 | 0.0001574 | 0.006 | 30.50 | 48.17 | 1.59 |
| TSPAN4 | 11:842807-867116 | 0.0008414 | 0.01 | 9.92 | 15.38 | 1.59 |
| RP11-469M7.1.1 | 2:201689395-201692061 | 0.003054 | 0.018 | 0.95 | 1.49 | 1.59 |
| NUP205 | 7:135242666-135333505 | 0.0001055 | 0.005 | 7.55 | 11.93 | 1.59 |
| TCEA2 | 20:62681188-62703700 | 0.0010396 | 0.011 | 12.01 | 18.81 | 1.59 |
| DOM3Z | HSCHR6_MHC_QBL:31815829-32011521 | 0.0015227 | 0.013 | 0.85 | 1.33 | 1.59 |
| C17orf70 | 17:79506910-79520987 | 0.0022013 | 0.016 | 11.37 | 17.80 | 1.59 |
| MXD3,RAB24 | 5:176728198-176739758 | 0.0049189 | 0.024 | 168.08 | 264.28 | 1.59 |
| LRWD1 | 7:102105375-102119354 | 0.0034177 | 0.019 | 11.32 | 17.52 | 1.59 |
| PPP1R37 | 19:45595049-45651335 | 0.0045345 | 0.023 | 4.34 | 6.80 | 1.59 |
| HYI | 1:43855552-43922666 | 0.0026453 | 0.017 | 5.12 | 7.82 | 1.59 |
| RP3-402G11.5.1 | 22:50639407-50656045 | 0.0050909 | 0.024 | 22.41 | 34.39 | 1.59 |
| RP11-34P13.11.1 | 1:134900-139697 | 0.0093279 | 0.035 | 308.50 | 488.25 | 1.59 |
| WDR54 | 2:74648804-74652882 | 0.0008339 | 0.01 | 6.51 | 10.24 | 1.59 |
| EXD3 | 9:140201347-140317714 | 0.0000649 | 0.005 | 2.81 | 4.46 | 1.59 |
| S1PR5 | 19:10623418-10628668 | 0.0044622 | 0.023 | 16.06 | 26.01 | 1.59 |
| SAMD1 | 19:14198651-14201848 | 0.0002365 | 0.007 | 7.67 | 12.06 | 1.59 |
| RP11-50C13.1.1 | 15:58879705-58883875 | 0.0137917 | 0.045 | 4.43 | 6.63 | 1.59 |
| MTX1 | 1:155165378-155197214 | 0.0007397 | 0.01 | 36.35 | 58.64 | 1.59 |
| BAD | 11:64037301-64056972 | 0.0038083 | 0.021 | 20.22 | 31.16 | 1.59 |
| SLC16A3 | 17:80186292-80197369 | 0.0065733 | 0.028 | 136.66 | 204.76 | 1.59 |
| AC018462.3.1,COMMD1 | 2:62095223-62374382 | 0.0019157 | 0.015 | 22.91 | 35.23 | 1.59 |
| EIF5B | 2:99613723-100016728 | 0.0003467 | 0.007 | 7.19 | 11.18 | 1.59 |
| HMHA1 | 19:1065929-1095391 | 0.0021607 | 0.015 | 136.23 | 208.87 | 1.59 |
| PARP1 | 1:224102740-226595780 | 0.0005539 | 0.009 | 12.62 | 19.67 | 1.59 |
| DGKZ | 11:46354454-46402104 | 0.000621 | 0.009 | 57.05 | 90.77 | 1.58 |
| RBM10 | X:47001614-47046212 | 0.0005304 | 0.009 | 15.31 | 23.95 | 1.58 |
| MAN2B1 | 19:12757324-12777556 | 0.0075771 | 0.031 | 88.87 | 135.50 | 1.58 |
| C21orf59,TCP10L | 21:33948861-33985176 | 0.0000602 | 0.005 | 9.71 | 15.21 | 1.58 |
| ASB1 | 2:239335382-239360891 | 0.0087358 | 0.034 | 8.73 | 13.51 | 1.58 |
| TRBV4-3 | HG7_PATCH:142062480-142062934 | 0.0017719 | 0.014 | 12.81 | 20.58 | 1.58 |
| ENO3 | 17:4848946-4860426 | 0.0095344 | 0.036 | 14.33 | 22.31 | 1.58 |
| WDR70 | 5:37379313-37753537 | 0.0020328 | 0.015 | 8.70 | 13.50 | 1.58 |
| FAM159A | 1:53099015-53135355 | 0.0027878 | 0.018 | 8.04 | 12.51 | 1.58 |
| XAB2 | 19:7684411-7694451 | 0.0003386 | 0.007 | 15.85 | 24.72 | 1.58 |
| ZNF385A | 12:54747444-54937889 | 0.0111098 | 0.039 | 40.34 | 59.33 | 1.58 |
| NOSIP | 19:50058969-50083813 | 0.0002153 | 0.006 | 97.28 | 151.46 | 1.58 |
| ALG12 | 22:50296866-50321188 | 0.0001196 | 0.006 | 7.88 | 12.32 | 1.58 |
| DDX54 | 12:113587662-113623284 | 0.000841 | 0.01 | 10.14 | 16.23 | 1.58 |
| PSMB6 | 17:4699438-4701798 | 0.0044773 | 0.023 | 77.39 | 116.83 | 1.58 |
| SH2B2.1 | HG115_PATCH:101928440-101964991 | 0.0012394 | 0.012 | 11.32 | 17.98 | 1.58 |
| ICAM2 | 17:62075710-62097994 | 0.0001639 | 0.006 | 47.82 | 75.14 | 1.58 |
| TNNI2 | 11:1860218-1862910 | 0.0078135 | 0.032 | 13.73 | 21.48 | 1.58 |
| RP9 | 7:33134408-33149013 | 0.0064512 | 0.028 | 6.40 | 9.61 | 1.58 |
| RP11-638I2.6.1 | 14:100657422-100772884 | 0.0132978 | 0.044 | 21.00 | 32.45 | 1.58 |
| CDK11A | 1:1634168-1655966 | 0.0003208 | 0.007 | 25.04 | 39.57 | 1.57 |
| MYBPC3 | 11:47290711-47374253 | 0.0020412 | 0.015 | 1.74 | 2.71 | 1.57 |
| CAPS | 19:5911385-5978320 | 0.013761 | 0.045 | 10.32 | 15.94 | 1.57 |
| PVRIG,STAG3 | 7:99775185-99869855 | 0.0041848 | 0.022 | 18.46 | 29.62 | 1.57 |
| XYLT2 | 17:48423452-48440499 | 0.0001186 | 0.006 | 3.93 | 6.21 | 1.57 |
| ATP6V1H | 8:54628116-54756118 | 0.0136063 | 0.045 | 14.19 | 21.18 | 1.57 |
| CORO1A | 16:30194147-30200397 | 0.0003092 | 0.007 | 889.92 | 1402.75 | 1.57 |
| FOXH1 | 8:145691425-145701718 | 0.0004911 | 0.008 | 3.07 | 4.77 | 1.57 |
| RP11-783K16.14.1 | 11:63974149-64001824 | 0.0019519 | 0.015 | 2.48 | 3.88 | 1.57 |
| PTPN6 | 12:7055630-7070479 | 0.0008243 | 0.01 | 269.88 | 421.76 | 1.57 |
| AC174470.1,FAM195B | 17:79780292-79791167 | 0.0002911 | 0.007 | 47.42 | 73.94 | 1.57 |
| SERTAD1 | 19:40927498-40931932 | 0.0016085 | 0.013 | 7.90 | 12.07 | 1.57 |
| TMEM176A | 7:150488372-150502208 | 0.0026778 | 0.017 | 48.03 | 68.53 | 1.57 |
| EPN1 | 19:56186560-56207132 | 0.0032097 | 0.019 | 40.64 | 61.91 | 1.57 |
| C22orf29,GNB1L | 22:19744225-19842462 | 0.0031856 | 0.019 | 6.08 | 9.33 | 1.57 |
| TRAP1 | 16:3692938-3767565 | 0.0002651 | 0.007 | 8.54 | 13.20 | 1.57 |
| AP003419.16.1 | 11:67195930-67202872 | 0.007665 | 0.031 | 9.91 | 14.87 | 1.57 |
| NUP214 | 9:134000947-134110057 | 0.0091761 | 0.035 | 236.63 | 361.32 | 1.57 |
| ETFB | 19:51848409-51869672 | 0.0084138 | 0.033 | 41.23 | 62.09 | 1.57 |
| TOP2B | 3:25639474-25706398 | 0.0002114 | 0.006 | 23.54 | 36.81 | 1.57 |
| PEMT | 17:17408876-17495022 | 0.0011567 | 0.012 | 7.36 | 11.37 | 1.57 |
| SHKBP1 | 19:41082756-41097301 | 0.0012002 | 0.012 | 162.06 | 250.61 | 1.57 |
| SELPLG | 12:109015685-109035094 | 0.0001091 | 0.006 | 289.19 | 455.62 | 1.57 |
| UNC93B1 | 11:67758574-67772452 | 0.0070818 | 0.03 | 59.01 | 90.30 | 1.57 |
| HSP90B1 | 12:104235228-104359486 | 0.0006695 | 0.009 | 60.27 | 93.11 | 1.57 |
| SIRT7 | 17:79869814-79876052 | 0.0013521 | 0.013 | 30.14 | 46.50 | 1.57 |
| AC010536.1,KLHDC4 | 16:87635440-87799598 | 0.0000635 | 0.005 | 11.01 | 17.00 | 1.57 |
| CCDC94 | 19:4247086-4269083 | 0.0002159 | 0.006 | 10.99 | 16.95 | 1.57 |
| TRAPPC9 | 8:140742585-141468678 | 0.004364 | 0.022 | 7.82 | 11.83 | 1.57 |
| PLA2G16 | 11:63340666-63389143 | 0.0018213 | 0.014 | 8.99 | 13.91 | 1.57 |
| BRAT1 | 7:2577510-2595361 | 0.0116362 | 0.041 | 15.14 | 22.72 | 1.57 |
| DNAJB11 | 3:186256229-186315061 | 0.0007981 | 0.01 | 20.12 | 30.67 | 1.57 |
| LETM2 | 8:38243724-38267045 | 0.0031776 | 0.019 | 5.66 | 8.17 | 1.56 |
| EHBP1L1 | 11:65343508-65360121 | 0.0053828 | 0.025 | 96.81 | 150.10 | 1.56 |
| RP11-744N12.2.1 | 11:128551134-128683161 | 0.0113535 | 0.04 | 3.88 | 5.95 | 1.56 |
| PHLDB3 | 19:43979254-44009087 | 0.0018845 | 0.014 | 0.98 | 1.52 | 1.56 |
| DVL1 | 1:1266693-1284760 | 0.0093672 | 0.035 | 5.09 | 7.60 | 1.56 |
| PPME1 | 11:73723762-74035750 | 0.0008873 | 0.01 | 5.62 | 8.59 | 1.56 |
| EIF3G | 19:10216898-10230599 | 0.0003222 | 0.007 | 129.86 | 200.40 | 1.56 |
| SAFB | 19:5623149-5668488 | 0.0001397 | 0.006 | 26.97 | 41.66 | 1.56 |
| AP000346.2.1,AP000347.4.1,ASLP1,C22orf43,GUSBP11 | 22:23950638-24059543 | 0.0005262 | 0.009 | 29.27 | 45.18 | 1.56 |
| AGER | HSCHR6_MHC_MCF:32222562-32228531 | 0.0123848 | 0.042 | 2.47 | 3.95 | 1.56 |
| KCTD17 | 22:37447778-37459430 | 0.0017148 | 0.014 | 7.95 | 12.32 | 1.56 |
| ATP13A2 | 1:17300996-17338423 | 0.0016053 | 0.013 | 10.96 | 16.57 | 1.56 |
| SCRN2 | 17:45908992-45918699 | 0.0058058 | 0.026 | 6.42 | 9.75 | 1.56 |
| RP11-384K6.2.1,RP11-384K6.6.1 | 4:119512927-119556134 | 0.0027782 | 0.018 | 6.00 | 9.01 | 1.56 |
| PTGES2 | 9:130882971-130891481 | 0.0029219 | 0.018 | 12.57 | 19.10 | 1.56 |
| CHRNE | 17:4736634-4806369 | 0.0001806 | 0.006 | 1.35 | 2.07 | 1.56 |
| NSDHL | X:151999510-152038273 | 0.0007336 | 0.01 | 4.39 | 6.59 | 1.56 |
| CD72 | 9:35605280-35647096 | 0.0000702 | 0.005 | 5.56 | 8.62 | 1.56 |
| TMEM93 | 17:3566195-3599698 | 0.0000947 | 0.005 | 27.16 | 42.13 | 1.56 |
| AC006547.8.1,TRMT2A | 22:20067754-20114878 | 0.0000759 | 0.005 | 24.30 | 37.92 | 1.56 |
| HRAS | 11:532241-537287 | 0.0028251 | 0.018 | 10.45 | 16.07 | 1.56 |
| NCLN | 19:3185874-3209572 | 0.0035579 | 0.02 | 16.88 | 25.47 | 1.55 |
| PTOV1 | 19:50354137-50363990 | 0.0044181 | 0.023 | 28.32 | 43.34 | 1.55 |
| MCAT | 22:43528885-43539400 | 0.0114318 | 0.04 | 4.23 | 6.17 | 1.55 |
| SEPW1 | 19:48281841-48287941 | 0.0024895 | 0.017 | 49.89 | 76.86 | 1.55 |
| GMDS | 6:1624040-2245926 | 0.0020425 | 0.015 | 11.49 | 17.45 | 1.55 |
| C1orf159 | 1:1017197-1051741 | 0.0078471 | 0.032 | 3.32 | 4.91 | 1.55 |
| HSPA1B | 6:31795511-31798031 | 0.0005454 | 0.009 | 8.48 | 13.20 | 1.55 |
| TCOF1 | 5:149737201-149779870 | 0.0039177 | 0.021 | 4.26 | 6.47 | 1.55 |
| PSMC5 | 17:61896794-61909386 | 0.0000291 | 0.004 | 51.05 | 79.05 | 1.55 |
| DRAP1 | 11:65686727-65689032 | 0.0016198 | 0.013 | 102.68 | 155.84 | 1.55 |
| MRPS12 | 19:39405903-39466380 | 0.0006753 | 0.009 | 16.97 | 25.80 | 1.55 |
| NAGLU | 17:40687950-40696464 | 0.0013575 | 0.013 | 5.36 | 8.16 | 1.55 |
| FAM83G | 17:18853988-18950439 | 0.0001263 | 0.006 | 0.77 | 1.23 | 1.55 |
| CUEDC2 | 10:104183001-104192418 | 0.0002958 | 0.007 | 26.15 | 40.08 | 1.55 |
| DNASE1L2,E4F1 | 16:2273559-2288712 | 0.0027395 | 0.017 | 7.83 | 11.62 | 1.55 |
| VPS45 | 1:150039368-150117505 | 0.000587 | 0.009 | 14.57 | 22.20 | 1.55 |
| FBP1 | 9:97365414-97402531 | 0.0085317 | 0.033 | 19.27 | 29.34 | 1.55 |
| NR2F6 | 19:17342693-17356151 | 0.0025614 | 0.017 | 1.75 | 2.74 | 1.55 |
| BLM | 15:91260557-91358859 | 0.0014156 | 0.013 | 2.64 | 3.85 | 1.55 |
| AC005329.7.1 | 19:1383525-1396466 | 0.0033897 | 0.019 | 1.17 | 1.83 | 1.55 |
| NOP58,SNORD70 | 2:203130438-203168389 | 0.0052125 | 0.025 | 17.64 | 26.33 | 1.55 |
| CCT3 | 1:156278758-156337664 | 7.15E-06 | 0.003 | 39.76 | 61.64 | 1.54 |
| LENG1 | 19:54641443-54663620 | 0.0014876 | 0.013 | 8.34 | 12.77 | 1.54 |
| CDK11B | 1:1571099-1588935 | 4.38E-06 | 0.003 | 15.43 | 23.69 | 1.54 |
| SCAMP3 | 1:155225769-155232221 | 0.0000121 | 0.003 | 26.97 | 41.31 | 1.54 |
| MRM1 | 17:34958000-34965397 | 0.002438 | 0.016 | 1.73 | 2.69 | 1.54 |
| GARS | 7:30634296-30673649 | 0.0070492 | 0.03 | 23.33 | 34.78 | 1.54 |
| HGS | 17:79651019-79669145 | 0.0008131 | 0.01 | 26.34 | 40.28 | 1.54 |
| APBA3 | 19:3708381-3761673 | 0.0008433 | 0.01 | 9.47 | 14.34 | 1.54 |
| LGALS3BP | 17:76967337-76976061 | 0.003067 | 0.018 | 8.49 | 12.54 | 1.54 |
| TDRD3 | 13:60970590-61148012 | 0.0000671 | 0.005 | 3.39 | 5.24 | 1.54 |
| ATXN7L2 | 1:110026100-110035426 | 0.0068771 | 0.029 | 2.08 | 3.03 | 1.54 |
| MAD2L2 | 1:11734537-11751707 | 0.000134 | 0.006 | 24.29 | 37.34 | 1.54 |
| DNAJC17 | 15:41060066-41106767 | 0.0003925 | 0.008 | 9.15 | 13.93 | 1.54 |
| CCDC9 | 19:47759730-47775208 | 0.0015483 | 0.013 | 10.13 | 15.44 | 1.54 |
| FKBP2 | 11:64008474-64011604 | 0.0024834 | 0.017 | 33.48 | 50.31 | 1.54 |
| FAM50B | 6:3832168-3857093 | 0.0078447 | 0.032 | 2.78 | 4.18 | 1.54 |
| NUDC | 1:27216978-27273353 | 0.0008692 | 0.01 | 23.50 | 35.62 | 1.54 |
| RNF113A | X:119004496-119010625 | 0.0000729 | 0.005 | 13.43 | 20.50 | 1.54 |
| USP5 | 12:6961291-6975796 | 0.0037349 | 0.021 | 9.68 | 14.24 | 1.54 |
| PIAS4 | 19:4007747-4039383 | 0.0003969 | 0.008 | 6.95 | 10.49 | 1.54 |
| C19orf53 | 19:13885256-13889582 | 0.0038477 | 0.021 | 48.45 | 73.15 | 1.54 |
| RP11-577H5.2.1 | 7:102322282-102330916 | 0.0106892 | 0.038 | 2.10 | 2.94 | 1.54 |
| SLC25A1 | 22:19163094-19166343 | 0.0024962 | 0.017 | 21.26 | 32.15 | 1.54 |
| LINC00174 | 7:65670185-65955095 | 0.0021247 | 0.015 | 2.25 | 3.47 | 1.54 |
| ATAD3B | 1:1407142-1433228 | 0.0028723 | 0.018 | 4.52 | 6.92 | 1.54 |
| OSBPL7 | 17:45884732-45899200 | 0.0005379 | 0.009 | 3.45 | 5.33 | 1.54 |
| TXNRD2 | 22:19863039-20004331 | 0.0112996 | 0.04 | 13.27 | 19.91 | 1.54 |
| RP11-169D4.2.1 | 11:72287184-72385635 | 0.0067824 | 0.029 | 2.37 | 3.54 | 1.54 |
| BATF | 14:75988767-76013358 | 0.0000109 | 0.003 | 25.56 | 39.57 | 1.54 |
| RP11-256P1.1.1 | 5:137620953-137685416 | 0.0039406 | 0.021 | 41.95 | 65.00 | 1.54 |
| FOXP4 | 6:41462590-41570122 | 0.0011969 | 0.012 | 1.87 | 2.79 | 1.54 |
| MLF2 | 12:6857169-6880118 | 0.0024249 | 0.016 | 102.90 | 157.87 | 1.54 |
| BRMS1 | 11:66097712-66112596 | 0.0003918 | 0.008 | 35.50 | 54.03 | 1.54 |
| CPNE1,NFS1,RBM12 | 20:34213952-34288906 | 0.0008205 | 0.01 | 140.00 | 214.68 | 1.53 |
| HNRNPA2B1 | 7:26229546-26252976 | 0.0021328 | 0.015 | 150.17 | 224.04 | 1.53 |
| MRPL51 | 12:6601149-6641121 | 0.0002418 | 0.007 | 56.83 | 85.78 | 1.53 |
| PDLIM7 | 5:176910394-176938275 | 0.0011807 | 0.012 | 119.61 | 184.98 | 1.53 |
| FGFBP2,PROM1 | 4:15961865-16086001 | 0.0095638 | 0.036 | 63.39 | 97.15 | 1.53 |
| MAP4K1 | 19:39078280-39108643 | 0.0009651 | 0.011 | 19.14 | 29.72 | 1.53 |
| MRPS15 | 1:36921318-36930038 | 0.0019803 | 0.015 | 28.37 | 43.33 | 1.53 |
| NDUFAB1 | 16:23592322-23607677 | 0.0012758 | 0.012 | 44.97 | 67.66 | 1.53 |
| CCDC101 | 16:28565235-28603111 | 0.0004957 | 0.008 | 17.63 | 27.06 | 1.53 |
| DNAJC1 | 10:22045465-22292698 | 0.0021338 | 0.015 | 9.16 | 13.72 | 1.53 |
| P2RY11,PPAN,PPAN-P2RY11 | 19:10216898-10230599 | 0.003473 | 0.02 | 15.93 | 24.65 | 1.53 |
| ABC7-42389800N19.1.2 | HG858_PATCH:64521-82015 | 0.0033136 | 0.019 | 6.13 | 8.45 | 1.53 |
| SLC38A10 | 17:79218799-79269347 | 0.0049117 | 0.024 | 24.64 | 36.31 | 1.53 |
| TBX21 | 17:45810609-45823485 | 0.012832 | 0.043 | 17.11 | 27.08 | 1.53 |
| AP000351.3.1,DDT | 22:24309088-24326106 | 0.000661 | 0.009 | 68.68 | 104.91 | 1.53 |
| FERMT3 | 11:63974149-64001824 | 0.0002042 | 0.006 | 120.90 | 183.95 | 1.53 |
| CCDC137 | 17:79633760-79640937 | 0.0001131 | 0.006 | 6.51 | 9.82 | 1.53 |
| FAR2 | 12:29302035-29534122 | 0.0106421 | 0.038 | 12.99 | 19.72 | 1.53 |
| PHB | 17:47481413-47492246 | 0.0001747 | 0.006 | 38.09 | 57.15 | 1.53 |
| ATP5C1 | 10:7830091-7849778 | 0.0001236 | 0.006 | 76.84 | 116.03 | 1.53 |
| DENND1C | 19:6464259-6481798 | 0.0001523 | 0.006 | 32.61 | 49.68 | 1.53 |
| NDUFS3,PTPMT1 | 11:47487495-47606114 | 0.0002334 | 0.007 | 62.15 | 94.12 | 1.53 |
| CD151 | 11:818901-842545 | 0.0031715 | 0.019 | 37.31 | 55.54 | 1.53 |
| LTBP3 | 11:65292547-65326401 | 0.0075067 | 0.031 | 13.26 | 19.31 | 1.53 |
| AC011498.1 | 19:4472254-4517716 | 0.0030679 | 0.018 | 11.26 | 16.88 | 1.53 |
| ACOT7 | 1:6324332-6454451 | 0.0069693 | 0.029 | 4.08 | 5.81 | 1.53 |
| SNRPA | 19:41256758-41271296 | 0.0017143 | 0.014 | 26.51 | 39.65 | 1.53 |
| CCDC56 | 17:40949652-40950704 | 0.0004162 | 0.008 | 30.80 | 45.91 | 1.53 |
| CD3D | 11:118209668-118213459 | 0.0006359 | 0.009 | 138.27 | 209.34 | 1.53 |
| SKIV2L | HSCHR6_MHC_QBL:31815829-32011521 | 0.003938 | 0.021 | 5.46 | 8.19 | 1.53 |
| ANXA2 | 15:60639332-60695082 | 0.0016516 | 0.014 | 223.27 | 329.76 | 1.53 |
| TIMM50 | 19:39971051-39981528 | 0.0023604 | 0.016 | 10.11 | 14.99 | 1.53 |
| TIMM17B | X:48750729-48760420 | 0.0014858 | 0.013 | 50.83 | 76.80 | 1.53 |
| C16orf7 | 16:89773541-89883065 | 0.0058883 | 0.027 | 17.15 | 26.15 | 1.53 |
| RP11-158M2.3.1 | 15:85734668-86293512 | 0.0109875 | 0.039 | 3.07 | 4.39 | 1.53 |
| SLX1A,SULT1A3,SULT1A3.1 | 16:30204254-30215631 | 0.0010164 | 0.011 | 63.45 | 94.34 | 1.53 |
| UBR4 | 1:19400999-19536770 | 0.0009869 | 0.011 | 51.45 | 77.73 | 1.52 |
| ARHGDIA | 17:79825597-79829282 | 0.0006448 | 0.009 | 131.84 | 197.46 | 1.52 |
| ZFPM1 | 16:88519724-88601572 | 0.011262 | 0.04 | 2.33 | 3.51 | 1.52 |
| HLA-F | HSCHR6_MHC_SSTO:29687513-29713718 | 0.004572 | 0.023 | 56.00 | 89.52 | 1.52 |
| CD3E | 11:118175259-118186890 | 0.0020318 | 0.015 | 174.09 | 264.07 | 1.52 |
| DGKQ | 4:952674-998316 | 0.0038283 | 0.021 | 5.07 | 7.78 | 1.52 |
| KIAA0664 | 17:2592679-2614927 | 0.0079778 | 0.032 | 3.35 | 5.05 | 1.52 |
| CABIN1 | 22:24407641-24574596 | 0.0080303 | 0.032 | 56.03 | 84.66 | 1.52 |
| BANF1 | 11:65764015-65771620 | 0.0001893 | 0.006 | 72.84 | 108.89 | 1.52 |
| MRPL1 | 4:78783673-78882347 | 0.0050039 | 0.024 | 8.96 | 13.31 | 1.52 |
| APOA1BP | 1:156561541-156564091 | 0.0000284 | 0.004 | 24.98 | 37.91 | 1.52 |
| SUPT16H | 14:21819630-21852425 | 0.0001071 | 0.005 | 13.68 | 20.65 | 1.52 |
| ELP4 | 11:31531296-31805546 | 0.000787 | 0.01 | 4.59 | 6.95 | 1.52 |
| ETHE1 | 19:44010871-44040282 | 0.0037504 | 0.021 | 28.04 | 41.57 | 1.52 |
| RP11-1167A19.14.1,SF3B2 | 11:65818199-65837090 | 0.0003918 | 0.008 | 58.42 | 87.71 | 1.52 |
| HTATSF1 | X:135579237-135594505 | 0.001003 | 0.011 | 7.35 | 10.94 | 1.52 |
| ADCK5 | 8:145596789-145634753 | 0.0084228 | 0.033 | 1.41 | 2.03 | 1.52 |
| PFN1 | 17:4848946-4860426 | 0.0003944 | 0.008 | 1402.63 | 2118.37 | 1.52 |
| RARRES3 | 11:63304280-63313934 | 0.0002881 | 0.007 | 127.51 | 193.00 | 1.52 |
| DNAJC30 | 7:73096600-73134017 | 0.0019992 | 0.015 | 4.37 | 6.44 | 1.52 |
| AC138035.1 | 5:180748651-180755068 | 0.007269 | 0.03 | 79.36 | 120.69 | 1.52 |
| ANKS3 | 16:4746512-4817219 | 0.0016793 | 0.014 | 3.01 | 4.45 | 1.51 |
| OSBPL5 | 11:3108345-3187969 | 0.0006245 | 0.009 | 10.46 | 15.86 | 1.51 |
| VARS | 6:31745294-31763730 | 0.0008883 | 0.01 | 1.31 | 2.02 | 1.51 |
| STX8 | 17:9153788-9479275 | 0.0000398 | 0.005 | 33.15 | 49.61 | 1.51 |
| RPS26 | 12:56435636-56438116 | 0.0009282 | 0.011 | 429.82 | 669.61 | 1.51 |
| MTFP1,RP4-539M6.19.1,SEC14L2 | 22:30279143-31530682 | 0.0003132 | 0.007 | 7.68 | 11.66 | 1.51 |
| SPAG7 | 17:4862522-4871132 | 0.0007102 | 0.009 | 31.07 | 46.31 | 1.51 |
| DDX41 | 5:176938577-176944470 | 0.0002389 | 0.007 | 28.99 | 43.19 | 1.51 |
| TSEN34 | 19:54693788-54697585 | 0.0050034 | 0.024 | 110.31 | 167.55 | 1.51 |
| LMF1,RP11-161M6.2.1 | 16:903633-1031596 | 0.0109324 | 0.039 | 7.38 | 10.33 | 1.51 |
| DNAJB5 | 9:34989637-34998897 | 0.0099299 | 0.037 | 1.03 | 1.55 | 1.51 |
| SIRT6 | 19:4174105-4182596 | 0.0029295 | 0.018 | 12.24 | 18.18 | 1.51 |
| AL021707.1,AL021707.2,GTPBP1 | 22:39101727-39190203 | 0.0001059 | 0.005 | 24.44 | 36.75 | 1.51 |
| GAA | 17:78075354-78093678 | 0.0049215 | 0.024 | 24.51 | 36.59 | 1.51 |
| NUBP1 | 16:10837677-10912621 | 0.0003324 | 0.007 | 18.59 | 27.48 | 1.51 |
| ALDOA | 16:30064410-30081778 | 0.0028986 | 0.018 | 918.85 | 1383.78 | 1.51 |
| PPIB | 15:64443913-64455404 | 0.0000139 | 0.003 | 230.05 | 345.41 | 1.51 |
| PRRC2A | 6:31588449-31605548 | 0.0061838 | 0.027 | 6.45 | 9.66 | 1.51 |
| LY6G5C | HSCHR6_MHC_MANN:31668498-31859776 | 0.0146588 | 0.047 | 0.91 | 1.26 | 1.50 |
| NDUFAF3 | 3:48955220-49060905 | 0.0052277 | 0.025 | 45.79 | 66.32 | 1.50 |
| NR2C2AP | 19:19256375-19314220 | 0.0001209 | 0.006 | 8.29 | 12.29 | 1.50 |
| SIPA1 | 11:65405567-65418401 | 0.0089287 | 0.034 | 101.83 | 148.60 | 1.50 |
| MRP63 | 13:21750783-21753223 | 0.0081468 | 0.032 | 7.13 | 10.29 | 1.50 |
| MCRS1 | 12:49932939-49961936 | 0.0006499 | 0.009 | 29.59 | 43.80 | 1.50 |
| C12orf45 | 12:105380087-105478355 | 0.003455 | 0.02 | 2.87 | 4.15 | 1.50 |
| LSM7 | 19:2321521-2328614 | 0.0030885 | 0.019 | 51.07 | 76.11 | 1.50 |
| FUT7 | 9:139901685-139931234 | 0.0073908 | 0.031 | 26.06 | 39.42 | 1.50 |
| HDHD3 | 9:116135698-116139279 | 0.0075833 | 0.031 | 6.35 | 9.30 | 1.50 |
| PSMC4 | 19:40477072-40487351 | 0.0006707 | 0.009 | 32.70 | 48.30 | 1.50 |
| DHX32 | 10:127512114-127585005 | 0.0030257 | 0.018 | 2.48 | 3.62 | 1.50 |
| SKAP1 | 17:46210801-46507594 | 0.0044043 | 0.022 | 29.44 | 44.43 | 1.50 |
| TSEN54 | 17:73511787-73520820 | 0.0026403 | 0.017 | 13.32 | 20.64 | 1.50 |
| RP11-252A24.7.1 | 16:74481324-74483790 | 0.0031768 | 0.019 | 7.12 | 10.44 | 1.50 |
| MIR639,TECR | 19:14640354-14676791 | 0.0000531 | 0.005 | 44.32 | 66.52 | 1.50 |
| PSMB3 | 17:36909002-36920483 | 0.0020243 | 0.015 | 265.99 | 399.55 | 1.50 |
| DAPK3 | 19:3958451-3971038 | 0.0005045 | 0.008 | 13.64 | 20.38 | 1.50 |
| MGST3 | 1:165600097-165631033 | 0.0000227 | 0.004 | 52.07 | 78.28 | 1.50 |
| HLA-B | HSCHR6_MHC_MCF:31398031-31401418 | 0.0019223 | 0.015 | 2033.27 | 3063.54 | 1.50 |
| ARHGAP9 | 12:57853917-57914300 | 0.0037545 | 0.021 | 379.87 | 571.99 | 1.50 |
| NDUFB10 | 16:2009508-2011976 | 0.0003784 | 0.007 | 89.39 | 132.97 | 1.50 |
| RP11-46F15.2.1 | 1:3539753-3541307 | 0.0082646 | 0.033 | 2.49 | 3.69 | 1.50 |
| DAK,RP11-286N22.3.1 | 11:61066922-61136683 | 0.0049056 | 0.024 | 11.64 | 16.60 | 1.50 |
| LINC00152 | 2:87754886-87906324 | 0.0052524 | 0.025 | 72.40 | 109.34 | 1.50 |
| RP11-521B24.5.1 | 14:105864915-105937066 | 0.0020898 | 0.015 | 10.00 | 14.98 | 1.50 |
| AC103810.1 | 17:62796900-62833243 | 0.0016732 | 0.014 | 9.87 | 6.53 | -1.50 |
| MSL2 | 3:135867763-135916083 | 0.0001731 | 0.006 | 20.59 | 13.72 | -1.50 |
| SLC23A2 | 20:4833001-4990939 | 0.0020175 | 0.015 | 6.83 | 4.47 | -1.50 |
| TMOD2 | 15:52015207-52108565 | 0.0000163 | 0.004 | 6.08 | 4.02 | -1.50 |
| TOPBP1 | 3:133317018-133380737 | 0.0008574 | 0.01 | 10.55 | 7.00 | -1.50 |
| NAB1 | 2:191511471-191573442 | 0.000245 | 0.007 | 5.76 | 3.81 | -1.50 |
| CCDC126 | 7:23636997-23684327 | 0.0016152 | 0.013 | 6.49 | 4.30 | -1.50 |
| USP6NL | 10:11502508-11653753 | 0.0105602 | 0.038 | 1.09 | 0.73 | -1.50 |
| TP53INP1 | 8:95825538-96128683 | 0.0000846 | 0.005 | 24.93 | 16.59 | -1.50 |
| CTSS | 1:150702550-150738433 | 0.0023468 | 0.016 | 1010.94 | 671.68 | -1.50 |
| CEP19 | 3:196366556-196462878 | 0.0148256 | 0.047 | 18.67 | 12.23 | -1.50 |
| SSFA2 | 2:182756559-182795465 | 0.0000778 | 0.005 | 21.12 | 14.05 | -1.50 |
| IRAK4 | 12:44152746-44183346 | 0.0005527 | 0.009 | 20.40 | 13.40 | -1.50 |
| EPHX2 | 8:27348295-27403081 | 0.013116 | 0.044 | 6.85 | 4.29 | -1.51 |
| OGFRL1 | 6:71961060-72037787 | 0.0001429 | 0.006 | 37.11 | 24.76 | -1.51 |
| RANBP6 | 9:6011042-6015618 | 0.0001163 | 0.006 | 4.29 | 2.88 | -1.51 |
| GS1-251I9.4.1 | 8:92072136-92082417 | 0.0007058 | 0.009 | 4.51 | 2.95 | -1.51 |
| UBL3 | 13:30338507-30424821 | 0.0002956 | 0.007 | 14.40 | 9.55 | -1.51 |
| PCNP | 3:101292938-101313281 | 0.0002763 | 0.007 | 44.12 | 28.94 | -1.51 |
| NUPL1 | 13:25875661-25923938 | 0.000296 | 0.007 | 11.56 | 7.62 | -1.51 |
| CMTM8 | 3:32280170-32411817 | 0.0042697 | 0.022 | 3.70 | 2.40 | -1.51 |
| ZNF740 | 12:53551447-53583208 | 0.0014558 | 0.013 | 9.63 | 6.40 | -1.51 |
| ZNF25 | 10:38238499-38265561 | 0.0020227 | 0.015 | 2.25 | 1.50 | -1.51 |
| ZNF552 | 19:58280996-58326281 | 0.0018509 | 0.014 | 7.94 | 5.23 | -1.51 |
| AC013400.2.1 | 2:20068620-20084808 | 0.0033642 | 0.019 | 2.50 | 1.66 | -1.51 |
| GPR34 | X:41374186-41782716 | 0.0031667 | 0.019 | 4.98 | 3.19 | -1.51 |
| PPIF | 10:81107224-81115093 | 0.0034685 | 0.02 | 55.62 | 35.80 | -1.51 |
| PIGF | 2:46717888-46852881 | 0.0001707 | 0.006 | 37.91 | 24.79 | -1.51 |
| C9orf30,C9orf30-TMEFF1,TMEFF1 | 9:103189437-103339918 | 0.0020614 | 0.015 | 4.56 | 2.93 | -1.51 |
| MAPK9 | 5:179660142-179719099 | 0.001929 | 0.015 | 6.80 | 4.46 | -1.51 |
| ZNF675 | 19:23835707-23870017 | 0.0016475 | 0.014 | 1.83 | 1.24 | -1.51 |
| EXOC6B | 2:72403112-73053177 | 0.0033327 | 0.019 | 1.12 | 0.73 | -1.51 |
| TGDS | 13:95226307-95248511 | 0.0005288 | 0.009 | 4.44 | 2.95 | -1.51 |
| TCFL5 | 20:61447595-61493115 | 0.0027884 | 0.018 | 4.78 | 3.04 | -1.51 |
| SNX13 | 7:17832465-17980130 | 0.0000814 | 0.005 | 7.26 | 4.77 | -1.52 |
| ZNF169 | 9:97021592-97063736 | 0.0001167 | 0.006 | 2.98 | 1.93 | -1.52 |
| YIPF5 | 5:143537722-143550278 | 0.002989 | 0.018 | 12.59 | 8.08 | -1.52 |
| RAB21 | 12:72148653-72184699 | 0.0015765 | 0.013 | 18.08 | 11.84 | -1.52 |
| ZNF439 | 19:11959575-11980306 | 0.0140067 | 0.046 | 1.88 | 1.17 | -1.52 |
| PURB | 7:44915895-44924960 | 0.0000299 | 0.004 | 7.38 | 4.83 | -1.52 |
| NSUN6,RP11-139J15.2.1,RP11-499P20.2.1 | 10:18429605-18948196 | 0.0121603 | 0.042 | 5.17 | 3.41 | -1.52 |
| RP11-830F9.5.1 | 16:88941265-89043612 | 0.0056759 | 0.026 | 3.18 | 2.02 | -1.52 |
| RP11-158K1.3.1 | 8:125500725-125740730 | 0.0086331 | 0.033 | 1.25 | 0.78 | -1.52 |
| RP11-59D5__B.2.1 | 6:64345724-66417118 | 0.0046919 | 0.023 | 4.63 | 3.03 | -1.52 |
| KLF4 | 9:110247132-110252763 | 0.0007102 | 0.009 | 12.92 | 8.28 | -1.52 |
| YPEL2 | 17:57409052-57479095 | 0.0005483 | 0.009 | 7.80 | 5.14 | -1.52 |
| LRCH1 | 13:47127302-47325710 | 0.002637 | 0.017 | 1.76 | 1.13 | -1.52 |
| DMXL2 | 15:51706293-51915030 | 0.0017196 | 0.014 | 30.32 | 19.55 | -1.52 |
| PWWP2A | 5:159436119-159546430 | 0.0092129 | 0.035 | 6.43 | 4.12 | -1.52 |
| C5orf4 | 5:154198050-154256353 | 0.0019454 | 0.015 | 20.02 | 12.51 | -1.52 |
| ZNF185 | X:152082985-152142025 | 0.0025588 | 0.017 | 18.37 | 12.31 | -1.53 |
| ING3 | 7:120590802-120617270 | 0.0003393 | 0.007 | 10.71 | 7.00 | -1.53 |
| SNX30 | 9:115513117-115637267 | 0.0003578 | 0.007 | 4.20 | 2.73 | -1.53 |
| RP11-196G18.22.1 | 1:149816064-149820591 | 0.0051121 | 0.024 | 6.28 | 4.02 | -1.53 |
| PPM1A | 14:60706838-60765805 | 0.0056928 | 0.026 | 84.94 | 52.51 | -1.53 |
| HP1BP3 | 1:21069153-21113816 | 0.0014927 | 0.013 | 34.04 | 22.31 | -1.53 |
| BRWD3 | X:79926352-80065187 | 0.0002027 | 0.006 | 3.90 | 2.55 | -1.53 |
| RP11-1H15.1.1,SBF2 | 11:9776316-10315754 | 0.0042835 | 0.022 | 8.70 | 5.63 | -1.53 |
| RLF | 1:40627044-40706593 | 0.0000612 | 0.005 | 4.99 | 3.27 | -1.53 |
| EXOC8 | 1:231468479-231490769 | 0.0002714 | 0.007 | 5.22 | 3.40 | -1.53 |
| AGAP8 | 10:51224676-51252947 | 0.0035624 | 0.02 | 5.88 | 3.76 | -1.53 |
| CDC42EP3 | 2:37827278-37965611 | 0.0022881 | 0.016 | 50.12 | 32.54 | -1.53 |
| TMBIM1 | 2:219135114-219232822 | 3.10E-06 | 0.003 | 132.63 | 86.44 | -1.53 |
| CSNK1G3 | 5:122847792-122952739 | 0.0009608 | 0.011 | 5.05 | 3.24 | -1.53 |
| HERC3 | 4:89442135-89629693 | 0.0015623 | 0.013 | 12.35 | 8.09 | -1.53 |
| GCNT1 | 9:79034751-79122332 | 0.0028194 | 0.018 | 3.34 | 2.21 | -1.53 |
| STRN3 | 14:31343719-31562818 | 0.0038263 | 0.021 | 3.46 | 2.13 | -1.53 |
| RNF138 | 18:29671827-29711524 | 0.0003498 | 0.007 | 15.55 | 10.20 | -1.53 |
| EEF2K | 16:22217602-22300059 | 0.0025889 | 0.017 | 1.98 | 1.25 | -1.53 |
| NPTN | 15:73735498-73926475 | 0.0010346 | 0.011 | 53.05 | 34.55 | -1.54 |
| TASP1 | 20:13202417-13619587 | 0.0040841 | 0.022 | 1.84 | 1.21 | -1.54 |
| ZNF333 | 19:14800612-14889353 | 0.0001045 | 0.005 | 5.87 | 3.78 | -1.54 |
| CRK | 17:1323982-1359552 | 0.0000186 | 0.004 | 13.67 | 8.91 | -1.54 |
| RAB11FIP2 | 10:119764426-119969663 | 0.0043695 | 0.022 | 1.87 | 1.17 | -1.54 |
| AFF1 | 4:87797357-88062206 | 0.0007329 | 0.01 | 7.98 | 5.12 | -1.54 |
| GXYLT2,PPP4R2 | 3:72937223-73118350 | 0.0000238 | 0.004 | 13.68 | 8.84 | -1.54 |
| COX6A1P2 | 6:37012606-37013177 | 0.0157675 | 0.049 | 6.89 | 4.04 | -1.54 |
| RP2 | X:46696374-46741793 | 0.0017309 | 0.014 | 19.83 | 12.47 | -1.54 |
| DUS4L | 7:106809405-107218906 | 0.0001019 | 0.005 | 2.03 | 1.32 | -1.54 |
| HERC2P9 | 15:28834637-28930410 | 0.0083478 | 0.033 | 3.04 | 1.90 | -1.54 |
| PIK3CG | 7:106505722-106547590 | 0.0060844 | 0.027 | 9.37 | 6.25 | -1.54 |
| RP11-202A13.2.1,RYK | 3:133784146-133969689 | 0.0007394 | 0.01 | 9.41 | 6.12 | -1.54 |
| CCDC13,HHATL,HIGD1A | 3:42734154-42846023 | 0.0001186 | 0.006 | 37.60 | 24.19 | -1.54 |
| RP11-285G1.8.1,RP11-445N18.2.1 | 10:45493145-45567036 | 0.0022227 | 0.016 | 3.37 | 2.20 | -1.54 |
| MARCH6 | 5:10353814-10435491 | 0.0007151 | 0.01 | 25.82 | 16.47 | -1.54 |
| AC079630.4.1 | 12:40579810-40763087 | 0.0087702 | 0.034 | 3.63 | 2.25 | -1.54 |
| PIP5K1A | 1:151170424-151222012 | 0.0024407 | 0.016 | 11.07 | 7.17 | -1.54 |
| CSF2RB | 22:37309669-37336491 | 0.0003699 | 0.007 | 137.16 | 88.29 | -1.54 |
| CCDC93 | 2:118617002-118771709 | 0.0102935 | 0.037 | 9.27 | 6.02 | -1.54 |
| RP1-111D6.3.1,TAB2 | 6:149539061-149732749 | 0.0000923 | 0.005 | 29.60 | 19.23 | -1.54 |
| FAM179B | 14:45397671-45543634 | 0.0135756 | 0.045 | 1.48 | 0.96 | -1.55 |
| ANGEL1 | 14:77253587-77292589 | 0.0128715 | 0.043 | 3.52 | 2.19 | -1.55 |
| ZBTB11 | 3:101367732-101396431 | 0.0010826 | 0.011 | 4.43 | 2.87 | -1.55 |
| IL6ST | 5:55230922-55290772 | 0.0000991 | 0.005 | 7.42 | 4.74 | -1.55 |
| GRPEL2 | 5:148724992-148737205 | 0.0000817 | 0.005 | 3.66 | 2.39 | -1.55 |
| THAP1 | 8:42691816-42698468 | 0.0004574 | 0.008 | 7.25 | 4.64 | -1.55 |
| EIF4A2,SNORA63,SNORA81,snR39B | 3:186500993-186524847 | 0.0024338 | 0.016 | 129.26 | 79.05 | -1.55 |
| EDEM1 | 3:5229330-5261642 | 0.0002599 | 0.007 | 18.01 | 11.59 | -1.55 |
| ZNF440 | 19:11925098-11946016 | 0.0040191 | 0.021 | 1.80 | 1.16 | -1.55 |
| CD84 | 1:160506862-160549306 | 0.0012704 | 0.012 | 4.78 | 3.08 | -1.55 |
| SETD6 | 16:58549382-58663790 | 0.0007389 | 0.01 | 2.84 | 1.81 | -1.55 |
| ATXN7 | 3:63850232-63989138 | 0.0022118 | 0.016 | 8.95 | 5.70 | -1.55 |
| C21orf67 | 21:46352728-46359828 | 0.014701 | 0.047 | 3.45 | 2.17 | -1.55 |
| EIF4G2,SNORD97 | 11:10804859-10830657 | 0.0049446 | 0.024 | 241.09 | 152.43 | -1.55 |
| TBC1D4 | 13:75858807-76056250 | 0.0028769 | 0.018 | 7.77 | 4.82 | -1.55 |
| ZNF844 | 19:12175513-12192380 | 0.0039197 | 0.021 | 1.44 | 0.92 | -1.55 |
| PAPOLG | 2:60983364-61029220 | 0.0010548 | 0.011 | 5.29 | 3.44 | -1.55 |
| AC027323.1,LYSMD3 | 5:89811427-90460038 | 0.0030704 | 0.018 | 6.69 | 4.19 | -1.55 |
| KIAA0247 | 14:70078312-70181859 | 0.0010099 | 0.011 | 38.62 | 24.77 | -1.55 |
| CBL | 11:119076751-119177651 | 0.00019 | 0.006 | 9.36 | 6.04 | -1.55 |
| ANGEL2 | 1:213165523-213189168 | 0.000291 | 0.007 | 9.10 | 5.82 | -1.55 |
| ZNF431 | 19:21324839-21368805 | 0.001693 | 0.014 | 1.99 | 1.26 | -1.56 |
| PRKY,U6 | Y:7142012-7249589 | 0.0153038 | 0.048 | 6.07 | 4.40 | -1.56 |
| SENP5 | 3:196594726-196661585 | 4.53E-06 | 0.003 | 10.40 | 6.65 | -1.56 |
| TTC39B | 9:15170842-15307358 | 0.0047164 | 0.023 | 2.33 | 1.50 | -1.56 |
| APPL2 | 12:105567073-105789875 | 0.000337 | 0.007 | 22.12 | 13.97 | -1.56 |
| MDM4 | 1:204485510-204542871 | 0.0000555 | 0.005 | 13.06 | 8.28 | -1.56 |
| ATP6V0A2 | 12:124196864-124245549 | 0.0007171 | 0.01 | 8.22 | 5.18 | -1.56 |
| NBPF14 | 1:148003641-148025863 | 0.0000575 | 0.005 | 9.67 | 6.21 | -1.56 |
| MMD | 17:53469973-53499341 | 0.0073413 | 0.03 | 10.73 | 6.89 | -1.56 |
| C8orf83 | 8:93895211-94029901 | 0.0053768 | 0.025 | 6.13 | 3.92 | -1.56 |
| TMEM65 | 8:125324230-125384933 | 0.0014043 | 0.013 | 3.92 | 2.47 | -1.56 |
| ARPC5 | 1:183592400-183604892 | 0.0000163 | 0.004 | 412.70 | 264.25 | -1.56 |
| ZNF107 | 7:64126500-64171404 | 0.0108434 | 0.039 | 1.97 | 1.16 | -1.56 |
| UFM1 | 13:38923985-38937140 | 0.0027886 | 0.018 | 18.33 | 11.56 | -1.56 |
| AC024560.3.1 | 3:197305083-197354777 | 0.0040881 | 0.022 | 5.00 | 3.17 | -1.56 |
| LA16c-358B7.3.1 | 16:1355547-1377019 | 0.0069173 | 0.029 | 2.17 | 1.31 | -1.56 |
| TET2 | 4:106067031-106200973 | 0.0000377 | 0.005 | 7.67 | 4.90 | -1.56 |
| UBE2J1 | 6:90036343-90062567 | 0.0000829 | 0.005 | 42.57 | 27.26 | -1.56 |
| CMPK1 | 1:47799468-47844511 | 0.0000379 | 0.005 | 39.24 | 25.18 | -1.57 |
| SNN | 16:11762269-11836704 | 0.0057574 | 0.026 | 107.60 | 67.40 | -1.57 |
| RP4-550H1.6.1 | 20:34633543-34638882 | 0.0001116 | 0.006 | 32.00 | 20.47 | -1.57 |
| ANKRD44 | 2:197831740-198175897 | 0.0142533 | 0.046 | 43.31 | 24.99 | -1.57 |
| TNFAIP3 | 6:138178422-138204449 | 0.0016561 | 0.014 | 13.47 | 8.49 | -1.57 |
| C11orf54 | 11:93394804-93547861 | 0.0003632 | 0.007 | 12.04 | 7.62 | -1.57 |
| SLC25A24 | 1:108676657-108744113 | 0.011324 | 0.04 | 6.94 | 4.20 | -1.57 |
| CD46 | 1:207925401-207968858 | 0.0002266 | 0.007 | 119.63 | 75.51 | -1.57 |
| LTN1 | 21:30300465-30365277 | 0.0102506 | 0.037 | 2.46 | 1.49 | -1.57 |
| TRIM52 | 5:180663908-180699308 | 0.0000595 | 0.005 | 7.78 | 4.96 | -1.57 |
| CYSLTR1 | X:77526960-77583048 | 0.0003815 | 0.007 | 12.95 | 8.06 | -1.57 |
| KLHL5 | 4:39046450-39128477 | 0.0008306 | 0.01 | 6.72 | 4.25 | -1.57 |
| JKAMP | 14:59895739-60043549 | 0.000017 | 0.004 | 31.13 | 19.81 | -1.57 |
| EGLN1 | 1:231499496-231560790 | 0.0004522 | 0.008 | 70.75 | 45.25 | -1.57 |
| FLI1 | 11:128551134-128683161 | 0.0007255 | 0.01 | 69.28 | 44.37 | -1.57 |
| ZNF641 | 12:48733790-48836730 | 0.0008019 | 0.01 | 20.06 | 12.97 | -1.57 |
| HIAT1 | 1:100503652-100598511 | 0.0009371 | 0.011 | 22.55 | 14.36 | -1.57 |
| ANKRD12 | 18:9136757-9285206 | 0.0000512 | 0.005 | 5.45 | 3.46 | -1.57 |
| ZBTB39 | 12:57392617-57400230 | 0.0046906 | 0.023 | 1.22 | 0.78 | -1.57 |
| RP11-344B5.2.1 | 9:132044736-132048007 | 0.0038735 | 0.021 | 15.41 | 10.22 | -1.57 |
| KIAA0664L3 | 16:31711910-31723305 | 0.0036808 | 0.02 | 4.84 | 3.00 | -1.57 |
| PDE3B | 11:14665268-14892350 | 0.0028648 | 0.018 | 7.54 | 4.85 | -1.57 |
| TAF13 | 1:109605120-109618624 | 0.0009833 | 0.011 | 14.65 | 9.23 | -1.57 |
| SRGN | 10:70847861-70864567 | 0.000851 | 0.01 | 2186.82 | 1397.30 | -1.58 |
| C6orf105 | 6:11712286-11807279 | 0.0149643 | 0.048 | 5.66 | 3.44 | -1.58 |
| TET3 | 2:74229839-74329698 | 0.0047171 | 0.023 | 1.39 | 0.87 | -1.58 |
| SPDYE6 | 7:102122891-102312182 | 0.0072543 | 0.03 | 1.12 | 0.69 | -1.58 |
| NUDT16P.1 | 3:130569438-131100319 | 0.0008464 | 0.01 | 5.22 | 3.27 | -1.58 |
| GTF2H3 | 12:124118374-124146479 | 0.000335 | 0.007 | 3.79 | 2.42 | -1.58 |
| SLC25A15 | 13:41363547-41386596 | 0.0043392 | 0.022 | 1.95 | 1.25 | -1.58 |
| SNX16 | 8:82704057-82755101 | 0.0048863 | 0.024 | 2.53 | 1.57 | -1.58 |
| DNAAF2 | 14:50091891-50101948 | 0.0089055 | 0.034 | 2.06 | 1.30 | -1.58 |
| BRD2 | HSCHR6_MHC_MCF:32935859-32980443 | 0.0033263 | 0.019 | 8.68 | 5.45 | -1.58 |
| RAB5A | 3:19988570-20053822 | 0.0001456 | 0.006 | 26.26 | 16.52 | -1.58 |
| ZNF674 | X:46357161-46404892 | 0.0002679 | 0.007 | 1.47 | 0.92 | -1.58 |
| CMTM6 | 3:32433162-32544900 | 5.84E-06 | 0.003 | 184.69 | 116.87 | -1.58 |
| STXBP5 | 6:147163701-147708707 | 0.0007341 | 0.01 | 7.02 | 4.49 | -1.58 |
| RP11-290F20.2.1 | 20:48909256-48937879 | 0.0006162 | 0.009 | 4.66 | 2.90 | -1.58 |
| SDPR | 2:192699027-193060435 | 0.0044923 | 0.023 | 14.90 | 9.46 | -1.58 |
| STAG3L3 | 7:72440226-72555749 | 0.0029666 | 0.018 | 6.44 | 3.89 | -1.59 |
| MTMR12 | 5:32227099-32313115 | 0.0018892 | 0.014 | 5.18 | 3.34 | -1.59 |
| RAP2C | X:131337052-131623996 | 0.0004995 | 0.008 | 26.34 | 16.25 | -1.59 |
| ZNF396 | 18:32946660-32957301 | 0.0069638 | 0.029 | 1.51 | 0.94 | -1.59 |
| ZFP36L2 | 2:43393799-43823185 | 0.0006968 | 0.009 | 122.90 | 77.96 | -1.59 |
| ZNF619 | 3:40518603-40531727 | 0.0056277 | 0.026 | 1.74 | 1.10 | -1.59 |
| SLC35A5 | 3:112251355-112304424 | 0.0002306 | 0.007 | 16.96 | 10.76 | -1.59 |
| TLR1,TLR6 | 4:38792297-38858438 | 0.0001753 | 0.006 | 84.59 | 53.08 | -1.59 |
| SATB1,TBC1D5 | 3:17199898-18568819 | 0.0013379 | 0.012 | 16.37 | 10.32 | -1.59 |
| NFATC2IP | 16:28962127-28978418 | 0.0037982 | 0.021 | 19.78 | 12.03 | -1.59 |
| EPT1 | 2:26531040-26618759 | 0.0075578 | 0.031 | 1.29 | 0.79 | -1.59 |
| MFN1 | 3:179065479-179112719 | 0.0004357 | 0.008 | 8.79 | 5.36 | -1.59 |
| ZNF445 | 3:44481261-44519162 | 0.0040265 | 0.021 | 1.62 | 0.97 | -1.59 |
| ZNF430 | 19:21203425-21242852 | 0.0001596 | 0.006 | 2.15 | 1.35 | -1.60 |
| BTAF1 | 10:93683821-93790082 | 0.0000514 | 0.005 | 8.45 | 5.19 | -1.60 |
| CSGALNACT2 | 10:43633933-43680756 | 0.002351 | 0.016 | 18.20 | 11.08 | -1.60 |
| RP11-137L10.6.1 | 10:75196185-75385711 | 0.0007162 | 0.01 | 3.20 | 1.99 | -1.60 |
| PDPR | 16:70147528-70221264 | 0.0020834 | 0.015 | 3.89 | 2.41 | -1.60 |
| DCBLD1 | 6:117609462-117923691 | 0.0153391 | 0.048 | 1.33 | 0.76 | -1.60 |
| SLFN13 | 17:33762114-33775856 | 0.0003996 | 0.008 | 4.87 | 2.89 | -1.60 |
| ZFP36L1 | 14:69254376-69263190 | 0.0001491 | 0.006 | 134.84 | 84.01 | -1.60 |
| PDPK1 | 16:2587964-2653189 | 0.0036609 | 0.02 | 12.43 | 7.89 | -1.60 |
| IFNAR1 | 21:34696733-34732168 | 0.0006841 | 0.009 | 33.18 | 20.60 | -1.60 |
| RG9MTD3 | 9:37510888-38069210 | 0.0011757 | 0.012 | 6.07 | 3.80 | -1.60 |
| SYNGAP1 | HSCHR6_MHC_SSTO:33527234-33560852 | 0.0075922 | 0.031 | 1.85 | 1.15 | -1.60 |
| SORD | 15:45315301-45369383 | 0.0034951 | 0.02 | 9.02 | 5.39 | -1.61 |
| ZNF626 | 19:20802744-20844406 | 0.0051262 | 0.024 | 2.72 | 1.68 | -1.61 |
| FAM126B | 2:201773695-201950473 | 0.0073884 | 0.031 | 3.58 | 2.20 | -1.61 |
| TMEM188 | 16:50058320-50070999 | 0.0003411 | 0.007 | 27.66 | 17.04 | -1.61 |
| GLRX | 5:95036952-95160087 | 0.0001377 | 0.006 | 255.48 | 158.60 | -1.61 |
| PRRC2C | 1:171454650-171562650 | 0.003775 | 0.021 | 28.85 | 17.29 | -1.61 |
| HIATL1 | 9:97136832-97223324 | 0.0000821 | 0.005 | 35.14 | 21.72 | -1.61 |
| WSB1 | 17:25621105-25640657 | 0.0010885 | 0.011 | 43.78 | 26.34 | -1.61 |
| SLC39A10 | 2:196440700-196602426 | 0.0020853 | 0.015 | 5.57 | 3.38 | -1.61 |
| POLK | 5:74664310-74896969 | 0.0002806 | 0.007 | 2.14 | 1.31 | -1.61 |
| NIPAL2,U6 | 8:99202060-99306621 | 0.0001636 | 0.006 | 4.44 | 2.68 | -1.61 |
| LMTK2 | 7:97736196-97838945 | 0.0002475 | 0.007 | 3.06 | 1.91 | -1.61 |
| STARD4 | 5:110831730-111353006 | 0.0060919 | 0.027 | 1.95 | 1.15 | -1.62 |
| CXorf38 | X:40488284-40506819 | 0.000375 | 0.007 | 12.18 | 7.49 | -1.62 |
| RP11-1334A24.4.1 | 5:176829140-176883283 | 0.0001683 | 0.006 | 8.44 | 5.17 | -1.62 |
| YRDC | 1:38268615-38275126 | 0.0000126 | 0.003 | 6.19 | 3.82 | -1.62 |
| PRDX3 | 10:120927214-120938345 | 0.000069 | 0.005 | 65.43 | 40.17 | -1.62 |
| GCET2 | 3:111805181-111852152 | 0.0057247 | 0.026 | 4.49 | 2.79 | -1.62 |
| NEK7 | 1:198126092-198291550 | 0.0021249 | 0.015 | 23.39 | 14.09 | -1.62 |
| DSE,RP1-93H18.1.1,RP3-486I3.7.1 | 6:116575335-116759442 | 0.0007542 | 0.01 | 7.47 | 4.58 | -1.62 |
| GPR160,RP11-379K17.9.1 | 3:169755716-169803191 | 0.0002362 | 0.007 | 28.55 | 17.22 | -1.62 |
| RP11-497D6.4.1 | 6:147163701-147708707 | 0.0064764 | 0.028 | 1.15 | 0.67 | -1.62 |
| ZNF468 | 19:53341785-53360902 | 0.0022069 | 0.016 | 2.92 | 1.77 | -1.63 |
| RP11-122G18.5.1 | 1:161337811-161341754 | 0.0019553 | 0.015 | 1.71 | 1.03 | -1.63 |
| MAPK6 | 15:52121824-52392720 | 0.0010198 | 0.011 | 3.00 | 1.84 | -1.63 |
| ZNF417 | 19:58417141-58427978 | 0.0020591 | 0.015 | 2.14 | 1.28 | -1.63 |
| EIF4E3 | 3:71728439-71805647 | 0.000697 | 0.009 | 40.72 | 24.90 | -1.63 |
| AC004017.1 | 19:58193356-58202072 | 0.0013192 | 0.012 | 1.30 | 0.80 | -1.63 |
| CTD-2008A1.2.1 | 15:45118568-45176978 | 0.0130525 | 0.044 | 2.72 | 1.44 | -1.63 |
| AC003682.1,ZNF550 | 19:58053207-58071231 | 0.0020184 | 0.015 | 1.55 | 0.96 | -1.63 |
| AKAP10 | 17:19807787-19881150 | 0.0000498 | 0.005 | 12.20 | 7.44 | -1.63 |
| HIST2H2BF,RP11-196G18.21.1 | 1:149754226-149783928 | 0.0107378 | 0.039 | 7.54 | 4.40 | -1.63 |
| KCNJ2 | 17:68164813-68176160 | 0.0039337 | 0.021 | 18.78 | 11.50 | -1.63 |
| GNRHR2 | 1:145477084-145516076 | 0.0084377 | 0.033 | 3.81 | 2.07 | -1.64 |
| EIF2C4 | 1:36273772-36323491 | 0.0001104 | 0.006 | 28.06 | 17.11 | -1.64 |
| RP11-327F22.1.1 | 16:50774820-50837249 | 0.007107 | 0.03 | 2.56 | 1.40 | -1.64 |
| PIK3R1 | 5:67511547-67597649 | 0.0008808 | 0.01 | 10.03 | 6.27 | -1.64 |
| ZSWIM1 | 20:44509865-44513905 | 0.0048017 | 0.024 | 4.51 | 2.74 | -1.64 |
| TXK | 4:48068409-48136273 | 0.0015928 | 0.013 | 6.52 | 3.68 | -1.64 |
| E2F3 | 6:20402136-20493945 | 0.0003289 | 0.007 | 9.57 | 5.76 | -1.64 |
| IMPA1 | 8:82570195-82598928 | 0.00421 | 0.022 | 5.97 | 3.48 | -1.64 |
| MLLT11 | 1:151020215-151042801 | 0.0028758 | 0.018 | 4.52 | 2.68 | -1.64 |
| CRADD | 12:94071150-94288616 | 0.0024479 | 0.016 | 10.83 | 6.29 | -1.64 |
| BCL2L13 | 22:18111620-18213388 | 0.0002298 | 0.007 | 20.03 | 11.92 | -1.64 |
| TLR4 | 9:120466609-120479149 | 0.0001018 | 0.005 | 49.35 | 30.02 | -1.64 |
| TNFRSF9 | 1:7979906-8000926 | 0.0115144 | 0.04 | 1.32 | 0.81 | -1.64 |
| MYNN | 3:169490852-169507504 | 0.0002373 | 0.007 | 6.45 | 3.97 | -1.64 |
| CTA-204B4.2.1 | 8:141515911-141518142 | 0.0003016 | 0.007 | 0.99 | 0.60 | -1.64 |
| ZNF561 | 19:9715355-9732075 | 0.0006888 | 0.009 | 7.45 | 4.44 | -1.64 |
| FLVCR1 | 1:213031596-213072705 | 0.0059127 | 0.027 | 3.41 | 2.15 | -1.65 |
| SLCO4C1 | 5:101569689-101632253 | 0.0005219 | 0.009 | 4.27 | 2.60 | -1.65 |
| CCDC125,CFL1P5 | 5:68576001-68628636 | 0.0034803 | 0.02 | 10.34 | 6.02 | -1.65 |
| GATS,GATS.1 | 7:99775185-99869855 | 0.0000764 | 0.005 | 7.00 | 4.22 | -1.65 |
| FAM175A | 4:84377084-84444501 | 0.0006556 | 0.009 | 6.36 | 3.81 | -1.65 |
| GPR157 | 1:9160363-9189250 | 0.006989 | 0.029 | 1.42 | 0.84 | -1.65 |
| RNF11 | 1:51701942-51739127 | 0.0062094 | 0.027 | 49.94 | 31.06 | -1.65 |
| ASB8 | 12:48541570-48574996 | 0.0005484 | 0.009 | 32.81 | 19.71 | -1.65 |
| RP11-368J21.1.1,RP11-368J21.2.1 | 16:22357256-22547842 | 0.0014711 | 0.013 | 26.73 | 15.74 | -1.66 |
| RAP1B | 12:69004618-69054372 | 0.0002727 | 0.007 | 138.75 | 84.14 | -1.66 |
| NRIP1 | 21:16333555-16437321 | 0.0011659 | 0.012 | 1.08 | 0.65 | -1.66 |
| CTDSP2,MIR26A2 | 12:58213709-58240522 | 0.000788 | 0.01 | 105.22 | 63.10 | -1.66 |
| AC091167.2 | 15:90818265-90820841 | 0.0015735 | 0.013 | 1.14 | 0.71 | -1.66 |
| STAG3L2 | 7:74071993-74306729 | 0.0059426 | 0.027 | 5.14 | 2.80 | -1.67 |
| CDK2 | 12:56295196-56367099 | 0.0090137 | 0.034 | 7.14 | 3.90 | -1.67 |
| GK-AS1 | X:30671475-30748725 | 0.0148308 | 0.047 | 3.40 | 2.00 | -1.67 |
| C6orf57 | 6:71276619-71299272 | 0.001628 | 0.013 | 3.82 | 2.25 | -1.67 |
| MYB | 6:135502452-135540311 | 0.0011919 | 0.012 | 2.49 | 1.44 | -1.67 |
| LYPLA1 | 8:54958937-55014577 | 0.0002875 | 0.007 | 30.07 | 17.82 | -1.67 |
| ARHGAP12 | 10:32094364-32217742 | 0.0003298 | 0.007 | 5.33 | 3.23 | -1.67 |
| NR2C2 | 3:14860468-15106842 | 0.0009135 | 0.011 | 3.49 | 2.06 | -1.67 |
| RP11-792D21.2.1 | 4:79567056-79603853 | 0.0007263 | 0.01 | 2.20 | 1.32 | -1.67 |
| C14orf43 | 14:74181824-74269973 | 0.0000722 | 0.005 | 8.04 | 4.80 | -1.67 |
| RINT1 | 7:105172531-105241322 | 0.006568 | 0.028 | 5.26 | 3.07 | -1.67 |
| RMI1 | 9:86595625-86618985 | 0.0000547 | 0.005 | 4.70 | 2.78 | -1.67 |
| BACH1,GRIK1-AS2,LINC00189 | 21:30565800-31312351 | 0.000908 | 0.011 | 38.28 | 22.29 | -1.67 |
| B3GNT2 | 2:62423247-62451866 | 0.0006463 | 0.009 | 8.76 | 5.19 | -1.67 |
| TNFSF4 | 1:173152872-173176452 | 0.0030505 | 0.018 | 1.64 | 0.95 | -1.67 |
| PNRC1 | 6:89790469-89794879 | 0.0002642 | 0.007 | 253.10 | 151.22 | -1.67 |
| UTP23 | 8:117654368-117889123 | 0.0010027 | 0.011 | 5.23 | 3.06 | -1.67 |
| FBXL3 | 13:77564794-77601330 | 0.0000331 | 0.005 | 25.18 | 15.09 | -1.67 |
| ICOS | 2:204801470-204826300 | 0.0011345 | 0.012 | 4.20 | 2.51 | -1.68 |
| PAQR3 | 4:79697495-79860592 | 0.0049245 | 0.024 | 5.78 | 3.35 | -1.68 |
| RP11-463D19.2.1,STAU2,UBE2W | 8:74332238-74791145 | 0.0000107 | 0.003 | 28.53 | 17.07 | -1.68 |
| FCAR | 19:55385548-55401838 | 0.0002717 | 0.007 | 33.55 | 19.93 | -1.68 |
| UBE2D1 | 10:60094734-60129403 | 0.0003114 | 0.007 | 85.35 | 51.30 | -1.68 |
| WHAMMP2 | 15:28947099-29002537 | 0.0096393 | 0.036 | 1.97 | 1.12 | -1.68 |
| DNAJC24 | 11:31391386-31453396 | 0.0117601 | 0.041 | 2.80 | 1.55 | -1.68 |
| SMYD4 | 17:1682828-1733170 | 0.0052004 | 0.025 | 3.23 | 1.88 | -1.68 |
| ZBED4 | 22:50247489-50282090 | 0.0001335 | 0.006 | 2.11 | 1.28 | -1.68 |
| MTF1 | 1:38279850-38325292 | 0.0033897 | 0.019 | 3.16 | 1.87 | -1.68 |
| ZNF92 | 7:64838711-64866038 | 0.0010107 | 0.011 | 1.98 | 1.21 | -1.68 |
| RP11-1319K7.1.1 | 5:69390201-69424261 | 0.0030115 | 0.018 | 13.22 | 7.57 | -1.68 |
| TRIM23 | 5:64885506-65167553 | 0.0054342 | 0.025 | 3.74 | 2.09 | -1.68 |
| RAC1P2 | 4:46725792-46726625 | 0.0138292 | 0.045 | 4.51 | 2.09 | -1.68 |
| RP11-98I9.4.1 | 6:99845926-99879249 | 0.0026985 | 0.017 | 1.47 | 0.87 | -1.69 |
| PHC1 | 12:9066491-9102551 | 0.0014755 | 0.013 | 3.22 | 1.89 | -1.69 |
| L3MBTL1 | 20:42136319-42179590 | 0.0046393 | 0.023 | 2.45 | 1.39 | -1.69 |
| MYSM1 | 1:59120410-59165764 | 0.0005943 | 0.009 | 3.48 | 1.98 | -1.69 |
| AKIRIN1 | 1:39456894-39471731 | 0.0000111 | 0.003 | 41.94 | 24.81 | -1.69 |
| RP11-173P15.5.1 | 12:121148237-121161443 | 0.0028643 | 0.018 | 9.34 | 5.62 | -1.70 |
| TSNAX | 1:231658133-231702270 | 0.0006989 | 0.009 | 14.70 | 8.45 | -1.70 |
| TMCC1 | 3:129366634-129612419 | 0.006356 | 0.028 | 30.22 | 16.80 | -1.70 |
| ZBTB43 | 9:129567284-129600489 | 0.0000149 | 0.004 | 2.19 | 1.30 | -1.70 |
| FPR2 | 19:52264182-52273760 | 0.0001809 | 0.006 | 146.52 | 87.14 | -1.70 |
| AP003068.23.1 | 11:64945076-64979477 | 0.0128888 | 0.043 | 12.04 | 6.45 | -1.70 |
| CTD-2368P22.1.1 | 19:58488420-58522600 | 0.0064431 | 0.028 | 1.34 | 0.79 | -1.70 |
| RIC3 | 11:8040790-8190602 | 0.0048235 | 0.024 | 3.97 | 2.30 | -1.70 |
| DNAJC25,DNAJC25-GNG10,GNG10 | 9:114393631-114432509 | 0.0060099 | 0.027 | 225.94 | 127.46 | -1.70 |
| SFMBT2 | 10:7200585-7453450 | 0.0047118 | 0.023 | 1.59 | 0.96 | -1.71 |
| BCL2L11 | 2:111876954-111924587 | 2.69E-06 | 0.003 | 8.21 | 4.83 | -1.71 |
| PPP4R1L | 20:56806187-56884495 | 0.0009087 | 0.011 | 4.20 | 2.39 | -1.71 |
| LYRM2 | 6:90142888-90348472 | 0.000047 | 0.005 | 8.16 | 4.77 | -1.71 |
| IKZF5 | 10:124690418-124768333 | 0.0001929 | 0.006 | 6.13 | 3.55 | -1.71 |
| POU5F1P3 | 12:8276227-8371507 | 0.0009831 | 0.011 | 5.88 | 3.34 | -1.72 |
| HHEX | 10:94447944-94455403 | 0.0000295 | 0.004 | 30.58 | 17.84 | -1.72 |
| FAM157B | 9:141106636-141149300 | 0.0047441 | 0.024 | 11.87 | 6.62 | -1.72 |
| NEU3 | 11:74699178-74729938 | 0.0062557 | 0.028 | 1.88 | 1.08 | -1.72 |
| NSFP1 | 17:44450220-44564507 | 0.0088114 | 0.034 | 1.50 | 0.81 | -1.72 |
| NT5C2 | 10:104845939-104953056 | 0.0000538 | 0.005 | 66.59 | 38.51 | -1.72 |
| ODC1 | 2:10580093-10588630 | 0.0010423 | 0.011 | 52.37 | 29.56 | -1.72 |
| ZNF267 | 16:31885078-31929914 | 0.0000904 | 0.005 | 11.98 | 6.95 | -1.72 |
| DUSP16 | 12:12628828-12715317 | 0.0067562 | 0.029 | 4.11 | 2.29 | -1.72 |
| P2RY10 | X:78200828-78217451 | 0.0043961 | 0.022 | 14.56 | 8.78 | -1.72 |
| ZBTB26 | 9:125677844-125693779 | 0.0000695 | 0.005 | 2.30 | 1.33 | -1.72 |
| WDR41 | 5:76506273-76916436 | 0.0029872 | 0.018 | 12.74 | 7.16 | -1.72 |
| AGAP10,BMS1P2,RP11-144G6.12.1 | 10:47186202-47243502 | 0.0003109 | 0.007 | 1.38 | 0.80 | -1.73 |
| MEGF9 | 9:123363090-123476748 | 0.0005309 | 0.009 | 69.08 | 39.99 | -1.73 |
| FAM86B1,FAM90A2P | 8:12029710-12053789 | 0.0018591 | 0.014 | 1.25 | 0.68 | -1.73 |
| GLS2 | 12:56843285-56882198 | 0.0009097 | 0.011 | 1.50 | 0.82 | -1.73 |
| CTC-454M9.1.1 | 5:87803362-88762215 | 0.0132196 | 0.044 | 3.94 | 2.25 | -1.73 |
| ERN1 | 17:62120389-62207502 | 0.0004253 | 0.008 | 2.08 | 1.21 | -1.73 |
| RP11-319J24.3.1,ST3GAL6 | 3:98433173-98620533 | 0.0000516 | 0.005 | 24.09 | 13.73 | -1.73 |
| ZNF776 | 19:58258163-58269527 | 0.0004642 | 0.008 | 5.39 | 3.05 | -1.73 |
| AC022596.1.1,CCDC144A | 17:16592850-16719854 | 0.0020894 | 0.015 | 5.18 | 2.94 | -1.73 |
| C10orf10 | 10:45306471-45490172 | 0.0043918 | 0.022 | 5.59 | 3.16 | -1.73 |
| RP11-463J10.3.1 | 14:52292912-52446060 | 0.0004732 | 0.008 | 53.03 | 29.77 | -1.74 |
| RBM12B | 8:94225530-94753245 | 0.0133625 | 0.044 | 2.01 | 1.08 | -1.74 |
| RP11-185E8.1.1 | 3:171185872-171187422 | 0.000342 | 0.007 | 10.17 | 5.78 | -1.74 |
| NEK4 | 3:52744799-52804965 | 0.0015762 | 0.013 | 3.45 | 1.91 | -1.74 |
| PROK2 | 3:71820806-71834357 | 0.0133271 | 0.044 | 202.51 | 119.37 | -1.75 |
| RNF24 | 20:3912067-3996229 | 0.0042165 | 0.022 | 24.59 | 13.79 | -1.75 |
| PRKAB2 | 1:146626684-146644129 | 0.0062788 | 0.028 | 3.70 | 2.12 | -1.75 |
| RP11-572P18.1.1 | 10:122114176-122114718 | 0.0144438 | 0.047 | 164.91 | 77.71 | -1.75 |
| CDK1 | 10:62538088-62554610 | 0.0086296 | 0.033 | 1.29 | 0.80 | -1.75 |
| C20orf118 | 20:35504533-35580246 | 0.0041623 | 0.022 | 6.01 | 3.19 | -1.75 |
| CHUK,ERLIN1 | 10:101909850-101989376 | 0.0004856 | 0.008 | 15.95 | 9.04 | -1.76 |
| GPR27 | 3:71728439-71805647 | 0.0024248 | 0.016 | 26.25 | 14.90 | -1.76 |
| P2RY14 | 3:150803483-151176497 | 0.0107307 | 0.038 | 9.54 | 4.54 | -1.76 |
| SNX32 | 11:65590492-65641063 | 0.0012724 | 0.012 | 8.60 | 4.63 | -1.76 |
| C4orf29 | 4:128838959-128960866 | 0.0003091 | 0.007 | 3.56 | 1.98 | -1.76 |
| RP11-110C15.4.1 | 3:185000728-185216845 | 0.0119979 | 0.041 | 2.73 | 1.45 | -1.77 |
| SPEN | 1:16160559-16266955 | 0.0004773 | 0.008 | 16.18 | 9.00 | -1.77 |
| ARMC8 | 3:137906108-138048728 | 0.0034006 | 0.019 | 16.98 | 8.26 | -1.77 |
| IL2RA | 10:6052651-6104333 | 0.0001894 | 0.006 | 3.59 | 1.94 | -1.77 |
| DDHD2 | 8:38082735-38239790 | 0.000069 | 0.005 | 25.40 | 14.57 | -1.77 |
| MZT1 | 13:73282494-73301825 | 0.0008622 | 0.01 | 3.92 | 2.21 | -1.77 |
| TCP11L2 | 12:106631654-106740793 | 0.0022559 | 0.016 | 19.53 | 11.19 | -1.78 |
| AC092620.2.1,SPOPL | 2:139259370-139331818 | 0.0000126 | 0.003 | 15.32 | 8.52 | -1.78 |
| RP11-44F14.2.1 | 16:53407404-53418657 | 0.0045078 | 0.023 | 10.11 | 5.45 | -1.78 |
| TTC33 | 5:40714576-40756077 | 0.0022081 | 0.016 | 3.49 | 1.90 | -1.78 |
| RP11-44F14.1.1,RP11-44F14.3.1 | 16:53399800-53406995 | 0.0009949 | 0.011 | 16.81 | 9.02 | -1.78 |
| RNF169 | 11:74459912-74660245 | 0.0021576 | 0.015 | 2.58 | 1.43 | -1.78 |
| DAPP1 | 4:100737989-100791311 | 0.0000177 | 0.004 | 44.01 | 24.63 | -1.78 |
| OR10Y1P,STX3 | 11:59480295-59573354 | 0.0006139 | 0.009 | 91.35 | 50.72 | -1.79 |
| ANKRA2 | 5:72848159-72877794 | 0.0064258 | 0.028 | 9.51 | 5.23 | -1.79 |
| KLHL2 | 4:165997255-166244308 | 0.0001212 | 0.006 | 21.52 | 11.90 | -1.79 |
| AP1AR | 4:113152892-113191203 | 0.0049729 | 0.024 | 3.56 | 1.98 | -1.79 |
| ZFAND1 | 8:82613568-82671750 | 3.25E-06 | 0.003 | 11.96 | 6.67 | -1.80 |
| AP002884.2.1,BCO2,RPS12P21,SDHD,TEX12 | 11:111895537-112095422 | 7.58E-06 | 0.003 | 105.88 | 58.75 | -1.80 |
| AC144521.1.1 | 3:17199898-18568819 | 0.0091083 | 0.035 | 3.00 | 1.49 | -1.80 |
| RP5-1113E3.3.1 | 1:10516578-10690815 | 0.0140637 | 0.046 | 1.69 | 0.76 | -1.80 |
| RP11-396K3.1.1 | 7:72569032-72619657 | 0.0026016 | 0.017 | 30.49 | 16.86 | -1.80 |
| ZNF669 | 1:247261405-247267674 | 0.0027176 | 0.017 | 3.14 | 1.70 | -1.81 |
| KLF6 | 10:3818187-3827473 | 7.84E-06 | 0.003 | 86.74 | 48.02 | -1.81 |
| RP11-796E2.4.1 | 12:92378755-92583436 | 0.0037592 | 0.021 | 16.40 | 8.51 | -1.81 |
| FAM76B | 11:95502105-95565857 | 0.0022247 | 0.016 | 6.06 | 3.24 | -1.81 |
| AC116366.5.1 | 5:131746327-131811736 | 0.0089437 | 0.034 | 23.13 | 11.24 | -1.81 |
| ZNF566 | 19:36936020-36980804 | 0.0029483 | 0.018 | 1.34 | 0.76 | -1.81 |
| ZNF611 | 19:53206065-53238307 | 0.0007331 | 0.01 | 1.43 | 0.79 | -1.81 |
| CTA-373H7.7.1 | 22:27042391-27176170 | 0.0042648 | 0.022 | 4.12 | 2.08 | -1.81 |
| CTD-2036P10.3.1 | 15:43030931-43213007 | 0.0064338 | 0.028 | 1.62 | 0.86 | -1.81 |
| RP11-288H12.3.1 | 6:160390130-160534539 | 0.0089794 | 0.034 | 2.72 | 1.53 | -1.82 |
| BBS10 | 12:76738253-76742222 | 0.004455 | 0.023 | 2.77 | 1.47 | -1.82 |
| PDE4B | 1:66258196-66840259 | 0.0030917 | 0.019 | 17.38 | 9.38 | -1.82 |
| AC107983.1.1,AC107983.6.1,CCDC144B | 17:18414534-18528930 | 0.0077231 | 0.031 | 2.08 | 1.06 | -1.82 |
| RP5-1043L13.1.1 | 20:58662894-59102516 | 0.0058897 | 0.027 | 4.15 | 2.06 | -1.82 |
| PPP1R3D | 20:58508818-58523735 | 0.0000476 | 0.005 | 14.46 | 7.90 | -1.82 |
| AL133458.1 | 6:166822851-167370679 | 0.0013549 | 0.013 | 11.62 | 6.22 | -1.82 |
| AC007278.3.1 | 2:103035148-103069025 | 0.0089895 | 0.034 | 1.86 | 0.91 | -1.82 |
| FAM18B1 | 17:18684518-18710027 | 0.0008498 | 0.01 | 13.55 | 7.46 | -1.83 |
| FGD5-AS1 | 3:14860468-15106842 | 0.0000578 | 0.005 | 31.17 | 17.00 | -1.83 |
| C7orf60 | 7:112459201-112579971 | 0.0000551 | 0.005 | 5.95 | 3.23 | -1.83 |
| ZNF708 | 19:21473962-21512212 | 0.0004801 | 0.008 | 1.76 | 0.97 | -1.83 |
| HM13-IT1 | 20:30102230-30161066 | 0.015449 | 0.049 | 5.75 | 2.77 | -1.83 |
| PTGS2 | 1:186640922-186649559 | 0.0001229 | 0.006 | 6.91 | 3.77 | -1.83 |
| RP11-651P23.4.1 | 3:149478891-149942977 | 0.0112087 | 0.04 | 2.73 | 1.29 | -1.83 |
| ATF1 | 12:51157492-51214905 | 0.0005527 | 0.009 | 11.85 | 6.44 | -1.83 |
| PFKFB2 | 1:207217193-207254369 | 0.0017581 | 0.014 | 5.08 | 2.57 | -1.83 |
| CNIH | 14:54893653-54908149 | 0.0012584 | 0.012 | 33.62 | 18.19 | -1.83 |
| FERP1 | X:122734411-122866906 | 0.0024498 | 0.016 | 2.05 | 1.07 | -1.84 |
| CCNJ | 10:97454773-97849995 | 0.0001416 | 0.006 | 2.40 | 1.28 | -1.84 |
| MAN2A2 | 15:91445447-91465814 | 0.0004277 | 0.008 | 67.54 | 35.71 | -1.84 |
| TMEM154 | 4:153539783-153601317 | 0.0007995 | 0.01 | 46.88 | 25.07 | -1.84 |
| CENPK | 5:64813592-64858998 | 0.0017157 | 0.014 | 2.98 | 1.55 | -1.84 |
| RP11-392O18.1.1 | 3:150259780-150348222 | 0.0000264 | 0.004 | 109.83 | 59.23 | -1.84 |
| ZNF397 | 18:32820993-32870196 | 0.0009632 | 0.011 | 5.93 | 3.18 | -1.84 |
| TOR1AIP1 | 1:179851176-179888412 | 0.0005667 | 0.009 | 29.80 | 16.36 | -1.85 |
| WDR45L | 17:80572437-80606411 | 0.0003734 | 0.007 | 18.79 | 10.08 | -1.85 |
| AF131215.2.1 | 8:10753554-11058875 | 0.0019934 | 0.015 | 1.30 | 0.70 | -1.85 |
| VEGFA | 6:43737920-43754224 | 0.0014505 | 0.013 | 5.36 | 2.84 | -1.85 |
| RP11-206L10.2.1,RP11-206L10.5.1 | 1:694411-714006 | 0.0087052 | 0.034 | 125.54 | 63.43 | -1.85 |
| AC087590.3.1 | 3:15295690-15382875 | 0.0000545 | 0.005 | 5.81 | 3.10 | -1.86 |
| RP5-1142A6.9.1 | 16:88772870-88851619 | 0.0005605 | 0.009 | 4.13 | 2.19 | -1.86 |
| VHL,snoU13 | 3:10182691-10193904 | 0.0004534 | 0.008 | 24.20 | 12.98 | -1.86 |
| ELOVL5 | 6:53132195-53213947 | 0.0002054 | 0.006 | 102.72 | 54.90 | -1.87 |
| ADAM1 | 12:112337066-112339195 | 0.0055811 | 0.026 | 1.21 | 0.62 | -1.87 |
| LL22NC03-86G7.1.1 | 22:22273792-22307209 | 0.0012517 | 0.012 | 1.59 | 0.82 | -1.87 |
| ADAT2 | 6:143747077-143771810 | 0.0014697 | 0.013 | 3.87 | 2.08 | -1.87 |
| LENG8-AS1 | 19:54955990-54974894 | 0.0037703 | 0.021 | 3.18 | 1.61 | -1.88 |
| MTRR | 5:7830490-7906138 | 0.0000823 | 0.005 | 9.41 | 5.08 | -1.88 |
| ARGLU1 | 13:107194020-107220512 | 0.0007045 | 0.009 | 71.83 | 37.59 | -1.88 |
| TIRAP | 11:126152959-126215644 | 0.0006731 | 0.009 | 4.58 | 2.37 | -1.88 |
| CASS4 | 20:54987167-55034396 | 0.002392 | 0.016 | 13.90 | 7.28 | -1.88 |
| ARRDC3 | 5:90664540-90679176 | 0.0013935 | 0.013 | 64.37 | 33.16 | -1.89 |
| GNB4 | 3:179116989-179169378 | 0.0016708 | 0.014 | 4.77 | 2.43 | -1.89 |
| SLC25A16 | 10:70242520-70287231 | 0.000865 | 0.01 | 5.59 | 2.80 | -1.89 |
| CTD-2119F7.2.1 | 16:51679666-51681043 | 0.0067525 | 0.029 | 14.14 | 6.58 | -1.90 |
| PELI1 | 2:64319785-64479993 | 0.0000784 | 0.005 | 49.72 | 26.06 | -1.91 |
| THAP6 | 4:76404246-76475683 | 0.0000734 | 0.005 | 6.98 | 3.63 | -1.91 |
| DYNC1LI2 | 16:66754795-66788643 | 0.0027677 | 0.018 | 7.52 | 3.77 | -1.91 |
| RBP5 | 12:7276279-7281538 | 0.0019795 | 0.015 | 1.95 | 1.02 | -1.91 |
| PHACTR1 | 6:12717892-13328776 | 0.0009525 | 0.011 | 5.64 | 2.86 | -1.92 |
| CTD-2353F22.1.1 | 5:36606456-36725297 | 0.0055486 | 0.026 | 3.79 | 1.97 | -1.92 |
| ZNF281 | 1:200375826-200379184 | 0.0038809 | 0.021 | 3.53 | 1.77 | -1.92 |
| LINC00481 | 6:31165536-31171745 | 0.0018781 | 0.014 | 6.68 | 3.30 | -1.92 |
| RBM3 | X:48431481-48437454 | 0.0001888 | 0.006 | 204.43 | 103.61 | -1.93 |
| RP11-809O17.1.1 | 8:142127376-142205907 | 0.0004889 | 0.008 | 9.19 | 4.63 | -1.93 |
| TANK | 2:161952741-162111154 | 0.0000707 | 0.005 | 48.61 | 25.17 | -1.94 |
| NFXL1 | 4:47849256-48042188 | 0.0002579 | 0.007 | 5.75 | 2.80 | -1.94 |
| RP11-1049A21.2.1 | 12:54747444-54937889 | 0.0051575 | 0.025 | 5.10 | 2.61 | -1.94 |
| CCT6P1 | 7:65216128-65228341 | 0.00016 | 0.006 | 3.10 | 1.57 | -1.94 |
| MARCH5 | 10:94050919-94113721 | 7.88E-07 | 0.002 | 16.29 | 8.34 | -1.94 |
| LRRN2 | 1:204586297-204654861 | 0.0029654 | 0.018 | 1.04 | 0.52 | -1.94 |
| CCR4 | 3:32993065-32997841 | 0.0061947 | 0.027 | 4.88 | 2.61 | -1.94 |
| GPR15 | 3:98216447-98312567 | 0.0125858 | 0.043 | 3.52 | 1.52 | -1.95 |
| AC069282.6.1 | 7:47735327-48019178 | 0.0068536 | 0.029 | 4.46 | 2.05 | -1.95 |
| RP11-856B14.1.1 | 11:65335952-65337894 | 0.0003929 | 0.008 | 1.61 | 0.81 | -1.95 |
| HCG11 | 6:26522075-26526807 | 0.0002581 | 0.007 | 1.79 | 0.92 | -1.95 |
| CSAD,RP11-1136G11.7.1 | 12:53551447-53583208 | 0.0002351 | 0.007 | 37.17 | 18.47 | -1.96 |
| ANK1 | 8:41510738-41754280 | 0.002602 | 0.017 | 27.33 | 13.52 | -1.96 |
| LRCH3 | 3:197518096-197615307 | 0.0095122 | 0.036 | 17.89 | 7.16 | -1.97 |
| MICALCL | 11:12297626-12380691 | 0.0063072 | 0.028 | 2.91 | 1.29 | -1.97 |
| TPRG1-AS1 | 3:188659503-189043093 | 0.0075575 | 0.031 | 1.22 | 0.60 | -1.97 |
| TMEM161B | 5:87485449-87794514 | 5.96E-06 | 0.003 | 4.18 | 2.13 | -1.97 |
| CD24P4 | Y:21034386-21239302 | 0.0074477 | 0.031 | 37.19 | 17.69 | -1.97 |
| ZNF486 | 19:20278022-20308984 | 0.0021413 | 0.015 | 1.98 | 0.95 | -1.97 |
| RELL1 | 4:37455562-37687998 | 0.000402 | 0.008 | 6.65 | 3.26 | -1.98 |
| SGK1 | 6:134490383-134639250 | 0.001105 | 0.011 | 71.66 | 35.43 | -1.98 |
| AC007620.3.1 | 3:179115404-179116506 | 0.0082351 | 0.032 | 15.47 | 7.59 | -1.98 |
| KLF7 | 2:207938860-208031991 | 0.0000182 | 0.004 | 8.10 | 4.12 | -1.99 |
| HRH4 | 18:22040592-22059921 | 0.0032106 | 0.019 | 1.65 | 0.78 | -1.99 |
| AZIN1 | 8:103838584-103990104 | 0.0000399 | 0.005 | 26.76 | 13.52 | -1.99 |
| RAPGEF2 | 4:160025329-160281321 | 0.000211 | 0.006 | 9.10 | 4.44 | -1.99 |
| PAX8 | 2:113969098-114036527 | 0.0094847 | 0.036 | 0.98 | 0.54 | -1.99 |
| P2RY12 | 3:150803483-151176497 | 0.0016117 | 0.013 | 2.21 | 1.07 | -2.00 |
| F2RL1 | 5:76114757-76131140 | 0.0001813 | 0.006 | 10.34 | 5.02 | -2.00 |
| SLC25A40 | 7:87462882-87538856 | 0.0004009 | 0.008 | 11.92 | 5.73 | -2.00 |
| AC138783.13.1,GTF2IP1 | 7:74508363-74789332 | 0.0014268 | 0.013 | 21.72 | 10.77 | -2.01 |
| RP11-154D6.1.1 | 6:71961060-72037787 | 0.0001493 | 0.006 | 128.37 | 62.16 | -2.01 |
| RP11-173P15.3.1 | 12:121124671-121139667 | 0.0050371 | 0.024 | 14.54 | 7.21 | -2.01 |
| UEVLD | 11:18552747-18610294 | 0.0000599 | 0.005 | 3.73 | 1.81 | -2.02 |
| RP11-611O2.5.1 | 12:69201955-69365350 | 0.0012861 | 0.012 | 17.90 | 8.48 | -2.02 |
| C14orf129 | 14:96747594-96853625 | 0.0001468 | 0.006 | 13.02 | 6.44 | -2.02 |
| NBPF8 | 1:144593362-144621656 | 0.0045299 | 0.023 | 5.20 | 2.42 | -2.04 |
| MIRLET7DHG | 9:96938883-96966818 | 0.0021021 | 0.015 | 1.72 | 0.75 | -2.04 |
| DR1 | 1:93615298-93828149 | 0.000056 | 0.005 | 21.24 | 10.44 | -2.04 |
| ASB16 | 17:42248073-42264082 | 0.0001672 | 0.006 | 1.33 | 0.65 | -2.06 |
| CTD-2574D22.3.1 | 16:29911695-29940254 | 0.0099852 | 0.037 | 1.11 | 0.46 | -2.06 |
| LONRF1 | 8:12579402-12613582 | 0.0001511 | 0.006 | 7.48 | 3.45 | -2.06 |
| RP11-565J7.3.1 | 1:212208918-212280742 | 0.0073203 | 0.03 | 31.65 | 12.28 | -2.07 |
| SCML1 | X:17755587-17773105 | 0.0006621 | 0.009 | 1.62 | 0.82 | -2.07 |
| TFAM | 10:60144781-60158981 | 0.0003011 | 0.007 | 7.95 | 3.71 | -2.07 |
| RP11-455F5.5.1 | 16:30194147-30200397 | 0.0057218 | 0.026 | 86.00 | 35.25 | -2.08 |
| SNAP23 | 15:42783430-42841000 | 0.0004864 | 0.008 | 90.64 | 43.65 | -2.08 |
| RNFT1 | 17:58029600-58042122 | 0.0009901 | 0.011 | 13.70 | 6.48 | -2.08 |
| RP11-298J23.6.1,USP49 | 6:41762106-41863099 | 0.0067509 | 0.029 | 1.01 | 0.42 | -2.09 |
| RP11-298I3.1.1 | 14:23398817-23451851 | 0.0025256 | 0.017 | 4.88 | 2.33 | -2.09 |
| RGS18 | 1:192127586-192154945 | 0.0000594 | 0.005 | 84.54 | 39.69 | -2.10 |
| RP11-930O11.1.1 | 14:56777597-56811719 | 0.0102971 | 0.037 | 1.20 | 0.46 | -2.10 |
| RP11-34P13.13.1 | 1:164404-173864 | 0.0032689 | 0.019 | 21.52 | 9.29 | -2.10 |
| NAMPT | 7:105888730-105926772 | 0.0001702 | 0.006 | 1173.99 | 564.70 | -2.10 |
| SNX10 | 7:26331540-26416321 | 2.33E-06 | 0.003 | 52.80 | 24.90 | -2.11 |
| AC123595.1 | 5:90584748-90585004 | 0.0075131 | 0.031 | 14.47 | 6.31 | -2.12 |
| RP11-488L18.4.1 | 1:247353152-247372795 | 0.0005981 | 0.009 | 13.88 | 6.65 | -2.12 |
| NEAT1 | 11:65190244-65213011 | 0.0002432 | 0.007 | 76.29 | 35.52 | -2.12 |
| RP11-471B22.2.1 | 14:90862845-90874605 | 0.0120315 | 0.041 | 7.45 | 2.85 | -2.12 |
| AC133750.1 | 3:121350245-121379774 | 0.0151748 | 0.048 | 11.37 | 4.81 | -2.12 |
| MTFR1 | 8:66556123-66754557 | 0.0000383 | 0.005 | 7.64 | 3.54 | -2.13 |
| ZNF621 | 3:40566368-40616176 | 0.003408 | 0.019 | 1.05 | 0.49 | -2.14 |
| SLC38A1 | 12:46581583-46663800 | 0.0013064 | 0.012 | 3.41 | 1.65 | -2.14 |
| TMEM192 | 4:165997255-166244308 | 0.0019901 | 0.015 | 3.28 | 1.49 | -2.14 |
| PLAGL1 | 6:144261436-144385735 | 0.0000115 | 0.003 | 10.47 | 4.83 | -2.15 |
| LYZ | 12:69742120-69748014 | 0.0001377 | 0.006 | 4099.60 | 1784.83 | -2.15 |
| RPL21P119 | 16:9250218-9250761 | 0.0033029 | 0.019 | 87.10 | 34.93 | -2.15 |
| CYSLTR2 | 13:49280950-49283498 | 0.0025025 | 0.017 | 2.08 | 0.92 | -2.15 |
| CTC-484P3.1.1 | 5:93488670-94620279 | 0.0040538 | 0.021 | 2.64 | 1.03 | -2.15 |
| MRPL53P1 | 1:113066139-113214241 | 0.0075168 | 0.031 | 2.45 | 1.03 | -2.17 |
| CXCL6,RP11-629B11.2.1 | 4:74702213-74714781 | 0.0014647 | 0.013 | 1.30 | 0.57 | -2.18 |
| APOL6 | 22:36044441-36057404 | 0.0012436 | 0.012 | 4.68 | 2.03 | -2.18 |
| SLC12A6 | 15:34525459-34630261 | 0.000107 | 0.005 | 16.90 | 7.64 | -2.18 |
| RBL2 | 16:53467888-53538323 | 0.000556 | 0.009 | 31.21 | 14.41 | -2.19 |
| CXCL5 | 4:74861358-74864496 | 0.0077512 | 0.031 | 1.94 | 0.79 | -2.19 |
| 7SK | 1:33780295-33896653 | 0.0152069 | 0.048 | 7.78 | 2.81 | -2.19 |
| HBB | 11:5246693-5250625 | 0.0022267 | 0.016 | 12955.25 | 5526.57 | -2.19 |
| BAIAP2L1 | 7:97881690-98030380 | 0.0065926 | 0.028 | 1.59 | 0.72 | -2.19 |
| RP11-632K20.7.1 | 15:32814893-32879814 | 0.0025492 | 0.017 | 2.64 | 1.17 | -2.20 |
| RP11-1280N14.3-001.1 | 5:70398724-70424580 | 0.0015037 | 0.013 | 3.76 | 1.59 | -2.21 |
| PHIP | 6:79650262-79787953 | 0.0004702 | 0.008 | 1.90 | 0.89 | -2.21 |
| RP11-335O4.3.1 | 12:68042117-68064699 | 0.0133656 | 0.044 | 1.21 | 0.37 | -2.21 |
| PAG1 | 8:81882525-82024303 | 0.001024 | 0.011 | 9.67 | 4.55 | -2.21 |
| ZNF587 | 19:58331093-58376485 | 0.0001909 | 0.006 | 4.71 | 2.07 | -2.22 |
| KCNE3 | 11:74041362-74197096 | 0.0000224 | 0.004 | 57.56 | 25.57 | -2.24 |
| PRDM5 | 4:121606073-121844025 | 0.0066432 | 0.029 | 3.07 | 0.86 | -2.25 |
| LIG4 | 13:108859793-108870716 | 0.000366 | 0.007 | 2.54 | 1.10 | -2.27 |
| KB-431C1.4.1 | 8:103251621-103425069 | 0.0005865 | 0.009 | 7.99 | 3.43 | -2.28 |
| HIST1H1PS1 | 6:26195793-26195999 | 0.0018147 | 0.014 | 38.84 | 16.10 | -2.28 |
| ASH1L,MIR555 | 1:155305058-155533735 | 0.0000564 | 0.005 | 4.10 | 1.77 | -2.28 |
| AC007000.12.1 | 7:76090992-77046115 | 0.0072997 | 0.03 | 1.23 | 0.51 | -2.28 |
| BZW1P2 | 3:115528640-117716439 | 0.0033154 | 0.019 | 8.61 | 2.89 | -2.29 |
| CTD-3126B10.1.1 | 16:2587964-2653189 | 0.0005234 | 0.009 | 4.47 | 1.90 | -2.30 |
| HSPB1P1 | 9:74622796-74623052 | 0.0091256 | 0.035 | 4.41 | 1.14 | -2.31 |
| RP11-695J4.2.1 | 12:14656594-14910897 | 0.0007374 | 0.01 | 3.16 | 1.34 | -2.32 |
| ARL11 | 13:50202434-50208008 | 0.0069767 | 0.029 | 56.51 | 17.47 | -2.33 |
| AC006160.8.1,FAM184B | 4:17630928-17783135 | 0.0001066 | 0.005 | 4.84 | 1.93 | -2.34 |
| PMAIP1 | 18:57567179-57571538 | 0.0000684 | 0.005 | 7.47 | 3.13 | -2.34 |
| UBE4A | 11:118230299-118302211 | 0.0002679 | 0.007 | 5.88 | 2.50 | -2.34 |
| P2RX7 | 12:121570621-121623876 | 0.0014084 | 0.013 | 3.06 | 1.28 | -2.35 |
| RP11-750H9.5.1 | 11:47404698-47438047 | 0.0001002 | 0.005 | 99.94 | 41.61 | -2.37 |
| TSSK4 | 14:24641061-24708448 | 0.0075795 | 0.031 | 3.96 | 1.20 | -2.37 |
| C12orf39 | 12:21679240-21757781 | 0.0003559 | 0.007 | 3.16 | 1.36 | -2.37 |
| B3GNT5 | 3:182895830-183146566 | 0.000071 | 0.005 | 5.16 | 2.19 | -2.38 |
| RP1-283E3.4.1,RP1-283E3.8.1 | 1:1656053-1677431 | 0.0038316 | 0.021 | 4.13 | 1.46 | -2.38 |
| RP11-575L7.8.1 | 9:86582997-86595569 | 0.002375 | 0.016 | 8.67 | 3.70 | -2.39 |
| ZNF137P | 19:53095290-53100660 | 0.0007007 | 0.009 | 1.52 | 0.60 | -2.39 |
| RP11-356C4.4.1 | 16:90167797-90244752 | 0.0004044 | 0.008 | 23.45 | 9.48 | -2.40 |
| AC135776.1 | 16:34232105-34232469 | 0.0014481 | 0.013 | 10.00 | 4.03 | -2.40 |
| RP11-793H13.3.1 | 12:53900471-54020199 | 0.0042662 | 0.022 | 1.48 | 0.58 | -2.40 |
| AC098824.5.1 | 2:43024364-43025852 | 0.0009646 | 0.011 | 1.90 | 0.68 | -2.41 |
| TMSL3 | 4:91048685-92523064 | 0.0001213 | 0.006 | 152.56 | 59.48 | -2.41 |
| TRIM56 | 7:100728719-100735017 | 2.27E-06 | 0.003 | 11.24 | 4.55 | -2.46 |
| RP11-120K18.3.1 | 16:31366454-31394318 | 0.0101605 | 0.037 | 4.28 | 1.52 | -2.48 |
| J01415.24,MT-ATP6,MT-CO3 | MT:8365-9990 | 0.0025529 | 0.017 | 39885.13 | 13424.01 | -2.49 |
| RP11-1149O23.2.1 | 8:23046791-23088439 | 0.0077945 | 0.032 | 1.72 | 0.73 | -2.49 |
| ANKRD10-IT1 | 13:111530886-111567416 | 0.0068189 | 0.029 | 8.19 | 3.04 | -2.50 |
| AC008993.1 | 19:65916-70966 | 0.0002748 | 0.007 | 8.12 | 3.22 | -2.51 |
| TBC1D14 | 4:6910968-7058368 | 0.0000601 | 0.005 | 29.13 | 11.49 | -2.53 |
| RP11-632C17__A.1.1 | 6:118228688-118638839 | 0.0012626 | 0.012 | 9.04 | 3.19 | -2.53 |
| SLC7A6 | 16:68298432-68344849 | 0.0006334 | 0.009 | 10.58 | 3.78 | -2.55 |
| DDX6 | 11:118575315-118661858 | 0.0001351 | 0.006 | 22.78 | 9.06 | -2.55 |
| RPS10L.1 | 20:814357-837992 | 0.0013038 | 0.012 | 1.32 | 0.49 | -2.57 |
| RP11-34P13.12.1 | 1:141473-149707 | 0.001153 | 0.012 | 20.38 | 7.59 | -2.57 |
| AC005841.1 | 12:2904118-3050306 | 0.010449 | 0.038 | 1.02 | 0.26 | -2.59 |
| RP11-3J10.4.1 | 9:37510888-38069210 | 0.000667 | 0.009 | 3.57 | 1.36 | -2.59 |
| RP11-329A14.1.1 | 1:48761043-48937845 | 0.0019157 | 0.015 | 3.01 | 0.87 | -2.59 |
| MORF4L1P1 | 1:220321634-220445796 | 0.001778 | 0.014 | 1.96 | 0.67 | -2.59 |
| RP1-50O24.6.1 | 1:27202623-27204736 | 0.0069843 | 0.029 | 7.32 | 2.27 | -2.65 |
| RP11-206L10.3.1 | 1:677192-685396 | 0.001661 | 0.014 | 7.35 | 2.49 | -2.68 |
| RNF149,SNORD89 | 2:101887680-101925163 | 0.000039 | 0.005 | 9638.26 | 3462.52 | -2.69 |
| IL8 | 4:74606222-74609433 | 0.0013862 | 0.013 | 9.03 | 2.94 | -2.72 |
| RPL21P28 | 7:19958603-20257027 | 0.0100706 | 0.037 | 27.07 | 6.44 | -2.75 |
| RP11-27I1.4.1 | 9:123555828-123561848 | 0.0014946 | 0.013 | 1.27 | 0.43 | -2.80 |
| CCNG2 | 4:78078303-78415440 | 0.0002079 | 0.006 | 25.51 | 9.14 | -2.82 |
| RP11-30L4.5.1 | 9:96208775-96328397 | 0.0017287 | 0.014 | 8.85 | 2.98 | -2.84 |
| AC073850.6.1 | 7:79998890-80308593 | 0.0104742 | 0.038 | 3.49 | 0.55 | -2.85 |
| RP11-707P17.2.1 | 15:51706293-51915030 | 0.0051958 | 0.025 | 1.23 | 0.42 | -2.85 |
| RP11-177C12.1.1 | 4:37869803-37871589 | 0.00376 | 0.021 | 2.00 | 0.56 | -2.93 |
| VNN2-AS1 | 6:133065008-133084598 | 0.0002302 | 0.007 | 8.29 | 2.80 | -2.95 |
| MTPN | 7:135611202-135662101 | 0.0003024 | 0.007 | 34.54 | 11.75 | -2.96 |
| RP1-309F20.2.1 | 20:57393973-57486247 | 0.0042493 | 0.022 | 6.60 | 2.05 | -3.01 |
| LSM12P1 | 8:35092974-35654068 | 0.0038009 | 0.021 | 1.90 | 0.52 | -3.02 |
| ZNF770 | 15:35270541-35280488 | 0.000149 | 0.006 | 2.31 | 0.78 | -3.02 |
| RP11-18H7.1.1 | 3:119813741-119855630 | 0.0093295 | 0.035 | 1.51 | 0.45 | -3.05 |
| AP000640.8.1 | 11:59480295-59573354 | 0.00555 | 0.026 | 2.64 | 0.57 | -3.06 |
| RPL39P | 20:55034483-55034639 | 0.0007625 | 0.01 | 1609.29 | 442.31 | -3.07 |
| EMR4P | 19:6952510-6990857 | 0.0000236 | 0.004 | 4.86 | 1.47 | -3.08 |
| RP11-325K4.2.1,RP11-325K4.3.1 | 16:56965959-56977798 | 0.0038012 | 0.021 | 2.36 | 0.64 | -3.08 |
| SMCR8 | 17:18218715-18226517 | 0.0002962 | 0.007 | 3.28 | 1.02 | -3.13 |
| FKBP1C | 6:63921350-63922929 | 0.0057951 | 0.026 | 20.24 | 4.70 | -3.17 |
| AC093162.5.1 | 2:85542163-85543652 | 0.0040675 | 0.022 | 4.84 | 1.47 | -3.26 |
| RP11-10A14.3.1 | 8:8993764-9009084 | 0.0025709 | 0.017 | 3.24 | 0.94 | -3.35 |
| PRR11 | 17:57233100-57279924 | 0.0023595 | 0.016 | 1.31 | 0.32 | -3.44 |
| RP11-181C21.4.1 | 1:78245308-78344106 | 0.000474 | 0.008 | 0.98 | 0.25 | -3.46 |
| AKIRIN2-AS1 | 6:88384789-88411927 | 1.25E-06 | 0.002 | 7.87 | 2.24 | -3.54 |
| ZDHHC20 | 13:21946716-22033509 | 0.0024743 | 0.017 | 9.65 | 2.58 | -3.81 |
| TAS2R40 | 7:142919129-142920162 | 0.0007178 | 0.01 | 1.38 | 0.29 | -3.82 |
| RP1-187N21.2.1 | 6:34252266-34254545 | 0.0011006 | 0.011 | 3.97 | 0.86 | -4.06 |
| AC016745.3.1 | 2:114182071-114182901 | 0.0102992 | 0.037 | 1.39 | 0.46 | -4.09 |
| AC073150.4.1 | 7:27497781-27498468 | 0.0008087 | 0.01 | 2.50 | 0.51 | -4.19 |
| SRP9P1 | 10:93558068-93625033 | 0.0032048 | 0.019 | 17.19 | 1.80 | -4.19 |
| RP11-246A10.1.1 | 3:151835961-151869513 | 0.0086877 | 0.034 | 1.62 | 0.31 | -4.33 |
| U1 | 1:149671722-149671868 | 0.0012901 | 0.012 | 179.71 | 34.96 | -4.42 |
| RP11-430B1.4.1 | 15:52599479-52821247 | 0.0134767 | 0.045 | 2.29 | 0.52 | -4.48 |
| MIR223 | X:65238711-65238821 | 0.0000447 | 0.005 | 104837.70 | 20086.97 | -4.60 |
| RP11-14N7.2.1 | 1:148928321-148951595 | 3.87E-06 | 0.003 | 40.83 | 8.46 | -4.67 |
| CTD-2235C13.1.1 | 5:74664310-74896969 | 0.0041563 | 0.022 | 3.02 | 0.37 | -5.25 |
| RP11-69H7.3.1 | 15:75487983-75504510 | 0.0000708 | 0.005 | 387.84 | 55.97 | -5.54 |
| AC093110.3.1 | 2:54683421-54896812 | 0.0055989 | 0.026 | 0.93 | 0.16 | -6.28 |
| RP11-9M16.2.1 | 9:117159003-117160783 | 0.0001565 | 0.006 | 175.91 | 19.82 | -7.49 |
| RP1-159A19.3.1 | 1:27975929-27976121 | 0.0161817 | 0.05 | 29.17 | 2.91 | -8.77 |
| RP11-110I1.5.1 | 11:118868851-118901616 | 0.0127409 | 0.043 | 49.80 | 4.58 | -9.81 |

**Table S5**. Canonical signaling pathways differentially expressed in blood 7 days after LVAD implant as compared to pre-LVAD expression. Differentially expressed transcripts with an FDR < 0.10 were analyzed with Ingenuity. The ratio is the fraction of genes represented in the dataset to the total number of genes in a known signaling pathway. A p-value is assigned and represents the likelihood of the event occurring by chance. Differentially expressed transcripts in each pathway are listed here.

| Ingenuity Canonical Pathway | p | Ratio | Transcripts |
| --- | --- | --- | --- |
| Tetrapyrrole Biosynthesis II | 0.0001 | 0.31 | UROS, ALAD, ALAS2, HMBS |
| CTLA4 Signaling in Cytotoxic T Lymphocytes | 0.0001 | 0.16 | CD247, AP2B1, HLA-DOA, PPP2R5D, CD4, AP1S2, PPP2R5B, HLA-DQA1, HLA-DRB1, AP2S1, PPP2CB, PIK3C3, HLA-DRA, CLTCL1, PIK3R6, PPP2R5C |
| RAN Signaling | 0.0003 | 0.25 | KPNB1, KPNA3, CSE1L, RANBP2, RAN, IPO5 |
| Antigen Presentation Pathway | 0.0003 | 0.23 | HLA-DOA, PDIA3, HLA-DRB3, HLA-DRA, HLA-DQA1, HLA-DRB1, CD74, HLA-DPB1, HLA-DPA1 |
| Cyclins and Cell Cycle Regulation | 0.0004 | 0.16 | TP53, RAF1, TFDP1, PPP2R5D, CUL1, HDAC1, PPP2R5B, CDKN2C, SIN3A, PPP2CB, CDK4, E2F1, PPP2R5C, E2F2 |
| Ovarian Cancer Signaling | 0.0005 | 0.13 | TP53, RAF1, TFDP1, VEGFB, TCF3, SIN3A, MLH1, PGF, BRAF, VEGFA, PRKAR2B, MSH2, PIK3C3, CDK4, E2F1, PIK3R6, FZD5, WNT11, FZD7 |
| OX40 Signaling Pathway | 0.0009 | 0.12 | CD247, BCL2L1, RELA, HLA-DOA, CD4, HLA-DRB3, HLA-DRA, HLA-DQA1, HLA-DRB1, HLA-DPB1, HLA-DPA1 |
| Pancreatic Adenocarcinoma Signaling | 0.0009 | 0.14 | TP53, RELA, RAF1, JAK1, TFDP1, VEGFB, SIN3A, PGF, PLD4, VEGFA, BCL2L1, PIK3C3, CDK4, E2F1, PIK3R6, STAT1, E2F2 |
| Heme Biosynthesis from Uroporphyrinogen-III I | 0.0010 | 0.27 | UROD, PPOX, FECH |
| Melatonin Degradation II | 0.0010 | 0.25 | MAOB, SMOX, MAOA |
| Interferon Signaling | 0.0010 | 0.22 | IFIT3, IFIT1, RELA, JAK1, IFI35, STAT1, IFNAR1, IRF1 |
| Cell Cycle Regulation by BTG Family Proteins | 0.0012 | 0.22 | PPP2CB, PPP2R5D, CDK4, E2F1, BTG2, PPP2R5B, PPP2R5C, E2F2 |
| Methylglyoxal Degradation I | 0.0040 | 0.18 | HAGH, GLO1 |
| Purine Ribonucleosides Degradation to Ribose-1-phosphate | 0.0040 | 0.12 | PNP, ADA |
| Role of BRCA1 in DNA Damage Response | 0.0040 | 0.15 | TP53, MSH2, SMARCA2, E2F1, SMARCD2, SLC19A1, STAT1, FANCA, MLH1, E2F2 |
| Cell Cycle: G2/M DNA Damage Checkpoint Regulation | 0.0049 | 0.17 | YWHAQ, TP53, KAT2B, YWHAG, YWHAB, TOP2B, CUL1, PKMYT1 |
| Phenylalanine Degradation IV (Mammalian, via Side Chain) | 0.0051 | 0.10 | MAOB, SMOX, GOT2, MAOA |
| Cytotoxic T Lymphocyte-mediated Apoptosis of Target Cells | 0.0054 | 0.11 | CD247, PRF1, HLA-DOA, HLA-DRB3, HLA-DRA, HLA-DQA1, HLA-DRB1, HLA-DPB1, HLA-DPA1 |
| Chronic Myeloid Leukemia Signaling | 0.0059 | 0.12 | TP53, RAF1, RELA, STAT5A, TFDP1, HDAC1, SIN3A, BCL2L1, PIK3C3, CDK4, E2F1, PIK3R6, E2F2 |
| Allograft Rejection Signaling | 0.0062 | 0.09 | PRF1, CD40LG, HLA-DOA, HLA-DRB3, HLA-DRA, HLA-DQA1, HLA-DRB1, HLA-DPB1, HLA-DPA1 |
| Dopamine Receptor Signaling | 0.0071 | 0.12 | PPP2CB, MAOB, PRKAR2B, PPP2R5D, PTS, PPP2R5B, PPP2R5C, SMOX, SLC18A2, QDPR, MAOA |
| Type I Diabetes Mellitus Signaling | 0.0078 | 0.12 | CD247, RELA, HLA-DOA, JAK1, HLA-DQA1, HLA-DRB1, MAPK13, MAP3K5, IRF1, PRF1, RIPK1, LTA, HLA-DRA, STAT1 |
| Glutathione Redox Reactions I | 0.0093 | 0.17 | MGST1, GPX1, GPX4, PRDX6 |
| Telomerase Signaling | 0.0098 | 0.13 | TP53, RAF1, PPP2R5D, HDAC1, PPP2R5B, IL2RB, ELF1, PPP2CB, PIK3C3, E2F1, PIK3R6, PPP2R5C, POT1 |
| HIF1 alpha Signaling | 0.0107 | 0.12 | SLC2A5, TP53, SLC2A1, VEGFB, MAPK13, MMP24, LDHB, PGF, VEGFA, PIK3C3, MMP8, PIK3R6, APEX1 |
| Adenine and Adenosine Salvage III | 0.0115 | 0.13 | PNP, ADA |
| Small Cell Lung Cancer Signaling | 0.0132 | 0.11 | TP53, BCL2L1, RELA, TFDP1, PIK3C3, CDK4, E2F1, PIK3R6, SIN3A, BIRC2 |
| Activation of IRF by Cytosolic Pattern Recognition Receptors | 0.0135 | 0.13 | IFIH1, RELA, RIPK1, LTA, DDX58, SIKE1, STAT1, IFIT2, IFNAR1 |
| IL-15 Signaling | 0.0135 | 0.13 | BCL2L1, RAF1, RELA, STAT5A, JAK1, PIK3C3, PIK3R6, MAPK13, IL2RB |
| Serotonin Receptor Signaling | 0.0135 | 0.13 | MAOB, PTS, SMOX, SLC18A2, QDPR, MAOA |
| Melanoma Signaling | 0.0148 | 0.15 | BRAF, TP53, RAF1, PIK3C3, CDK4, E2F1, PIK3R6 |
| Role of JAK1, JAK2 and TYK2 in Interferon Signaling | 0.0151 | 0.19 | RAF1, RELA, JAK1, STAT1, IFNAR1 |
| Tumoricidal Function of Hepatic Natural Killer Cells | 0.0151 | 0.21 | M6PR, PRF1, SERPINB9, SRGN, ITGAL |
| IL-9 Signaling | 0.0182 | 0.15 | RELA, STAT5A, JAK1, PIK3C3, PIK3R6, STAT1 |
| Granzyme A Signaling | 0.0191 | 0.20 | GZMA, PRF1, SET, APEX1 |
| Putrescine Degradation III | 0.0191 | 0.13 | ALDH1B1, MAOB, SMOX, MAOA |
| Role of CHK Proteins in Cell Cycle Checkpoint Control | 0.0191 | 0.14 | TP53, PPP2CB, PPP2R5D, E2F1, PPP2R5B, SLC19A1, PPP2R5C, E2F2 |
| Bladder Cancer Signaling | 0.0204 | 0.12 | VEGFA, TP53, RAF1, TFDP1, MMP8, CDK4, E2F1, VEGFB, MMP24, SIN3A, PGF |
| Proline Biosynthesis I | 0.0219 | 0.14 | ALDH18A1, PYCR1 |
| 14-3-3-mediated Signaling | 0.0224 | 0.11 | RAF1, YWHAG, PDIA3, YWHAB, TUBB2A, MAP3K5, TUBB, TUBA1B, YWHAQ, PIK3C3, PIK3R6, SNCA, AKT1S1 |
| Tryptophan Degradation X (Mammalian, via Tryptamine) | 0.0234 | 0.14 | ALDH1B1, MAOB, SMOX, MAOA |
| RhoA Signaling | 0.0245 | 0.11 | ACTR2, ARHGEF12, SEPT9, EZR, SEPT7, MYL4, PIP4K2B, CDC42EP1, ANLN, PIP5K1B, PIP4K2A, MSN |
| Ceramide Signaling | 0.0263 | 0.11 | PPP2CB, RAF1, RELA, CNKSR1, PPP2R5D, PIK3C3, PPP2R5B, PIK3R6, PPP2R5C, SMPD3 |
| T Helper Cell Differentiation | 0.0263 | 0.13 | STAT4, CD40LG, HLA-DOA, HLA-DRA, HLA-DQA1, HLA-DRB1, STAT1, TBX21, GATA3 |
| Renal Cell Carcinoma Signaling | 0.0282 | 0.12 | VEGFA, RAPGEF1, RAF1, PAK2, SLC2A1, PIK3C3, PIK3R6, FH, PDGFB |
| DNA Methylation and Transcriptional Repression Signaling | 0.0339 | 0.17 | HDAC1, SAP130, SIN3A, RBBP4 |
| Cdc42 Signaling | 0.0347 | 0.08 | CD247, RAF1, ACTR2, HLA-DOA, PAK2, HLA-DQA1, HLA-DRB1, MAPK13, HLA-DPA1, LLGL1, HLA-DRB3, HLA-DRA, MYL4, HLA-DPB1 |
| Tetrahydrofolate Salvage from 5,10-methenyltetrahydrofolate | 0.0347 | 0.18 | MTHFD2L, GART |
| Tight Junction Signaling | 0.0355 | 0.10 | RELA, CSTF1, CLDN15, RAB13, PPP2R5D, PPP2R5B, CSDA, PPP2CB, CPSF6, PRKAR2B, JAM3, LLGL1, CDK4, MYL4, PPP2R5C, CLDN9 |
| Cell Cycle: G1/S Checkpoint Regulation | 0.0363 | 0.12 | TP53, TFDP1, CDK4, E2F1, CUL1, HDAC1, SIN3A, E2F2 |
| ERK/MAPK Signaling | 0.0363 | 0.09 | MYCN, RAPGEF1, RAF1, YWHAG, PAK2, YWHAB, PPP2R5D, DUSP6, PPP2R5B, ELF1, BRAF, YWHAQ, PPP2CB, PRKAR2B, PIK3C3, PIK3R6, PPP2R5C, STAT1 |
| Glioma Signaling | 0.0363 | 0.10 | TP53, RAF1, TFDP1, PIK3C3, CDK4, E2F1, PIK3R6, CDKN2C, PDGFB, SIN3A, E2F2 |
| p70S6K Signaling | 0.0363 | 0.10 | RAF1, YWHAG, JAK1, PDIA3, YWHAB, PPP2R5D, PPP2R5B, YWHAQ, PPP2CB, PIK3C3, PIK3R6, LYN, PPP2R5C |
| NF-KB Activation by Viruses | 0.0389 | 0.11 | RAF1, RELA, CCR5, RIPK1, CD4, PIK3C3, PIK3R6, ITGAV, ITGAL |
| Amyotrophic Lateral Sclerosis Signaling | 0.0407 | 0.09 | VEGFA, CCS, TP53, BCL2L1, PIK3C3, GPX1, PIK3R6, VEGFB, PGF, SSR4, BIRC2 |
| PI3K/AKT Signaling | 0.0407 | 0.09 | TP53, RAF1, RELA, YWHAG, JAK1, YWHAB, PPP2R5D, PPP2R5B, MAP3K5, YWHAQ, PPP2CB, BCL2L1, PPP2R5C |
| Mitotic Roles of Polo-Like Kinase | 0.0437 | 0.12 | PPP2CB, SMC3, PPP2R5D, PPP2R5B, PKMYT1, PPP2R5C, CDC16, RAD21 |
| Role of PI3K/AKT Signaling in the Pathogenesis of Influenza | 0.0437 | 0.11 | KPNA3, RELA, CCR5, PIK3C3, PIK3R6, ZNF346, IFNAR1, MLH1 |
| VEGF Signaling | 0.0437 | 0.10 | EIF1AY, VEGFA, BCL2L1, RAF1, PIK3C3, PIK3R6, VEGFB, EIF2B3, ELAVL1, PGF |

**Table S6**. Canonical signaling pathways differentially expressed in blood 6 months (180 days) after LVAD implant as compared to pre-LVAD expression. Differentially expressed transcripts with an FDR < 0.10 were analyzed with Ingenuity. The ratio is the fraction of genes represented in the dataset to the total number of genes in a known signaling pathway. A p-value is assigned and represents the likelihood of the event occurring by chance. Differentially expressed transcripts in each pathway are listed here.

| Ingenuity Canonical Pathway | p | Ratio | Molecules |
| --- | --- | --- | --- |
| Protein Ubiquitination Pathway | 0.000000 | 0.30 | UBE2A, HSPA1A/HSPA1B, USP5, PSMA7, UBE2L3, DNAJC3, UBE2V2, FBXW7, PSMB8, HSPA5, PSMC5, USP48, HSPA4, UBE2B, PSMD10, UCHL5, ANAPC11, DNAJC30, PSMC2, BIRC3, UBE2J1, USP38, BIRC6, PSMC4, PSME2, PSMD6, DNAJC2, THOP1, PSMD3, UBE2S, PSMD11, CBL, USP32, PSMA5, PSMD12, ANAPC5, PSMA4, BAP1, PSMB1, HSP90AA1, PSMD1, PSMD4, PSMB3, DNAJC17, PSMB10, HLA-A, UBE2N, CUL1, USP11, DNAJA1, PSMB6, SMURF1, UBE4A, USP3, HSP90B1, PSMC6, USP42, USP16, DNAJC1, HLA-B, PSMD14, PSMB4, PSMD13, HSPH1, USP1, UBE2D1, PSMD8, PSME1, DNAJC24, PSMC1, USP22, PSMD2, DNAJB11, USP46, UBA1, UBC, PSMC3, DNAJB5, UBE2D3, DNAJC7 |
| NGF Signaling | 0.000000 | 0.36 | MAP2K4, RAP1B, RAF1, MAP3K11, PIK3R1, SOS2, PIK3R5, RPS6KA3, PDPK1, HRAS, KRAS, CRK, SMPD1, MAP3K5, CREB5, CDC42, MAP3K10, IKBKG, RHOG, SMPD4, MAP3K7, PIK3CG, PIK3C3, SOS1, RPS6KB2, MAP3K2, ATM, MAP3K14, AKT2, MAP3K6, RRAS, CREB3, RAC1, MAPK9, IKBKE, BAX, SMPD2, ATF2, ROCK1, PTPN11, MAP3K8, MAP3K3 |
| B Cell Receptor Signaling | 0.000000 | 0.32 | MAP2K4, RAF1, GAB2, MAP3K11, BAD, PIK3R1, SOS2, HRAS, KRAS, CREB5, PTPRC, MAP3K10, IKBKG, PIK3CG, NFKBIB, ATM, MAP3K2, MAP3K14, PTPN6, AKT2, CD19, RRAS, CREB3, RAC1, MALT1, ATF2, PTPN11, VAV1, PIK3AP1, CAMK2G, BLNK, PIK3R5, MAP3K5, CDC42, CD79A, MTOR, MAP3K7, PIK3C3, SOS1, RPS6KB2, CAMK2B, CD79B, MAP3K6, MAPK9, IKBKE, MAPK14, DAPP1, BCL10, PAG1, MAP3K8, MAP3K3, PRKCB |
| Mitochondrial Dysfunction | 0.000000 | 0.28 | MAP2K4, NDUFA10, NCSTN, NDUFB10, NDUFS6, NDUFA8, CASP3, CYCS, NDUFS7, ATP5A1, SURF1, NDUFB1, GSR, ATP5C1, PRDX3, NDUFS5, NDUFB11, TXN2, NDUFA6, NDUFB7, NDUFA12, CYC1, TXNRD2, COX5B, HSD17B10, PRDX5, RHOT2, PSENEN, COX7A2L, PARK7, NDUFAB1, CYB5R3, NDUFS2, OGDH, CASP8, AIFM1, SDHA, NDUFV1, COX7A2, GLRX2, MAPK9, UQCR10, COX11, NDUFV2, NDUFS8, CAT, NDUFA3, UQCRC1, UQCRQ |
| Germ Cell-Sertoli Cell Junction Signaling | 0.000000 | 0.31 | MAP2K4, TGFBR1, MAP3K11, AXIN1, PIK3R1, MLLT4, PDPK1, HRAS, KRAS, TUBB4A, TGFBR2, MAP3K10, RHOG, TGFB1, KEAP1, PIK3CG, ACTA1, ATM, MAP3K2, MAP3K14, RRAS, RAC1, RAC3, RHOQ, ZYX, TUBA3C/TUBA3D, CLINT1, TNF, FNBP1, RHOT2, PIK3R5, CTNNA1, MAP3K5, CDC42, IQGAP1, AGGF1, MAP3K7, PIK3C3, RAB8B, TUBB1, PAK4, EPN1, PAK2, MAP3K6, TUBB4B, TUBG1, MAPK9, ACTG1, MAPK14, MAP3K8, MAP3K3 |
| PKCθ Signaling in T Lymphocytes | 0.000000 | 0.29 | MAP2K4, FYN, MAP3K11, CD3E, PIK3R1, CD4, SOS2, PIK3R5, HLA-DRB1, HRAS, KRAS, MAP3K5, MAP3K10, IKBKG, LCK, MAP3K7, PIK3CG, PIK3C3, SOS1, HLA-DRA, NFKBIB, CAMK2B, ATM, MAP3K2, MAP3K14, MAP3K6, RRAS, RAC1, IKBKE, MALT1, RAC3, CD3D, BCL10, ZAP70, FCER1G, VAV1, MAP3K8, MAP3K3, CARD11, LCP2, CAMK2G |
| HGF Signaling | 0.000001 | 0.34 | MAP2K4, RAP1B, RAF1, RAPGEF1, MAP3K11, PIK3R1, SOS2, PIK3R5, HRAS, KRAS, MAP3K5, CDC42, MAP3K10, MAP3K7, PIK3CG, PIK3C3, SOS1, ATM, MAP3K2, MAP3K14, AKT2, MAP3K6, RRAS, CRKL, RAC1, MAPK9, STAT3, ELF1, ATF2, ELF2, PTPN11, MAP3K8, PTGS2, MAP3K3, CDK2, PRKCB |
| TNFR1 Signaling | 0.000005 | 0.40 | MAP2K4, MAP4K2, MAP3K14, PAK4, PAK2, CASP3, CYCS, APAF1, TNFAIP3, IKBKE, CDC42, TANK, TRADD, IKBKG, RIPK1, CRADD, CASP2, CASP8, NFKBIB, TNF, BIRC3 |
| Systemic Lupus Erythematosus Signaling | 0.000009 | 0.24 | SNRPC, CD3E, SNRPE, ZMAT5, PIK3R1, SOS2, TXNL4A, HRAS, KRAS, SART1, PTPRC, LCK, SNRPA, PIK3CG, PRPF40A, SNRPD1, NHP2L1, HLA-F, LSM4, ATM, PRPF38A, AKT2, PTPN6, RRAS, SNRPF, IL6R, SNRNP35, CBL, CD72, IGHG4, SF3B4, TNF, HLA-A, HNRNPA2B1, PIK3R5, IGHG1, CD79A, LSM14A, MTOR, IGHG3, LSM7, EFTUD2, PIK3C3, SOS1, HLA-B, IGHM, SNRPD2, PRPF31, HNRNPC, LSM10, SNRNP40, LSM14B, CD79B, PRPF38B, CD3D, FCER1G, PRPF6, SNRNP70, LSM5 |
| FLT3 Signaling in Hematopoietic Progenitor Cells | 0.000011 | 0.36 | GAB2, RAF1, BAD, PIK3R1, SOS2, RPS6KA3, PIK3R5, HRAS, PDPK1, KRAS, CREB5, EIF4EBP1, MTOR, PIK3CG, PIK3C3, SOS1, STAT5B, ATM, AKT2, RRAS, CREB3, STAT3, FLT3LG, ATF2, CBL, MAPK14, PTPN11 |
| Insulin Receptor Signaling | 0.000013 | 0.29 | RAF1, FOXO4, FYN, PPP1CC, RAPGEF1, TSC1, EIF2B4, BAD, SGK1, PIK3R1, SOS2, PIK3R5, HRAS, PDPK1, CRK, KRAS, EIF2B2, EIF4EBP1, MTOR, PPP1R7, PDE3B, PIK3C3, PIK3CG, SOS1, RPS6KB2, STX4, PPP1CA, ATM, AKT2, RRAS, CRKL, PPP1R14A, PPP1R3D, PRKAR2B, RHOQ, CBL, PTPN11, SH2B2, EIF2B5, PPP1R12A, PRKAR1A |
| Regulation of eIF4 and p70S6K Signaling | 0.000023 | 0.24 | RAF1, EIF2S2, EIF2B4, EIF4EBP2, PIK3R1, SOS2, PIK3R5, PDPK1, HRAS, PPP2R3B, RPS6, KRAS, PAIP2, EIF3C/EIF3CL, EIF2B2, EIF4EBP1, EIF2C4, MTOR, EIF3B, EIF1AX, PIK3C3, PIK3CG, SOS1, ATM, PABPC1, AKT2, RRAS, RPS28, RPS19, ITGA5, EIF3E, EIF3G, PPP2R1A, MAPK14, RPS5, RPS26, EIF4A3, PPP2R2B, EIF2B5, EIF3I, RPS15, EIF3K |
| Chronic Myeloid Leukemia Signaling | 0.000025 | 0.31 | RAF1, GAB2, TGFBR1, BAD, PIK3R1, SUV39H1, SOS2, PIK3R5, HRAS, CRK, KRAS, E2F3, RBL1, TGFBR2, CTBP1, IKBKG, TGFB1, PIK3CG, PIK3C3, CTBP2, SOS1, STAT5B, ATM, AKT2, RBL2, HDAC4, RRAS, CRKL, IKBKE, HDAC3, PTPN11, CDKN1B |
| SAPK/JNK Signaling | 0.000025 | 0.31 | MAP2K4, MAP3K11, PIK3R1, SOS2, PIK3R5, HRAS, KRAS, CRK, MAP3K5, CDC42, TRADD, MAP3K10, LCK, MAP3K7, PIK3CG, PIK3C3, SOS1, MAP4K1, GNA13, ATM, MAP3K2, SH2D2A, MAP4K2, RRAS, CRKL, RAC1, MAPK9, RAC3, ATF2, RIPK1, FCER1G, MAP3K3 |
| Glucocorticoid Receptor Signaling | 0.000032 | 0.24 | MAP2K4, RAF1, POLR2F, TAF11, TGFBR1, PRKAB2, CD3E, SGK1, HSPA1A/HSPA1B, PIK3R1, SOS2, HRAS, SMARCD2, KRAS, GTF2E2, POMC, TAF13, HSPA5, TGFBR2, HSPA4, IKBKG, TGFB1, PIK3CG, PRKAA1, POLR2H, NFKBIB, ATM, IL8, SMAD2, MAP3K14, AKT2, RRAS, RAC1, GTF2F1, STAT3, ERCC2, TAF9B, TAF1, POLR2E, SMARCA2, HSP90AA1, TNF, PIK3R5, GTF2H2, CCL5, NR3C1, SMARCA4, PTGES3, GTF2A2, HSP90B1, POLR2A, NCOA2, MAP3K7, PIK3C3, ANXA1, SOS1, STAT5B, GTF2H3, TAF6, MAPK9, IKBKE, CD3D, POLR2L, POLR2G, MAPK14, FKBP4, NCOR2, PTGS2, NRIP1 |
| PI3K Signaling in B Lymphocytes | 0.000035 | 0.29 | CD81, BLNK, RAF1, FYN, PDIA3, PIK3R1, PDPK1, HRAS, KRAS, PLEKHA4, PLCH2, PLEKHA3, CD79A, PTPRC, BLK, IKBKG, PIK3CG, PLCL1, NFKBIB, PLCD4, CAMK2B, CD19, AKT2, CD79B, ATF1, RRAS, RAC1, IKBKE, PLCL2, MALT1, ATF2, TLR4, CBL, DAPP1, BCL10, SH2B2, VAV1, PIK3AP1, PLEKHA2, CAMK2G, PRKCB |
| Production of Nitric Oxide and Reactive Oxygen Species in Macrophages | 0.000038 | 0.24 | MAP2K4, MAP3K11, PIK3R1, PPP2R3B, SPI1, MAP3K10, IKBKG, RHOG, PPP1R7, CYBA, PIK3CG, NFKBIB, PPP1CA, ATM, MAP3K2, MAP3K14, PTPN6, AKT2, TYK2, RAC1, PPP1R14A, PCYOX1, NCF4, TLR2, PPP2R1A, RHOQ, PPP2R2B, PPP1R12A, TNF, FNBP1, SIRPA, RAP1B, PPP1CC, RHOT2, PIK3R5, MAP3K5, LYZ, MAP3K7, PIK3C3, SERPINA1, MAP3K6, MAPK9, IKBKE, APOL1, TLR4, PPP1R3D, MAPK14, CAT, MAP3K8, MAP3K3, PRKCB |
| ERK/MAPK Signaling | 0.000052 | 0.25 | RAPGEF1, RAF1, BAD, PIK3R1, SOS2, HRAS, PPP2R3B, KRAS, TLN1, CREB5, PPP1R7, PIK3CG, PPP1CA, ATM, RRAS, CRKL, CREB3, RAC1, PPP1R14A, ITGA5, MAPKAPK5, STAT3, RAC3, ELF1, ATF2, PPP2R1A, ARAF, PPP2R2B, PPP1R12A, RAP1B, FYN, PPP1CC, PTK2B, DUSP6, PIK3R5, CRK, DUSP2, EIF4EBP1, KSR1, LAMTOR3, PIK3C3, SOS1, PAK4, PAK2, ATF1, PLA2G4C, ELF2, PPP1R3D, PRKAR2B, PRKCB, PRKAR1A |
| Primary Immunodeficiency Signaling | 0.000065 | 0.34 | BLNK, CD19, IL2RG, CD3E, CD4, IGHG1, RFX5, CD3D, CD79A, PTPRC, IL7R, ICOS, LCK, IKBKG, IGHG3, ZAP70, IGHG4, ADA, IGHM, IGHA1, IGHD |
| Apoptosis Signaling | 0.000072 | 0.31 | MAP2K4, RAF1, BAD, HRAS, KRAS, MAP3K5, IKBKG, NFKBIB, CASP8, BIRC3, MCL1, AIFM1, ENDOG, MAP3K14, CASP3, RRAS, CYCS, BIRC6, APAF1, IKBKE, BAX, CDK1, PARP1, ROCK1, CAPNS1, CASP2, SPTAN1, TNF, BCL2L11 |
| Estrogen Receptor Signaling | 0.000087 | 0.29 | RAF1, TAF11, POLR2F, CCNC, SOS2, GTF2H2, HRAS, KRAS, TAF13, NR3C1, G6PC3, SMARCA4, CTBP1, SPEN, POLR2A, NCOA2, MED15, SOS1, CTBP2, MED16, POLR2H, GTF2H3, MED18, PRKDC, TAF6, RRAS, GTF2F1, ERCC2, MED12, MED27, TAF9B, POLR2L, POLR2G, HDAC3, TAF1, POLR2E, NCOR2, NRIP1, MED24 |
| CD27 Signaling in Lymphocytes | 0.000089 | 0.35 | MAP2K4, MAP3K14, MAP3K11, MAP3K6, CASP3, CYCS, APAF1, MAPK9, IKBKE, MAP3K5, MAP3K10, IKBKG, MAP3K7, IKBKAP, MAP3K8, CASP8, NFKBIB, MAP3K3, MAP2K5, MAP3K2 |
| Huntington's Disease Signaling | 0.000093 | 0.24 | MAP2K4, POLR2F, SGK1, HSPA1A/HSPA1B, PIK3R1, SOS2, HRAS, PDPK1, CREB5, HSPA5, CDK5R1, MAP3K10, GNB4, HSPA4, PIK3CG, VAMP3, POLR2H, ATM, AKT2, HDAC4, CASP3, CYCS, CLTC, CREB3, UBE2S, STX1A, NAPG, GPAA1, ATF2, TAF9B, DYNC1I2, HDAC3, POLR2E, CASP2, PIK3R5, MTOR, POLR2A, ARFIP2, CDK5, PIK3C3, SOS1, CASP8, SDHA, GNAQ, APAF1, MAPK9, BAX, ZDHHC17, RCOR1, POLR2G, POLR2L, CAPNS1, DCTN1, NCOR2, UBC, PRKCB |
| CD28 Signaling in T Helper Cells | 0.000100 | 0.27 | MAP2K4, FYN, CD3E, PIK3R1, CD4, ARPC5, PIK3R5, HLA-DRB1, PDPK1, CDC42, PTPRC, LCK, IKBKG, ACTR3, PIK3CG, PIK3C3, HLA-DRA, NFKBIB, ATM, ACTR2, PTPN6, AKT2, ARPC5L, RAC1, MAPK9, IKBKE, MALT1, CD3D, PTPN11, WAS, BCL10, ZAP70, FCER1G, VAV1, CARD11, LCP2 |
| Acute Myeloid Leukemia Signaling | 0.000129 | 0.32 | MAP2K4, RAF1, BAD, PIK3R1, SOS2, PIK3R5, HRAS, KRAS, SPI1, EIF4EBP1, MTOR, PIK3C3, PIK3CG, SOS1, RPS6KB2, STAT5B, ATM, AKT2, RRAS, STAT3, FLT3LG, CSF1R, CSF2RB, ARAF, TCF7L2, MAP2K5 |
| T Cell Receptor Signaling | 0.000129 | 0.28 | MAP2K4, RAF1, FYN, CD3E, PIK3R1, CD4, SOS2, PIK3R5, HRAS, KRAS, CD8B, PTPRC, LCK, IKBKG, TXK, PIK3CG, PIK3C3, SOS1, ATM, RRAS, RAC1, IKBKE, MALT1, CD3D, CBL, BCL10, PAG1, ZAP70, VAV1, CARD11, LCP2 |
| Renal Cell Carcinoma Signaling | 0.000170 | 0.32 | RAF1, RAPGEF1, AKT2, PAK4, PAK2, RRAS, PIK3R1, SOS2, PIK3R5, RAC1, HRAS, CRK, KRAS, CDC42, ARNT, VEGFA, PTPN11, TGFB1, PIK3CG, PIK3C3, SOS1, FH, UBC, ATM |
| NRF2-mediated Oxidative Stress Response | 0.000186 | 0.26 | MAP2K4, RAF1, PIK3R1, DNAJC3, HRAS, KRAS, CLPP, PIK3CG, ABCC1, KEAP1, JUND, GCLM, CBR1, ACTA1, GSTK1, ATM, RRAS, TXNRD1, GSR, ERP29, SQSTM1, MGST3, GSTP1, MAP2K5, AKR7A2, DNAJC17, PPIB, PRDX1, PIK3R5, MAP3K5, DNAJA1, CUL3, AKR1A1, MAP3K7, PIK3C3, VCP, DNAJC1, SOD1, MAPK9, ACTG1, GSTO1, MAPK14, DNAJB11, CAT, CCT7, GSTO2, DNAJC7, DNAJB5, PRKCB |
| Actin Nucleation by ARP-WASP Complex | 0.000224 | 0.32 | ACTR2, PPP1R12C, ARPC5L, RRAS, RHOT2, SOS2, ARPC5, RAC1, HRAS, ITGA5, KRAS, CDC42, ROCK1, RHOG, ACTR3, RHOQ, WAS, SOS1, BAIAP2, PPP1R12A, FNBP1 |
| Neuregulin Signaling | 0.000234 | 0.28 | RAF1, BAD, PIK3R1, SOS2, HRAS, PDPK1, RPS6, CRK, KRAS, CDK5R1, MTOR, HSP90B1, CDK5, SOS1, RPS6KB2, STAT5B, RNF41, MATK, AKT2, RRAS, CRKL, ITGA5, ERBB2IP, PICK1, PTPN11, HSP90AA1, CDKN1B, PRKCB |
| Ceramide Signaling | 0.000263 | 0.30 | MAP2K4, RAF1, BAD, PIK3R1, PIK3R5, HRAS, PPP2R3B, SMPD1, KRAS, KSR1, SMPD4, SPHK2, PIK3CG, PIK3C3, SPHK1, ATM, AKT2, S1PR5, RRAS, CYCS, CERK, SMPD2, S1PR4, PPP2R1A, PPP2R2B, TNF |
| Sertoli Cell-Sertoli Cell Junction Signaling | 0.000275 | 0.23 | MAP2K4, RAF1, MAP3K11, AXIN1, CTNNA1, MLLT4, HRAS, KRAS, SYMPK, MAP3K5, CDC42, TUBB4A, MAP3K10, AGGF1, MAP3K7, KEAP1, ACTA1, RAB8B, MAP3K2, TUBB1, MAP3K14, EPN1, AKT2, MAP3K6, TUBB4B, RRAS, TUBG1, RAC1, MAPK9, ITGA5, ACTG1, ATF2, EPB41, CLDN5, MAPK14, PRKAR2B, WAS, MAP3K8, TUBA3C/TUBA3D, SPTAN1, CLINT1, MAGI2, TNF, MAP3K3, PRKAR1A |
| tRNA Charging | 0.000288 | 0.17 | CARS, GARS, MARS, FARSB, FARSA, EPRS, RARS, YARS, FARS2, KARS, AARS, VARS, SARS2, SARS |
| Hypoxia Signaling in the Cardiovascular System | 0.000295 | 0.33 | P4HB, UBE2A, UBE2N, UBE2L3, CREB3, BIRC6, UBE2V2, UBE2S, CREB5, UBE2D1, ATF2, ARNT, VEGFA, HSP90B1, UBE2B, HIF1AN, HSP90AA1, NFKBIB, UBE2J1, LDHA, UBE2D3, ATM |
| Integrin Signaling | 0.000295 | 0.25 | MAP2K4, RAPGEF1, RAF1, MAP3K11, PIK3R1, ARPC5, SOS2, HRAS, TLN1, KRAS, RHOG, PIK3CG, TSPAN4, ACTA1, ATM, ACTR2, AKT2, RRAS, CRKL, RAC1, ITGA5, TNK2, RAC3, MYL12A, RHOQ, ZYX, PPP1R12A, FNBP1, RAP1B, FYN, RAP2A, ITGA2B, RHOT2, PIK3R5, CRK, CDC42, ITGB7, ARF6, ACTR3, PIK3C3, SOS1, PAK4, PAK2, ASAP1, ARPC5L, GIT1, ACTG1, ROCK1, CAPNS1, WAS, LIMS1 |
| Granzyme B Signaling | 0.000339 | 0.56 | ENDOG, PRKDC, PRF1, CASP3, CYCS, APAF1, CASP8, GZMB, PARP1 |
| Fcγ Receptor-mediated Phagocytosis in Macrophages and Monocytes | 0.000363 | 0.28 | GAB2, FYN, PTK2B, PIK3R1, ARPC5, CRK, TLN1, CDC42, ARF6, ACTR3, EZR, PIK3CG, RPS6KB2, VAMP3, ACTA1, ACTR2, AKT2, PLD3, ARPC5L, RAC1, FYB, RAC3, ACTG1, PIP5K1A, CBL, WAS, VAV1, LCP2, PRKCB |
| PPARα/RXRα Activation | 0.000427 | 0.25 | MAP2K4, RAF1, TGFBR1, PRKAB2, PDIA3, SOS2, HRAS, KRAS, ACVR2B, PLCH2, GK, TGFBR2, IKBKG, HSP90B1, TGFB1, MAP3K7, FASN, SOS1, PRKAA1, PLCL1, NFKBIB, GOT2, STAT5B, PLCD4, IL1RAP, SMAD2, MAP3K14, RRAS, ACOX1, CKAP5, GNAQ, NR2C2, IKBKE, PLCL2, MED12, ACVR1B, AIP, MAPK14, PRKAR2B, HSP90AA1, NCOR2, ADCY7, MED24, PRKAR1A, PRKCB |
| LPS-stimulated MAPK Signaling | 0.000447 | 0.29 | MAP2K4, MAP3K14, RAF1, ATF1, RRAS, PIK3R1, PIK3R5, RAC1, HRAS, MAPK9, IKBKE, KRAS, MAP3K5, CDC42, ATF2, TLR4, IKBKG, MAPK14, MAP3K7, PIK3C3, PIK3CG, NFKBIB, ATM, PRKCB |
| Fcγ_RIIB Signaling in B Lymphocytes | 0.000479 | 0.27 | MAP2K4, BLNK, CD79B, RRAS, PIK3R1, PIK3R5, HRAS, PDPK1, MAPK9, KRAS, CD79A, PIK3CG, PIK3C3, SOS1, DOK1, ATM |
| Angiopoietin Signaling | 0.000490 | 0.30 | PAK4, AKT2, GRB14, PAK2, BAD, RRAS, DOK2, PIK3R1, PIK3R5, HRAS, IKBKE, CRK, KRAS, IKBKG, PTPN11, PIK3CG, PIK3C3, SOS1, IKBKAP, STAT5B, NFKBIB, ATM |
| iCOS-iCOSL Signaling in T Helper Cells | 0.000513 | 0.27 | GAB2, CD3E, BAD, PIK3R1, CD4, PIK3R5, TRAT1, HLA-DRB1, PDPK1, PLEKHA4, PLEKHA3, PTPRC, IKBKG, LCK, PIK3CG, PIK3C3, HLA-DRA, NFKBIB, CAMK2B, ATM, IL2RG, AKT2, RAC1, IKBKE, CD3D, ICOS, ZAP70, FCER1G, VAV1, IL2RA, PLEKHA2, LCP2, CAMK2G |
| IL-2 Signaling | 0.000513 | 0.33 | RAF1, IL2RG, AKT2, PTK2B, RRAS, PIK3R1, SOS2, PIK3R5, HRAS, KRAS, LCK, PTPN11, PIK3CG, PIK3C3, SOS1, CSNK2A1, IL2RA, STAT5B, ATM |
| TCA Cycle II (Eukaryotic) | 0.000550 | 0.24 | SDHA, SUCLG1, ACO2, DLST, IDH3A, MDH1, FH, OGDH, ACO1, IDH3B |
| DNA Double-Strand Break Repair by Non-Homologous End Joining | 0.000646 | 0.42 | PRKDC, XRCC6, LIG4, XRCC5, PARP1, ATM, XRCC1, NBN |
| Regulation of IL-2 Expression in Activated and Anergic T Lymphocytes | 0.000661 | 0.28 | MAP2K4, FYN, RAF1, TGFBR1, CD3E, SOS2, HRAS, KRAS, TGFBR2, IKBKG, TGFB1, SOS1, NFKBIB, SMAD2, RRAS, RAC1, TOB1, MAPK9, IKBKE, MALT1, CD3D, BCL10, ZAP70, VAV1, CARD11 |
| JAK/Stat Signaling | 0.000776 | 0.31 | RAF1, PTPN6, AKT2, RRAS, PIK3R1, TYK2, SOS2, GNAQ, PIK3R5, HRAS, SOCS4, KRAS, STAT3, MTOR, PIAS4, PTPN11, PIK3CG, PIK3C3, SOS1, STAT5B, SOCS5, ATM |
| Molecular Mechanisms of Cancer | 0.000776 | 0.21 | MAP2K4, RAPGEF1, GAB2, RAF1, TGFBR1, BAD, AXIN1, SUV39H1, PIK3R1, SOS2, HRAS, NCSTN, KRAS, ARHGEF1, RBL1, TGFBR2, RHOG, GNA15, TGFB1, PIK3CG, GNA13, NFKBIB, BIRC3, BMP8B, ATM, SMAD2, AKT2, CASP3, RRAS, CYCS, TYK2, RAC1, RAC3, CBL, RHOQ, PTPN11, CDK2, FNBP1, CAMK2G, RAP1B, RAP2A, FYN, RHOT2, CTNNA1, PIK3R5, CRK, PSENEN, MAP3K5, CDC42, E2F3, SYNGAP1, LAMTOR3, NLK, BBC3, MAP3K7, PIK3C3, SOS1, ARHGEF3, CASP8, CAMK2B, PRKDC, PAK4, PMAIP1, PAK2, HAT1, DVL1, APAF1, GNAQ, MAPK9, BAX, NBN, MAPK14, PRKAR2B, CDKN1B, ADCY7, BCL2L11, PRKAR1A, PRKCB |
| 14-3-3-mediated Signaling | 0.000794 | 0.27 | MAP2K4, TSC1, RAF1, BAD, PDIA3, PIK3R1, PIK3R5, HRAS, KRAS, MAP3K5, PLCH2, TUBB4A, PIK3CG, PIK3C3, PLCL1, PDCD6IP, PLCD4, ATM, TUBB1, AKT2, RRAS, TUBB4B, TUBG1, MAPK9, VIM, BAX, PLCL2, CBL, TUBA3C/TUBA3D, CDKN1B, TNF, PRKCB |
| CD40 Signaling | 0.000813 | 0.30 | MAP2K4, MAP3K14, TRAF3, ATF1, PIK3R1, PIK3R5, TNFAIP3, MAPK9, IKBKE, STAT3, TANK, IKBKG, MAPK14, MAP3K7, PIK3CG, PIK3C3, IKBKAP, NFKBIB, MAP2K5, FCER2, ATM |
| ERK5 Signaling | 0.000813 | 0.32 | IL6ST, SH2D2A, BAD, SGK1, RRAS, CREB3, GNAQ, RPS6KA3, MEF2A, HRAS, KRAS, CREB5, ATF2, PTPN11, RPS6KB2, MAP3K8, GNA13, WNK1, MAP3K3, MAP2K5, MAP3K2 |
| EIF2 Signaling | 0.001000 | 0.22 | RPL24, PPP1CC, RAF1, EIF2S2, EIF2B4, PIK3R1, SOS2, PIK3R5, PDPK1, HRAS, RPS6, KRAS, EIF3C/EIF3CL, EIF2B2, EIF2C4, RPL6, EIF3B, EIF1AX, PIK3C3, PIK3CG, SOS1, EIF5, RPL19, RPL12, RPL8, PPP1CA, RPL13, ATM, PABPC1, AKT2, RRAS, RPS28, RPS19, EIF3E, EIF3G, RPS5, RPS26, RPL28, RPL26L1, EIF4A3, EIF2B5, EIF3I, RPS15, EIF3K |
| CTLA4 Signaling in Cytotoxic T Lymphocytes | 0.001023 | 0.28 | FYN, CD3E, PIK3R1, CD4, CLTB, PIK3R5, TRAT1, HLA-DRB1, PPP2R3B, LCK, PIK3C3, PIK3CG, HLA-DRA, ATM, AKT2, PTPN6, AP1M1, CLTC, CD3D, PPP2R1A, PTPN11, CLTA, PPP2R2B, ZAP70, FCER1G, AP1G1, LCP2 |
| Assembly of RNA Polymerase II Complex | 0.001175 | 0.32 | POLR2F, TAF11, TAF6, GTF2H2, GTF2F1, GTF2E2, ERCC2, TAF13, TAF9B, POLR2G, GTF2A2, POLR2L, TAF1, POLR2A, POLR2E, DR1, POLR2H, GTF2H3 |
| CNTF Signaling | 0.001175 | 0.31 | IL6ST, RAF1, RRAS, PIK3R1, TYK2, RPS6KA3, PIK3R5, HRAS, KRAS, STAT3, MTOR, PTPN11, PIK3C3, PIK3CG, SOS1, RPS6KB2, ATM |
| Prostate Cancer Signaling | 0.001202 | 0.26 | RAF1, BAD, SUV39H1, PIK3R1, SOS2, PIK3R5, HRAS, PDPK1, KRAS, CREB5, MTOR, HSP90B1, PIK3CG, PIK3C3, SOS1, NFKBIB, ATM, AKT2, RRAS, CREB3, ATF2, HSP90AA1, CDKN1B, GSTP1, CDK2 |
| Rac Signaling | 0.001230 | 0.25 | MAP2K4, RAF1, MAP3K11, PTK2B, PIK3R1, ARPC5, PIK3R5, PIKFYVE, HRAS, KRAS, CDC42, IQGAP1, PARD6A, CDK5R1, ANK1, ACTR3, ARFIP2, PIK3CG, PIK3C3, BAIAP2, ATM, ACTR2, PAK4, PAK2, ARPC5L, RRAS, RAC1, ITGA5, PIP5K1A, SH3RF1 |
| NF-KB Signaling | 0.001514 | 0.25 | SIGIRR, AZI2, TRAF3, TGFBR1, PIK3R1, UBE2N, TLR8, PIK3R5, TNFAIP3, HRAS, KRAS, TANK, TGFBR2, IKBKG, LCK, TLR10, MAP3K7, PIK3C3, PIK3CG, CSNK2A1, TDP2, NFKBIB, CASP8, ATM, MAP3K14, AKT2, RRAS, RELB, MALT1, TAB3, TLR2, TLR4, RIPK1, BCL10, ZAP70, FCER1G, MAP3K8, TIRAP, TNF, MAP3K3, CARD11, IRAK4, PRKCB |
| mTOR Signaling | 0.001585 | 0.21 | TSC1, PRKAB2, PIK3R1, RHOT2, PIK3R5, RPS6KA3, PDPK1, HRAS, PPP2R3B, RPS6, KRAS, RICTOR, EIF3C/EIF3CL, EIF4EBP1, VEGFA, MTOR, RHOG, EIF3B, PIK3C3, PIK3CG, PRKAA1, RPS6KB2, EIF4B, ATM, RHEB, AKT2, PLD3, RRAS, RPS28, RPS19, RAC1, EIF3E, EIF3G, DGKZ, PPP2R1A, RHOQ, RPS5, RPS26, EIF4A3, PPP2R2B, EIF3I, RPS15, FNBP1, EIF3K, PRKCB |
| FAK Signaling | 0.001778 | 0.25 | FYN, RAF1, PIK3R1, SOS2, PIK3R5, HRAS, PDPK1, CRK, TLN1, KRAS, PIK3C3, PIK3CG, SOS1, ACTA1, ATM, PAK4, AKT2, PAK2, RRAS, ASAP1, RAC1, ITGA5, ACTG1, CAPNS1, WAS |
| Myc Mediated Apoptosis Signaling | 0.001778 | 0.32 | MAP2K4, AKT2, CASP3, BAD, CYCS, RRAS, PIK3R1, SOS2, PIK3R5, APAF1, HRAS, MAPK9, KRAS, BAX, PIK3CG, PIK3C3, SOS1, CASP8, ATM |
| IL-3 Signaling | 0.001820 | 0.30 | GAB2, RAPGEF1, RAF1, PTPN6, AKT2, IL3RA, BAD, RRAS, PIK3R1, CRKL, PIK3R5, RAC1, HRAS, KRAS, STAT3, CSF2RB, PIK3CG, PIK3C3, SOS1, STAT5B, ATM, PRKCB |
| PI3K/AKT Signaling | 0.001862 | 0.23 | RAF1, GAB2, TSC1, BAD, PIK3R1, SOS2, PDPK1, HRAS, PPP2R3B, KRAS, MAP3K5, EIF4EBP1, MTOR, HSP90B1, IKBKG, PIK3CG, SOS1, RPS6KB2, NFKBIB, MCL1, RHEB, AKT2, RRAS, TYK2, ITGA5, IKBKE, PPP2R1A, LIMS1, PPP2R2B, HSP90AA1, MAP3K8, PTGS2, CDKN1B |
| Paxillin Signaling | 0.001905 | 0.25 | MAP2K4, ITGA2B, PTK2B, PIK3R1, SOS2, PIK3R5, HRAS, KRAS, CRK, TLN1, CDC42, PTPN12, ITGB7, ARF6, ARFIP2, PIK3CG, PIK3C3, SOS1, ACTA1, ATM, PAK4, PAK2, RRAS, RAC1, ITGA5, MAPK9, ACTG1, MAPK14 |
| Ephrin Receptor Signaling | 0.001950 | 0.22 | RAP1B, RAF1, FYN, RAPGEF1, AXIN1, ARPC5, SOS2, HRAS, KRAS, CRK, CDC42, CREB5, EPHB6, VEGFA, GNB4, EFNB2, ACTR3, GNA15, PIK3CG, SOS1, GNA13, ACTR2, MAP3K14, GRIN3B, AKT2, PAK4, PAK2, KALRN, ARPC5L, RRAS, CRKL, SH2D3C, CREB3, RAC1, GNAQ, ITGA5, STAT3, RAC3, ATF2, ROCK1, ABI1, PTPN11, WAS, DOK1 |
| Mevalonate Pathway I | 0.002291 | 0.25 | MVD, HADHB, PMVK, IDI1, ACAT1, HMGCR, HMGCS1 |
| VEGF Signaling | 0.002512 | 0.25 | EIF2S2, RAF1, EIF2B4, PTK2B, BAD, PIK3R1, SOS2, PIK3R5, HRAS, KRAS, EIF2B2, ARNT, VEGFA, EIF1AX, PIK3CG, PIK3C3, SOS1, ACTA1, ATM, AKT2, RRAS, ACTG1, ROCK1, EIF2B5, PRKCB |
| p70S6K Signaling | 0.002754 | 0.25 | RAF1, BAD, PDIA3, PIK3R1, SOS2, PIK3R5, PDPK1, HRAS, PPP2R3B, RPS6, KRAS, PLCH2, CD79A, MTOR, F2RL1, PIK3CG, PIK3C3, SOS1, EEF2K, PLCL1, PLCD4, ATM, CD19, IL2RG, AKT2, CD79B, RRAS, GNAQ, PLCL2, PPP2R1A, PPP2R2B, PRKCB |
| RANK Signaling in Osteoclasts | 0.002951 | 0.26 | MAP2K4, RAF1, MAP3K11, PTK2B, PIK3R1, PIK3R5, MAP3K5, MAP3K10, IKBKG, MAP3K7, PIK3CG, PIK3C3, NFKBIB, BIRC3, MAP3K2, ATM, MAP3K14, AKT2, MAP3K6, MAPK9, IKBKE, MAPK14, CBL, MAP3K8, MAP3K3 |
| Tumoricidal Function of Hepatic Natural Killer Cells | 0.003090 | 0.42 | ENDOG, PRF1, SRGN, CASP3, CYCS, APAF1, BAX, CASP8, GZMB, AIFM1 |
| Natural Killer Cell Signaling | 0.003236 | 0.25 | RAF1, FYN, KLRD1, PIK3R1, SOS2, PIK3R5, HRAS, NCR3, KRAS, LCK, PIK3C3, PIK3CG, SOS1, HCST, ATM, PAK4, AKT2, PTPN6, PAK2, RRAS, RAC1, RAC3, PTPN11, CD244, ZAP70, FCER1G, VAV1, LCP2, PRKCB |
| Cdc42 Signaling | 0.003388 | 0.20 | MAP2K4, RAF1, MAP3K11, CD3E, HLA-A, ARPC5, HLA-DRB1, CDC42, IQGAP1, PARD6A, ACTR3, HLA-DRB3, BAIAP2, HLA-DRA, HLA-B, EXOC5, HLA-F, HLA-DPB1, ACTR2, PAK4, PAK2, ARPC5L, ITGA5, MAPK9, TNK2, CD3D, ATF2, MYL12A, MAPK14, EXOC8, HLA-DRB4, WAS, FCER1G, PPP1R12A, VAV1 |
| Role of JAK1 and JAK3 inβc Cytokine Signaling | 0.003388 | 0.28 | BLNK, IL2RG, PTK2B, FES, RRAS, PIK3R1, PIK3R5, HRAS, KRAS, STAT3, IL7, IL7R, PTPN11, PIK3C3, PIK3CG, SH2B2, IL2RA, STAT5B, ATM |
| Cardiac Hypertrophy Signaling | 0.003548 | 0.21 | MAP2K4, RAF1, TGFBR1, MAP3K11, PIK3R1, HRAS, KRAS, PLCH2, TGFBR2, MAP3K10, GNB4, RHOG, GNA15, TGFB1, PIK3CG, GNA13, PLCL1, ATM, MAP3K2, MAP3K14, RRAS, IL6R, PLCL2, CACNA1A, ATF2, MYL12A, RHOQ, EIF2B5, FNBP1, EIF2B4, PDIA3, RHOT2, PIK3R5, MAP3K5, EIF2B2, MTOR, MAP3K7, PIK3C3, SOS1, PLCD4, MAP3K6, MAPKAPK3, MEF2A, GNAQ, MAPK9, ROCK1, MAPK14, PRKAR2B, MAP3K8, MAP3K3, ADCY7, PRKAR1A |
| Cytotoxic T Lymphocyte-mediated Apoptosis of Target Cells | 0.003890 | 0.20 | CASP3, CD3E, CYCS, HLA-A, APAF1, HLA-DRB1, CD3D, PRF1, HLA-DRB4, HLA-DRB3, HLA-DRA, FCER1G, HLA-B, CASP8, HLA-F, HLA-DPB1, GZMB |
| Melanocyte Development and Pigmentation Signaling | 0.003890 | 0.26 | RAF1, PTPN6, RRAS, PIK3R1, CREB3, SOS2, RPS6KA3, PIK3R5, HRAS, CRK, POMC, KRAS, CREB5, ATF2, PRKAR2B, PTPN11, PIK3CG, PIK3C3, SOS1, SH2B2, RPS6KB2, ADCY7, PRKAR1A, ATM |
| Virus Entry via Endocytic Pathways | 0.004169 | 0.25 | FLNB, FYN, HLA-A, PIK3R1, CLTB, PIK3R5, HRAS, KRAS, CDC42, ITGB7, FLNA, PIK3C3, PIK3CG, HLA-B, ACTA1, ATM, RRAS, CLTC, RAC1, ITGA5, RAC3, ACTG1, CLTA, TFRC, PRKCB |
| Renin-Angiotensin Signaling | 0.004365 | 0.23 | MAP2K4, RAF1, PTK2B, PIK3R1, SOS2, PIK3R5, HRAS, KRAS, CCL5, PIK3C3, PIK3CG, SOS1, ATM, PAK4, PTPN6, PAK2, RRAS, GNAQ, RAC1, MAPK9, STAT3, ATF2, MAPK14, PRKAR2B, SHC2, ADCY7, TNF, PRKCB, PRKAR1A |
| Sphingosine-1-phosphate Signaling | 0.004365 | 0.24 | PTK2B, PDIA3, PIK3R1, RHOT2, PIK3R5, SMPD1, PLCH2, RHOG, SMPD4, PIK3C3, PIK3CG, SPHK1, GNA13, PLCL1, CASP8, PLCD4, ATM, AKT2, S1PR5, CASP3, GNAQ, RAC1, PLCL2, SMPD2, S1PR4, RHOQ, CASP2, ADCY7, FNBP1 |
| Type I Diabetes Mellitus Signaling | 0.004365 | 0.24 | MAP2K4, CD3E, HLA-A, HLA-DRB1, MAP3K5, TRADD, IKBKG, PRF1, MAP3K7, HLA-DRA, HLA-B, HLA-F, CASP8, NFKBIB, IL1RAP, MAP3K14, CASP3, CYCS, APAF1, MAPK9, SOCS4, IKBKE, CD3D, RIPK1, MAPK14, FCER1G, TNF, SOCS5, GZMB |
| Ascorbate Recycling (Cytosolic) | 0.004467 | 0.23 | GSTO2, GLRX, GSTO1 |
| OX40 Signaling Pathway | 0.004467 | 0.19 | MAP2K4, TNFSF4, TRAF3, TNFRSF4, CD3E, HLA-A, CD4, HLA-DRB1, MAPK9, CD3D, HLA-DRB4, HLA-DRB3, HLA-DRA, HLA-B, FCER1G, HLA-F, NFKBIB, HLA-DPB1 |
| Neurotrophin/TRK Signaling | 0.004677 | 0.27 | MAP2K4, RAF1, RRAS, PIK3R1, CREB3, SOS2, PIK3R5, HRAS, PDPK1, KRAS, MAP3K5, CDC42, CREB5, ATF2, PTPN11, PIK3C3, PIK3CG, SOS1, MAP2K5, ATM |
| Regulation of Actin-based Motility by Rho | 0.004677 | 0.26 | ACTR2, PAK4, PFN1, PAK2, ARPC5L, RHOT2, ARPC5, RAC1, PIKFYVE, CDC42, RAC3, ROCK1, PIP5K1A, MYL12A, RHOG, ACTR3, RHOQ, WAS, BAIAP2, PPP1R12A, ARHGDIA, FNBP1, ACTA1 |
| TWEAK Signaling | 0.004677 | 0.32 | MAP3K14, TRADD, TRAF3, IKBKG, RIPK1, CASP3, CYCS, APAF1, IKBKE, CASP8, NFKBIB, BIRC3 |
| Lymphotoxin Beta Receptor Signaling | 0.004898 | 0.28 | MAP3K14, TRAF3, AKT2, CASP3, CYCS, RELB, PIK3R1, PIK3R5, APAF1, PDPK1, LTB, IKBKE, IKBKG, PIK3C3, PIK3CG, IKBKAP, ATM |
| GM-CSF Signaling | 0.005129 | 0.28 | RAF1, AKT2, RRAS, PIK3R1, SOS2, PIK3R5, HRAS, KRAS, STAT3, CSF2RB, PTPN11, PIK3CG, PIK3C3, SOS1, STAT5B, ATM, CAMK2B, CAMK2G, PRKCB |
| IL-4 Signaling | 0.005129 | 0.27 | HMGA1, PTPN6, IL2RG, AKT2, RRAS, PIK3R1, TYK2, SOS2, PIK3R5, HRAS, HLA-DRB1, KRAS, NR3C1, MTOR, PIK3CG, PIK3C3, HLA-DRA, SOS1, RPS6KB2, FCER2, ATM |
| Hereditary Breast Cancer Signaling | 0.005248 | 0.23 | POLR2F, PIK3R1, PIK3R5, SMARCD2, HRAS, KRAS, SMARCA4, POLR2A, PIK3C3, PIK3CG, RFC2, POLR2H, SLC19A1, BLM, ATM, AKT2, C17orf70, HDAC4, RRAS, FANCG, TUBG1, CDK1, NBN, POLR2L, POLR2G, HDAC3, POLR2E, SMARCA2, H2AFX, UBC |
| Induction of Apoptosis by HIV1 | 0.005495 | 0.28 | MAP2K4, MAP3K14, CASP3, CYCS, APAF1, MAPK9, IKBKE, MAP3K5, BAX, TRADD, IKBKG, RIPK1, BBC3, IKBKAP, CASP8, NFKBIB, TNF, BIRC3 |
| GNRH Signaling | 0.006166 | 0.22 | MAP2K4, RAF1, MAP3K11, SOS2, HRAS, KRAS, MAP3K5, CDC42, CREB5, MAP3K10, MAP3K7, SOS1, MAP3K2, CAMK2B, MAP3K14, PAK4, PAK2, MAP3K6, RRAS, CREB3, GNAQ, RAC1, MAPK9, ATF2, PRKAR2B, MAPK14, MAP3K8, ADCY7, MAP3K3, CAMK2G, PRKAR1A, PRKCB |
| B Cell Development | 0.006310 | 0.33 | IL7R, PTPRC, CD19, CD79B, HLA-DRA, HLA-DRB1, IGHM, DNTT, IL7, CD79A, IGHD |
| PAK Signaling | 0.006310 | 0.23 | MAP2K4, RAF1, PAK4, PAK2, ARHGAP10, PTK2B, CASP3, RRAS, PIK3R1, SOS2, PIK3R5, RAC1, MAPK9, ITGA5, HRAS, KRAS, CDC42, GIT1, MYL12A, PIK3CG, PIK3C3, SOS1, TNF, ATM |
| IL-6 Signaling | 0.006761 | 0.24 | MAP2K4, IL6ST, RAF1, PIK3R1, SOS2, PIK3R5, HRAS, KRAS, VEGFA, IKBKG, MAP3K7, PIK3C3, PIK3CG, SOS1, CSNK2A1, NFKBIB, IL1RAP, ATM, MCL1, IL8, MAP3K14, AKT2, RRAS, IL6R, MAPK9, IKBKE, STAT3, MAPK14, PTPN11, TNF |
| ErbB2-ErbB3 Signaling | 0.007244 | 0.28 | RAF1, BAD, RRAS, PIK3R1, TYK2, SOS2, PIK3R5, HRAS, PDPK1, KRAS, STAT3, PIK3CG, PIK3C3, SOS1, CDKN1B, STAT5B, ATM |
| Endometrial Cancer Signaling | 0.007943 | 0.28 | RAF1, AKT2, BAD, RRAS, AXIN1, PIK3R1, SOS2, PIK3R5, CTNNA1, HRAS, PDPK1, KRAS, PIK3C3, PIK3CG, SOS1, ATM |
| 4-1BB Signaling in T Lymphocytes | 0.008318 | 0.31 | MAP2K4, MAP3K14, IKBKG, TNFRSF9, MAPK14, MAPK9, IKBKE, IKBKAP, MAP3K5, NFKBIB, ATF2 |
| Breast Cancer Regulation by Stathmin1 | 0.008913 | 0.21 | RAF1, PPP1CC, PIK3R1, SOS2, PIK3R5, HRAS, PPP2R3B, KRAS, ARHGEF1, CDC42, TUBB4A, E2F3, GNB4, PPP1R7, PIK3CG, PIK3C3, SOS1, RB1CC1, GNA13, ARHGEF3, PPP1CA, CAMK2B, ATM, TUBB1, TUBB4B, RRAS, TUBG1, RAC1, GNAQ, PPP1R14A, CDK1, ROCK1, PPP1R3D, PPP2R1A, PRKAR2B, PPP2R2B, PPP1R12A, TUBA3C/TUBA3D, CDKN1B, ADCY7, CDK2, PRKAR1A, CAMK2G, PRKCB |
| Role of MAPK Signaling in the Pathogenesis of Influenza | 0.009550 | 0.26 | PLA2G16, MAP2K4, RAF1, AKT2, CASP3, RRAS, PLA2G4C, HRAS, MAPK9, KRAS, CCL5, BAX, MAP3K5, ATF2, MAPK14, RARRES3, PTGS2, TNF |
| Aldosterone Signaling in Epithelial Cells | 0.010233 | 0.21 | RAF1, DNAJC17, PDIA3, HSPA1A/HSPA1B, SGK1, PIK3R1, SOS2, PIK3R5, PIKFYVE, DNAJC3, PDPK1, KRAS, HSPA5, DNAJA1, PLCH2, HSPA4, HSP90B1, PIK3CG, PIK3C3, SOS1, DNAJC1, PLCL1, DNAJC30, PLCD4, ATM, HSPH1, DNAJC2, PLCL2, PIP5K1A, DNAJC24, DNAJB11, HSP90AA1, DNAJB5, DNAJC7, PRKCB |
| Antigen Presentation Pathway | 0.010233 | 0.30 | HLA-DRB4, HLA-A, PDIA3, HLA-DRB3, HLA-DRA, HLA-B, HLA-DRB1, PSMB8, CD74, HLA-F, HLA-DPB1, PSMB6 |
| Death Receptor Signaling | 0.010471 | 0.27 | MAP2K4, MAP3K14, CASP3, CYCS, APAF1, IKBKE, MAP3K5, TANK, TRADD, IKBKG, RIPK1, CRADD, CASP2, CASP8, NFKBIB, TNF, BIRC3 |
| IL-17 Signaling | 0.010965 | 0.27 | MAP2K4, IL8, MAP3K14, AKT2, RRAS, PIK3R1, PIK3R5, HRAS, MAPK9, KRAS, CXCL5, IL17RA, ATF2, MAPK14, MAP3K7, TIMP1, PIK3C3, PIK3CG, PTGS2, ATM |
| PTEN Signaling | 0.010965 | 0.22 | RAF1, FOXO4, TGFBR1, BAD, PIK3R1, SOS2, PIK3R5, PDPK1, HRAS, KRAS, CDC42, TGFBR2, IKBKG, PIK3CG, SOS1, CSNK2A1, RPS6KB2, MCRS1, AKT2, CASP3, RRAS, RAC1, ITGA5, IKBKE, RAC3, CBL, CDKN1B, MAGI2, BCL2L11 |
| Type II Diabetes Mellitus Signaling | 0.010965 | 0.18 | MAP2K4, PRKAB2, PIK3R1, PKM, PIK3R5, PDPK1, SMPD1, MAP3K5, SLC27A4, TRADD, IKBKG, MTOR, SMPD4, MAP3K7, PIK3CG, PIK3C3, PRKAA1, NFKBIB, ATM, MAP3K14, AKT2, MAPK9, SOCS4, IKBKE, SMPD2, SH2B2, TNF, SOCS5, PRKCB |
| Role of Tissue Factor in Cancer | 0.012023 | 0.24 | FYN, PTK2B, PIK3R1, RPS6KA3, PIK3R5, HRAS, KRAS, CDC42, BLK, VEGFA, LCK, MTOR, F2RL1, PIK3CG, PIK3C3, GNA13, STAT5B, ATM, IL8, P4HB, AKT2, CASP3, RRAS, GNAQ, RAC1, MAPK14, PTPN11 |
| ATM Signaling | 0.012589 | 0.28 | MAP2K4, SMC3, TRIM28, CREB3, MAPK9, CREB5, CDK1, ATF2, NBN, MDM4, MAPK14, H2AFX, BRAT1, TLK2, BLM, CDK2, ATM |
| NF-KB Activation by Viruses | 0.012882 | 0.24 | MAP3K14, RAF1, AKT2, RRAS, CD4, PIK3R1, PIK3R5, HRAS, ITGA5, IKBKE, KRAS, IKBKG, LCK, RIPK1, PIK3C3, PIK3CG, IKBKAP, NFKBIB, ATM, PRKCB |
| Prolactin Signaling | 0.012882 | 0.25 | FYN, RAF1, RRAS, PIK3R1, SOS2, PIK3R5, HRAS, PDPK1, SOCS4, KRAS, STAT3, NR3C1, PTPN11, PIK3C3, PIK3CG, SOS1, STAT5B, SOCS5, ATM, PRKCB |
| Role of JAK2 in Hormone-like Cytokine Signaling | 0.013804 | 0.31 | SH2B3, SH2B1, PTPN6, PTPN11, SH2B2, TYK2, SOCS4, STAT3, STAT5B, SOCS5, SIRPA |
| HMGB1 Signaling | 0.014791 | 0.24 | MAP2K4, IL8, AKT2, AGER, HAT1, RRAS, PIK3R1, RHOT2, PIK3R5, RAC1, HRAS, MAPK9, KRAS, CDC42, TLR4, RHOG, MAPK14, RHOQ, PIK3CG, PIK3C3, TNF, FNBP1, MAP2K5, ATM |
| Prostanoid Biosynthesis | 0.014791 | 0.31 | PTGES2, PTGS1, PTGS2, TBXAS1, PTGES3 |
| RAR Activation | 0.014791 | 0.21 | MAP2K4, AKR1C3, PIK3R1, SMARCD2, GTF2H2, PDPK1, MAP3K5, PSMC5, SMARCA4, VEGFA, PRMT1, TGFB1, PNRC1, PIK3CG, CSNK2A1, NR2F6, STAT5B, PNPLA4, GTF2H3, SMAD2, AKT2, IL3RA, NRIP2, RAC1, MAPK9, ERCC2, PARP1, CSF2RB, RBP7, MAPK14, PRKAR2B, DHRS9, SMARCA2, NCOR2, NRIP1, RBP5, ADCY7, SCAND1, PRKAR1A, PRKCB |
| Role of JAK family kinases in IL-6-type Cytokine Signaling | 0.014791 | 0.33 | MAP2K4, IL6ST, MAPK14, PTPN11, IL6R, TYK2, MAPK9, STAT3, STAT5B |
| Pyridoxal 5'-phosphate Salvage Pathway | 0.015136 | 0.24 | MAP2K4, DAPK1, AKT2, PAK2, MAP3K6, SGK1, DMPK, MAPK6, MAPK9, CDK1, PKN1, ARAF, CDK5, PRKAA1, MAP3K8, HIPK1, CDK2 |
| Salvage Pathways of Pyrimidine Ribonucleotides | 0.015488 | 0.21 | MAP2K4, DAPK1, AKT2, PAK2, MAP3K6, SGK1, DMPK, MAPK6, MAPK9, PKN1, CDK1, APOBEC3A, NME3, ARAF, CDK5, APOBEC3H, PRKAA1, CMPK1, MAP3K8, HIPK1, CDK2 |
| Chondroitin and Dermatan Biosynthesis | 0.015849 | 0.31 | CSGALNACT2, CHSY1, CHPF, CSGALNACT1 |
| Methylmalonyl Pathway | 0.015849 | 0.25 | PCCA, PCCB, MUT |
| Pentose Phosphate Pathway (Oxidative Branch) | 0.015849 | 0.27 | PGD, PGLS, H6PD |
| Docosahexaenoic Acid (DHA) Signaling | 0.016218 | 0.25 | AKT2, CASP3, BAD, CYCS, PIK3CG, PIK3R1, PIK3C3, APAF1, PIK3R5, PDPK1, BAX, ATM |
| Glycoaminoglycan-protein Linkage Region Biosynthesis | 0.016982 | 0.27 | XYLT2, B3GALT6, B3GAT3, B3GAT2 |
| Nucleotide Excision Repair Pathway | 0.017378 | 0.31 | POLR2G, POLR2L, POLR2F, POLR2A, POLR2E, GTF2H2, POLR2H, ERCC2, RAD23B, XPA, GTF2H3 |
| Estrogen-Dependent Breast Cancer Signaling | 0.017783 | 0.24 | HSD17B10, AKT2, RRAS, PIK3R1, CREB3, PIK3R5, HRAS, KRAS, CREB5, ATF2, HSD17B8, PIK3CG, PIK3C3, STAT5B, HSD17B4, HSD17B1, ATM |
| IL-15 Signaling | 0.017783 | 0.25 | RAF1, IL2RG, AKT2, RRAS, PIK3R1, TYK2, PIK3R5, HRAS, KRAS, STAT3, RAC3, LCK, MAPK14, PIK3C3, PIK3CG, STAT5B, ATM |
| Role of NFAT in Regulation of the Immune Response | 0.018621 | 0.19 | BLNK, RAF1, FYN, CD3E, PIK3R1, CD4, SOS2, PIK3R5, HLA-DRB1, HRAS, KRAS, CD79A, CABIN1, GNB4, IKBKG, LCK, GNA15, PIK3CG, PIK3C3, SOS1, HLA-DRA, GNA13, NFKBIB, ORAI1, ATM, AKT2, CD79B, RRAS, CSNK1G3, MEF2A, GNAQ, IKBKE, CD3D, ATF2, ZAP70, FCER1G, IKBKAP, LCP2 |
| Mouse Embryonic Stem Cell Pluripotency | 0.019055 | 0.24 | IL6ST, RAF1, ID2, AKT2, RRAS, AXIN1, PIK3R1, TYK2, DVL1, SOS2, PIK3R5, HRAS, KRAS, STAT3, MAPK14, PTPN11, MAP3K7, PIK3C3, PIK3CG, SOS1, DVL3, DVL2, TCF7L2, ATM |
| Antiproliferative Role of TOB in T Cell Signaling | 0.019498 | 0.35 | PABPC1, TGFBR2, SMAD2, TGFBR1, TGFB1, CUL1, TOB1, CDKN1B, CDK2 |
| Eicosanoid Signaling | 0.019498 | 0.20 | PLA2G16, PTGIR, AKR1C3, PLA2G4C, TBXA2R, PTGS1, FPR2, ALOX15B, PTGES2, RARRES3, CYSLTR1, PTGS2, ALOX5, TBXAS1, CYSLTR2, DPEP2 |
| Glycolysis I | 0.019498 | 0.20 | PGK1, TPI1, ENO1, ENO3, PGAM1, PKM, GAPDH, FBP1, ALDOA |
| Telomerase Signaling | 0.019498 | 0.24 | RAF1, PIK3R1, SOS2, PIK3R5, PPP2R3B, HRAS, PDPK1, KRAS, PTGES3, HSP90B1, PIK3C3, PIK3CG, SOS1, ATM, IL2RG, AKT2, HDAC4, RRAS, ELF1, PPP2R1A, ELF2, HDAC3, PPP2R2B, HSP90AA1, IL2RA |
| Crosstalk between Dendritic Cells and Natural Killer Cells | 0.020893 | 0.23 | IL2RG, IL3RA, HLA-A, KLRD1, NCR3, HLA-DRB1, LTB, TLN1, ACTG1, CSF2RB, TLR4, PRF1, HLA-DRB4, ICAM3, HLA-DRB3, HLA-DRA, HLA-B, HLA-F, TNF, ACTA1, CAMK2B, CAMK2G |
| Erythropoietin Signaling | 0.021380 | 0.23 | RAF1, PTPN6, AKT2, RRAS, PIK3R1, SOS2, PIK3R5, HRAS, PDPK1, KRAS, CBL, PIK3CG, PIK3C3, SOS1, NFKBIB, STAT5B, ATM, PRKCB |
| Fc Epsilon RI Signaling | 0.021878 | 0.22 | MAP2K4, FYN, RAF1, PIK3R1, SOS2, PIK3R5, HRAS, PDPK1, KRAS, PIK3C3, PIK3CG, SOS1, ATM, AKT2, RRAS, PLA2G4C, RAC1, MAPK9, RAC3, MAPK14, PTPN11, FCER1G, VAV1, TNF, LCP2, PRKCB |
| Pancreatic Adenocarcinoma Signaling | 0.021878 | 0.22 | MAP2K4, RAF1, TGFBR1, BAD, SUV39H1, PIK3R1, PIK3R5, KRAS, E2F3, CDC42, VEGFA, TGFBR2, TGFB1, PIK3CG, PIK3C3, ATM, SMAD2, AKT2, PLD3, TYK2, RAC1, MAPK9, STAT3, PTGS2, CDKN1B, CDK2 |
| PDGF Signaling | 0.022909 | 0.24 | MAP2K4, RAF1, RRAS, PIK3R1, CRKL, TYK2, SOS2, PIK3R5, HRAS, CRK, KRAS, STAT3, SPHK2, PIK3CG, PIK3C3, SOS1, SPHK1, CSNK2A1, ATM, PRKCB |
| Glutaryl-CoA Degradation | 0.023442 | 0.21 | HSD17B10, HADHB, ACAT1, HSD17B4, HSD17B8 |
| Dendritic Cell Maturation | 0.023988 | 0.19 | MAP2K4, HLA-A, PDIA3, PIK3R1, PIK3R5, LTB, HLA-DRB1, IGHG1, CREB5, PLCH2, IKBKG, IGHG3, PIK3CG, HLA-DRB3, PIK3C3, HLA-DRA, HLA-B, PLCL1, CD1C, NFKBIB, COL18A1, PLCD4, ATM, MAP3K14, AKT2, RELB, CREB3, MAPK9, CD58, IKBKE, PLCL2, ATF2, TLR2, TLR4, MAPK14, HLA-DRB4, IGHG4, FCER1G, TNF, IFNAR1 |
| IL-17A Signaling in Airway Cells | 0.023988 | 0.24 | MAP2K4, AKT2, PIK3R1, TYK2, PIK3R5, MAPK9, IKBKE, STAT3, CXCL5, IL17RA, IKBKG, MAPK14, MAP3K7, PIK3C3, PIK3CG, NFKBIB, ATM |
| Gα12/13 Signaling | 0.024547 | 0.21 | MAP2K4, RAF1, PTK2B, PIK3R1, TBXA2R, PIK3R5, HRAS, ARHGEF1, KRAS, MAP3K5, CDC42, IKBKG, F2RL1, LPAR2, PIK3CG, PIK3C3, GNA13, NFKBIB, ATM, AKT2, RRAS, MEF2A, MAPK9, IKBKE, ROCK1, MYL12A, VAV1 |
| Role of PKR in Interferon Induction and Antiviral Response | 0.024547 | 0.26 | TRAF3, IKBKG, MAPK14, CASP3, CYCS, MAP3K7, APAF1, IKBKE, CASP8, NFKBIB, TNF, ATF2 |
| GDNF Family Ligand-Receptor Interactions | 0.025119 | 0.25 | MAP2K4, RAF1, RRAS, DOK2, PIK3R1, SOS2, PIK3R5, RAC1, HRAS, MAPK9, KRAS, CDC42, PDLIM7, PIK3CG, PIK3C3, SOS1, DOK1, ATM |
| Macropinocytosis Signaling | 0.025119 | 0.24 | RAB5A, RRAS, PIK3R1, USP6NL, RAC1, PIK3R5, HRAS, ITGA5, KRAS, CDC42, CSF1R, ITGB7, ARF6, ABI1, PIK3C3, PIK3CG, ATM, PRKCB |
| Role of IL-17A in Arthritis | 0.025704 | 0.24 | MAP2K4, IL8, PIK3R1, PIK3R5, MAPK9, CXCL5, CCL5, IL17RA, ATF2, MAPK14, PIK3CG, PIK3C3, PTGS2, NFKBIB, ATM |
| Signaling by Rho Family GTPases | 0.025704 | 0.19 | MAP2K4, RAF1, MAP3K11, SEPT9, PIK3R1, ARPC5, PIKFYVE, ARHGEF1, PARD6A, MAP3K10, GNB4, RHOG, GNA15, EZR, PIK3CG, BAIAP2, GNA13, ACTA1, ATM, ACTR2, RAC1, ITGA5, PKN1, PIP5K1A, MYL12A, RHOQ, PPP1R12A, FNBP1, PPP1R12C, PTK2B, RHOT2, PIK3R5, CDC42, IQGAP1, ACTR3, ARFIP2, PIK3C3, ARHGEF3, PAK4, PAK2, ARPC5L, GNAQ, MAPK9, VIM, CDC42EP3, SEPT1, ACTG1, ROCK1, WAS |
| Isoleucine Degradation I | 0.026303 | 0.20 | HSD17B10, HADHB, ECHS1, BCAT2, ACAD8, ACAT1 |
| Reelin Signaling in Neurons | 0.026303 | 0.24 | MAP2K4, FYN, MAP3K11, CRKL, PIK3R1, PIK3R5, ITGA5, MAPK9, ARHGEF1, CDK5R1, BLK, MAP3K10, LCK, CDK5, PIK3CG, PIK3C3, MAP4K1, ARHGEF3, ATM, PAFAH1B3 |
| TGF-β Signaling | 0.026915 | 0.24 | MAP2K4, SMAD2, RAF1, TGFBR1, FOXH1, RRAS, SOS2, HRAS, MAPK9, KRAS, ACVR2B, ACVR1B, SMURF1, TGFBR2, MAPK14, PIAS4, MAP3K7, TGFB1, RUNX2, SOS1, MAP4K1 |
| Flavin Biosynthesis IV (Mammalian) | 0.027542 | 0.25 | FLAD1, RFK |
| Glutamine Biosynthesis I | 0.027542 | 0.25 | CCDC92, GLUL |
| Glutathione Redox Reactions II | 0.027542 | 0.29 | GSR, GLRX |
| Hypusine Biosynthesis | 0.027542 | 0.60 | DHPS, EIF5A, DOHH |
| NADH Repair | 0.027542 | 0.22 | APOA1BP, GAPDH |
| AMPK Signaling | 0.029512 | 0.18 | CAB39, TSC1, PRKAB2, PIK3R1, PIK3R5, SMARCD2, PDPK1, PPP2R3B, SMARCA4, PPM1D, EIF4EBP1, MTOR, PIK3CG, FASN, PIK3C3, PRKAA1, PPM1A, ATM, AKT2, PPM1G, PFKFB2, PPP2R1A, MAPK14, PRKAR2B, SMARCA2, PPM1B, PPP2R2B, CHRNE, HMGCR, PRKAR1A |
| HER-2 Signaling in Breast Cancer | 0.029512 | 0.24 | TSC1, AKT2, BAD, RRAS, PIK3R1, SOS2, PIK3R5, HRAS, KRAS, MAP3K5, CDC42, PARD6A, ITGB7, PIK3C3, PIK3CG, SOS1, CDKN1B, ATM, PRKCB |
| Leptin Signaling in Obesity | 0.029512 | 0.23 | AKT2, PDIA3, PIK3R1, PIK3R5, POMC, PLCL2, STAT3, PLCH2, PRKAR2B, PTPN11, PDE3B, PIK3CG, PIK3C3, AGRP, PLCL1, ADCY7, PLCD4, PRKAR1A, ATM |
| Thrombopoietin Signaling | 0.030200 | 0.24 | GAB2, RAF1, RRAS, PIK3R1, PIK3R5, HRAS, KRAS, STAT3, PTPN11, PIK3CG, PIK3C3, SOS1, STAT5B, ATM, PRKCB |
| Role of BRCA1 in DNA Damage Response | 0.030903 | 0.25 | RBL2, FAM175A, C17orf70, ATF1, FANCG, SMARCD2, BRCC3, RBL1, E2F3, SMARCA4, NBN, SMARCA2, RFC2, SLC19A1, BLM, ATM |
| TNFR2 Signaling | 0.031623 | 0.27 | MAP2K4, TANK, MAP3K14, IKBKG, TNFAIP3, IKBKE, NFKBIB, BIRC3, TNF |
| Gluconeogenesis I | 0.033884 | 0.16 | PGK1, ENO1, ENO3, PGAM1, GAPDH, FBP1, ALDOA, MDH1 |
| 2-oxobutanoate Degradation I | 0.034674 | 0.18 | PCCA, PCCB, MUT |
| Activation of IRF by Cytosolic Pattern Recognition Receptors | 0.036308 | 0.22 | MAP2K4, TRAF3, PPIB, MAPK9, IKBKE, IRF3, ATF2, TANK, IRF7, IKBKG, RIPK1, IKBKAP, PIN1, NFKBIB, TNF, IFNAR1 |
| RAN Signaling | 0.036308 | 0.25 | KPNA3, KPNA4, CSE1L, RANGAP1, RAN, RANBP1 |
| FGF Signaling | 0.038019 | 0.23 | RAF1, PTPN6, AKT2, CRKL, PIK3R1, CREB3, SOS2, RAC1, PIK3R5, HRAS, CRK, MAP3K5, STAT3, CREB5, ATF2, MAPK14, PTPN11, PIK3C3, PIK3CG, SOS1, ATM |
| Protein Kinase A Signaling | 0.038019 | 0.18 | RAF1, TGFBR1, BAD, PTPN23, CREB5, PLCH2, TGFBR2, PTPRC, GNB4, DUSP3, TGFB1, PPP1R7, TDP2, GNA13, ANAPC11, NFKBIB, PPP1CA, PLCL1, PTPN6, HIST1H1E, CREB3, PTPN18, PPP1R14A, PDE4B, PLCL2, PTP4A1, ATF2, PDE8A, MYL12A, PTPN11, EYA3, ANAPC5, PTPRN2, PPP1R12A, SIRPA, CAMK2G, RAP1B, PPP1CC, FLNB, PTK2B, PDE7A, TNNI2, PDIA3, DUSP6, PTPN12, DUSP2, PHKA2, TIMM50, PTPRJ, PDE3B, FLNA, H1FX, MTM1, RYR1, PLCD4, CAMK2B, ATF1, PTP4A2, RYR2, GNAQ, PYGB, ROCK1, PPP1R3D, PRKAR2B, ADD3, CDC14B, AKAP10, ADD1, PTGS2, AKAP9, ADCY7, TCF7L2, PRKAR1A, DUSP16, PRKCB |
| Inhibition of Angiogenesis by TSP1 | 0.043652 | 0.26 | MAP2K4, VEGFA, TGFBR2, FYN, AKT2, MAPK14, TGFBR1, CASP3, TGFB1, MAPK9 |
| Fatty Acid β-oxidation I | 0.047863 | 0.20 | HSD17B10, HADHB, ECHS1, ECI2, HSD17B4, SLC27A4, ACAA2, ECI1, HSD17B8 |

**Table S7**. Upstream regulators predicted to be activated or inhibited 7 days (A) or 6 months (B) after LVAD implant by Ingenuity’s Upstream Regulators analysis and confirmed by RNA sequencing. A p-value is generated based on overlap between genes in the dataset and known targets affected by an upstream regulator, and a z-score is used to infer likely activation states of a regulator based on a model of known downstream effects. Observed fold change is included with * indicating statistical significance (FDR < 0.10).

**A.** 7 days post LVAD

| **Upstream Regulator** | **Fold Change** | **Molecule Type** | **Predicted Activation State** | **Activation z-score** | **p-value of overlap** | **Target molecules in dataset** |
| --- | --- | --- | --- | --- | --- | --- |
| **Activated** | | | | | | |
| GATA1 | 2.5* | transcription regulator | Activated | 2.654 | 0.00026 | ABCB10, AHSP, ALAS2, ANK1, BCL2L1, BTG2, CDK4, CDKN2C, EPB42, FLI1, GATA1, GATA3, HBD, KLF1, NFE2, RNASE2, SOX6, SPTB, TFR2, TFRC, TGM2 |
| CEBPE | 2.8* | transcription regulator | Activated | 2.474 | 0.0025 | BCL2L1, CAMP, CDK4, CEACAM8, ELANE, ITGAM, LCN2, LTF, MMP8 |
| EPO | 1.4 | cytokine | Activated | 2.643 | 0.0000000 | ABCA13, AHSP, ALAS2, ANK1, AOC2, BCL2L1, BLVRB, BNIP3L, BZW2, CA1, CA2, CHIT1, CREG1, CTSK, CUL1, DES, DYRK3, FECH, FOSB, GATA1, GATA3, GSPT1, H2AFY, HBD, HK3, HLA-DRB1, HNRNPF, ILF2, KAT2B, KLF1, KPNB1, MLH1, MPO, NFE2, PDCD4, RELA, RHAG, SMARCD2, SRGN, STRADB, TFRC, TP53 TXNIP, VEGFA |
| CSF3 | 2.7 | cytokine | Activated | 3.894 | 0.0000001 | BCL2L1, CD177, CDK4, CEACAM8, CEBPE, CX3CR1, DES, DUSP6, ELANE, FUS, GATA1, GNLY, HLA-DPA1, HLA-DQA1, HLA-DRA, HLA-DRB1, ITGAM, LCN2, LTA, LTF, MMP8, MPO, ODC1, PRTN3, PYHIN1, TFDP1, TFRC, TP53 |
| MAPK1 | 1.3 | kinase | Activated | 2.573 | 0.000020 | AIM1, CSGALNACT2, DDX58, E2F1, ENO3, FAM20A, FHL2, GATA3, GBP1, GBP5, HERC5, IFI16, IFI35, IFIH1, IFIT1, IFIT2, IFIT3, IFIT5, ITGAV, LAP3, LGALS3, MAOB, MAPK13, OAS2, PDGFB, PROS1, PSME2, RGS16, SAMHD1, SFRP2, SMARCC1, STAT1, TBX21, TP53, TRIM5, UBE2C, UBE2L6, ZC3HAV1 |
| SOCS1 | 1.6 | other | Activated | 3.513 | 0.000399 | BCL2L1, CDK4, CXCL10, DDX58, GBP5, IFIH1, IFIT1, IFIT1B, IFIT2, IFIT3, IFNAR1, IL2RB, IRF1, MAP3K5, OAS2, STAT1, STAT5A |
| TGFB1 | 1.2 | growth factor | Activated | 2.582 | 0.00065 | ABCE1, ACP5, ACSM3, ALDH18A1, ALDH5A1, ARL4A, ASGR2, BCL2L1, BNIP3L, BSG, CAMP, CAPRIN1, CCR5, CDK4, CDKN2C, CLIC4, COL6A2, CTSK, CXCL10, CYB561, DACH1, DAXX, DDX21, DES, DYNLL1, E2F1, EHMT2, EIF4A3, EIF4H, ELMO1, EMILIN1, EOMES, EPB49, FLI1, FNBP1, FOSB, FSTL3, FUS, GATA1, GATA3, GBP1, GUSB, GZMA, HDGF, HEBP1, HEXIM1, HLA-DQA1, HLA-DRB1, HNRNPC, HSPA5, IFI16, IFIH1, IFIT3, IGFBP4, IL2RB, INHBA, IRF1, ITGAL, ITGAM, ITGAV, KLF4, KPNA3, LCN2, LGALS3, LRBA, LTA4H, MAOA, MAPK13, MAPK8IP3, MARK3, MEF2C, MGAT3, MPZ, MRFAP1L1, MSN, MXD4, MXI1, MYCN, NMNAT2, OLR1, PATZ1, PCOLCE2, PDE4D, PDGFB, PDHB, PDZK1IP1, PINK1, PKIG, PLAGL2, PNP, PPID, PRPS1, RAD21, RGCC, ROBO3, RPA2, RUNX3, SAE1, SDC1, SERPINH1, SLAMF1, SLC2A1, SRR, STAT1, STK16, STOML2, TBX21, TCF12, TFRC, TGM2, TP53, TPM1, TPST2, TTC22, TUBB2A, TXNIP, USP25, VEGFA |
| RUNX1 | 1.2 | transcription regulator | Activated | 2.137 | 0.0015 | ACHE, BTG2, CD4, GATA3, ITGAL, KRT1, MMP8, MPO, NFE2, RGCC, SLC22A4, SOX6, VEGFA |
| **Inhibited** | | | | | | |
| STAT1 | -1.7* | transcription regulator | Inhibited | -2.521 | 0.0116 | ALAS2, BATF2, BCL2L1, CXCL10, DPP4, GATA3, GBP1, GBP5, HLA-DRB1, IFI35, IFIT1, IFIT1B, IFIT2, IRF1, NFE2, PRF1, PSME2, SAMHD1, SERPING1, STAT1, TBX21, TP53, TRAFD1, WARS |
| CD2 | -1.6* | receptor | Inhibited | -2.804 | 0.00252 | CD4, CD40LG, CD8A, HLA-DPA1, ITGAL, STAT1, STAT4, TP53 |

**B.** 6 months post LVAD

| Upstream Regulator | Fold Change | Molecule Type | Predicted Activation State | Activation z-score | p-value of overlap | Target molecules in dataset |
| --- | --- | --- | --- | --- | --- | --- |
| **Activated** | | | | | | |
| IL4 | 1.1 | cytokine | Activated | 2.599 | 0.0024 | ABCA2, ACOX1, ADAM19, ADRM1, ALG3, ALOX5, ANXA2, ANXA6, APRT, ARMC6, BAX, BCAT2, BCL11A, BIN1, BOP1, CAPG, CASP3, CBX3, CCL5, CCR4, CCT3, CD1C, CD2, CD58, CD79A, CDC42, CDK1, CDKN1B, CELF2, CLEC7A, CMAHP, COL18A1, COL6A2, CSF2RB, CST7, CTNNA1, CXCR3, CYSLTR1, CYSLTR2, DCTN1, DECR1, EBNA1BP2, EGLN1, EIF3G, F2RL1, FCER1G, FCER2, FLT3LG, FPR2, FUT7, GNA15, GPT, GZMB, HCLS1, HDDC2, HK2, HMGCR, HSPA1A/HSPA1B, ICOS, IFI30, IFNAR1, IGHG1, IGHG3, IGHG4, IL12RB1, IL27RA, IL2RA, IL3RA, IL7R, IL8, IRF7, ITGB7, ITPA, JUND, KCNAB2, KIAA0664, KLRD1, LAS1L, LGALS3BP, LIG4, LMAN2, LTB, MAPKAPK3, MATK, MCL1, MEFV, MOV10, MTDH, NCL, NOC2L, OBFC2A, PDCD1, PDE4B, PDK1, PET112, PHB, PIN1, PKP3, PLA2G4C, PLIN2, PMVK, POU2AF1, PPM1G, PRDM1, PREPL, PRF1, PRKDC, PRMT1, PRNP, PSMB10, PSMB8, PSME1, PSTPIP1, PTGS2, RAB5A, RNF5, S100A10, SDF2L1, SELP, SELPLG, SERPINB6, SIRPA, SNRPA, SPHK2, SRM, SYMPK, SYNGR2, TBX21, TGFB1, THOP1, TICAM1, TIMP1, TLR2, TLR4, TMED5, TNF, TNFRSF4, TNFRSF9, TNFSF4, TRAF3, TXK, UQCRC1, VIM, XPO7, XRCC5, XRCC6, ZFP36 |
| STAT4 | 1.3 | transcription regulator | Activated | 2.007 | 0.046 | ACAP1, ACSS1, AHNAK, AKAP8L, BAD, BCL2L11, C10orf54, CDK2, CDKN1B, CIR1, COPG1, DPM3, FCER1G, FYB, GLG1, GTF2E2, HCFC2, HEXB, HSPA1A/HSPA1B, IL12RB1, IL2RA, ING2, KCNK7, KLF9, LAMTOR3, LTBP3, LUC7L, MAGED1, MAP3K8, MATK, MBOAT2, PDK1, RAMP1, RCN3, RNF138, SELPLG, SERTAD1, SETD5, SH2B1, STK32C, TBX21, TMEM167B, TNF, VEGFA, ZNF394, ZNF524 |
| PAX5 | 1.8 | transcription regulator | Activated | 2.25 | 0.047 | BLK, BLNK, CD19, CD72, CD79A, CSF1R, FCER2, POU2AF1, PRDM1 |
| CD38 | 1.1 | enzyme | Activated | 2.206 | 0.049 | ALDOA, ANXA2, ANXA6, CCDC164, CHST12, CISD1, CRELD2, CSF2RB, EGLN1, EIF4EBP1, ENDOG, GALK1, IFI30, IGHG1, IL2RA, IL6R, ITGB7, KCNAB2, MANF, MTDH, OBFC2A, PDK1, PGM1, PRDM1, PRDX4, RASGRP2, RPN1, S100A4, SDF2L1, SLC16A3, SPCS2, STK39, TMED5, TPI1, VIM |

Table S8. TaqMan Gene Expression Assays used for RTQPCR confirmation of selected genes.

| Gene | Taqman Gene Expression Assay |
| --- | --- |
| CA-1 | Hs01100176_m1 |
| ABCG2 | Hs01053790_m1 |
| AHSP | Hs00372339_g1 |
| RAP1B | Hs04275955_g1 |
| SELL | Hs00174151_m1 |
| GAB1 | Ha00157646_m1 |
| MTPN | Hs00377581_m1 |
| KLF2 | Hs00360439_g1 |
| TMSB4X | Hs03407480_gH |
| NOSIP | Hs00211028_m1 |
| TXNIP | Hs01006900_g1 |
| BAG6 (BAT3) | Hs00190383_m1 |
| BNIP3L | Hs01087963_m1 |
| IL16 | Hs00189606_m1 |
| SCAMP2 | Hs00194730_m1 |
| SP1 | Hs00916521_m1 |
| TLR4 | Hs00152939_m1 |
| HLA-DRA | Hs00219575_m1 |
| PRF1 | Hs00169473_m1 |
| CD4 | Hs01058407_m1 |
| PDIA3 | Hs00607126_m1 |
| CD2 | Hs00233515_m1 |
